# Supplementary material for: Disrupted glucocorticoid receptor cell signalling causes a ciliogenesis defect in the fetal mouse renal tubule
Source: EMBO Rep. 2025 Apr 17;26(11):2883–909. doi: 10.1038/s44319-025-00454-0 (PMC12152183; doi:10.1038/s44319-025-00454-0)
Supplement: Supplementary file 1 — Table EV1 [file 44319_2025_454_MOESM1_ESM.pdf]

Table EV1: RNA-seq data of differentially expressed genes in GR-null kidneys at E18.5

Gene_symbols	logFC	logCPM	F	PValue	FDR
AC131675.2	6.759241	-1.00932	44.46036	1.64E-05	0.001571
G630018N14Rik	-6.7274	-0.84238	35.84086	2.6E-05	0.002117
Gm26596	-5.13045	-0.25716	44.48097	5.66E-06	0.000869
Gm5045	-4.79589	-0.54728	18.91237	0.000508	0.011364
Ifit1	-4.2831	2.521699	117.5653	9.74E-09	1.57E-05
9530056K15Rik	-3.59129	-0.39516	32.2992	3.52E-05	0.002484
Ifi213	-3.4576	0.324608	39.94865	1.06E-05	0.001311
Gm33091	-3.30007	-1.02986	17.00618	0.000811	0.014651
Ifi27l2a	-3.26618	3.253994	28.31661	7.11E-05	0.003666
AC113540.1	-3.19847	0.748105	28.23556	7.22E-05	0.003692
Tgtp1	-3.15176	1.088508	57.75023	1.14E-06	0.000302
Crisp1	-3.14781	2.082563	92.5979	5.1E-08	3.72E-05
Chgb	-3.04637	0.350465	10.18084	0.005743	0.044674
Kap	-3.00075	6.957383	73.59569	2.38E-07	9.39E-05
Oard1	-2.9461	2.519285	24.66191	0.000144	0.005472
Gm4951	-2.84016	-0.14482	28.20354	7.26E-05	0.003694
Gm9523	-2.69745	-0.69779	15.93591	0.001068	0.016876
Slfn4	-2.66493	0.232999	17.41911	0.000731	0.013857
Isg15	-2.64805	3.839791	72.53901	2.62E-07	9.66E-05
Gbp3	-2.61072	3.835127	84.68148	9.35E-08	5.02E-05
Mir10a	-2.58977	0.473408	24.94879	0.000136	0.005266
Gm12749	-2.57845	-0.86543	17.31533	0.00075	0.013967
Slc22a26	-2.5574	1.27511	48.36914	3.42E-06	0.000606
Gm15726	-2.55072	1.858504	26.82731	9.41E-05	0.004193
E030022I16Rik	-2.52737	-0.41169	17.55317	0.000707	0.013592
Phf11d	-2.49921	1.191028	37.14877	1.62E-05	0.001571
Rps12-ps24	-2.42001	-0.47852	11.39177	0.003901	0.035991
Gm4070	-2.40898	-0.05256	18.47337	0.000564	0.011981
Ripply2	-2.4063	0.446526	28.32203	7.11E-05	0.003666
Htr4	-2.39382	-0.67827	13.76554	0.001929	0.023877
Nap1l2	-2.36968	-0.5476	12.24265	0.003007	0.030837
Gm15889	-2.3626	1.121905	45.55309	4.91E-06	0.000802
Defb29	-2.33346	1.770294	38.35007	1.35E-05	0.001411
Oasl2	-2.31527	4.945646	108.4224	1.72E-08	1.9E-05
Rpl36a-ps1	-2.29675	-0.41309	10.07162	0.005953	0.045574
Gm10709	-2.26462	4.355623	97.6058	3.55E-08	3E-05
Oas3	-2.24599	0.091005	16.03791	0.00104	0.016616
Ddx60	-2.2433	2.005434	55.66337	1.44E-06	0.000354
Stfa1	-2.23565	0.389199	19.32648	0.000461	0.010845
Usp18	-2.23471	2.812786	49.19634	3.08E-06	0.00057
Slc17a2	-2.21411	0.079757	18.15653	0.000609	0.012535
Rad9a	2.212308	0.462662	16.968	0.000819	0.014733
Gm15247	-2.20728	1.513747	39.44591	1.15E-05	0.001345
Oas2	-2.20596	2.883816	79.78732	1.39E-07	6.68E-05
Gbp6	-2.20213	1.102473	24.63098	0.000145	0.005483

Gypa	2.198065	0.77617	38.3727	1.34E-05	0.001411
Mx2	-2.18636	1.983073	38.89754	1.24E-05	0.001367
Irf7	-2.18348	4.139507	64.89009	5.42E-07	0.000169
F630028O10Rik	-2.17626	0.157009	18.59334	0.000548	0.011771
H2-Q2	-2.14844	0.583743	17.3606	0.000742	0.013898
Gm14760	2.099986	-0.70179	18.32324	0.000585	0.012249
Mir8116	-2.09456	-0.25733	14.55588	0.001547	0.021149
G530011O06Rik	-2.0873	2.186457	29.40857	5.83E-05	0.003344
Gm16089	-2.08531	-0.25824	17.0821	0.000795	0.014461
Iigp1	-2.05746	3.012155	89.12937	6.61E-08	4.34E-05
BE692007	-2.0549	-0.44525	11.10702	0.004265	0.037882
Gm3788	2.044387	3.6469	202.8203	1.9E-10	3.37E-06
Ahsg	2.02816	3.045631	25.21587	0.000129	0.005076
Nfya	2.025358	1.534889	23.87022	0.000169	0.006069
Mmp8	-2.01697	-0.30122	13.5535	0.002048	0.024773
Acan	2.005967	0.001924	15.22513	0.001289	0.018924
Trim30d	-1.99062	1.09891	27.32896	8.56E-05	0.004034
Plk4	-1.97262	1.382102	41.69882	8.29E-06	0.001122
Zbp1	-1.95505	0.434697	19.43786	0.000449	0.010715
Mndal	-1.93495	3.232278	84.93577	9.16E-08	5.02E-05
Bmp15	-1.93254	-0.67765	10.5195	0.005144	0.041947
Ranbp3l	-1.90976	2.610746	53.03457	1.94E-06	0.000436
Phf11b	-1.90217	-0.01739	13.23014	0.002248	0.026306
Nccrp1	-1.90101	2.525414	71.0542	3E-07	0.000105
Muc3a	-1.89484	-0.54851	11.73784	0.003505	0.03381
Gbp7	-1.88471	3.602237	88.11453	7.15E-08	4.48E-05
Gvin1	-1.85776	-0.34829	13.31105	0.002196	0.025957
Ifi204	-1.84556	1.692881	41.2227	8.87E-06	0.001173
Gm13192	1.838388	1.693054	27.91994	7.66E-05	0.003781
Slc41a2	-1.83359	0.865109	22.6463	0.000219	0.006997
Rps8-ps1	1.833139	1.200552	25.00013	0.000135	0.005223
Meg3	-1.83191	0.678564	15.15866	0.001313	0.019091
Sphkap	-1.82764	-0.34935	12.40825	0.002862	0.030253
Hba-a1	1.825835	9.135222	145.6962	2.12E-09	6.25E-06
Gm3226	1.819283	0.556649	20.50061	0.000351	0.009404
Sv2c	-1.81743	0.082453	10.91484	0.004532	0.039078
Gm6170	-1.81437	0.229818	10.6819	0.004882	0.04054
Gm14858	-1.81254	-0.75828	9.759495	0.006603	0.048359
Chac1	1.806239	0.436038	12.54548	0.002747	0.029564
Gbp5	-1.80584	0.167603	12.08185	0.003157	0.031994
Rbp2	1.801875	1.341373	38.68584	1.28E-05	0.001394
Cyp1a1	-1.79404	-0.07963	12.13743	0.003104	0.031629
Gm44349	-1.79189	0.282765	11.9881	0.003248	0.032411
Cbx3-ps7	-1.78461	2.523659	70.77444	3.08E-07	0.000105
Gm11221	-1.78287	1.513985	28.08221	7.43E-05	0.0037
Gm12174	-1.77575	3.694382	84.2308	9.69E-08	5.05E-05
Trim30a	-1.76777	3.278232	101.7325	2.67E-08	2.63E-05

Slc10a2	-1.74827	2.508444	74.13052	2.27E-07	9.37E-05
Gbp4	-1.7455	1.799431	40.1212	1.04E-05	0.001287
Slc4a10	-1.74534	0.00712	14.08715	0.001762	0.022795
Syt4	-1.72389	0.895769	10.96797	0.004456	0.038619
AC121967.1	-1.71662	2.066215	36.79481	1.71E-05	0.001571
Gm13841	-1.71475	5.268833	173.6617	5.93E-10	4.05E-06
1700023H06Rik	-1.71428	-0.47808	11.04921	0.004343	0.038255
Gm20400	-1.70324	0.343552	18.20109	0.000603	0.012513
Gm47283	-1.70038	2.729631	23.5318	0.000182	0.006331
Sp100	-1.696	4.210684	75.848	1.95E-07	8.45E-05
Gm7862	-1.69598	0.530065	13.07397	0.002352	0.026766
Gabrg1	-1.68252	0.090914	12.01212	0.003224	0.032317
Alb	1.679759	5.090802	11.00648	0.004402	0.038395
Ifi44	-1.67918	4.629149	109.856	1.57E-08	1.85E-05
Slc14a2	-1.65992	4.04879	45.45064	4.98E-06	0.000802
Ifi208	-1.65405	0.406935	16.22607	0.00099	0.016146
Zfp982	-1.65131	0.426512	16.13753	0.001013	0.016387
Bst1	-1.64794	1.840071	34.38738	2.49E-05	0.002056
Gm11808	-1.64481	6.943755	121.9143	7.54E-09	1.34E-05
Rpl30-ps10	-1.638	3.251086	13.53546	0.002059	0.024867
C7	-1.63279	0.191799	11.84153	0.003396	0.0333
Sycp3	-1.63026	0.103582	10.47971	0.005211	0.042182
Hbb-bs	1.627041	9.176868	170.2532	6.85E-10	4.05E-06
Ifi203	-1.62702	3.442988	68.41429	3.85E-07	0.000124
Gm10226	-1.6162	0.031056	11.89341	0.003343	0.032985
Gm7392	-1.6146	0.04282	12.29496	0.00296	0.030709
Ifi209	-1.61278	0.199586	12.96847	0.002426	0.027244
Trim5	-1.58069	0.332152	10.88645	0.004573	0.039177
Oas1a	-1.57221	2.240044	28.86052	6.44E-05	0.003561
Xaf1	-1.56643	3.834791	60.33099	8.65E-07	0.000243
Rtp4	-1.55673	3.99558	81.77415	1.18E-07	5.99E-05
4930413G21Rik	-1.54919	1.694696	22.10895	0.000246	0.007517
Spink8	-1.5488	3.80051	85.85209	8.52E-08	4.87E-05
Gm853	-1.52786	4.150964	123.7731	6.77E-09	1.33E-05
AU015836	-1.52568	0.888409	20.26798	0.00037	0.009594
Ifi206	-1.52458	0.353125	14.49223	0.001574	0.021287
Gm26775	-1.51957	-0.34819	9.840489	0.006427	0.047677
P2ry12	-1.51231	-0.10576	10.36137	0.005415	0.043066
Gm38393	-1.5109	2.151721	21.85299	0.00026	0.007776
Msc	-1.50547	0.691086	15.21201	0.001294	0.018959
Mid1	-1.48809	5.146358	15.4007	0.00123	0.018282
Rps16	-1.48131	7.037588	100.1185	2.98E-08	2.78E-05
Gm11539	-1.47661	0.888098	16.72964	0.00087	0.015226
S100g	-1.47369	8.123206	99.29613	3.16E-08	2.8E-05
Mrps23	1.464934	0.726804	13.89285	0.00186	0.023418
Naaladl2	-1.46057	3.058705	44.85879	5.38E-06	0.000852
Gm17828	1.458276	2.852764	22.58782	0.000222	0.007048

Gm3055	-1.45452	0.146519	13.45428	0.002107	0.02528
Gm36266	-1.45305	1.547837	26.9242	9.24E-05	0.004189
Clec7a	-1.44894	-0.1863	9.964268	0.006168	0.046319
Pip5kl1	1.447566	0.828331	16.50255	0.000922	0.015579
Grem2	-1.4426	2.983593	39.74141	1.1E-05	0.001332
mt-Nd6	-1.44085	7.648031	60.52148	8.48E-07	0.000243
Gm38372	-1.43892	1.381712	16.55188	0.00091	0.015545
Socs2	-1.43797	5.072104	151.1616	1.62E-09	6.25E-06
Agt	-1.41488	2.835794	29.67498	5.56E-05	0.003283
S100a8	-1.41128	1.493241	16.84546	0.000844	0.015
Trp53-ps	1.411265	1.024863	17.84523	0.000658	0.013064
Gm26532	1.395767	1.910026	41.93357	8.02E-06	0.001094
Cep290	-1.39484	4.145204	78.86486	1.51E-07	7.03E-05
Alas2	1.388428	5.01334	95.2798	4.19E-08	3.38E-05
Zfp469	1.384556	3.161289	33.52482	2.87E-05	0.002212
Gm16418	-1.38015	1.926289	18.03654	0.000627	0.012757
Ifih1	-1.37121	3.685917	77.95854	1.63E-07	7.4E-05
Gm45441	1.360865	-0.10986	10.93829	0.004499	0.038843
Defb1	-1.35896	5.205287	80.92963	1.27E-07	6.24E-05
C1ql4	-1.35303	1.303126	19.34886	0.000458	0.010831
Gm44686	-1.34984	0.61606	9.667057	0.006811	0.049262
Lsm5	-1.34955	1.290616	19.13353	0.000482	0.011039
Gbp2	-1.34221	1.635634	13.61176	0.002015	0.024566
Hnmt	-1.33978	0.972437	13.77429	0.001924	0.023851
Oas1b	-1.33616	2.738078	31.10701	4.32E-05	0.002804
Ifit3	-1.33579	4.101485	50.0477	2.78E-06	0.000539
1700016C15Rik	-1.33404	0.718955	11.23417	0.004098	0.037048
Gm14399	-1.32481	1.974592	22.05851	0.000249	0.007561
Rpl7a-ps3	1.322404	0.598001	12.35468	0.002908	0.030423
Ugt3a2	-1.32229	3.679938	55.8249	1.41E-06	0.000353
Lrrc4c	-1.31896	0.800839	14.41499	0.001608	0.02149
Gm6180	-1.31676	1.518051	22.19936	0.000241	0.007423
Snhg14	-1.31617	3.458857	40.51259	9.81E-06	0.001235
Higd1b	-1.31246	0.0899	10.32516	0.005479	0.043324
Rpl36-ps12	-1.30825	4.003782	51.75813	2.26E-06	0.000471
Gm12963	-1.30793	0.380102	11.45664	0.003823	0.035563
Igf2	1.302241	6.844098	113.2036	1.27E-08	1.85E-05
Dok6	-1.30123	1.813181	21.76069	0.000265	0.007852
Gabra2	-1.30059	0.822915	12.244	0.003006	0.030837
Hbb-bt	1.295995	6.701385	135.9164	3.48E-09	8.82E-06
Gm15421	-1.29564	1.550665	21.45773	0.000284	0.008134
A730063M14Rik	-1.29474	0.322798	10.50007	0.005176	0.042012
Tldc2	-1.29367	0.927351	10.43942	0.005279	0.042407
Cdv3-ps	-1.29202	1.211393	15.22561	0.001289	0.018924
Gm12338	-1.28945	3.816659	73.80512	2.34E-07	9.39E-05
Herc6	-1.28807	2.952324	30.62672	4.69E-05	0.002941
Chrnb4	1.287233	2.683498	57.92884	1.12E-06	0.000301

Grik3	1.281011	1.256248	9.997458	0.006101	0.045988
Gm12070	1.273493	1.4744	21.7064	0.000268	0.00788
Gm29770	-1.27345	0.70517	13.31114	0.002196	0.025957
Cp	-1.2722	3.078155	55.87052	1.4E-06	0.000353
Cyp27b1	-1.26044	6.895068	49.74911	2.88E-06	0.000543
Gm8942	-1.2593	2.612414	23.15802	0.000197	0.006594
Aqp6	-1.25603	3.620872	32.28244	3.53E-05	0.002484
Gm8730	-1.25331	6.960493	110.9171	1.46E-08	1.85E-05
Tma7-ps	-1.2449	3.729024	52.15368	2.16E-06	0.000461
Gm12264	-1.24157	1.19131	13.38225	0.002151	0.025583
Gm10705	-1.23551	3.208134	13.13384	0.002312	0.026628
Map7d3	-1.23462	1.364019	13.57688	0.002035	0.024692
Nr1i2	1.230119	1.007053	15.14522	0.001317	0.019098
D630023F18Rik	1.226374	1.078385	16.42569	0.00094	0.015724
Gm12966	-1.22605	4.426822	87.79315	7.33E-08	4.48E-05
Hmgn2	-1.22506	7.580867	106.1412	1.99E-08	2.08E-05
Pof1b	-1.22411	0.531629	10.5326	0.005122	0.041895
Zfp947	-1.22336	1.558432	15.91065	0.001075	0.016954
Slc7a12	-1.22013	4.288319	25.48931	0.000122	0.004955
Gm8995	-1.21748	2.618562	28.34473	7.08E-05	0.003666
Lgals3bp	-1.20976	5.751887	37.23878	1.59E-05	0.001561
Gm43031	-1.20678	1.250903	13.88257	0.001866	0.023418
Gm12138	-1.20201	1.47514	16.3539	0.000958	0.015869
Gm10269	-1.19623	2.072244	15.52965	0.001189	0.017996
Aard	-1.19578	1.341835	11.45246	0.003828	0.03559
Hsd3b7	1.185165	0.267687	10.84386	0.004636	0.039436
Psmb8	-1.17574	2.788786	26.1869	0.000107	0.004561
9930017N22Rik	-1.17552	1.022454	11.94785	0.003288	0.032599
Rps26-ps1	-1.17492	4.95135	17.25891	0.000761	0.014122
Epsti1	-1.17447	1.602329	18.08517	0.00062	0.012694
Gm36401	-1.1729	1.415815	13.23082	0.002247	0.026306
Guca2b	-1.17245	1.824877	17.02039	0.000808	0.014628
Siglec1	-1.16801	1.698515	19.02007	0.000495	0.011183
Oasl1	-1.16578	2.224499	15.18027	0.001305	0.019027
Fyb2	-1.16162	0.644353	10.95461	0.004475	0.038704
Defb19	-1.15934	3.193847	33.56796	2.85E-05	0.002208
Pin4	-1.15571	1.020431	10.71809	0.004826	0.040204
Gm23935	1.154662	7.208114	50.72466	2.56E-06	0.000515
Tafa1	-1.15318	0.820633	11.96241	0.003273	0.032565
Atp5l	-1.15262	4.69461	92.19447	5.25E-08	3.72E-05
Zfp157	-1.15222	5.058897	110.1748	1.53E-08	1.85E-05
Slc5a2	1.1496	4.600954	55.22227	1.51E-06	0.000357
Tgm5	1.148846	1.69382	15.93516	0.001068	0.016876
Eno1b	1.137543	5.656581	148.1194	1.88E-09	6.25E-06
B830017H08Rik	-1.12771	1.380395	10.15951	0.005784	0.044856
Samd9l	-1.12655	5.49576	63.47571	6.25E-07	0.000185
Tmigd1	-1.12205	3.102483	29.32087	5.92E-05	0.003386

Gm14567	-1.12127	2.185614	17.24558	0.000763	0.014125
Gm43667	-1.1193	1.66426	15.56733	0.001177	0.017881
Hsd3b4	-1.11698	2.577886	11.94702	0.003289	0.032599
Zfp992	-1.1168	1.453559	10.99204	0.004423	0.038529
Gbp9	-1.11289	4.237978	36.33119	1.83E-05	0.001632
Prdm6	1.109999	0.508423	10.11586	0.005867	0.045188
Fgl1	-1.10784	1.045058	10.05129	0.005993	0.045684
H2-T23	-1.10453	3.81697	28.8515	6.45E-05	0.003561
Gm2999	-1.10407	2.812216	29.92657	5.31E-05	0.003203
Nkx3-1	1.101085	3.015706	41.98881	7.96E-06	0.001094
Gm37303	-1.10013	1.191064	13.14406	0.002305	0.026596
Egfl6	-1.09816	4.060354	30.07811	5.17E-05	0.003129
Xlr3a	1.091051	1.050057	10.20772	0.005693	0.044483
Gm26566	-1.08897	1.080459	10.39644	0.005353	0.042751
Ccdc116	1.088901	0.559734	11.07413	0.004309	0.038124
Fgl2	-1.08735	3.766013	29.49533	5.74E-05	0.003325
Lamb3	-1.084	2.086544	13.34581	0.002174	0.025783
Gm10243	-1.08385	5.132242	35.65043	2.04E-05	0.001763
Gm11273	-1.0821	3.558937	34.06922	2.63E-05	0.002119
Gm6485	-1.07122	1.94322	18.71084	0.000533	0.011623
Them7	-1.0707	3.377378	21.83586	0.000261	0.007776
Tspan1	-1.06461	2.323877	25.39556	0.000124	0.00499
Serpina6	-1.06297	7.642361	28.57314	6.78E-05	0.003628
Smim27	-1.06178	1.580219	12.39407	0.002874	0.030257
Pak3	-1.06082	1.280148	11.26908	0.004053	0.036833
Gm12346	-1.06062	2.697712	19.22868	0.000471	0.010923
Luzp2	-1.05982	1.316344	11.27963	0.00404	0.036768
Parp9	-1.05583	4.17728	56.8374	1.26E-06	0.000324
Gm13212	-1.05042	1.542152	16.13049	0.001015	0.016402
Gm43300	-1.04242	2.442721	20.25224	0.000372	0.009594
Ddx58	-1.03563	5.268868	76.45732	1.85E-07	8.21E-05
Ifit3b	-1.03545	2.644687	17.23539	0.000765	0.014138
Gm33035	-1.03443	1.962239	16.2603	0.000981	0.016107
Ifit2	-1.03437	4.685568	87.03074	7.77E-08	4.59E-05
Tmem100	-1.02866	4.264405	72.34275	2.67E-07	9.66E-05
Rtn4rl2	1.025488	1.607576	14.24065	0.001688	0.022261
Itgb8	-1.02398	4.601208	43.53777	6.43E-06	0.00095
Abcg3	-1.0218	1.014784	10.12491	0.00585	0.045093
Stab2	1.021204	1.415248	9.924792	0.00625	0.046692
Gm8399	1.02045	3.430076	28.98787	6.29E-05	0.003534
Lrif1	-1.01993	4.278563	40.18176	1.03E-05	0.001285
9230110F11Rik	-1.01959	3.125347	33.70741	2.79E-05	0.002185
Ntng1	-1.01877	1.888518	15.15661	0.001313	0.019091
Parp14	-1.01788	4.646403	52.20019	2.14E-06	0.000461
Gm12254	-1.01409	3.88413	44.4307	5.7E-06	0.000869
Pld5	1.009573	1.281437	14.67998	0.001495	0.020706
Gpx3	-1.0091	9.251595	27.83461	7.78E-05	0.003818

Tmem56	-1.00603	2.643381	27.04397	9.03E-05	0.004129
Fgfbp3	1.005365	3.291478	20.31008	0.000367	0.009594
Irgm2	-1.00508	3.527683	29.70898	5.52E-05	0.003274
Gm13493	-1.00325	1.256923	10.93743	0.0045	0.038843
Rab27b	-1.00301	2.874825	34.12267	2.6E-05	0.002117
Znf41-ps	-1.00284	2.064514	12.397	0.002871	0.030253
Gm7143	1.001415	2.291077	16.69515	0.000877	0.015235
C3	-1.00109	2.635401	14.50019	0.001571	0.021287
Gm5436	-0.99983	1.692255	15.00473	0.001368	0.01966
Col26a1	0.994919	6.919936	125.0519	6.3E-09	1.33E-05
Gm21399	0.993982	2.417644	24.22416	0.000158	0.005778
Gm37607	-0.98954	1.315578	10.02479	0.006046	0.045769
Slc22a29	-0.98874	3.60027	25.38491	0.000125	0.00499
Calb1	-0.98816	7.6273	93.99892	4.6E-08	3.55E-05
Ifi203-ps	-0.98556	2.051698	17.78	0.000668	0.013164
Stat1	-0.98471	5.350141	55.40005	1.48E-06	0.000357
Zfp820	-0.98279	2.87319	28.47208	6.91E-05	0.003646
Cetn4	-0.98123	1.672356	10.67708	0.004889	0.040564
Gm3362	-0.98038	4.391952	19.99182	0.000395	0.009951
Gm9844	0.979193	3.456334	46.49978	4.34E-06	0.000726
Hist1h2ae	0.975932	1.568588	13.10681	0.00233	0.026703
Ccl11	0.972019	1.895763	19.47992	0.000444	0.010674
Irgm1	-0.97187	3.340096	39.49663	1.14E-05	0.001344
Aqp4	-0.96724	4.981952	54.56686	1.63E-06	0.000375
Gm43158	-0.96559	1.20765	10.37852	0.005384	0.042904
Fgf12	-0.96425	2.233748	14.07919	0.001766	0.022797
Gm7072	-0.96228	4.046105	33.56211	2.85E-05	0.002208
Gm14681	-0.95995	4.555547	48.82533	3.23E-06	0.000584
Crybb1	-0.95845	1.312039	9.991122	0.006114	0.046046
Ptx3	0.952604	3.431104	41.35653	8.7E-06	0.00116
A430033K04Rik	-0.95178	3.701502	45.4657	4.97E-06	0.000802
Chpt1	-0.94931	7.149339	73.08893	2.5E-07	9.61E-05
Gm10080	-0.94903	2.400194	19.91603	0.000402	0.010039
Gm6087	-0.9483	1.4602	9.693649	0.00675	0.049029
Tpi-rs11	0.94439	3.134066	22.70233	0.000216	0.006997
Srp54a	-0.9399	3.054502	21.16077	0.000303	0.008522
Rpl22l1	-0.93599	6.552293	49.77061	2.87E-06	0.000543
Cebpd	-0.93537	4.461841	16.99967	0.000812	0.01466
P4ha3	0.933358	3.473823	37.35723	1.56E-05	0.001554
Gm16185	-0.93295	4.050975	21.92174	0.000256	0.007696
Aqp2	-0.93256	8.364894	31.34886	4.14E-05	0.002719
Spink1	-0.9323	4.030377	34.7787	2.34E-05	0.001977
H3f3a-ps1	-0.93076	2.240123	17.13934	0.000784	0.014342
Tnn	0.929675	2.470311	22.40341	0.000231	0.007191
Elavl4	-0.92763	2.496459	10.88273	0.004579	0.039177
Lcorl	-0.92728	5.373436	50.49986	2.63E-06	0.000524
Gm6472	-0.92709	8.090556	69.61079	3.44E-07	0.000113

Rps27rt	0.925875	6.996234	21.14573	0.000304	0.008537
Rbfox1	-0.92459	2.333604	16.89734	0.000833	0.014897
Gm9824	0.923662	5.600016	10.77124	0.004744	0.039882
Gm42732	-0.92358	2.489862	11.87272	0.003364	0.033132
Proc	-0.92162	2.515971	9.68655	0.006767	0.049085
Cdkl2	-0.92096	2.052495	14.68454	0.001493	0.020696
Rnaset2b	-0.9208	3.951616	34.93258	2.28E-05	0.001945
Dhx58	-0.91819	2.277695	14.57186	0.00154	0.021099
Mt2	0.915931	4.679814	40.81485	9.4E-06	0.001216
Bicd1	-0.91519	3.793823	37.68924	1.49E-05	0.001516
Gm6548	-0.91495	4.31773	52.50822	2.07E-06	0.000453
Proz	0.914254	2.090787	18.02721	0.000629	0.012757
Fam241a	-0.91148	2.647806	20.29169	0.000368	0.009594
Bst2	-0.90921	6.259031	26.53955	9.95E-05	0.004333
Zfp808	-0.90893	2.347789	15.99846	0.00105	0.016703
Fut9	-0.90719	6.243192	58.31086	1.07E-06	0.000293
Esf1	-0.90598	4.835779	36.86278	1.69E-05	0.001571
Trim12c	-0.90591	4.224745	43.00848	6.91E-06	0.000973
Hdc	-0.90584	7.86687	70.67017	3.11E-07	0.000105
Snhg3	-0.90446	1.973871	16.14535	0.001011	0.016379
Glt28d2	-0.90344	1.849055	11.58774	0.003671	0.034803
Pcgf5	-0.90263	3.408553	32.16142	3.6E-05	0.002496
Tap1	-0.90193	3.912343	26.69737	9.65E-05	0.004256
Pcsk9	0.901775	5.180354	70.5944	3.13E-07	0.000105
Adra1d	0.90161	2.272149	14.98505	0.001376	0.019692
Zfp984	-0.90149	3.573968	38.1559	1.39E-05	0.001421
Gdap10	-0.90135	1.807372	12.38726	0.00288	0.030284
H3f3a-ps2	-0.89995	1.444921	9.814559	0.006483	0.047889
Ttr	-0.89993	5.009778	12.38688	0.00288	0.030284
Arhgap5	-0.89938	6.639793	49.40796	3.01E-06	0.000561
Mpv17l	-0.89875	4.043013	34.55701	2.43E-05	0.002026
Ccdc18	-0.89801	2.999544	19.18802	0.000476	0.011014
Fgd4	-0.89381	4.849107	43.08591	6.84E-06	0.00097
Abhd11os	0.893806	1.986179	16.23288	0.000988	0.016146
D030007L05Rik	0.891497	1.454578	10.46927	0.005228	0.042286
Arc	0.890425	1.95362	11.64115	0.003611	0.034416
AU041133	-0.8896	2.260926	15.7763	0.001114	0.017287
Spp1	-0.88844	8.874155	32.61238	3.34E-05	0.002436
Tnfsf10	-0.88637	4.070028	36.30104	1.84E-05	0.001632
Hmox1	0.884028	4.803501	65.63689	5.03E-07	0.000159
Klk1	-0.8831	5.038178	18.67444	0.000538	0.011679
Atp6v1g3	-0.88293	3.813506	33.96748	2.67E-05	0.002119
Eif2ak2	-0.88229	5.077304	53.93733	1.75E-06	0.000398
BC037032	-0.88218	2.249895	14.28864	0.001665	0.0221
H2-Q6	-0.88179	3.10784	16.62332	0.000894	0.015337
Svep1	0.880142	5.280813	48.02841	3.57E-06	0.000627
Aspn	-0.87901	4.45131	39.22573	1.18E-05	0.001361

Sprr2f	0.878945	4.121864	18.10583	0.000617	0.012645
Mkx	-0.87772	1.924943	12.86567	0.0025	0.02776
D3Ertd751e	-0.87598	4.07402	25.14337	0.000131	0.005121
Hist1h3c	0.874979	1.785867	15.46065	0.001211	0.01812
Ly6c1	-0.87334	3.782974	24.00449	0.000165	0.005972
Ihh	-0.87293	4.666946	23.76843	0.000173	0.006128
Esco1	-0.87185	4.558793	47.15539	3.99E-06	0.000687
Ccdc88a	-0.87083	5.420083	48.9946	3.16E-06	0.000578
Emilin3	0.870403	3.477354	34.93288	2.28E-05	0.001945
Hp	-0.86998	2.21429	11.75015	0.003492	0.033759
Rps23-ps1	-0.86981	3.690631	25.15403	0.000131	0.005121
Srgn	-0.86768	2.576445	19.92759	0.0004	0.010028
Ro60	-0.86619	5.329857	43.28135	6.66E-06	0.000964
Tmem267	-0.8657	3.189949	15.69192	0.001139	0.017567
Ikzf5	-0.86535	2.515482	15.27438	0.001272	0.018769
Gm11992	-0.86407	3.382422	18.75869	0.000527	0.011576
Nr1h4	-0.86331	3.041918	22.68545	0.000217	0.006997
Gm17066	-0.8632	3.168134	19.82354	0.00041	0.010214
2210011C24Rik	-0.86156	2.69463	17.12049	0.000788	0.014381
Rpl38-ps2	-0.86132	5.862358	35.414	2.12E-05	0.001821
Rdh12	-0.86039	2.336863	14.7619	0.001462	0.020406
Rdh16	-0.85524	3.33848	9.620581	0.006918	0.049717
Lpl	-0.85204	5.8476	72.85984	2.55E-07	9.61E-05
Kif20b	-0.85119	5.338663	38.91534	1.24E-05	0.001367
Slc22a19	-0.85115	6.546165	28.16505	7.32E-05	0.003694
Uba7	-0.8494	4.601303	19.93232	0.0004	0.010028
Rpl21	-0.84897	5.853288	19.7303	0.000419	0.010335
Spin4	-0.84881	2.759115	14.15599	0.001728	0.022489
Nmi	-0.84543	3.805468	39.14029	1.2E-05	0.001362
Lrrcc1	-0.84401	5.513329	44.01869	6.02E-06	0.000898
Gm3325	-0.84368	2.909647	15.42525	0.001222	0.018216
Cmah	-0.8434	2.23673	10.74811	0.004779	0.039988
Slc26a4	-0.84321	2.539	11.11773	0.004251	0.037792
Cd46	-0.84312	2.651174	20.39699	0.00036	0.009502
Acsm5	-0.84199	3.582785	9.639745	0.006874	0.049538
Scg5	-0.84064	2.9785	16.27791	0.000977	0.016049
Mirg	0.839343	2.249703	13.39469	0.002144	0.02556
Zfp518a	-0.83851	5.066586	40.50489	9.82E-06	0.001235
Trim12a	-0.83543	3.624134	28.65541	6.68E-05	0.003623
Lca5l	-0.83389	2.168643	13.21385	0.002259	0.026361
Kcnq1ot1	-0.83339	4.954147	32.16277	3.6E-05	0.002496
Acox2	-0.83339	3.413401	11.65751	0.003593	0.034337
Robo3	-0.83308	2.104156	12.60284	0.002701	0.029341
Ctss	-0.83286	3.165187	20.84708	0.000325	0.008887
Rtn1	-0.83155	3.074294	16.56255	0.000908	0.015524
Gbgt1	0.830216	3.17018	31.62609	3.95E-05	0.002632
Plppr4	-0.83001	1.692497	11.04993	0.004342	0.038255

Ctse	-0.82643	3.711712	25.84507	0.000114	0.004731
Lix1	-0.82561	2.173901	12.77912	0.002564	0.028164
Rps15a-ps6	0.82422	2.996277	21.32614	0.000292	0.008307
Glrb	-0.82393	2.624534	12.93059	0.002453	0.027398
Gm7331	0.822631	2.110277	12.42814	0.002845	0.030202
Igtp	-0.82205	3.101821	18.53941	0.000555	0.011878
Cxcl14	-0.82177	5.961227	39.62474	1.12E-05	0.001336
Far2	-0.82143	2.69949	17.62557	0.000694	0.013496
Gm45792	-0.82069	2.387086	11.47679	0.003799	0.035415
Slco4c1	-0.81912	4.078811	29.11032	6.15E-05	0.003484
Cenpf	-0.81829	6.69268	55.29714	1.5E-06	0.000357
Napsa	-0.81448	7.829305	23.27102	0.000192	0.006495
Gm10736	-0.80974	4.95962	12.65835	0.002657	0.028952
Chac2	-0.80701	3.327705	26.49286	0.0001	0.004351
H2-K1	-0.80661	5.747226	17.9581	0.00064	0.012873
Rnf128	-0.80659	5.939863	64.67931	5.54E-07	0.000169
Tmcc1	0.803732	7.400175	90.07204	6.16E-08	4.2E-05
Smc4	-0.80183	7.031929	39.01268	1.22E-05	0.001367
Mybl1	-0.79737	3.100696	16.24473	0.000985	0.016128
Rtl10	0.795562	2.230926	13.60156	0.002021	0.024587
Lrrc19	-0.79552	3.086363	12.71189	0.002615	0.028603
Pde1a	-0.79504	4.252841	36.70994	1.73E-05	0.001571
Zfp758	-0.79462	3.00577	18.9193	0.000507	0.011364
Gm4459	-0.7945	2.771878	14.89995	0.001408	0.019964
Tchh	-0.79231	5.477534	60.37567	8.61E-07	0.000243
Gm5518	-0.792	6.326995	50.02102	2.79E-06	0.000539
Nutf2-ps1	0.790877	2.674819	19.50117	0.000442	0.010636
Rpl13a	-0.79084	8.945922	34.04205	2.64E-05	0.002119
Fbp2	0.790155	4.210344	36.7869	1.71E-05	0.001571
Spata1	-0.78984	2.33429	12.29933	0.002957	0.03069
Gm1821	0.78962	3.987755	28.44125	6.95E-05	0.003646
Cpxm2	-0.78954	3.851405	17.18265	0.000775	0.014275
Fah	-0.78829	5.323721	37.54575	1.52E-05	0.00154
Zbtb41	-0.78567	5.663663	55.05283	1.54E-06	0.000359
Ano5	-0.78515	2.805521	20.36805	0.000362	0.009536
Parp10	-0.78441	3.899626	18.15112	0.00061	0.012535
Rabgap1l	-0.78335	4.588302	45.98926	4.64E-06	0.000769
Malat1	-0.78279	9.225411	44.03006	6.02E-06	0.000898
Ppig	-0.78127	6.289783	50.77941	2.54E-06	0.000515
Zfp292	-0.78117	6.443165	38.37934	1.34E-05	0.001411
Zfp52	-0.77985	2.520574	14.54616	0.001551	0.021149
Gm8991	-0.77927	3.93015	23.34819	0.000189	0.00644
Slc22a27	-0.7766	3.184396	14.78281	0.001453	0.020325
Rpl9-ps6	-0.77619	6.358287	36.85743	1.69E-05	0.001571
Cenpe	-0.77512	6.216251	38.6302	1.29E-05	0.001397
Rab11b-ps2	0.774946	4.27403	38.20114	1.38E-05	0.001421
Aadat	-0.77394	7.28383	27.11653	8.91E-05	0.004123

1810026B05Rik	-0.77348	4.920156	28.44272	6.95E-05	0.003646
Mis18bp1	-0.77269	5.101237	27.49567	8.29E-05	0.003962
Btc	-0.77216	4.280477	38.42262	1.33E-05	0.001411
Rnf213	-0.7709	6.735745	74.56604	2.19E-07	9.23E-05
Aldh1a1	-0.76975	1.87221	10.39388	0.005358	0.042767
Vwa1	-0.76819	4.080729	28.66402	6.67E-05	0.003623
Cmpk2	-0.76753	4.557132	30.52602	4.78E-05	0.002952
Smc6	-0.76674	6.242978	51.96286	2.21E-06	0.000465
Agbl3	-0.76486	3.316156	24.21841	0.000158	0.005778
Gm44120	-0.76437	4.716864	48.56575	3.34E-06	0.000598
Arhgef38	-0.76338	4.697023	41.43489	8.61E-06	0.001156
Thbs2	0.761972	7.269348	58.66248	1.03E-06	0.000286
Gm5559	0.76123	2.216795	11.1832	0.004164	0.037354
Fmod	0.760957	4.250938	26.64571	9.75E-05	0.004277
Gm2000	-0.76091	4.570123	39.20915	1.19E-05	0.001361
Zfp273	-0.76013	2.511616	16.43866	0.000937	0.015692
Dpysl5	0.759795	3.728539	25.64272	0.000118	0.004874
Depdc1a	-0.75977	3.870432	14.62247	0.001519	0.02094
Bri3	0.759351	5.463197	33.41885	2.92E-05	0.002218
Zfp40	-0.7593	3.699514	21.5292	0.000279	0.008059
Hsd3b6	-0.75871	2.927157	10.68353	0.004879	0.04054
Apela	-0.75461	3.436828	16.9722	0.000818	0.014732
Gm26767	-0.75451	2.10722	10.854	0.004621	0.039376
Prrg1	-0.75433	3.783162	20.27997	0.000369	0.009594
Irf9	-0.75425	5.722527	32.66287	3.31E-05	0.002436
Svip	-0.75383	3.962603	26.55114	9.93E-05	0.004333
Pcmtd1	-0.75246	5.867635	41.96389	7.99E-06	0.001094
Slc51a	0.751526	2.496504	13.30539	0.0022	0.025957
Gsdmc2	0.751424	3.495177	17.90558	0.000648	0.01295
Cacnb4	-0.75056	3.092781	15.68564	0.00114	0.017567
Frmpd4	-0.74978	2.128379	10.2901	0.005542	0.043686
Tctex1d2	-0.7485	2.703912	14.45814	0.001589	0.021408
Gm20075	-0.74754	3.665114	24.23189	0.000157	0.005778
Snca	0.747062	3.069885	17.95351	0.00064	0.012873
Gm20008	-0.74659	3.461914	23.02133	0.000202	0.006704
Gprin3	-0.74516	2.573741	13.22255	0.002253	0.026312
Fam126b	-0.74397	4.537952	19.12909	0.000483	0.011039
Kcnc2	-0.74351	4.15882	32.52533	3.39E-05	0.002448
Ros1	-0.7413	4.317286	32.59022	3.35E-05	0.002436
Prss35	0.74108	2.643734	10.72588	0.004814	0.040145
Pgap1	-0.74094	5.35005	34.25066	2.55E-05	0.002092
Phlda2	-0.7403	2.045759	10.0451	0.006006	0.045684
Cyp2j11	-0.74026	3.942033	24.90567	0.000137	0.0053
9530082P21Rik	-0.73981	2.208493	12.83517	0.002522	0.027857
Phip	-0.73956	6.683289	31.02092	4.38E-05	0.002825
Acat3	-0.73954	2.950156	10.11943	0.00586	0.045154
P2ry2	-0.73755	2.161545	10.87581	0.004589	0.039217

Zfp945	-0.73502	5.201062	40.696	9.56E-06	0.001222
Ciart	0.734571	2.353591	12.45217	0.002825	0.03013
Got2-ps1	0.733847	1.692309	10.38013	0.005382	0.0429
Rps29	-0.73342	2.805977	17.87137	0.000653	0.013018
Ikzf2	-0.73206	3.569055	19.31622	0.000462	0.010856
Aunip	-0.7319	2.287102	12.31603	0.002942	0.030623
Angptl4	0.731376	3.306627	24.65112	0.000144	0.005472
Abcc2	0.73117	5.505992	44.38861	5.73E-06	0.000869
Rbm38	0.730648	4.73717	41.15218	8.96E-06	0.001173
Car5b	-0.72962	4.09097	21.61364	0.000274	0.007963
Ifi35	-0.72875	4.108376	12.83691	0.002521	0.027857
Gm49602	-0.72844	2.296241	12.67084	0.002647	0.02888
Gm12696	-0.72741	3.333466	19.28157	0.000465	0.010882
2610037D02Rik	-0.72735	2.745777	14.47358	0.001582	0.021364
Ifitm3	-0.72718	6.663426	18.63216	0.000543	0.011708
Shh	-0.72711	2.743229	11.4265	0.003859	0.035758
Cybb	-0.72706	2.174754	10.23132	0.005649	0.044268
Btbd8	-0.72593	2.955208	18.66206	0.000539	0.011679
Kazald1	-0.72467	3.233791	17.10659	0.00079	0.014416
Fsd1l	-0.72454	3.825883	15.19246	0.001301	0.019012
AI987944	-0.72295	2.963955	16.51117	0.00092	0.015579
Klf9	-0.72265	4.838395	36.77212	1.71E-05	0.001571
Mtln	-0.72158	3.891457	15.52245	0.001191	0.018002
Fbp1	-0.72149	8.06391	21.64151	0.000272	0.007941
Triqk	-0.72066	2.763766	11.0037	0.004406	0.038406
Emp3	0.72064	5.117618	47.41669	3.86E-06	0.000671
Igip	-0.72043	3.795897	27.87254	7.72E-05	0.003804
Ccdc160	-0.71844	2.851425	16.72272	0.000871	0.015226
Slit3	0.718267	5.144456	35.65945	2.04E-05	0.001763
Chmp1b	-0.71723	4.877963	32.09502	3.64E-05	0.002512
4632427E13Rik	-0.71712	2.228095	10.17163	0.005761	0.044776
Cacng4	0.717067	3.448656	17.23337	0.000766	0.014138
Mfap3l	-0.71692	4.907937	30.16334	5.09E-05	0.003103
AC132253.9	-0.71675	2.214002	12.04134	0.003196	0.032155
Rcor2	0.716598	4.911615	52.86945	1.98E-06	0.000439
Lrrn3	-0.71642	2.19802	11.85397	0.003383	0.033248
Sec62	-0.71604	7.084734	63.46803	6.25E-07	0.000185
Brcc3	-0.71438	4.715685	26.8819	9.31E-05	0.004189
Ccdc39	-0.71399	3.371588	11.69677	0.00355	0.03407
Lztfl1	-0.71383	4.981964	43.25036	6.69E-06	0.000964
Galnt3	-0.71374	4.838655	43.38431	6.57E-06	0.000962
Slf1	-0.71307	4.69227	40.68279	9.58E-06	0.001222
Pura	-0.71279	4.763723	30.88167	4.49E-05	0.002874
Rb1cc1	-0.71164	5.778839	41.11851	9E-06	0.001173
Bche	-0.71136	2.45318	10.41009	0.005329	0.042694
Gm17300	0.710957	2.73129	12.968	0.002426	0.027244
Snap91	-0.71041	5.575018	33.44976	2.91E-05	0.002218

Sclt1	-0.71012	4.721856	31.08365	4.33E-05	0.002805
Bace2	-0.71	2.494832	11.66144	0.003589	0.034336
Nexn	-0.70902	3.886498	23.48387	0.000184	0.006357
Scai	-0.70681	5.737092	24.87138	0.000138	0.005325
Slfn5	-0.70619	4.907005	34.50679	2.45E-05	0.002026
Casp8ap2	-0.70572	5.155543	23.03351	0.000202	0.0067
Zbtb20	-0.7054	6.025167	38.32289	1.35E-05	0.001411
Nbeal1	-0.70519	5.556713	26.80361	9.46E-05	0.004201
Arg2	-0.70434	4.060266	22.63031	0.00022	0.006997
Abcd2	-0.70432	2.663105	10.93714	0.0045	0.038843
Napb	-0.70357	2.545437	13.7939	0.001913	0.023769
Atrx	-0.70264	7.867552	30.35524	4.92E-05	0.00301
Umod	-0.70229	7.224637	9.767469	0.006586	0.048287
Rnf186	-0.70195	3.246613	16.52634	0.000916	0.015579
CAAA01118383.1	-0.70154	5.186637	28.47275	6.91E-05	0.003646
Tspan12	-0.70133	5.499931	37.4293	1.55E-05	0.001554
Slc5a3	-0.70097	6.196041	49.99779	2.79E-06	0.000539
Tagln	0.700589	6.029659	50.97405	2.48E-06	0.000512
2610044O15Rik8	-0.69976	4.194788	26.86564	9.34E-05	0.004189
Fam199x	-0.69741	4.890146	25.25976	0.000128	0.005048
Slc6a4	0.696561	2.10974	11.46396	0.003815	0.035538
Zfp994	-0.69598	3.046982	13.47091	0.002097	0.025194
Astn2	0.695897	2.866994	17.38103	0.000738	0.013886
2002-09-01	-0.69518	3.279411	11.78951	0.00345	0.033593
Nox4	-0.69506	3.88882	22.96545	0.000205	0.006772
Etv1	-0.69498	3.458831	22.27146	0.000237	0.007374
Gpr135	-0.69475	2.690064	13.4635	0.002102	0.02523
Dlgap1	-0.69409	2.227443	10.52065	0.005142	0.041947
Gatm	-0.69406	2.744897	11.03799	0.004359	0.038255
Ugt8a	-0.69267	4.248675	26.10777	0.000108	0.004599
Uprt	-0.68905	2.943518	13.90635	0.001853	0.023418
Atp6v1c2	-0.68882	3.601528	24.33679	0.000154	0.005735
Tnni1	0.687574	2.193322	11.62605	0.003628	0.034508
Atp11c	-0.6866	4.908235	25.85405	0.000114	0.004731
Ift74	-0.68632	5.005332	39.58373	1.12E-05	0.001336
Chml	-0.68624	4.301347	23.98784	0.000165	0.005972
Zfp942	-0.68588	4.210497	24.00877	0.000165	0.005972
Tmx3	-0.68434	6.269341	21.98418	0.000253	0.007644
Dek	-0.68391	7.1724	33.92105	2.69E-05	0.002119
Slfn9	-0.68356	4.882959	32.51099	3.4E-05	0.002448
Hmga1	0.682597	6.665747	38.74873	1.27E-05	0.001389
Ogfrl1	-0.68235	5.061051	31.83816	3.81E-05	0.002579
Kcnt2	-0.68185	3.220701	12.7714	0.00257	0.02821
Rasd2	0.681772	2.897915	10.7529	0.004772	0.039946
Angpt1	-0.68147	4.903287	34.9079	2.29E-05	0.001945
Snhg11	-0.68095	3.653272	18.25929	0.000594	0.01241
Igdcc4	0.680523	5.521774	42.74359	7.17E-06	0.001001

Ptger3	-0.68043	3.871623	9.820754	0.00647	0.047851
Matr3	-0.68028	7.436729	36.96014	1.66E-05	0.001571
Gm43980	-0.67849	4.253309	11.82358	0.003415	0.03341
Mpp5	-0.6782	5.948675	37.29436	1.58E-05	0.001556
Adgrb2	0.678126	4.522565	24.47379	0.00015	0.005624
H2-D1	-0.67792	6.354297	17.86711	0.000654	0.013018
Ppp1r3c	0.677742	5.968372	22.23562	0.000239	0.007403
Gm38394	-0.6774	3.358488	10.56038	0.005077	0.041652
Hgd	-0.67728	5.14363	18.22577	0.000599	0.012482
Zfp944	-0.67574	3.759043	23.12278	0.000198	0.006617
Trmt112-ps2	0.674008	4.167871	24.25966	0.000156	0.005778
Mier1	-0.67399	6.278387	36.74448	1.72E-05	0.001571
Zfp72	-0.67319	2.767245	12.13915	0.003103	0.031629
Ppm1k	-0.67279	4.209091	24.36376	0.000153	0.005715
Tbca	-0.67276	5.936007	39.27212	1.18E-05	0.001361
Tmem30b	-0.67202	4.693725	32.07736	3.66E-05	0.002512
Cndp1	0.671954	3.807383	21.82563	0.000262	0.00778
Gm32618	-0.67142	2.828433	15.85972	0.001089	0.017096
Xrcc4	-0.67114	3.491863	17.20614	0.000771	0.014206
Car9	0.66927	2.234483	10.51775	0.005147	0.041947
Crebrf	-0.66899	5.069343	39.85864	1.08E-05	0.001319
Dzip3	-0.66816	5.80686	24.24931	0.000157	0.005778
Slc16a3	0.667918	5.659018	27.06065	9E-05	0.004129
Gm10177	0.667638	4.519385	31.624	3.95E-05	0.002632
Myrf	0.665549	4.319136	22.71809	0.000216	0.006993
Col4a4	-0.66516	6.160746	57.1091	1.22E-06	0.000319
Parp12	-0.66456	5.233908	27.22784	8.72E-05	0.00408
Cfap69	-0.66448	3.276919	17.57807	0.000702	0.013587
Map9	-0.66226	3.938341	16.91988	0.000829	0.014862
Lrriq1	-0.66143	4.949418	32.22173	3.57E-05	0.002496
Zbtb6	-0.66118	4.593745	18.8624	0.000514	0.011449
Mfap2	0.659619	7.083605	43.15792	6.77E-06	0.000968
Mob3a	0.659372	3.612049	25.20958	0.000129	0.005076
Gm12918	-0.65823	4.935159	28.58376	6.77E-05	0.003628
Lipo3	-0.6579	4.019309	18.90419	0.000509	0.011364
Fibcd1	0.65731	2.785899	11.21118	0.004128	0.037164
Fndc1	0.65668	2.636207	9.928561	0.006242	0.046653
Lin7c	-0.65581	6.716777	36.42933	1.8E-05	0.001616
Tceal1	-0.65529	3.397582	13.87852	0.001868	0.023418
Chmp4c	-0.65421	3.721435	20.43977	0.000356	0.009453
Rpl24	-0.6539	5.860205	31.72642	3.88E-05	0.002616
Cep126	-0.65271	3.954338	21.83754	0.000261	0.007776
Pex12	0.652324	2.937603	16.21759	0.000992	0.016152
Mtm1	-0.65229	2.991035	16.03951	0.001039	0.016616
Zmat1	-0.65132	3.254766	12.9577	0.002433	0.027268
Slc26a7	-0.6512	3.678994	11.69652	0.00355	0.03407
Nfe2l3	-0.65084	2.603419	9.886307	0.00633	0.047113

Inpp4b	-0.65079	3.958195	16.97937	0.000816	0.01472
Adamts14	0.650728	3.309835	18.77839	0.000524	0.011551
Tspan8	-0.65037	5.340895	20.12004	0.000383	0.00975
Hltf	-0.64888	5.027188	28.99603	6.28E-05	0.003534
Ttc14	-0.64863	6.634727	18.93038	0.000506	0.011351
Haus3	-0.64827	4.065181	15.6872	0.00114	0.017567
Rp2	-0.64744	4.712133	17.7549	0.000672	0.013203
Zxdb	-0.6471	2.924721	11.38616	0.003908	0.036011
Efnb3	0.647022	4.847797	25.69491	0.000117	0.004843
Ndufb1-ps	-0.64599	4.56598	13.51163	0.002073	0.025003
Gm20300	-0.64543	4.00277	16.63206	0.000892	0.015332
Smc2	-0.64514	6.439168	26.93442	9.22E-05	0.004189
Gm17709	0.644545	2.705925	14.84482	0.001429	0.02014
Stk26	-0.64429	3.91827	22.66373	0.000218	0.006997
Zfp850	-0.64411	2.965922	11.56457	0.003698	0.034926
Gm10093	0.641043	4.327788	15.08622	0.001339	0.01931
Rock1	-0.64027	5.929043	38.95237	1.23E-05	0.001367
Dynlt1-ps1	0.639693	5.476557	28.48827	6.89E-05	0.003646
Slco1a6	-0.63933	4.075179	17.97143	0.000638	0.012873
Zfp950	-0.63888	5.563473	25.41386	0.000124	0.00499
Hist3h2a	-0.63878	3.491137	14.10115	0.001755	0.022741
Eea1	-0.63763	5.670273	21.48048	0.000282	0.008129
Usp53	-0.63762	4.458168	27.58457	8.15E-05	0.003917
Kcnh7	-0.63623	3.205661	12.2555	0.002996	0.030835
Them6	-0.63544	2.828419	12.37323	0.002892	0.030302
Myh14	-0.63514	3.498652	17.15027	0.000782	0.014317
Vbp1	-0.63471	5.71119	28.90639	6.38E-05	0.003555
Cd59a	-0.63329	4.575429	22.69137	0.000217	0.006997
Zbtb12	0.633165	6.183844	46.86287	4.14E-06	0.000705
Bcap29	-0.63282	3.83994	20.24474	0.000372	0.009594
Ipcef1	-0.63187	3.675574	20.83069	0.000326	0.008906
Hmgn5	-0.63155	4.557786	30.98748	4.41E-05	0.002831
Tbc1d8b	-0.63134	4.297474	16.66218	0.000885	0.015289
Cwf19l2	-0.6311	4.557622	22.21594	0.00024	0.007422
Osr1	0.630779	3.949981	18.22124	0.0006	0.012482
Armc6	0.630771	4.402058	28.73797	6.58E-05	0.003591
Cela1	0.630685	3.991948	22.77412	0.000213	0.00694
Slc16a4	-0.63057	4.824931	16.03559	0.00104	0.016616
Ccdc112	-0.63038	3.865159	12.23329	0.003016	0.030888
Gja5	0.630199	5.311848	24.79536	0.00014	0.005365
Yes1	-0.62894	5.6811	38.36596	1.34E-05	0.001411
Slc16a7	-0.62883	5.351539	21.22713	0.000298	0.008451
Tmed5	-0.62869	4.878896	17.84271	0.000658	0.013064
Corin	-0.62868	6.019215	32.6174	3.34E-05	0.002436
E030013I19Rik	-0.62837	2.676558	10.85305	0.004622	0.039376
Spopl	-0.62823	5.080106	28.28489	7.15E-05	0.003677
Gm11703	-0.62808	3.36817	10.49374	0.005187	0.042047

Ptpn4	-0.62759	5.496543	21.41847	0.000286	0.008192
Ryr1	0.62736	2.824107	12.25253	0.002998	0.030835
Cdc42ep2	0.626499	4.824355	39.6681	1.11E-05	0.001336
Cmc1	-0.62633	3.627698	18.6603	0.000539	0.011679
Socs4	-0.62623	5.664022	22.81396	0.000211	0.006902
Dynlt3	-0.62606	5.89563	39.18447	1.19E-05	0.001361
Serpina10	-0.62564	4.118208	14.87541	0.001417	0.020021
Arid4b	-0.62546	5.987809	31.83212	3.81E-05	0.002579
Gcnt1	-0.62535	6.788476	28.81026	6.5E-05	0.003577
Gm19950	-0.62438	4.931094	23.15434	0.000197	0.006594
Efna3	0.62351	2.92911	13.68191	0.001975	0.02418
Trim34a	-0.62341	3.030389	12.57243	0.002725	0.029426
Sult1d1	-0.6234	6.478968	23.43112	0.000186	0.006383
Ndrg1	0.622775	8.662221	26.90558	9.27E-05	0.004189
Kng2	-0.62259	6.332641	20.62575	0.000341	0.009228
Gm9385	-0.62248	3.226333	11.99569	0.00324	0.032378
Dnaaf2	-0.62194	2.829483	11.34327	0.00396	0.03625
St8sia2	0.621597	4.798209	28.14135	7.35E-05	0.003694
Osbpl8	-0.61968	5.631353	19.36633	0.000456	0.010801
Kif21b	0.619357	4.331599	23.82687	0.000171	0.006099
Exph5	-0.61838	3.12197	13.27858	0.002217	0.026078
Galnt16	0.616883	4.534486	30.37961	4.9E-05	0.003008
Aoc3	0.616649	4.737521	33.99511	2.66E-05	0.002119
C1qa	-0.61589	3.629613	13.9108	0.001851	0.023413
Zfp760	-0.61583	4.751664	26.85888	9.36E-05	0.004189
B2m	-0.61513	6.822942	33.60754	2.83E-05	0.002208
Mmp17	0.613989	4.30589	20.34174	0.000364	0.009565
Gcnt4	-0.61338	3.072228	13.13126	0.002313	0.026631
Mrps36	-0.61296	3.985276	22.23995	0.000239	0.007403
Arid4a	-0.61285	5.238724	33.33261	2.96E-05	0.002218
Cenpk	-0.61221	3.518087	10.74146	0.00479	0.040036
Thoc2	-0.61205	6.62461	25.52385	0.000121	0.004944
Sfn	-0.61143	3.98677	16.08256	0.001028	0.016477
Acsl4	-0.61127	5.718931	34.53595	2.43E-05	0.002026
Ghr	-0.61075	6.67001	44.39724	5.73E-06	0.000869
Sgo2a	-0.61006	3.808155	16.37693	0.000952	0.015835
Pdxk	0.609761	5.550854	27.82381	7.8E-05	0.003818
Zup1	-0.60941	4.126938	13.25787	0.00223	0.026182
Gca	-0.60875	4.732341	16.3001	0.000971	0.016031
Vps13a	-0.60839	5.118891	28.96272	6.32E-05	0.003534
Rnf138	-0.60833	5.097013	30.72197	4.62E-05	0.002902
Dipk1a	-0.60544	3.502228	16.85572	0.000842	0.014976
Mogat2	0.605406	4.024861	11.42372	0.003863	0.035758
Vnn1	-0.60487	3.020962	9.664865	0.006816	0.049262
Fam204a	-0.60359	4.795725	22.15477	0.000243	0.007456
Klhl28	-0.60324	4.383593	17.51382	0.000714	0.01365
Fam217b	0.602962	3.522629	14.90888	0.001404	0.019935

Micu3	-0.60296	3.964359	15.82637	0.001099	0.017165
Eif1	0.602761	7.765081	39.06341	1.21E-05	0.001367
Mif	0.602701	8.389812	37.0919	1.63E-05	0.001571
Zfp933	-0.60244	3.74297	18.0312	0.000628	0.012757
Rsrc2	-0.60121	6.885195	33.37021	2.94E-05	0.002218
Lpar2	0.601096	4.726976	28.19774	7.27E-05	0.003694
Sh3bgrl	-0.60081	6.904943	37.33948	1.57E-05	0.001554
Ubald2	0.600212	5.540994	25.33908	0.000126	0.005002
Nsrp1	-0.60004	4.810145	28.10734	7.39E-05	0.003694
Nudt12	-0.60004	3.549337	11.04348	0.004351	0.038255
Rif1	-0.59973	6.208499	22.19093	0.000242	0.007423
Ranbp2	-0.59941	7.201322	28.12766	7.37E-05	0.003694
Srpx2	0.598975	3.140424	15.50343	0.001197	0.018076
Cav2	-0.59835	5.176518	27.2921	8.62E-05	0.004052
Zfp943	-0.59815	4.064679	18.01286	0.000631	0.012773
Cep85	-0.59797	5.935475	13.22557	0.002251	0.026312
Gtpbp10	-0.59784	4.813404	18.57109	0.000551	0.011816
Gm50010	-0.59727	3.008667	10.50248	0.005172	0.042012
Mki67	-0.59717	8.343656	20.90154	0.000321	0.008848
Gja4	0.596677	4.497264	22.48138	0.000227	0.007096
Lrp5	0.596355	7.210308	36.79966	1.7E-05	0.001571
Zfp638	-0.59552	6.670749	32.45002	3.43E-05	0.00245
Smc5	-0.59469	6.052552	25.37398	0.000125	0.00499
Cfap36	-0.59417	5.045589	29.73862	5.49E-05	0.003274
Col1a1	0.594122	10.49943	27.12916	8.89E-05	0.004123
Hhip	-0.59393	2.981874	13.87144	0.001872	0.023418
Thrb	-0.59373	2.70567	9.709882	0.006714	0.048843
Rora	-0.59353	4.088182	12.29333	0.002962	0.030709
2005-09-01	0.593328	3.546759	17.50282	0.000716	0.013659
Prox1	-0.5931	2.903384	11.37781	0.003918	0.036024
Zfp654	-0.59301	4.963544	23.6222	0.000178	0.00625
Far1	-0.59206	7.135947	28.58186	6.77E-05	0.003628
Lpcat2	-0.59201	2.681014	10.00182	0.006092	0.04597
Cox7c	-0.59196	5.429567	27.60792	8.12E-05	0.003911
Msi2	-0.5917	7.391004	25.00892	0.000134	0.005223
Lactb2	-0.58993	6.666714	21.74664	0.000266	0.007854
Styx	-0.58964	4.277215	11.67132	0.003578	0.034269
Gkap1	-0.58898	4.562061	28.56603	6.79E-05	0.003628
Gramd1c	-0.58845	3.362349	11.32108	0.003988	0.036426
Mzt1	-0.58797	6.085728	28.73759	6.58E-05	0.003591
Nckap5	-0.58666	2.954352	10.88644	0.004573	0.039177
Kif5b	-0.5866	7.318123	29.50771	5.73E-05	0.003325
Tkfc	0.586438	6.795712	44.87892	5.37E-06	0.000852
Bclaf3	-0.58562	4.199653	20.48778	0.000352	0.009406
Tsga10	-0.58479	3.460706	10.76088	0.00476	0.039936
Ptbp3	-0.58465	7.7505	27.44704	8.37E-05	0.003984
Mme	-0.5844	7.766978	30.53888	4.77E-05	0.002952

Serpinf2	0.584199	6.134204	16.69758	0.000877	0.015235
Acta2	0.584075	8.775747	44.58569	5.58E-06	0.000869
Acadsb	-0.58316	6.43539	28.41048	6.99E-05	0.00365
Tcf24	-0.58316	3.573798	18.22853	0.000599	0.012482
Gamt	0.583053	3.62678	14.14206	0.001735	0.022548
Dmxl1	-0.58233	6.092932	25.49745	0.000122	0.004955
Wbp4	-0.58157	5.606336	35.68245	2.03E-05	0.001763
Slc8a1	-0.58115	7.138648	35.82251	1.98E-05	0.001741
Aldh1a3	-0.58077	6.453283	36.44367	1.8E-05	0.001616
Chrna4	0.580583	3.923318	20.58282	0.000345	0.009289
Sh3pxd2b	0.580426	6.844391	29.6318	5.6E-05	0.003298
Akap9	-0.58017	6.760153	30.73389	4.61E-05	0.002902
Dnajc21	-0.58016	4.967162	28.59582	6.76E-05	0.003628
Oxgr1	-0.57924	3.836704	14.25088	0.001683	0.022249
Aimp2	0.579131	5.122246	21.36754	0.000289	0.008269
Slc7a5	0.578607	5.215986	13.49144	0.002085	0.025114
Cltrn	-0.57838	7.172723	23.7678	0.000173	0.006128
Olfr1033	-0.57803	3.401645	10.8358	0.004648	0.039476
Zfp606	-0.57699	5.128834	26.42609	0.000102	0.004397
Cox17	-0.57647	4.957897	27.42356	8.4E-05	0.003984
Chm	-0.57645	5.042432	23.7103	0.000175	0.006161
Crabp2	0.575878	3.562007	16.51249	0.000919	0.015579
Ndufa1	-0.57566	4.841718	19.63963	0.000428	0.010396
Cyp2j5	-0.57564	6.870712	14.87923	0.001416	0.020017
Tm2d1	-0.57458	4.32508	21.60034	0.000275	0.007973
Pibf1	-0.57399	3.707034	18.05399	0.000625	0.012747
Ube3a	-0.57334	6.296424	18.74137	0.000529	0.011596
Rida	-0.5732	6.929592	22.63049	0.00022	0.006997
Zfp322a	-0.57316	5.606291	23.15327	0.000197	0.006594
Pcbd2	-0.57305	3.579257	12.30835	0.002949	0.030661
Ccdc186	-0.57269	5.784865	17.17257	0.000777	0.014282
Gm3756	0.572091	4.472577	16.50218	0.000922	0.015579
Cr2	-0.57096	2.814005	10.19728	0.005713	0.044557
Rps3a3	0.570876	5.237812	27.04108	9.04E-05	0.004129
Igf2bp3	0.570676	4.77303	26.67098	9.7E-05	0.004267
Id4	-0.5703	5.935217	29.50777	5.73E-05	0.003325
Hist1h1c	-0.57021	5.016464	13.06089	0.002361	0.026766
Dnaaf3	-0.57013	3.037045	12.05997	0.003178	0.032046
Rpe	-0.57006	5.150554	24.79356	0.00014	0.005365
Exoc5	-0.56962	5.815783	21.93034	0.000256	0.007695
Kitl	-0.56913	5.771943	26.86255	9.35E-05	0.004189
Larp4	-0.56893	6.425492	27.68091	8.01E-05	0.003871
Lysmd3	-0.56876	4.713913	15.87234	0.001086	0.017076
Ptbp2	-0.56844	6.26085	21.01785	0.000313	0.008688
Ccdc34	-0.56794	5.384398	22.72864	0.000215	0.00699
Kansl1l	-0.56681	5.599033	25.52407	0.000121	0.004944
Nup188	0.566623	6.824298	26.73374	9.58E-05	0.004242

Tmem178	-0.56606	4.939228	20.88452	0.000322	0.008856
Stk3	-0.56604	4.896641	25.95087	0.000112	0.004675
Cacna2d2	0.566007	4.062967	21.93037	0.000256	0.007695
Fibin	-0.56579	4.934667	27.67606	8.01E-05	0.003871
Aoc1	-0.56578	4.202573	16.02392	0.001043	0.016651
Prpf40a	-0.56571	6.754215	18.15802	0.000609	0.012535
Fbln5	0.565591	7.429223	46.80376	4.17E-06	0.000705
Kif5c	-0.56545	4.164916	15.94213	0.001066	0.016875
Ier3	-0.56461	4.770546	14.60267	0.001527	0.020966
Dtx3l	-0.56393	3.858948	15.60724	0.001164	0.017751
Sphk1	-0.5638	3.042013	10.0472	0.006002	0.045684
Knl1	-0.56366	4.918384	11.43702	0.003847	0.035686
Erbin	-0.5636	7.552283	29.41026	5.83E-05	0.003344
Lum	0.563253	4.705407	15.99985	0.00105	0.016703
Zfp800	-0.56321	3.834982	9.815765	0.00648	0.047889
Prxl2c	-0.56236	5.430359	29.57418	5.66E-05	0.003321
Gtf2a1	-0.56219	5.764842	23.76526	0.000173	0.006128
Fcho2	-0.56182	6.538907	22.92348	0.000207	0.006789
Tmem106b	-0.56169	6.924883	36.26417	1.85E-05	0.001633
Cd248	0.561593	6.195391	34.73612	2.36E-05	0.001981
Ptprv	0.56068	3.224384	10.79004	0.004716	0.039756
Vps37a	-0.55961	5.516089	26.30627	0.000104	0.004478
4930412O13Rik	-0.55912	2.801777	10.22722	0.005657	0.044298
Mysm1	-0.55909	5.772883	22.56469	0.000223	0.007056
Srm	0.558471	6.127845	30.09929	5.15E-05	0.003128
Gpbp1	-0.55844	6.329169	24.25789	0.000156	0.005778
Zfp81	-0.5582	4.813777	25.90039	0.000113	0.00471
Pcmtd2	-0.55762	6.328284	30.76649	4.58E-05	0.002901
Fnip1	-0.55761	5.184225	16.70429	0.000875	0.015235
Tspan18	0.55752	6.848959	32.43459	3.44E-05	0.00245
Hint3	-0.55751	3.376514	13.06068	0.002361	0.026766
Cep83	-0.55751	4.894293	14.24251	0.001687	0.022261
Cenpc1	-0.55718	5.140966	14.12458	0.001743	0.022625
Parvb	0.557128	5.065367	26.3931	0.000102	0.004414
Gm15542	-0.55702	3.781165	16.3146	0.000968	0.015986
Dpy19l4	-0.55696	5.557429	29.29636	5.95E-05	0.00339
Creb3l1	0.55695	5.542017	36.89254	1.68E-05	0.001571
Zfp763	-0.55687	4.147895	16.00106	0.00105	0.016703
Gpr155	-0.55668	3.647526	10.69743	0.004858	0.040395
Cryab	-0.55649	5.08132	14.36302	0.001631	0.02172
Peg10	0.556354	5.570406	19.13053	0.000482	0.011039
Napepld	-0.55524	4.057619	15.60833	0.001164	0.017751
Cwc27	-0.55511	4.498477	25.33965	0.000126	0.005002
Procr	0.554984	3.257624	9.776227	0.006566	0.048231
Foxq1	-0.55497	4.728229	10.64894	0.004934	0.040818
Tmem47	-0.55469	4.986682	16.7648	0.000862	0.015159
Gulp1	-0.55388	4.648714	12.57526	0.002723	0.029419

1110059G10Rik	-0.55353	3.687503	14.79684	0.001448	0.020277
Mrps18c	-0.55256	4.169619	21.79802	0.000263	0.007801
Rps2-ps13	0.55254	6.214548	22.53866	0.000224	0.007062
Zfp229	-0.55169	3.86703	13.87242	0.001871	0.023418
2007-03-01	-0.55124	6.580716	20.52624	0.000349	0.009367
Ccdc66	-0.55114	4.107938	12.53146	0.002759	0.029606
Rpl19	0.551135	8.647589	23.73606	0.000174	0.006153
Ptar1	-0.55099	6.196911	15.7236	0.001129	0.017483
Nol8	-0.55093	4.556091	18.8365	0.000517	0.011455
Ankrd12	-0.55031	4.81886	15.16212	0.001311	0.019091
Tacstd2	-0.54963	5.902344	28.12532	7.37E-05	0.003694
5730455P16Rik	-0.5495	4.74582	16.33174	0.000963	0.01593
Nxt2	-0.54949	4.360691	16.85648	0.000842	0.014976
Tigd2	-0.54918	3.848726	17.94383	0.000642	0.012873
R3hdml	0.548962	4.685031	18.9063	0.000509	0.011364
Suco	-0.54856	6.004716	28.78641	6.52E-05	0.003581
Gm9770	0.548276	4.084503	16.92098	0.000828	0.014862
Mgat4a	-0.5479	5.356436	33.10758	3.07E-05	0.002291
Ppp4r3b	-0.5479	6.371137	31.48981	4.04E-05	0.002673
Mt1	0.547857	6.017709	19.78302	0.000414	0.010296
Lingo1	0.547835	3.505079	13.69239	0.001969	0.024149
Notch3	0.54763	7.299752	31.5923	3.97E-05	0.002637
Kif18a	-0.54736	3.919094	12.56778	0.002729	0.029449
Zc3hav1	-0.54702	4.382766	15.99916	0.00105	0.016703
Pde4a	0.546885	4.545366	23.6641	0.000177	0.006208
Gm11361	-0.54688	4.038969	14.03738	0.001786	0.022889
Cd2ap	-0.54638	7.321279	25.06264	0.000133	0.005181
Vma21	-0.54615	5.25314	20.27022	0.00037	0.009594
Hcn4	0.545997	3.229483	13.94563	0.001833	0.023295
2610008E11Rik	-0.54548	5.535258	26.11497	0.000108	0.004599
Ythdc2	-0.54518	4.197349	16.46919	0.00093	0.015638
Mocs1	0.544422	4.95529	29.5348	5.7E-05	0.003325
Odf2l	-0.54394	4.861272	18.70013	0.000534	0.011639
Elmod2	-0.54335	4.902364	23.4924	0.000183	0.006357
Zfx	-0.54314	5.961751	21.20721	0.0003	0.008462
Fn1	0.542771	9.966992	23.38248	0.000188	0.006406
Nhs	-0.54269	4.707464	22.27025	0.000237	0.007374
Gm12184	0.542229	3.740697	18.34689	0.000582	0.012207
U2surp	-0.54203	6.78408	18.83405	0.000518	0.011455
Zfp62	-0.54186	5.791993	28.89502	6.4E-05	0.003555
Srsf10	-0.54145	7.309177	20.55858	0.000347	0.009312
Filip1l	-0.54136	5.603236	29.19882	6.05E-05	0.00344
Sez6l2	0.541182	2.816805	9.631546	0.006893	0.049605
Gm20559	-0.5404	4.169768	13.40329	0.002139	0.025514
Gk	-0.54027	6.561674	17.89964	0.000649	0.012955
Rin3	0.540029	4.981698	22.48416	0.000227	0.007096
1810022K09Rik	-0.5399	4.02495	10.76277	0.004757	0.039936

St8sia6	-0.53986	3.835644	14.99055	0.001374	0.019687
Bmpr1b	-0.53985	4.664865	25.28592	0.000127	0.005033
Pycr1	0.539377	3.851159	16.28206	0.000976	0.016047
Rbm34	-0.53887	4.772709	18.59214	0.000548	0.011771
Tank	-0.53877	4.111238	11.98725	0.003249	0.032411
Cox6c	-0.5386	7.334628	28.0431	7.48E-05	0.003716
Col4a3	-0.53842	5.346932	11.48717	0.003787	0.035359
Col8a2	0.538376	5.325626	18.97371	0.000501	0.011263
Des	0.538334	5.211256	18.97558	0.0005	0.011263
Chic1	-0.53806	4.050238	12.61914	0.002688	0.029235
Tuba1c	0.537438	7.05383	33.39096	2.93E-05	0.002218
Rerg	-0.53647	4.315382	16.52642	0.000916	0.015579
Ubxn2a	-0.53641	4.420193	19.04367	0.000492	0.011163
Ambp	0.536297	3.196556	9.970902	0.006155	0.04624
Mastl	-0.5357	4.010923	13.38682	0.002149	0.025566
Trp53inp1	-0.53562	5.222706	18.20525	0.000602	0.012513
Zfp738	-0.5356	4.699256	13.14959	0.002301	0.026594
Dcun1d1	-0.53533	5.301198	21.96536	0.000254	0.007663
Prpf39	-0.53503	6.417174	20.47855	0.000353	0.00941
Hic2	0.534828	3.45361	15.94928	0.001064	0.016874
Brwd3	-0.53451	4.791351	11.09871	0.004276	0.037925
Zfp120	-0.53417	3.580516	9.895676	0.00631	0.04703
Bloc1s1	0.533924	4.872996	18.48875	0.000562	0.011971
Polr2l	0.53346	5.109434	21.58128	0.000276	0.007994
Mllt3	-0.53289	5.188982	25.58816	0.00012	0.004904
Resf1	-0.53258	5.460144	19.01452	0.000496	0.011183
Itih5	-0.53255	4.507011	21.74422	0.000266	0.007854
Ppl	-0.53229	4.161991	16.64556	0.000889	0.015317
Ap1s2	-0.53151	4.448131	19.09809	0.000486	0.011062
Alpl	-0.53126	5.970732	20.86543	0.000323	0.008864
Ildr2	-0.53113	4.820628	18.62962	0.000543	0.011708
Phgdh	0.530493	8.074772	30.58536	4.73E-05	0.002952
Nell1	0.530105	3.769046	14.86837	0.00142	0.02004
Snrpc	0.530067	6.158846	33.94941	2.68E-05	0.002119
Nr1d2	-0.52985	5.010963	19.64434	0.000428	0.010396
Stra6	0.529828	4.640422	14.08228	0.001764	0.022795
Gm5884	0.529502	4.11075	19.43493	0.000449	0.010715
Zfp729a	-0.5293	4.180811	15.57841	0.001173	0.017856
Smarca1	-0.529	5.338924	21.18131	0.000301	0.008497
Top2b	-0.52895	7.625268	27.09098	8.95E-05	0.004129
Veph1	-0.52878	5.643118	27.27526	8.64E-05	0.004054
Cpm	-0.52839	5.892449	31.24494	4.22E-05	0.002748
Zbtb44	-0.52791	5.547967	27.18347	8.79E-05	0.004103
Rps12-ps3	0.527834	8.664753	19.18226	0.000477	0.011014
Manea	-0.52776	5.228056	17.26711	0.000759	0.014107
Tbxa2r	0.52753	3.758141	14.0831	0.001764	0.022795
Slc35c1	0.527312	4.737457	22.90959	0.000207	0.006789

Rnpc3	-0.52686	4.957044	15.43763	0.001218	0.018201
Trip11	-0.52638	5.847951	25.4248	0.000124	0.00499
mt-Tp	-0.52633	5.164888	11.5757	0.003685	0.03488
Rrm2b	-0.52583	4.116107	16.2007	0.000997	0.016177
Dcn	0.525263	6.874148	32.47449	3.42E-05	0.00245
Rnf152	-0.5252	4.192045	14.97463	0.00138	0.019693
Ogdhl	0.525087	4.322876	19.25045	0.000469	0.01091
Zfp946	-0.52494	4.046036	9.970662	0.006155	0.04624
Lmx1b	0.524571	4.3994	22.50492	0.000226	0.007094
Krt23	-0.52436	3.812856	10.32643	0.005476	0.043324
Abcb7	-0.52416	5.264621	17.48621	0.000719	0.013671
1700020I14Rik	-0.5241	7.616742	17.78944	0.000667	0.013162
Rassf6	-0.52402	4.673631	13.33291	0.002182	0.025862
Rnf165	0.523864	3.109454	9.938714	0.006221	0.046535
Riox1	0.523851	4.216092	16.51635	0.000918	0.015579
Pnrc2	-0.52341	7.008099	28.96368	6.32E-05	0.003534
Tbc1d32	-0.52341	4.211121	15.46698	0.001209	0.018113
Acbd5	-0.52319	5.515454	21.56306	0.000277	0.008013
Cdh3	0.523188	6.269626	20.42283	0.000358	0.009474
Col14a1	0.52318	7.446083	37.34483	1.57E-05	0.001554
Serpina3g	-0.52307	3.699674	10.57671	0.00505	0.041526
Lmod1	0.522875	3.1215	9.970794	0.006155	0.04624
Sim1	-0.52272	5.168634	15.62591	0.001159	0.017709
Slc29a2	0.522252	3.90088	16.80298	0.000854	0.015072
Mbnl1	-0.52184	6.093566	24.7918	0.00014	0.005365
Abcc3	-0.52109	4.513553	10.0468	0.006002	0.045684
Sox7	0.520981	4.485467	18.10597	0.000617	0.012645
Rps21	-0.52096	7.545838	18.41289	0.000573	0.012071
Mcoln3	-0.52063	4.885678	18.88588	0.000511	0.0114
Snrnp27	-0.52003	4.990947	18.06442	0.000623	0.012731
Acer3	-0.51964	4.014483	11.64149	0.003611	0.034416
Gm10154	-0.51963	4.400795	13.68223	0.001975	0.02418
Nup210	0.519488	6.563003	21.47548	0.000282	0.008129
Dll4	0.519323	5.311423	28.00876	7.53E-05	0.00373
Cd47	-0.51855	5.761005	32.74536	3.27E-05	0.002413
Sigmar1	0.518504	5.718782	27.53424	8.23E-05	0.003944
G2e3	-0.51821	5.987483	15.64217	0.001154	0.017679
Lnpk	-0.51811	4.156351	11.51863	0.00375	0.035237
Papln	0.517949	4.9484	14.48434	0.001578	0.021317
Ecscr	0.517637	4.597139	20.86907	0.000323	0.008864
1700066M21Rik	-0.51676	3.593778	11.99426	0.003242	0.032378
Creb1	-0.51674	6.486622	22.67113	0.000218	0.006997
Trmt61a	0.516666	3.906458	15.19749	0.001299	0.019002
N4bp2l2	-0.51649	6.187238	20.08751	0.000386	0.009785
Zswim3	0.516297	3.209533	10.1623	0.005778	0.044853
Ndufa13	-0.51597	6.182395	17.36493	0.000741	0.013897
Zfp612	-0.51586	3.947619	10.00654	0.006083	0.045948

Slc3a1	-0.51573	6.508982	17.33236	0.000747	0.013952
F8	-0.51528	3.478099	9.947016	0.006204	0.046466
Bclaf1	-0.51513	7.580979	22.08563	0.000247	0.007542
Fam120aos	-0.51487	4.465794	13.14723	0.002303	0.026594
Celf2	-0.51479	4.316435	11.82524	0.003413	0.03341
Aldh18a1	0.514717	6.021535	25.37871	0.000125	0.00499
Arhgef15	0.514461	5.941846	31.921	3.75E-05	0.00257
Tlr3	-0.51378	3.33019	9.941681	0.006215	0.046509
Itga5	0.513448	6.701384	38.16112	1.39E-05	0.001421
Phf20l1	-0.51329	6.419441	20.47255	0.000353	0.00941
Igf2r	0.512784	8.117834	36.43969	1.8E-05	0.001616
Pabpc4l	-0.5127	3.237137	10.4628	0.005239	0.042336
B230118H07Rik	-0.51266	4.245656	16.71927	0.000872	0.015226
Ktn1	-0.51241	6.94086	24.4582	0.00015	0.005626
Nkain4	0.512344	3.659759	11.26991	0.004052	0.036833
Tuba1a	0.511654	7.83667	32.19047	3.59E-05	0.002496
Pdcd10	-0.51118	4.958561	21.13448	0.000305	0.008545
Jrkl	-0.51103	3.879655	10.18455	0.005736	0.044674
Map3k2	-0.51097	5.563494	12.95878	0.002432	0.027268
Adam19	0.510626	6.505929	30.4523	4.84E-05	0.00298
Zfp809	-0.5103	4.03777	14.23471	0.001691	0.022261
Rbbp8	-0.51011	5.233235	20.59232	0.000344	0.009284
Ccng1	-0.50977	5.93531	31.29258	4.18E-05	0.002735
2007-09-01	-0.50893	7.51551	17.04513	0.000803	0.014567
Nipbl	-0.50865	6.926562	26.26939	0.000105	0.0045
Krcc1	-0.50795	5.465147	18.18995	0.000604	0.012513
Slc8a2	0.507267	3.199328	11.74595	0.003497	0.033766
Cntnap1	0.50697	3.858816	16.41786	0.000942	0.015727
Cenpw	-0.50671	3.783272	13.45191	0.002109	0.02528
Rnf170	-0.50669	4.557055	17.94531	0.000642	0.012873
Actg1	0.506598	9.922964	22.55761	0.000223	0.007056
Hyal1	0.506561	3.077812	9.831772	0.006446	0.047736
mt-Nd1	-0.5064	11.0717	20.17094	0.000379	0.009699
Polr2a	0.505665	8.038157	23.60243	0.000179	0.006263
Zranb2	-0.50559	7.129317	24.23617	0.000157	0.005778
Gipc2	-0.50449	5.17743	19.14426	0.000481	0.011039
Ppp1r18	0.504223	5.727473	33.34262	2.96E-05	0.002218
Glmn	-0.50402	3.686208	11.35548	0.003945	0.036168
Mier3	-0.50401	5.313598	16.43743	0.000937	0.015692
Bmpr2	-0.50374	6.839494	19.67261	0.000425	0.010394
Foxm1	0.503113	6.467514	19.44704	0.000448	0.010715
Elmo1	0.502647	4.501428	19.75029	0.000417	0.010323
H2afj	-0.50245	6.205189	20.43945	0.000356	0.009453
Mpc2	-0.50235	6.956886	32.29716	3.52E-05	0.002484
Dleu2	-0.50226	4.862454	10.97827	0.004442	0.038603
Etfrf1	-0.50086	4.964385	23.85014	0.00017	0.006082
Pola1	-0.5004	5.397131	18.42603	0.000571	0.012071

Fam57a	0.500266	4.187968	17.63586	0.000692	0.013476
Zfp51	-0.50017	3.112906	10.45043	0.00526	0.042407
Pnpla8	-0.5	6.23228	22.83085	0.000211	0.00689
H2afz	-0.49981	5.882848	29.43132	5.8E-05	0.003344
Hells	-0.49953	5.6324	16.82012	0.00085	0.015043
Cntln	-0.49935	5.008973	15.53388	0.001187	0.017991
Neto2	-0.49926	5.167305	19.98513	0.000395	0.009952
Vamp7	-0.49924	4.838283	11.73403	0.00351	0.033816
Rpl23a	0.499098	8.751193	26.05134	0.000109	0.004616
Neat1	-0.49907	8.423002	13.18852	0.002275	0.026443
Crip1	0.498476	4.889528	22.03037	0.00025	0.007594
Rpsa-ps2	0.498392	3.624467	9.680917	0.006779	0.049138
Sh3bp2	0.498295	4.00545	13.00409	0.0024	0.027106
Wdr62	0.498188	3.759219	12.41527	0.002856	0.030253
Gm5148	-0.49796	4.440936	11.79583	0.003444	0.033565
Snx13	-0.4976	5.718585	29.9021	5.34E-05	0.003206
Arfip1	-0.4976	4.571818	20.19392	0.000377	0.009664
Mfap1b	-0.49745	3.6781	12.90238	0.002473	0.027539
Nceh1	-0.49724	6.024347	17.40979	0.000733	0.013857
Tent5a	-0.49722	3.400749	10.58363	0.005039	0.041452
Ccne2	-0.49698	4.177732	9.66566	0.006814	0.049262
Vegfd	0.496708	4.918362	19.2783	0.000466	0.010882
Slc35a3	-0.49599	5.925864	16.91757	0.000829	0.014862
Lrch2	-0.49594	4.764807	17.57192	0.000704	0.013587
Ssb	-0.4958	7.188251	20.94985	0.000317	0.008807
Sh3yl1	-0.49567	4.479754	17.53431	0.00071	0.013619
Rfesd	-0.49564	3.277095	9.858968	0.006388	0.047444
Mospd1	-0.49537	4.311521	11.63721	0.003616	0.034426
Plagl2	0.495289	5.670987	15.8536	0.001091	0.017104
Ankrd13c	-0.49507	5.937176	26.07806	0.000109	0.004603
Smim10l1	-0.49469	5.979985	23.11907	0.000198	0.006617
Vcpip1	-0.49465	5.63751	19.65594	0.000426	0.010396
Zfp280d	-0.49443	5.501307	16.7085	0.000874	0.015235
Zfp948	-0.49414	4.209636	14.05009	0.00178	0.022888
Abca5	-0.49402	3.771345	10.40407	0.00534	0.042694
Wnt9b	-0.49326	5.411145	15.31199	0.00126	0.018658
Suz12	-0.49278	6.430809	18.03598	0.000628	0.012757
Gm6863	0.492734	6.809654	24.0738	0.000162	0.005915
3830406C13Rik	-0.49266	4.625038	14.26608	0.001676	0.022172
Smim8	-0.49251	3.439275	11.55606	0.003707	0.03498
Wdr54	0.492418	3.589988	10.82391	0.004665	0.039536
Gsdme	-0.49206	4.521795	16.08147	0.001028	0.016477
Pdgfd	-0.49173	4.411426	15.47623	0.001206	0.018113
Cdh5	0.491663	7.803588	34.01715	2.65E-05	0.002119
Pdlim2	0.491235	5.512876	29.81918	5.41E-05	0.003243
Tmf1	-0.491	5.304097	24.84561	0.000139	0.005341
Myo7a	0.490773	5.85584	27.79721	7.83E-05	0.003826

Atp1b2	0.490551	5.200086	28.38696	7.02E-05	0.00365
Zfp386	-0.49009	4.940368	19.14174	0.000481	0.011039
Ehd2	0.489918	6.973521	37.06898	1.64E-05	0.001571
Tmppe	0.489813	3.545129	10.44937	0.005262	0.042407
Cntfr	0.48971	5.993638	23.0627	0.000201	0.006671
Dnajc12	-0.48943	3.431422	11.03583	0.004362	0.038255
Ankrd26	-0.48887	4.840711	14.50923	0.001567	0.021268
Slc25a36	-0.48883	7.111437	22.94768	0.000205	0.006784
Rnf208	0.488632	4.327577	10.67486	0.004893	0.040569
Zfp526	0.488387	4.02859	13.26474	0.002226	0.026148
Tle6	0.487828	3.194053	10.13271	0.005835	0.045085
Zfp955b	-0.48713	4.538735	13.13589	0.00231	0.026628
Tax1bp1	-0.4869	7.195772	31.63279	3.94E-05	0.002632
Dclk1	-0.48689	5.103477	18.83819	0.000517	0.011455
Clca3a1	-0.48687	5.441781	18.03605	0.000628	0.012757
Znrf2	-0.4868	5.750114	23.33507	0.000189	0.006445
Trim23	-0.48672	5.002991	16.57797	0.000904	0.015486
Stat2	-0.48638	5.221534	15.8232	0.0011	0.017165
Syf2	-0.48628	6.010027	27.07811	8.97E-05	0.004129
Osgepl1	-0.48604	3.955667	11.77547	0.003465	0.033598
Ache	-0.48576	4.42509	16.72766	0.00087	0.015226
Nos3	0.485715	5.249493	21.29457	0.000294	0.008352
Ube2v1	0.485606	3.613404	11.73749	0.003506	0.03381
Apoe	-0.48559	7.987934	15.07622	0.001342	0.019346
Gm10297	-0.48494	3.783571	10.16492	0.005774	0.044835
2310022B05Rik	0.484878	7.260411	31.8394	3.81E-05	0.002579
Cir1	-0.48484	4.08025	12.49223	0.002791	0.029881
Zdhhc20	-0.48417	6.420306	28.39657	7.01E-05	0.00365
Nmrk1	-0.48408	4.477302	14.41391	0.001609	0.02149
Katnbl1	-0.48384	4.712073	12.51285	0.002774	0.029728
Luc7l3	-0.48365	7.761575	13.90995	0.001851	0.023413
Gm10698	-0.48353	4.824646	18.36867	0.000579	0.012157
Gm37494	-0.48342	5.95421	18.15263	0.00061	0.012535
Rpl7a	0.483413	7.935623	12.96595	0.002427	0.027244
Me1	-0.48341	4.882526	17.09455	0.000793	0.01443
Rpl10a	0.483307	6.776022	26.56962	9.89E-05	0.00433
Mterf3	-0.48308	5.056913	17.51415	0.000714	0.01365
Rps27l	-0.4828	6.055892	24.31655	0.000155	0.005747
Zfp101	-0.48248	5.286815	12.83409	0.002523	0.027857
Rc3h2	-0.48246	6.011441	19.51683	0.000441	0.010627
Rpl6l	-0.48239	4.283977	15.25748	0.001278	0.018835
Trim21	-0.48223	3.535815	12.00974	0.003226	0.032317
Mn1	0.482187	3.820507	12.8643	0.002501	0.02776
Slc1a1	-0.48201	4.876607	15.45912	0.001211	0.01812
Snx24	0.481263	3.919724	14.17139	0.001721	0.022459
Wipf3	0.48115	4.046267	15.68195	0.001142	0.017569
Spata7	-0.48087	3.561725	9.836568	0.006436	0.047699

Calcrl	-0.48053	5.894085	16.28517	0.000975	0.016047
Icosl	0.48038	4.26561	17.01448	0.000809	0.014635
Mutyh	0.480178	3.460887	10.45246	0.005257	0.042407
Chordc1	-0.48016	6.203451	14.78228	0.001454	0.020325
Syvn1	0.48012	6.108791	33.04444	3.11E-05	0.002305
Grwd1	0.479743	5.436888	20.33016	0.000365	0.009576
Zfp160	-0.47964	4.76431	14.88461	0.001414	0.020017
0610010F05Rik	-0.4796	4.43853	14.42629	0.001603	0.02149
Stag2	-0.47919	7.092604	23.72419	0.000175	0.006156
2810013P06Rik	-0.47901	4.170881	14.91278	0.001403	0.019935
Inka1	0.478867	4.312108	14.36238	0.001632	0.02172
Adcy3	0.478265	4.857695	15.15259	0.001315	0.019091
2610021A01Rik	-0.47822	4.227754	11.8165	0.003422	0.033416
Tceal9	-0.47767	7.401555	23.40975	0.000186	0.006383
Gls	-0.47757	7.58649	21.12066	0.000306	0.008558
Marcksl1	0.477312	8.943888	25.68684	0.000117	0.004843
Cdadc1	-0.47715	5.187752	20.19807	0.000376	0.009664
Actn1	0.476897	7.785708	30.81593	4.54E-05	0.002886
Plppr3	0.476389	6.492902	33.32694	2.97E-05	0.002218
Upf3b	-0.47634	5.423307	16.82649	0.000848	0.015043
Tmem30a	-0.47629	7.236773	23.91088	0.000168	0.006043
Pnn	-0.47621	7.603792	23.4363	0.000185	0.006383
Bdp1	-0.47612	5.696723	12.44732	0.002829	0.030131
Prex1	0.475901	6.053927	23.40896	0.000186	0.006383
Yme1l1	-0.47566	6.818019	19.27794	0.000466	0.010882
Zfp626	-0.47556	4.233138	12.24544	0.003005	0.030837
Tceal8	-0.47546	6.253947	20.11762	0.000383	0.00975
Nek7	-0.47523	6.244492	30.55023	4.76E-05	0.002952
Dnm3os	-0.47523	3.717961	9.600132	0.006966	0.049939
Mfap4	0.474818	6.918053	28.23125	7.23E-05	0.003692
Gm7367	-0.4748	4.543674	12.38033	0.002886	0.030284
Thsd7a	-0.47467	4.829524	15.80116	0.001106	0.017229
D430019H16Rik	0.47454	5.430591	17.66199	0.000688	0.013404
BC030867	0.474379	3.860198	11.46208	0.003817	0.03554
Skil	-0.47424	5.991927	14.09207	0.001759	0.022782
Sltm	-0.47423	6.628251	21.70688	0.000268	0.00788
Ndufc2	0.474082	6.030174	21.62829	0.000273	0.007951
Arsa	0.474033	4.869289	19.74134	0.000418	0.010323
Nek1	-0.47397	5.079291	14.92657	0.001398	0.019887
Arl6	-0.47356	4.295289	13.06331	0.002359	0.026766
Cetn2	-0.47354	5.410145	21.06137	0.00031	0.008631
Tcea1	-0.47341	6.036891	15.13553	0.001321	0.019117
Crem	-0.47317	4.140365	11.89275	0.003343	0.032985
Plxna1	0.472947	7.16721	25.1203	0.000131	0.005133
Cables2	0.472912	5.298464	22.77085	0.000213	0.00694
Mef2a	-0.47279	6.675572	27.71029	7.96E-05	0.003868
Tiparp	-0.47271	4.368729	14.97989	0.001378	0.019693

Nts	-0.47239	4.064893	14.55187	0.001548	0.021149
Gm6793	-0.4722	7.165929	27.43654	8.38E-05	0.003984
Rpusd1	0.472082	3.993104	14.61506	0.001522	0.02094
Mtus1	-0.47173	6.3583	30.85567	4.51E-05	0.002876
Zfp65	-0.47094	3.882929	11.81662	0.003422	0.033416
Slc5a8	-0.47078	6.18873	10.88404	0.004577	0.039177
Wapl	-0.47065	6.795951	20.88336	0.000322	0.008856
Cdc73	-0.47035	5.797629	14.03394	0.001788	0.022889
Rbm25	-0.47034	7.505449	17.47697	0.00072	0.013688
Reln	0.470206	6.172157	11.68273	0.003565	0.034186
Clec2d	-0.46929	4.42478	9.986197	0.006124	0.046101
Zfp366	0.469073	4.20037	13.59859	0.002022	0.024591
Smyd5	0.468841	5.864488	27.37477	8.48E-05	0.00401
Zdhhc21	-0.46868	5.614733	12.33769	0.002923	0.0305
Mfsd4b1	-0.46819	4.839885	12.40586	0.002864	0.030253
Pcm1	-0.46812	7.072069	19.34097	0.000459	0.010836
Zfp280c	-0.46791	5.040651	13.84461	0.001886	0.023546
Ndufc1	-0.46789	5.375287	15.86993	0.001086	0.017076
Mtdh	-0.46718	7.038743	23.0835	0.0002	0.006654
Pom121	0.467151	6.733356	17.53207	0.000711	0.013619
Arsk	-0.46623	4.382778	10.66679	0.004906	0.040603
Slc35a4	0.466148	7.016906	26.82989	9.41E-05	0.004193
Pfkfb3	0.46585	5.608165	15.20106	0.001298	0.018999
Crebl2	-0.46565	3.990123	10.85902	0.004613	0.03935
Nudc-ps1	0.465114	3.902944	11.0062	0.004403	0.038395
Zfp37	-0.46501	3.694807	9.676172	0.00679	0.049182
Washc4	-0.46317	5.659198	17.5607	0.000706	0.013591
Diaph2	-0.46316	4.095151	11.27778	0.004042	0.036771
Cul4b	-0.46313	5.679379	14.41954	0.001606	0.02149
Gm45855	0.462697	4.32409	10.10657	0.005885	0.045229
Wwp1	-0.46249	6.584326	19.02945	0.000494	0.011183
Plxnd1	0.462411	8.747479	26.12586	0.000108	0.004599
Tasor2	-0.46232	5.74862	15.47286	0.001207	0.018113
Flt4	0.462238	5.715069	23.78697	0.000172	0.006128
Sympk	0.462228	7.638771	27.14716	8.86E-05	0.004121
Mxra7	0.462065	4.833583	11.84988	0.003387	0.033252
Gm11993	0.462045	4.298007	16.68724	0.000879	0.015235
Cacna1h	0.46188	5.717229	26.51476	1E-04	0.004343
Cetn3	-0.46188	5.976079	23.41251	0.000186	0.006383
Pank3	-0.4618	6.603813	17.57368	0.000703	0.013587
Slc33a1	-0.46179	5.525716	19.91046	0.000402	0.010039
Ccnf	0.461752	5.879758	19.41572	0.000451	0.010736
Usp45	-0.46171	4.775255	13.69787	0.001966	0.024138
Pdcd5	-0.46159	4.248835	14.19147	0.001711	0.022428
Hook1	-0.46131	5.888901	17.48657	0.000719	0.013671
Mns1	-0.46125	4.245081	13.6041	0.002019	0.024586
Scaper	-0.46114	4.440652	16.89636	0.000834	0.014897

Rbm39	-0.46013	8.978352	18.41351	0.000573	0.012071
Zc2hc1a	-0.45977	4.895517	18.47351	0.000564	0.011981
Ramp2	0.459208	6.428716	21.86067	0.00026	0.007776
Appl1	-0.45916	6.220974	18.19485	0.000604	0.012513
Tex9	-0.45914	4.074739	10.28837	0.005545	0.043691
Mcts1	-0.45914	4.805068	11.6429	0.003609	0.034416
Enpp4	-0.45909	3.765623	10.75557	0.004768	0.039936
Sucnr1	0.457968	3.456486	11.03152	0.004368	0.038255
Brip1	-0.45789	4.03015	9.69977	0.006737	0.048968
Ttbk2	-0.4575	4.738597	11.0972	0.004278	0.037925
Scarf1	0.457494	5.061918	22.65403	0.000219	0.006997
Masp1	0.457127	3.433219	11.03955	0.004357	0.038255
Adamtsl1	0.456948	4.732183	11.86824	0.003368	0.033158
Srek1	-0.45667	7.15538	19.76611	0.000416	0.010313
Senp8	-0.45645	3.656986	9.718907	0.006694	0.048715
Slc17a1	-0.45619	5.741832	18.18485	0.000605	0.012513
Fra10ac1	-0.45604	4.528224	13.63644	0.002001	0.024428
St3gal1	0.45603	6.333708	24.65823	0.000144	0.005472
Tmem117	-0.45594	4.191831	13.1214	0.00232	0.026673
Syap1	-0.45591	6.066165	21.6708	0.000271	0.007916
Dnajb9	-0.45501	4.710331	15.74948	0.001121	0.017393
Orc4	-0.45499	5.302528	16.81625	0.000851	0.015043
Lepr	-0.4549	4.923336	20.0946	0.000385	0.009785
Kdelr1	0.454836	7.189595	26.72742	9.59E-05	0.004242
mt-Nd4	-0.45478	10.18771	22.47059	0.000227	0.0071
Sorbs2	-0.45457	5.775042	19.55341	0.000437	0.010564
Ccdc174	-0.45382	4.42661	15.30137	0.001263	0.018695
Mmut	-0.45346	6.160437	14.49221	0.001574	0.021287
Zscan22	0.453033	4.386994	13.31941	0.002191	0.025945
Senp7	-0.4526	5.43891	10.63227	0.00496	0.040996
Gtf3c5	0.452462	5.803149	25.76913	0.000116	0.004788
Noc4l	0.452003	5.161724	19.96892	0.000397	0.009971
Jak2	-0.45196	5.455611	21.4623	0.000283	0.008134
Zbtb33	-0.45179	5.405814	20.9234	0.000319	0.008846
Lars2	0.451575	6.594521	26.95063	9.19E-05	0.004189
Tuba1b	0.451281	9.563798	24.45148	0.00015	0.005626
Rsf1	-0.45128	6.247837	15.91811	0.001073	0.016936
P4ha2	0.451078	4.755069	14.07675	0.001767	0.022797
Mal2	-0.45052	6.367285	17.12065	0.000788	0.014381
Rpl12	0.450483	6.198294	25.95486	0.000111	0.004675
Plekhg2	0.450277	7.237604	31.40443	4.1E-05	0.002703
Csgalnact2	-0.45023	4.806433	14.89762	0.001409	0.019964
Abhd17b	-0.45013	4.766948	12.79092	0.002555	0.028136
Prdm11	0.44969	6.275275	17.36817	0.00074	0.013897
Rrp12	0.449154	4.673429	11.76767	0.003474	0.033615
Unc119	0.4487	4.977434	16.29223	0.000973	0.016047
Mapk11	0.448696	4.52954	17.35491	0.000743	0.013903

Col1a2	0.448235	10.35158	18.95231	0.000503	0.011306
Fbxo30	-0.44816	5.260575	13.7024	0.001963	0.024123
Egln3	0.447669	5.757363	14.53542	0.001555	0.021149
Ptges	-0.44747	5.845379	17.67463	0.000686	0.013378
Flvcr1	-0.44744	4.53174	10.48963	0.005194	0.042066
Tax1bp3	-0.44696	4.908977	17.81918	0.000662	0.01311
Myl9	0.446902	7.653929	24.72089	0.000142	0.00543
Tmem255a	0.446716	4.632511	13.89537	0.001859	0.023418
Susd2	0.446312	6.323421	19.23061	0.000471	0.010923
Carf	-0.44627	3.885395	10.01628	0.006063	0.045842
Ehd3	0.446233	5.665799	21.81251	0.000262	0.00779
Arhgdia	0.446227	9.046117	19.65881	0.000426	0.010396
Lgalsl	-0.44622	5.248844	13.44639	0.002112	0.025303
Vcam1	0.446102	5.566018	20.90205	0.000321	0.008848
Pycrl	0.445998	6.094604	25.84133	0.000114	0.004731
Tbl1xr1	-0.44595	5.75577	18.52211	0.000558	0.011913
Lsm10	0.445931	3.954473	11.96621	0.003269	0.032545
Cops2	-0.44577	6.69355	20.35077	0.000363	0.00956
Slc15a4	0.445712	4.475688	14.68791	0.001492	0.020693
Nav3	-0.44549	5.191485	14.21296	0.001701	0.022319
Acadm	-0.4447	7.695204	26.09206	0.000108	0.004602
Alkbh8	-0.44466	5.256289	12.2071	0.00304	0.031097
Prpf8	0.444372	8.675766	18.74277	0.000529	0.011596
Slc39a8	-0.44435	6.041207	12.96494	0.002428	0.027244
Snhg8	-0.44434	4.819192	12.42883	0.002844	0.030202
Phf2	0.444307	6.7256	19.31004	0.000462	0.010858
Ttyh3	0.444094	8.073308	18.0136	0.000631	0.012773
Mest	0.443771	9.996203	16.86228	0.000841	0.014976
Ccdc90b	-0.44365	5.076312	16.7201	0.000872	0.015226
Ap1b1	0.443211	7.05564	29.72725	5.5E-05	0.003274
Hmgn3	-0.44318	5.316764	19.38637	0.000454	0.010765
Cluh	0.443157	7.655924	27.72549	7.94E-05	0.003867
Adh1	-0.44265	5.947448	9.836699	0.006435	0.047699
Lypla1	-0.44193	6.126378	16.71659	0.000873	0.015226
Rpl14-ps1	-0.44187	7.601531	21.20924	0.0003	0.008462
Actn4	0.441737	9.240474	23.42273	0.000186	0.006383
Ubxn2b	-0.44173	4.70958	14.9354	0.001394	0.019872
Tmem167	-0.44148	6.067498	16.06791	0.001032	0.016521
Ctif	0.441474	4.961734	13.06221	0.00236	0.026766
Usp16	-0.44147	5.597109	16.77696	0.000859	0.015127
Rbm26	-0.44137	6.525124	13.08965	0.002341	0.026746
Pcdh1	0.44129	5.85737	20.90811	0.00032	0.008848
Mcam	0.441135	7.501476	28.10557	7.4E-05	0.003694
Gm7536	-0.44107	7.725262	15.22029	0.001291	0.018933
Eny2	-0.44096	6.349204	20.82041	0.000327	0.008913
Vil1	0.440774	6.060547	18.30251	0.000588	0.012295
Sat1	-0.44021	6.688799	15.48326	0.001203	0.018113

Kif18b	0.439898	5.371865	18.72439	0.000531	0.011614
Erap1	-0.4393	5.036876	11.77822	0.003462	0.033598
Aph1c	-0.43907	3.786037	10.32817	0.005473	0.043324
Vps26a	-0.43876	6.022583	22.56605	0.000223	0.007056
Uri1	-0.43876	6.086708	23.9651	0.000166	0.005988
Wdr46	0.4385	5.431843	15.81293	0.001103	0.017196
Cnot3	0.438469	6.823364	25.29528	0.000127	0.005033
Tmem44	0.43794	5.492895	20.49547	0.000352	0.009404
Gdpd5	0.437805	5.102385	18.66972	0.000538	0.011679
Purb	-0.43738	7.508673	17.86621	0.000654	0.013018
Pla2g16	-0.43699	4.347567	11.17322	0.004177	0.03742
Cpeb4	-0.43666	5.525826	13.73085	0.001948	0.024031
Pdia5	0.436628	5.531785	21.65451	0.000272	0.007931
Rpf2	-0.43656	4.325282	13.27344	0.00222	0.0261
Fgfr1op	-0.4365	5.463544	13.40926	0.002135	0.025505
ccdc198	-0.4363	5.839906	18.85404	0.000515	0.011455
Cspp1	-0.43563	5.873455	19.27997	0.000466	0.010882
Thoc1	-0.43527	5.333767	14.15412	0.001729	0.022489
St8sia1	-0.43496	4.72207	11.97613	0.00326	0.032484
Dhx36	-0.43491	6.330306	18.76533	0.000526	0.011572
Rac3	0.434785	3.869127	10.71824	0.004825	0.040204
Plk1	0.434546	6.331059	16.82175	0.000849	0.015043
Taf7	-0.43437	4.50431	12.59858	0.002704	0.02936
Mphosph8	-0.43428	5.196911	14.40141	0.001614	0.021548
Taok1	-0.43423	6.853325	19.45932	0.000446	0.010711
Jun	-0.43414	6.829361	15.52196	0.001191	0.018002
mt-Co1	-0.43411	12.88217	15.65039	0.001151	0.01767
Sardh	0.434028	6.990171	21.71937	0.000268	0.00788
Itga8	-0.43398	7.102613	18.64503	0.000541	0.011707
Cd55	-0.43398	3.77296	10.02717	0.006041	0.045753
Asah2	-0.43392	4.897533	13.89828	0.001858	0.023418
Rad50	-0.43388	5.143862	17.61945	0.000695	0.013497
Col25a1	0.433706	3.39545	9.798906	0.006517	0.048001
Lgals9	-0.43364	5.833927	18.49854	0.000561	0.011966
Mlst8	0.43355	5.667943	22.19504	0.000241	0.007423
Serbp1	-0.43324	8.838263	19.40898	0.000452	0.010736
Mbtps2	-0.43311	5.112416	14.76305	0.001461	0.020406
Sema4g	0.433059	6.728507	28.47356	6.91E-05	0.003646
Gng2	-0.43301	6.027564	25.39712	0.000124	0.00499
Med12l	-0.43298	3.904897	9.828364	0.006453	0.04777
Mtf2	-0.43268	5.741335	14.64756	0.001508	0.020852
Mrps18b	0.432626	4.171167	12.69337	0.00263	0.028713
Arl4a	-0.43216	5.150359	13.28991	0.002209	0.026028
Thop1	0.431803	6.108357	19.33155	0.00046	0.010845
Tasor	-0.43149	5.903119	14.69272	0.00149	0.020682
Rsad2	-0.4312	6.046853	20.30701	0.000367	0.009594
Smc3	-0.43111	6.801085	14.11655	0.001747	0.02266

Kdm4b	0.430974	6.547219	19.1391	0.000481	0.011039
Zim1	-0.43081	6.143843	23.9927	0.000165	0.005972
Plxna2	0.430592	5.206995	18.32313	0.000585	0.012249
Pdlim7	0.430556	6.437691	24.12432	0.000161	0.005866
Eloc	-0.43048	5.71939	16.39684	0.000947	0.015789
Cdkl1	-0.43022	6.130393	17.76373	0.000671	0.013202
Ccdc14	-0.43012	4.121973	10.36882	0.005401	0.042981
Trim28	0.429962	8.994688	16.14284	0.001012	0.016379
Nid2	0.429892	7.971198	23.2762	0.000192	0.006495
Sema3a	-0.42912	5.408312	16.25637	0.000982	0.016109
Krit1	-0.42878	5.650568	11.08008	0.004301	0.038071
Hspg2	0.428771	9.236734	17.29665	0.000754	0.014018
Pkn2	-0.42875	6.658636	19.15848	0.000479	0.011039
Vcl	0.428652	6.786497	19.68655	0.000423	0.010383
Fyttd1	-0.42845	6.622259	12.96684	0.002427	0.027244
Plscr3	0.428393	6.421789	20.75629	0.000332	0.009001
Naa80	0.428298	4.575386	13.11219	0.002326	0.026692
Tapbp	-0.42806	5.903561	15.42421	0.001222	0.018216
Pik3c2a	-0.42779	5.987357	11.99803	0.003238	0.032378
Cnot6l	-0.42735	7.116652	18.43826	0.000569	0.012069
Fabp5	0.427182	6.644446	20.56306	0.000346	0.009312
Dnaaf5	0.426981	5.148501	17.69375	0.000683	0.013358
Tmem170b	-0.42693	4.451236	11.45721	0.003823	0.035563
Atg9a	0.42682	5.355224	19.238	0.00047	0.010923
Eif2s2	-0.4267	6.441998	22.91694	0.000207	0.006789
Rsbn1	-0.42665	4.72999	11.03019	0.00437	0.038255
D030045P18Rik	-0.42645	4.062942	10.05439	0.005987	0.045684
Hpf1	-0.42645	5.192708	13.58556	0.00203	0.024665
Rb1	-0.42641	5.360769	10.55511	0.005085	0.0417
Klhl24	-0.42598	7.021743	24.19299	0.000159	0.005796
Rabep1	-0.42567	5.91213	19.12337	0.000483	0.011039
Akr1b3	-0.42554	5.994103	19.51645	0.000441	0.010627
Atp5a1	0.425494	5.50424	16.51734	0.000918	0.015579
Scoc	-0.4251	5.211637	13.21104	0.00226	0.026365
Rps16-ps2	0.424961	6.972229	11.48702	0.003787	0.035359
D930016D06Rik	-0.42492	4.828613	12.93834	0.002447	0.027353
Ints5	0.424545	5.65814	14.2229	0.001696	0.022274
Bcl9l	0.423839	6.697103	18.41746	0.000572	0.012071
Aasdhppt	-0.42355	4.934749	16.50482	0.000921	0.015579
Cyp2d26	0.423545	7.020941	15.70713	0.001134	0.017544
Hoxc10	0.423451	5.673756	20.69944	0.000336	0.009089
Trim2	-0.42343	6.23603	21.0961	0.000307	0.008582
Dctd	0.42325	5.45191	21.36161	0.00029	0.008269
Nynrin	0.423227	5.995558	17.812	0.000663	0.013119
Hoxd3	-0.42306	3.944805	10.44627	0.005267	0.042407
Itm2a	-0.42305	5.756325	20.76333	0.000331	0.009001
Pdzk1	-0.42278	7.746903	15.46716	0.001209	0.018113

Mob1b	-0.42259	5.729273	10.89899	0.004555	0.039136
Atp6v0d2	-0.42239	3.927822	11.19425	0.004149	0.037268
Rps24-ps3	-0.42239	5.314045	10.04513	0.006006	0.045684
Snapc3	-0.42201	5.080002	16.96322	0.00082	0.014736
Nol7	-0.42188	6.21745	24.49734	0.000149	0.005609
Acvr2a	-0.4217	5.360039	11.018	0.004386	0.038346
Frk	-0.42115	4.887197	11.31834	0.003991	0.036439
6430548M08Rik	0.421111	4.611386	9.818731	0.006474	0.047864
Zfp862-ps	0.420838	4.967866	13.91447	0.001849	0.023413
Zfp932	-0.42072	4.659766	15.43611	0.001219	0.018201
Stag1	-0.42069	6.483108	20.77719	0.00033	0.008986
Smarcad1	-0.42063	5.935032	13.76688	0.001928	0.023877
Fam76b	-0.4203	5.70883	13.71585	0.001956	0.024098
Rfx3	-0.42005	5.066169	16.87814	0.000837	0.014951
Tcf7l1	0.419978	6.157508	15.28629	0.001268	0.018756
Rnft1	-0.41995	5.183395	12.20276	0.003044	0.031119
Fgd1	0.419939	5.717955	18.80721	0.000521	0.011514
Rps10-ps2	0.419485	7.583143	17.55759	0.000706	0.013591
Ppp2r3a	-0.41901	6.115802	21.03015	0.000312	0.008678
Ddx10	-0.41865	5.038263	14.98377	0.001376	0.019692
Rps6ka2	0.418463	5.66028	15.68792	0.00114	0.017567
Rgp1	0.418453	6.621199	23.88693	0.000169	0.00606
Adcy4	0.418448	5.139924	16.36981	0.000954	0.015835
Gm42418	-0.41835	6.921799	12.58442	0.002716	0.029411
Sdc2	-0.41834	7.035541	19.69538	0.000423	0.010383
Mvb12b	0.418121	6.276153	17.55821	0.000706	0.013591
Ccne1	0.418094	4.403342	12.65074	0.002663	0.028999
Platr25	-0.41805	3.968972	10.12798	0.005844	0.045088
Naa15	-0.41786	6.559263	15.32079	0.001257	0.01863
Gm6136	-0.41742	6.697858	17.93182	0.000644	0.012882
Iars	0.416989	6.938506	22.92557	0.000206	0.006789
Rab3il1	0.416698	4.959563	17.75444	0.000672	0.013203
Pdik1l	-0.41662	4.543751	10.44121	0.005276	0.042407
Sox18	0.416413	6.060665	11.23574	0.004096	0.037048
Serf1	-0.41622	4.772388	14.04634	0.001782	0.022888
Olfml2b	0.416065	4.569859	11.52407	0.003744	0.035219
Adamts2	0.41606	4.987139	17.61645	0.000696	0.013497
Senp6	-0.41595	7.157983	18.78764	0.000523	0.011549
Gps1	0.415809	6.223815	20.26808	0.00037	0.009594
Odr4	-0.41576	5.172141	13.92388	0.001844	0.023405
Rgma	0.415628	4.911302	12.58356	0.002716	0.029411
Lsm2	0.415358	5.492322	13.88514	0.001864	0.023418
9430015G10Rik	0.415336	5.794123	16.39509	0.000948	0.015789
Zyx	0.415079	7.260017	17.10054	0.000792	0.014423
Rhog	0.414891	5.093862	14.55156	0.001549	0.021149
Fgfr4	0.414305	5.780654	20.3787	0.000361	0.009527
Stab1	0.414286	6.794624	20.15928	0.00038	0.009699

Elovl7	-0.41422	5.110561	10.75987	0.004762	0.039936
Gypc	0.413293	4.841277	16.2199	0.000992	0.016152
Wdr55	0.413193	4.802664	15.83644	0.001096	0.017151
Rcc1	0.413021	5.834498	20.70033	0.000336	0.009089
Rps3a2	0.412996	5.420121	9.774038	0.006571	0.048235
Iqsec1	0.412968	6.683487	23.59225	0.000179	0.006264
Zfp691	0.412937	3.996738	11.4789	0.003797	0.035411
Anapc13	-0.41292	4.92064	10.04881	0.005998	0.045684
Ypel5	-0.41282	5.001284	14.74205	0.00147	0.020492
Znrd1	0.412616	4.863966	13.80808	0.001906	0.023691
Atp5md	-0.4126	6.106741	14.26812	0.001675	0.022172
Cep162	-0.41255	4.67412	10.96811	0.004456	0.038619
Smchd1	-0.4124	6.82416	13.96957	0.001821	0.023172
Cox11	-0.41231	4.137342	10.91202	0.004536	0.039078
Serpinf1	0.412195	5.259332	12.52048	0.002768	0.029685
Rpp30	-0.41189	3.736897	9.960145	0.006177	0.046343
Stxbp6	-0.41167	5.538967	18.17961	0.000606	0.012513
Pcbp4	0.411424	6.877624	25.60023	0.000119	0.004904
Dld	-0.41116	7.321399	19.96446	0.000397	0.009971
Pnisr	-0.41083	8.078266	10.83468	0.004649	0.039476
Cdt1	0.410828	5.841283	12.58245	0.002717	0.029411
Trnt1	-0.41056	5.461699	14.2356	0.00169	0.022261
Lrp1	0.410509	8.316783	15.15328	0.001315	0.019091
Polr2j	0.4103	5.205438	11.61055	0.003645	0.034636
Rpl9	0.41016	8.315631	21.09416	0.000307	0.008582
Lgr4	-0.40999	7.76428	23.20438	0.000195	0.006574
Tead3	0.409809	6.103968	23.15739	0.000197	0.006594
Bmt2	-0.4098	5.193522	11.10492	0.004268	0.037888
Kiz	-0.40968	4.855731	15.68939	0.001139	0.017567
Phtf2	-0.40945	4.447588	10.01481	0.006066	0.045842
Fbxl3	-0.40909	6.400885	20.25904	0.000371	0.009594
Prpf4b	-0.40898	7.538737	13.1477	0.002302	0.026594
Gemin5	0.408849	5.927911	16.6434	0.000889	0.015317
Sufu	0.408764	6.740773	23.51993	0.000182	0.006334
Kctd12	-0.40863	4.905719	15.85873	0.00109	0.017096
Ampd2	0.408595	6.666973	22.49978	0.000226	0.007094
Rnf44	0.408372	7.640316	22.67458	0.000218	0.006997
Kdr	0.40798	7.24293	25.95839	0.000111	0.004675
Mybl2	0.407871	4.765706	9.640329	0.006872	0.049538
Mrpl43	0.407753	5.259246	13.78138	0.00192	0.023822
Cox16	-0.40765	4.157882	9.773059	0.006573	0.048235
Ctdspl2	-0.40758	5.801919	11.37657	0.00392	0.036024
Plod1	0.407272	6.555192	19.68972	0.000423	0.010383
Mgrn1	0.407173	7.31912	18.17743	0.000606	0.012513
6330403K07Rik	0.407079	4.975301	12.51155	0.002775	0.029728
mt-Nd5	-0.40694	10.53446	11.36845	0.00393	0.036077
Gpr182	0.406801	4.838495	13.06472	0.002358	0.026766

Dgkz	0.406465	7.097409	22.0192	0.000251	0.007599
Pacs1	0.405999	6.285924	19.40979	0.000452	0.010736
Mob4	-0.40595	5.835386	20.3993	0.000359	0.009502
Syncrip	-0.40588	7.630358	10.47716	0.005215	0.042197
Trpc1	-0.40573	4.2136	10.09432	0.005909	0.045332
Zfyve16	-0.4055	5.363571	11.93234	0.003303	0.032681
Samd4b	0.4055	6.711989	19.60147	0.000432	0.010475
Gm10232	-0.4054	4.491556	12.32301	0.002936	0.030598
Rap1a	-0.4052	5.982133	13.27923	0.002216	0.026078
Dph2	0.405098	4.199316	10.5019	0.005173	0.042012
Pacs2	0.405092	6.815845	22.5372	0.000224	0.007062
Rasa1	-0.40508	6.248363	12.34992	0.002912	0.030424
Cpne3	-0.40502	6.233052	13.97655	0.001817	0.023143
Coro2b	0.404976	5.984738	19.87758	0.000405	0.010101
Ift81	-0.40494	5.290549	10.70584	0.004845	0.040343
Prr5	0.404764	5.357389	11.04185	0.004353	0.038255
Fbn1	0.404564	7.510854	19.1267	0.000483	0.011039
Retreg1	-0.40445	5.781799	16.11058	0.00102	0.016437
Cdc42ep1	0.404347	6.199756	16.12395	0.001017	0.016415
Klhl9	-0.40425	7.005304	13.18659	0.002276	0.026443
Fam133b	-0.40412	4.984224	9.957775	0.006182	0.04636
Mfge8	0.40387	7.684548	19.05493	0.000491	0.011162
Nt5dc2	0.403792	7.705704	13.18702	0.002276	0.026443
Usp49	0.403731	4.708716	12.40707	0.002863	0.030253
Rock2	-0.40373	6.585649	10.81982	0.004671	0.039552
Rplp2	0.403577	9.084787	11.71988	0.003525	0.033926
Pnpt1	-0.40353	5.503615	14.45725	0.001589	0.021408
Naa50	-0.40337	7.141797	12.78136	0.002562	0.028164
Cldn11	0.402919	5.863042	17.16348	0.000779	0.014292
Fbxo10	0.402898	5.421138	14.81806	0.00144	0.020207
Tspan4	0.402839	5.979844	17.58611	0.000701	0.013584
Smim3	-0.40265	4.65246	13.9961	0.001807	0.023072
Luc7l2	-0.40262	7.901891	16.57792	0.000904	0.015486
Hspa13	-0.40259	5.747404	12.16664	0.003077	0.031442
Cand2	0.402386	6.244488	15.5554	0.001181	0.017904
Dcakd	0.402369	7.10803	13.35797	0.002167	0.02571
Reep4	0.402236	5.355016	15.94581	0.001065	0.016874
Fundc1	-0.40211	5.179479	10.89109	0.004566	0.039177
Zcchc7	-0.40211	5.455061	15.49385	0.0012	0.018091
Ncoa7	-0.40185	5.57465	11.50285	0.003769	0.035297
Efemp2	0.401454	6.913686	17.40642	0.000733	0.013857
Rc3h1	-0.40103	6.067848	11.7936	0.003446	0.033569
Sipa1l2	0.400826	6.597073	17.53668	0.00071	0.013619
Cdkn1b	-0.40082	6.55083	20.08325	0.000386	0.009785
Gpd1	0.4006	6.856406	15.46729	0.001209	0.018113
Fkbp10	0.399809	7.14871	16.10846	0.001021	0.016437
Ica1	-0.39976	4.212893	9.971334	0.006154	0.04624

Tead2	0.399701	7.643773	24.49798	0.000149	0.005609
Pdgfrb	0.399251	7.860298	16.22941	0.000989	0.016146
Pde3b	-0.39893	4.544025	11.92593	0.00331	0.032726
Marcks	-0.3989	8.216982	14.97531	0.001379	0.019693
Fancm	-0.3988	4.740005	11.59192	0.003666	0.03478
1600012H06Rik	-0.39876	5.388814	16.66673	0.000884	0.015286
Zfp644	-0.39862	6.011881	13.86001	0.001878	0.023461
Carm1	0.398507	6.791004	19.64583	0.000427	0.010396
Selenon	0.39839	6.683805	18.72635	0.000531	0.011614
Atm	-0.3983	5.292797	10.44308	0.005273	0.042407
Dnm1l	-0.39807	6.949708	14.04471	0.001783	0.022888
Kri1	0.398064	4.580943	9.890066	0.006322	0.047094
Gpr153	0.397733	5.144763	14.4536	0.001591	0.021408
Aspa	-0.39752	5.255095	10.73055	0.004806	0.040138
Mfsd6	-0.39752	4.345806	12.09952	0.00314	0.031865
Zfp592	0.397459	6.319398	17.24735	0.000763	0.014125
Plgrkt	-0.39743	4.568097	12.12263	0.003118	0.031703
AW011738	-0.39731	4.487477	12.75041	0.002586	0.028315
Ppp4r2	-0.39711	6.169918	10.17988	0.005745	0.044674
Fmo2	-0.39709	5.290273	10.30816	0.005509	0.043499
Mybbp1a	0.397063	8.030188	15.14567	0.001317	0.019098
Sirt1	-0.39669	5.172892	11.82086	0.003417	0.033416
Prmt1	0.396621	8.070667	23.29698	0.000191	0.006484
Rbm12	-0.39661	4.759935	10.83975	0.004642	0.039469
Trpc3	0.396296	4.352102	12.37914	0.002887	0.030284
Prkaa2	-0.39629	5.541365	14.50401	0.001569	0.021283
Acsl3	-0.39597	6.52702	18.78411	0.000524	0.011549
Ephb2	0.395864	6.01069	11.56785	0.003694	0.034926
Myc	0.395693	6.264117	13.48211	0.002091	0.025147
Ehf	-0.39548	5.538069	13.38785	0.002148	0.025566
Nemf	-0.39526	6.126509	11.08383	0.004296	0.038045
Fam135a	-0.39522	6.0593	13.81211	0.001903	0.023691
Mapkbp1	0.394986	5.513209	18.55365	0.000553	0.011851
Col12a1	0.394853	7.727486	11.67726	0.003571	0.034225
Ptges3	-0.39462	6.51315	12.87147	0.002495	0.027737
Syngap1	0.394457	6.15053	20.16302	0.000379	0.009699
Hs1bp3	0.394428	5.002767	14.82007	0.001439	0.020207
Clcnka	-0.39442	5.554208	17.78314	0.000668	0.013164
Ireb2	-0.39432	7.194666	13.57212	0.002038	0.024692
Mapk7	0.394263	6.309033	18.66962	0.000538	0.011679
Flnc	0.394247	5.961365	11.34065	0.003964	0.036261
Map2k3	0.394238	6.329223	21.33518	0.000291	0.008304
Trps1	-0.39411	5.832175	16.69158	0.000878	0.015235
Pnpo	0.394035	5.014534	11.93222	0.003303	0.032681
Dab2ip	0.393904	7.114323	18.63907	0.000542	0.011708
Kdelr3	0.393886	4.561861	11.73097	0.003513	0.033829
Crebzf	-0.39387	7.066678	16.45356	0.000933	0.015671

Azi2	-0.39387	6.062039	20.24739	0.000372	0.009594
Llph	-0.39362	4.548588	12.07581	0.003163	0.031994
Aplnr	0.39359	7.381436	19.26548	0.000467	0.010886
Mgp	-0.39325	6.751378	10.88948	0.004569	0.039177
Ramac	-0.39317	5.282499	11.71001	0.003536	0.034009
Mmp14	0.393128	7.997604	16.2106	0.000994	0.016164
Kirrel	0.393126	7.025924	20.1295	0.000382	0.00975
CT010467.1	0.392844	8.442475	16.46207	0.000931	0.015652
Mbip	-0.39281	4.14158	10.31816	0.005491	0.043384
Msmo1	-0.39276	5.398367	13.22401	0.002252	0.026312
Ndufaf4	-0.3927	4.851207	11.25006	0.004078	0.036968
Kifc1	0.392391	6.122571	19.67008	0.000425	0.010394
Abtb2	0.392188	4.290897	12.14081	0.003101	0.031629
Ruvbl2	0.392164	6.754354	19.02485	0.000495	0.011183
Cyp4a31	0.392154	6.135172	17.41167	0.000732	0.013857
Epn1	0.392034	7.408524	15.77942	0.001113	0.017287
Celsr1	0.391951	6.599857	13.36991	0.002159	0.025656
Gm10131	0.391944	5.268723	14.69514	0.001489	0.020682
Map7	0.391799	6.627804	19.72378	0.00042	0.010337
Fmr1	-0.39124	6.495374	11.41879	0.003868	0.035777
Polr2f	-0.3912	5.108699	12.81969	0.002534	0.027951
Lnpep	-0.39101	6.773104	9.64677	0.006858	0.049482
Rps6kb1	-0.3909	5.981445	14.17956	0.001717	0.022445
Uchl5	-0.39059	4.659951	10.06808	0.00596	0.045587
Arhgef10	0.390487	6.179701	18.42621	0.000571	0.012071
Pfkl	0.389963	8.430102	14.18307	0.001715	0.022442
Tie1	0.389669	7.49428	16.65319	0.000887	0.015309
Ostf1	-0.38945	6.557539	17.25002	0.000762	0.014125
Tbc1d23	-0.38924	5.683335	16.08792	0.001026	0.016477
Prkch	0.389079	4.92019	11.65616	0.003595	0.034337
Rad23a	0.388524	5.813571	19.5866	0.000433	0.010497
Arl6ip6	-0.38851	5.181483	11.94273	0.003293	0.032614
Ccar1	-0.38845	7.024502	10.56125	0.005075	0.041652
Mlh3	-0.38837	4.61427	9.950621	0.006196	0.046438
Mplkip	-0.3883	4.81963	14.04441	0.001783	0.022888
Ints9	0.38827	5.115511	11.53665	0.00373	0.035116
Tbrg4	0.388219	6.218196	18.48687	0.000562	0.011971
Itpr3	0.38799	6.820102	21.2585	0.000296	0.008406
Npat	-0.38768	5.233947	12.13173	0.00311	0.031647
Rnf126	0.387664	6.286112	14.49218	0.001574	0.021287
Tm9sf4	0.387521	6.861128	21.69602	0.000269	0.007885
Dnajc3	-0.38743	6.705678	15.64209	0.001154	0.017679
Txlng	-0.38735	4.543771	12.40019	0.002869	0.030253
Zfp523	0.387344	6.41779	15.23857	0.001285	0.018892
Xrcc3	0.387323	5.177069	13.17453	0.002284	0.02647
Nbl1	0.387319	4.623952	9.684411	0.006771	0.0491
Smarca5	-0.38707	6.785229	11.41392	0.003874	0.035812

Lgals1	0.386953	7.459204	11.03231	0.004367	0.038255
Tmub2	0.386887	5.816434	17.67426	0.000686	0.013378
1810055G02Rik	0.386715	4.87561	11.04082	0.004355	0.038255
Arhgap45	0.386257	4.518578	11.12688	0.004238	0.03774
Tmem132a	0.385986	6.952381	17.05847	0.0008	0.014532
S1pr2	0.385693	5.498636	13.87412	0.00187	0.023418
Xrn1	-0.38561	5.970524	14.42169	0.001605	0.02149
Tedc2	0.385537	4.126232	10.93726	0.0045	0.038843
Scrn3	-0.38549	4.651897	10.96767	0.004457	0.038619
Bbip1	-0.38542	4.794242	12.25977	0.002992	0.030824
Gga2	0.385107	7.167782	22.16959	0.000243	0.007445
Fkbp3	-0.38507	6.662598	16.47359	0.000929	0.015635
Dhcr7	0.38495	5.633078	17.68228	0.000685	0.013378
Col2a1	-0.38492	5.704501	12.40371	0.002866	0.030253
Tmem9b	-0.38491	5.398131	14.19015	0.001712	0.022428
L3mbtl2	0.38486	4.721517	11.24925	0.004079	0.036968
Mad1l1	0.384629	5.379207	12.9053	0.002471	0.027539
Arap3	0.384305	6.683818	20.20897	0.000375	0.009659
Prep	0.384279	6.520021	16.20775	0.000995	0.016164
Rgs3	0.384244	5.917639	19.74369	0.000418	0.010323
Esam	0.384187	6.100349	18.00101	0.000633	0.012795
Mocs2	-0.38388	5.53271	14.61334	0.001522	0.02094
Strap	0.383765	7.288137	16.44191	0.000936	0.015692
Crym	0.383643	5.176906	14.16054	0.001726	0.022482
Fbxo21	0.383617	6.921674	16.09835	0.001023	0.016465
Gys1	0.383576	6.540009	20.27872	0.000369	0.009594
Acsf2	0.383377	6.081188	16.54524	0.000912	0.015556
Nnat	0.383321	5.752618	14.53518	0.001556	0.021149
Fbln2	0.383184	5.234594	13.57275	0.002037	0.024692
Ncstn	0.383035	7.435991	16.19751	0.000997	0.016177
E2f4	0.382994	6.376152	21.83968	0.000261	0.007776
Dmpk	0.382923	5.484925	11.77527	0.003466	0.033598
Gm6563	-0.38292	5.445262	13.6146	0.002013	0.024563
Bcar1	0.382369	7.051382	16.37355	0.000953	0.015835
Reep1	0.382253	4.268183	10.75517	0.004769	0.039936
Notch1	0.382187	7.236522	10.69867	0.004856	0.040395
Cyp39a1	-0.38215	4.260233	10.32135	0.005486	0.043359
Cspg4	0.382037	6.647583	13.09769	0.002336	0.026727
Eif4ebp1	0.381907	5.50932	11.15485	0.004201	0.037579
Qtrt1	0.381771	4.981817	13.11562	0.002324	0.026683
Casc4	-0.38171	5.861005	18.40171	0.000574	0.012089
Kctd15	0.381642	6.522143	19.76368	0.000416	0.010313
Uck1	0.381243	5.917021	13.66871	0.001982	0.024254
Eif1ad	0.381202	5.725868	17.16157	0.00078	0.014292
Xiap	-0.38113	7.11227	14.39329	0.001618	0.02158
Bod1l	-0.38048	6.134803	14.51069	0.001566	0.021268
Wdr18	0.380445	6.273956	20.05879	0.000389	0.009826

Cerk	0.380403	5.366646	16.87161	0.000839	0.014961
Mcm3	0.380388	7.318407	12.944	0.002443	0.027325
Mxd3	0.380292	4.451162	10.81561	0.004678	0.039584
Rpl10a-ps1	0.37986	5.957816	13.56394	0.002042	0.024716
Hook3	-0.37985	6.77009	11.13937	0.004222	0.037678
Tmem119	0.379399	4.968217	14.17461	0.001719	0.022459
Srgap1	0.379373	6.185687	17.73646	0.000675	0.013247
Impdh1	0.379008	5.309767	14.16661	0.001723	0.02246
Zdhhc9	0.378824	5.936388	16.56098	0.000908	0.015524
Trpm7	-0.37858	7.000711	11.03792	0.004359	0.038255
Slc12a2	-0.37844	6.994538	13.0659	0.002358	0.026766
Arl5b	-0.37815	5.975162	10.10055	0.005897	0.045259
Zbtb10	-0.3781	5.539048	15.96731	0.001059	0.016824
Igfbp4	0.377987	9.48235	13.0787	0.002349	0.026766
Plod3	0.377921	6.834357	19.50182	0.000442	0.010636
Rsbn1l	-0.3778	5.130029	10.98231	0.004436	0.038572
Nrp2	0.377754	5.94073	11.22799	0.004106	0.037063
Sub1	-0.37772	7.030888	19.10115	0.000486	0.011062
Ofd1	-0.37765	4.169674	9.810189	0.006492	0.04792
Aff3	0.377445	5.759856	15.89875	0.001078	0.016992
Cpn1	0.377125	6.906129	16.79159	0.000856	0.015088
Actr2	-0.37703	7.440967	13.17239	0.002286	0.02647
Pbrm1	-0.37694	6.898998	13.54076	0.002056	0.024847
Dync2h1	-0.37665	5.714443	9.630436	0.006895	0.049605
Vars	0.376549	7.54338	15.27462	0.001272	0.018769
Naa40	0.376478	6.668502	19.10285	0.000486	0.011062
Bet1	-0.37646	5.200365	11.35128	0.003951	0.036197
Tmed7	-0.37641	7.150629	13.78109	0.00192	0.023822
Tpr	-0.37631	7.489154	16.36943	0.000954	0.015835
Impdh2-ps	0.376157	6.088508	13.74144	0.001942	0.023991
Dysf	0.375987	5.948055	18.06385	0.000623	0.012731
Mbnl2	-0.37574	6.966041	10.49926	0.005178	0.042012
Ergic1	0.375717	7.667854	16.51411	0.000919	0.015579
Slk	-0.37565	7.373691	14.62576	0.001517	0.02094
Selenot	-0.37544	7.65164	16.34332	0.00096	0.015897
Atr	-0.37539	5.632789	11.81571	0.003423	0.033416
Ube2w	-0.37523	5.325542	10.78835	0.004718	0.039759
Foxk1	0.374934	6.507966	13.30952	0.002197	0.025957
Psme3	0.374632	7.101128	19.26681	0.000467	0.010886
Ccdc50	-0.37439	7.519013	15.06211	0.001347	0.019388
Ttc33	-0.37427	4.62011	9.799599	0.006515	0.048001
Ssh1	0.374259	5.437911	12.90397	0.002472	0.027539
Gli2	0.374249	4.892483	10.00046	0.006095	0.04597
Rpgr	-0.37423	4.320307	9.651573	0.006846	0.049422
Smg9	0.374046	5.123538	9.902579	0.006296	0.046978
Tmem25	0.373947	5.91596	12.02301	0.003214	0.032308
Col6a3	0.373891	7.620305	14.82862	0.001435	0.020181

Arl15	-0.37374	5.489248	15.79287	0.001109	0.017242
Txn2	0.37344	7.018718	17.93916	0.000643	0.012873
Impad1	-0.37338	7.195369	10.82659	0.004661	0.039521
Tdrp	-0.37316	4.476118	10.59431	0.005021	0.041368
Zkscan8	-0.37311	5.911704	14.18264	0.001715	0.022442
Kdm7a	-0.37267	5.570483	9.736778	0.006654	0.048544
Sh2b1	0.372606	6.999134	22.0722	0.000248	0.007551
Slc16a12	-0.3726	5.687417	10.06877	0.005959	0.045587
Mphosph10	-0.37253	4.598343	10.76388	0.004755	0.039936
Gm10039	0.372376	6.443784	19.40355	0.000452	0.010736
Morf4l1-ps1	-0.3719	5.423717	13.71124	0.001959	0.024113
Flnb	0.371749	7.790628	18.60094	0.000547	0.011771
Proser3	0.371745	4.597985	12.90226	0.002473	0.027539
Vmp1	-0.3717	6.959835	16.89883	0.000833	0.014897
Dusp9	0.371665	5.496892	14.02527	0.001792	0.022928
Trim32	0.37153	6.12436	13.43647	0.002118	0.025358
Ipo4	0.371509	5.846596	11.58627	0.003673	0.034803
Ift27	0.371424	4.461281	11.06697	0.004319	0.038191
Tmem201	0.370713	6.568505	17.95006	0.000641	0.012873
Megf9	-0.37059	5.762985	15.11995	0.001326	0.019181
Lamtor1	0.370264	6.16804	16.63933	0.00089	0.015318
Kat2b	-0.37026	4.918734	12.35395	0.002909	0.030423
Rmc1	0.370204	4.88125	13.64234	0.001997	0.024417
Cdh4	0.369944	5.602199	11.26336	0.004061	0.036861
Eif5b	-0.36987	6.917914	13.88105	0.001867	0.023418
Ifrd2	0.369853	5.315498	14.72024	0.001479	0.020591
Sf3a2	0.369724	6.128184	11.5082	0.003763	0.035285
Rangap1	0.369635	7.063386	14.28533	0.001667	0.022103
Cab39l	-0.36939	5.556341	12.13305	0.003108	0.031647
Spindoc	0.369144	5.452407	13.93286	0.00184	0.023362
Emc2	-0.36851	5.411613	14.31935	0.001651	0.021928
Zfand3	0.368243	7.148747	19.44303	0.000448	0.010715
Kcnk1	-0.36821	5.337645	15.37037	0.00124	0.018415
Rph3al	0.36808	4.718554	11.87931	0.003357	0.033102
Rlim	-0.36796	6.623776	9.766068	0.006589	0.04829
Ruvbl1	0.367954	6.110732	15.40698	0.001228	0.018282
Smarcd1	0.367806	6.762689	13.88213	0.001866	0.023418
Kcnh2	0.367398	4.961471	11.50736	0.003764	0.035285
Crb2	0.367296	4.494434	9.860364	0.006385	0.047442
Synj2bp	-0.36723	5.568608	10.33766	0.005456	0.043273
Clock	-0.367	5.43361	10.85019	0.004626	0.039376
Arrdc3	-0.36677	7.077319	11.9104	0.003325	0.032863
Nacc1	0.366742	7.545309	13.91982	0.001846	0.023409
Pitpnm2	0.366708	6.105716	16.72747	0.00087	0.015226
Zfp414	0.366663	4.95301	11.44691	0.003835	0.035614
Pigs	0.366623	6.484587	16.23878	0.000987	0.016138
Guf1	-0.36644	6.247702	11.95274	0.003283	0.032599

2900026A02Rik	0.366437	6.257296	13.69139	0.00197	0.024149
Pgm2l1	-0.36638	4.90847	10.10571	0.005887	0.045229
Shoc2	-0.3661	5.903267	11.37877	0.003917	0.036024
Trip4	-0.36608	5.406052	10.37349	0.005393	0.042935
Ppp2r1a	0.365791	8.492417	14.27504	0.001672	0.02215
Tubb6	0.3657	6.306337	14.42586	0.001603	0.02149
Sh3bp4	0.365622	6.25977	17.82422	0.000661	0.013109
U2af2	0.365606	8.468836	17.37568	0.000739	0.01389
Slc40a1	-0.36558	6.802392	17.39641	0.000735	0.013877
Tspan9	0.365562	7.642603	16.28674	0.000975	0.016047
Slc25a22	0.36535	5.249337	12.95132	0.002438	0.0273
Gm49336	-0.36526	5.765115	9.759312	0.006604	0.048359
2004-09-01	-0.36525	6.144966	17.33789	0.000746	0.013947
Msi1	0.364916	5.32857	12.84226	0.002517	0.027857
Sp3	-0.3645	6.692836	15.55708	0.00118	0.017904
Urb1	0.364356	4.800835	12.75793	0.00258	0.028276
Rgs19	0.364306	4.563813	9.884947	0.006333	0.047115
Dnajb4	-0.3641	5.136089	12.40152	0.002868	0.030253
Sgpp1	-0.36394	5.31677	10.16101	0.005781	0.044853
Ubxn7	-0.36367	6.679925	12.81182	0.002539	0.027998
H19	0.363408	11.39991	14.9113	0.001403	0.019935
Strada	0.363398	5.064858	11.097	0.004278	0.037925
Slmap	-0.36299	6.517581	13.09187	0.00234	0.026746
Speg	0.362793	6.528397	14.61562	0.001522	0.02094
Larp7	-0.36272	5.714069	10.74655	0.004782	0.039989
Pex2	-0.3627	5.707577	17.0369	0.000804	0.014582
Hsdl2	-0.36268	5.138682	11.65306	0.003598	0.034351
Rpl37	0.362601	7.531801	10.26048	0.005596	0.043974
Cnn2	0.36259	8.277582	15.8789	0.001084	0.017066
Wdr86	0.362537	5.837923	15.25503	0.001279	0.018835
Eml1	0.362526	6.474063	18.39677	0.000575	0.012089
Evi5	-0.36249	6.404854	14.03382	0.001788	0.022889
Uba3	-0.36245	6.17223	11.21437	0.004123	0.037146
1700017B05Rik	0.362297	5.559358	10.18199	0.005741	0.044674
Prpf19	0.362035	7.870864	17.21731	0.000769	0.014181
Lasp1	0.362	7.917264	16.67664	0.000882	0.015262
Eif3a	-0.36185	8.557402	13.64043	0.001998	0.024417
Myo9a	-0.36176	6.897266	13.42398	0.002126	0.025414
Usp1	-0.36173	6.692582	13.36169	0.002164	0.0257
Traf4	0.361714	6.986615	17.1749	0.000777	0.014282
Ccdc47	-0.36147	6.955268	12.35026	0.002912	0.030424
Clk1	-0.36141	7.712602	10.90397	0.004548	0.039102
Mmp11	0.361361	5.552926	12.53897	0.002753	0.02957
Ssx2ip	-0.36109	5.411093	12.06545	0.003172	0.03203
Scfd1	-0.36103	5.471083	12.99709	0.002405	0.027127
Paxbp1	-0.361	6.502924	10.52735	0.005131	0.041934
Gm46430	-0.36072	4.661624	11.15741	0.004198	0.037568

Dram2	-0.36052	5.725716	14.74085	0.00147	0.020492
Ppp1r10	0.360329	6.509527	11.50285	0.003769	0.035297
Supt6	0.360012	7.298217	19.04841	0.000492	0.011163
Eif4a-ps4	0.359919	7.448055	20.04121	0.00039	0.009852
Gsr	-0.35991	6.759944	15.66555	0.001147	0.017615
Mast2	0.359714	6.564883	10.88908	0.004569	0.039177
Serinc1	-0.35952	7.547817	16.74902	0.000865	0.015205
Ube2s	0.359495	6.114737	18.71194	0.000533	0.011623
Myef2	-0.35937	6.33251	10.79535	0.004708	0.039736
Ufl1	-0.35931	5.578326	13.23165	0.002247	0.026306
Tmem123	-0.35886	6.921888	18.83769	0.000517	0.011455
Cxadr	-0.35883	6.456344	9.871411	0.006361	0.047288
Nr3c1	-0.35875	6.255422	14.03737	0.001786	0.022889
Ltbp1	0.358695	7.841896	15.18854	0.001302	0.019016
Sema4d	0.358555	6.497538	17.95906	0.000639	0.012873
Angptl2	0.358526	6.462198	15.27466	0.001272	0.018769
Ddb1	0.358432	9.390941	15.95621	0.001062	0.016858
Ptp4a2	-0.35843	7.702012	15.63847	0.001155	0.01768
Sf3a1	0.358359	7.225064	12.0063	0.00323	0.032333
Jagn1	0.358091	5.315819	12.58448	0.002716	0.029411
2310022A10Rik	0.357961	4.972605	10.79753	0.004705	0.039736
Hira	0.357815	5.23512	12.02194	0.003215	0.032308
Zfp148	-0.35752	6.503186	11.42219	0.003864	0.035758
Cmtm7	0.357476	5.238699	12.23529	0.003014	0.030888
Fbxl19	0.35722	6.906907	16.10821	0.001021	0.016437
3110039I08Rik	-0.35687	6.089094	10.03822	0.006019	0.045684
Uhrf1	0.3567	7.481471	12.63068	0.002679	0.029154
Zkscan7	-0.35639	4.594098	9.960585	0.006176	0.046343
Hps1	0.356336	4.704881	10.05698	0.005982	0.045684
Nop2	0.356295	5.919616	16.24477	0.000985	0.016128
Ube2c	0.356104	6.70038	12.54687	0.002746	0.029564
Hcfc1	0.355936	8.02901	13.24749	0.002237	0.026244
Stim1	0.355755	4.741232	11.14656	0.004212	0.037658
Slc12a7	0.355663	6.437896	17.80624	0.000664	0.013123
Psip1	-0.35532	7.321382	14.70468	0.001485	0.020647
Rpl35	-0.35525	8.5154	12.3007	0.002955	0.03069
Mrc2	0.354856	7.009341	12.41834	0.002853	0.030253
Micall1	0.35478	6.382726	17.71382	0.000679	0.013307
Crip2	0.353943	6.82734	12.05038	0.003187	0.032121
Hsf1	0.353907	6.193636	17.38296	0.000737	0.013886
Tns2	0.353772	6.986367	17.32597	0.000748	0.01396
Dele1	0.353741	6.314265	15.5927	0.001169	0.017804
Arid1a	0.353441	8.281528	14.46841	0.001584	0.021378
Zfp385a	0.352926	5.122249	11.31152	0.004	0.036479
Magt1	-0.35274	6.655892	10.50114	0.005175	0.042012
Cast	-0.35259	5.501785	11.86607	0.003371	0.033162
Ago2	0.35239	7.33972	11.96843	0.003267	0.032542

Bcorl1	0.352104	5.441361	13.38796	0.002148	0.025566
Lrrc59	0.352061	7.629196	16.79096	0.000856	0.015088
Gnl1	0.352038	6.249049	15.00708	0.001368	0.01966
Pkm	0.351961	10.31696	10.96555	0.00446	0.038626
Numbl	0.351744	5.414993	12.66861	0.002649	0.028882
Tonsl	0.351699	5.185351	12.00032	0.003236	0.032373
Togaram1	-0.35153	5.630752	12.01094	0.003225	0.032317
Kctd10	0.351385	6.601464	16.81454	0.000851	0.015043
Oser1	0.351306	4.31325	10.0311	0.006034	0.045752
Tdp1	0.351291	5.10608	10.0325	0.006031	0.045751
Mrto4	0.351057	4.983488	12.22913	0.00302	0.030909
Scin	-0.35098	5.907359	12.59631	0.002706	0.029362
Eln	0.350691	7.888222	12.94931	0.002439	0.0273
Etaa1	-0.35064	4.882618	10.8982	0.004556	0.039136
Dchs1	0.35045	7.261013	11.4797	0.003796	0.035411
Cdc37l1	-0.35039	6.303325	10.63982	0.004948	0.040918
Rpl36a-ps2	0.350191	6.765508	10.27143	0.005576	0.043875
Atp5e	-0.35004	6.873953	9.732841	0.006662	0.048568
Ptn	-0.35	8.268876	13.14246	0.002306	0.026596
Kctd11	0.349951	4.374387	10.05459	0.005987	0.045684
Dr1	-0.34945	6.029393	12.77977	0.002563	0.028164
Ncapd2	0.349247	7.981547	13.11704	0.002323	0.026683
Ubl4a	0.349102	6.004314	14.23296	0.001691	0.022261
Dipk2a	-0.34884	6.0905	11.6999	0.003547	0.03407
Impdh2	0.348769	6.344307	11.39468	0.003898	0.03599
Strn4	0.348739	7.188468	17.50968	0.000715	0.01365
Katnb1	0.348356	5.405778	11.20176	0.00414	0.037218
Atxn10	0.348225	7.598279	14.34152	0.001641	0.021826
Atad1	-0.34822	6.720595	11.95425	0.003281	0.032599
Slc35b2	0.348088	6.466793	14.83754	0.001432	0.020164
Pprc1	0.347938	6.125649	12.31438	0.002943	0.030623
Rnf144a	0.347436	6.475972	15.08805	0.001338	0.01931
Srprb	0.347373	5.618002	14.5472	0.00155	0.021149
Tcf4	-0.34737	6.882096	13.19628	0.00227	0.026443
Tpt1	0.347301	9.338795	11.13849	0.004223	0.037678
Upf3a	-0.34726	5.56918	11.20729	0.004133	0.037172
Ptpn23	0.347253	6.753902	17.49216	0.000718	0.013671
Tgm2	0.347087	6.320789	17.32028	0.000749	0.013965
Ednrb	-0.34684	6.541304	13.23167	0.002247	0.026306
Gas2	-0.34669	6.991003	13.74978	0.001937	0.023967
Ephb3	0.346462	6.268285	12.25326	0.002998	0.030835
Ndufa4l2	0.346385	5.080638	10.51059	0.005159	0.041985
Fpgs	0.346116	5.805289	13.99037	0.00181	0.023072
Smarca4	0.346015	8.078887	11.13344	0.00423	0.0377
Zfp335	0.345947	5.306295	11.02863	0.004372	0.038255
Ptma	0.345919	10.18437	14.36193	0.001632	0.02172
Met	-0.34592	6.479649	16.48087	0.000927	0.015621

Dhx37	0.345795	5.433831	12.44407	0.002831	0.030131
Rps6ka1	0.345781	6.073183	11.21806	0.004119	0.037141
Gmfb	-0.34554	6.857312	14.04733	0.001781	0.022888
Ipo13	0.345404	5.932998	13.56867	0.00204	0.0247
Sp2	0.3454	5.028202	11.0453	0.004349	0.038255
Adarb1	0.34528	4.753803	10.51407	0.005153	0.041965
Mafb	0.34483	6.617181	15.84302	0.001094	0.017136
Mpp2	0.344318	5.439773	12.39646	0.002872	0.030253
Natd1	0.344238	5.984151	13.20255	0.002266	0.026412
Zbtb7c	0.344223	4.891449	10.62039	0.004979	0.041098
Mcm5	0.343776	7.192344	12.01759	0.003219	0.032314
Pcdhgc3	0.343696	5.638114	9.942649	0.006213	0.046509
Usp11	0.343666	6.374076	16.4222	0.000941	0.015724
Msantd4	-0.34353	6.047558	13.73065	0.001948	0.024031
Obsl1	0.343524	7.658655	14.87905	0.001416	0.020017
Sumo3	0.343013	7.433451	16.49855	0.000923	0.015579
Ccdc124	-0.34265	5.841525	11.35993	0.00394	0.036137
Cndp2	-0.34233	7.865234	12.8499	0.002511	0.027843
Prxl2a	-0.34228	6.338688	14.233	0.001691	0.022261
Gbf1	0.342082	6.78623	14.44166	0.001596	0.021455
Mef2d	0.341897	6.663676	15.5668	0.001177	0.017881
Tssc4	0.341855	5.578118	11.94605	0.003289	0.032599
Dixdc1	-0.34183	4.689169	10.14965	0.005803	0.044962
Mier2	0.341366	5.868241	13.43287	0.00212	0.025367
Abcf2	0.34122	6.602107	15.11165	0.001329	0.019209
Ubr4	0.341171	7.879847	14.81399	0.001441	0.020214
Zmiz1	0.340682	8.045952	12.34079	0.00292	0.030489
Arhgap1	0.340567	6.923661	11.51211	0.003758	0.035285
Rap1gap2	0.340555	5.774806	11.14137	0.004219	0.037678
Tns3	0.34055	7.918302	16.48128	0.000927	0.015621
Stxbp3	-0.34041	4.815338	11.30592	0.004007	0.036497
Hoxd9	-0.34008	6.698859	11.51987	0.003749	0.035237
Xpo5	0.340063	6.752468	16.08517	0.001027	0.016477
Ap1g2	0.339986	4.806898	10.66749	0.004905	0.040603
Atxn3	-0.33989	5.080801	10.13069	0.005839	0.045085
Gna12	0.339702	7.186099	12.26036	0.002991	0.030824
Aftph	-0.33967	5.809555	13.00094	0.002403	0.027114
Mmp15	0.339521	5.619239	11.3492	0.003953	0.036201
Foxc1	-0.33914	6.025045	10.44029	0.005278	0.042407
Zfp687	0.339113	6.30701	15.82692	0.001099	0.017165
Dnajb6	-0.33899	5.841111	10.23567	0.005641	0.044254
Lyar	-0.33891	5.420898	10.59993	0.005012	0.041312
Yipf4	-0.3388	5.675887	11.43819	0.003845	0.035686
Atxn7l3	0.338635	6.780559	14.55972	0.001545	0.021149
Rmnd5b	0.338624	5.292951	11.52371	0.003745	0.035219
Ttll12	0.338339	5.849328	12.42515	0.002847	0.030211
Naa30	-0.33803	5.649772	12.5755	0.002723	0.029419

Mllt1	0.337933	6.970565	12.60433	0.0027	0.029341
Ythdf3	-0.33791	7.334827	10.6156	0.004987	0.041142
Baz2b	-0.33787	6.338003	11.14255	0.004217	0.037678
Mgat3	0.337577	6.173649	13.47323	0.002096	0.025194
Hsd11b2	0.337495	6.301896	10.44234	0.005274	0.042407
Atp8a1	-0.33737	5.639707	11.01315	0.004393	0.038386
C1qbp	0.337334	7.121904	10.73433	0.004801	0.040108
Rbbp6	-0.33732	7.134803	12.44667	0.002829	0.030131
Lama1	0.337285	7.964303	11.05229	0.004339	0.038255
Traf7	0.337156	7.089704	17.46173	0.000723	0.013726
Bop1	0.337142	5.912072	10.80405	0.004695	0.039711
Pnkd	0.337103	6.313282	15.18445	0.001304	0.019022
Dvl3	0.337077	6.849041	17.38522	0.000737	0.013886
Hax1	0.337069	5.666312	14.16933	0.001722	0.022459
Denr	-0.33691	5.945757	12.24961	0.003001	0.030837
Fuca2	-0.33666	6.959394	12.07875	0.00316	0.031994
Wiz	0.336593	6.926475	11.76859	0.003473	0.033615
Sall2	0.33643	6.747146	12.04547	0.003192	0.032133
Uimc1	0.336255	7.58378	13.5806	0.002033	0.024683
Ifitm2	0.336144	7.41797	12.69371	0.002629	0.028713
Mettl1	0.336011	4.754108	10.38318	0.005376	0.042877
Rnf168	-0.33598	5.365523	9.733114	0.006662	0.048568
Col6a2	0.335973	8.423992	12.00977	0.003226	0.032317
Zfp260	-0.33594	6.429094	10.10384	0.005891	0.045229
Zfand1	-0.33588	4.999915	10.1111	0.005877	0.045229
Tbx3	-0.33582	6.472115	13.17555	0.002284	0.02647
Atp8b1	-0.33582	4.759394	10.44579	0.005268	0.042407
Ppp1cb	-0.33561	7.957775	13.15584	0.002297	0.02658
Nsf	0.33544	5.79177	12.30013	0.002956	0.03069
Ube3b	0.335311	7.113623	12.75712	0.002581	0.028276
G6pdx	0.335303	5.622345	10.45849	0.005246	0.042376
Capn15	0.335273	6.225616	14.53956	0.001554	0.021149
Sh2d4a	-0.33511	4.725995	10.21474	0.00568	0.04444
Zfp646	0.335112	6.380599	12.83633	0.002521	0.027857
Pwp1	0.334875	5.759402	12.98554	0.002413	0.027171
Pfdn2	0.334593	5.896378	12.2732	0.00298	0.030775
Msto1	0.334422	5.656869	10.02805	0.00604	0.045753
Nek6	0.334314	5.605652	12.7681	0.002572	0.02822
Ksr1	0.334003	5.339665	10.77836	0.004733	0.039838
Cyb5r3	0.333968	7.123468	13.10626	0.00233	0.026703
Ppif	0.333949	6.032331	14.99811	0.001371	0.019663
Ptprf	0.33363	8.488058	12.12018	0.00312	0.031703
Nol9	0.333521	5.847021	13.99421	0.001808	0.023072
Mal	-0.33322	7.381217	15.23761	0.001285	0.018892
Pten	-0.33305	6.288461	10.33629	0.005459	0.043273
Pkdcc	0.332864	8.079617	14.79974	0.001447	0.020277
Rxra	0.332842	7.056721	14.22639	0.001694	0.02227

Arl5a	-0.33281	6.84754	11.37495	0.003922	0.036024
Vim	0.332687	9.367545	12.26057	0.002991	0.030824
Hspa8	0.332659	10.33122	11.98532	0.00325	0.032411
Nudt21	-0.33252	5.818686	10.35925	0.005418	0.043077
Col5a1	0.332496	8.179618	11.55133	0.003713	0.035001
Prrc2a	0.332288	9.350362	9.604287	0.006956	0.04991
Pds5b	-0.33195	6.81517	9.749974	0.006624	0.04843
Glis2	0.331924	7.287499	15.47394	0.001206	0.018113
Tdp2	-0.33185	5.822402	10.42044	0.005312	0.04263
Tgfb1i1	0.331853	6.430032	10.90715	0.004543	0.039084
Spaca6	-0.33169	6.261657	14.53919	0.001554	0.021149
Uba1	0.331588	8.861503	14.61361	0.001522	0.02094
Ap2a1	0.331382	6.822359	11.54696	0.003718	0.035022
Csde1	-0.33129	8.126609	12.90846	0.002468	0.027539
Avl9	-0.33115	5.523237	13.18492	0.002278	0.026443
Hoxd10	-0.33113	6.135725	10.40329	0.005341	0.042694
Robo4	0.330905	5.988921	13.51932	0.002069	0.024965
Prmt5	0.330818	6.768431	15.40227	0.00123	0.018282
Sec24c	0.330737	7.826484	16.62828	0.000892	0.015332
Cplx2	0.330619	5.696635	13.09656	0.002337	0.026727
Gm10282	0.330583	7.880094	15.00131	0.00137	0.019662
Mcm2	0.330542	7.256739	10.06461	0.005967	0.04562
Tspan7	0.330477	6.160872	14.41566	0.001608	0.02149
Mmab	0.330387	5.754145	14.12593	0.001743	0.022625
Nr2f2	-0.33022	8.124605	12.27302	0.00298	0.030775
Cmpk1	-0.33011	6.535214	13.05421	0.002366	0.026782
Atp13a1	0.330087	6.867093	16.68878	0.000879	0.015235
Mpp6	-0.33007	5.897202	13.08059	0.002348	0.026766
Ocln	-0.33005	5.198667	11.04912	0.004343	0.038255
Dll1	0.330015	5.755586	9.697344	0.006742	0.048988
Vcp-rs	0.329934	6.526991	10.30734	0.005511	0.043499
Vat1	0.329625	6.855078	15.79904	0.001107	0.017229
Ece1	0.329438	7.134348	14.04748	0.001781	0.022888
Rab11fip2	-0.32943	5.189465	10.02933	0.006037	0.045753
Lrrc58	-0.32938	6.973041	9.895425	0.006311	0.04703
Lemd3	-0.32937	5.181192	10.85844	0.004614	0.03935
Col3a1	0.32931	10.77102	11.23017	0.004103	0.037057
Sar1b	-0.32929	5.569083	12.47457	0.002806	0.029953
Flna	0.329139	10.26544	12.06237	0.003175	0.032041
Smad3	0.329	7.191132	11.59954	0.003658	0.034716
Coro7	0.328912	5.650203	12.98745	0.002412	0.027171
Snhg17	0.328854	6.474067	16.35816	0.000957	0.015866
Dgkh	-0.32826	5.48184	9.752612	0.006618	0.048407
St6galnac2	-0.32826	5.344059	11.44821	0.003833	0.035614
Bgn	0.328197	8.172101	11.56447	0.003698	0.034926
Pls3	-0.32818	7.417484	13.96129	0.001825	0.02321
Ccm2	0.328072	5.576633	9.915077	0.00627	0.046823

Ddx19a	0.327927	6.043101	11.13613	0.004226	0.037687
Cdca4	0.327903	5.9721	10.14527	0.005811	0.044968
Zranb1	-0.32787	6.267813	12.28947	0.002965	0.030726
Pou3f3	-0.32771	7.126522	15.61384	0.001162	0.01775
Gnas	0.327319	9.465344	11.26662	0.004056	0.036843
Sec24d	0.327316	6.414991	10.97609	0.004445	0.038611
Smg5	0.327101	6.870943	15.33924	0.001251	0.018553
Cic	0.327032	7.665675	15.13937	0.00132	0.019112
Uck2	0.326976	6.974925	14.93536	0.001394	0.019872
Grk2	0.326965	6.758894	13.0977	0.002336	0.026727
Adck1	0.326822	4.803443	9.992221	0.006112	0.046046
Pter	0.326815	6.282685	13.06265	0.00236	0.026766
Man1a	-0.32672	5.643918	10.54606	0.0051	0.041783
Kifc5b	0.326708	4.717593	9.822166	0.006467	0.047849
Ankrd52	0.3267	7.014639	12.47405	0.002806	0.029953
Adgra3	0.326506	7.701957	13.48711	0.002088	0.025128
Coq6	0.326463	5.233523	10.538	0.005113	0.041853
Scarb1	0.326452	6.379468	12.87488	0.002493	0.027726
Copg1	0.326397	8.0427	14.92809	0.001397	0.019887
Ubc	0.32639	6.339593	10.04132	0.006013	0.045684
Prkaca	0.325763	6.837694	14.33736	0.001643	0.021835
Ier3ip1	-0.32576	5.403383	10.7837	0.004725	0.039799
Col4a3bp	-0.32565	6.265478	13.88746	0.001863	0.023418
Atp6v1h	-0.32552	6.266907	11.6126	0.003643	0.034632
Phf3	-0.32546	6.891586	10.97194	0.004451	0.038619
Atad3a	0.325282	7.028204	13.05626	0.002364	0.026782
Zmiz2	0.325062	6.985873	12.4761	0.002805	0.029953
Erg28	-0.32502	5.373731	11.63289	0.00362	0.034453
Insig1	-0.32486	5.766367	12.38504	0.002882	0.030284
Mars	0.324849	6.282885	11.03281	0.004366	0.038255
Pa2g4	0.324836	7.631253	13.84019	0.001888	0.023559
Zfp362	0.324684	6.717807	10.80009	0.004701	0.039723
Eef1g	0.324549	8.944078	10.53203	0.005123	0.041895
Shmt2	0.324528	6.979183	13.83308	0.001892	0.02359
Ubap2l	0.324517	7.940574	15.63001	0.001157	0.017705
Cntrl	-0.32441	6.376982	12.37416	0.002891	0.030302
Ccdc97	0.324369	5.899338	12.74441	0.00259	0.028347
Tbl2	0.324279	5.872151	12.98501	0.002414	0.027171
Med12	0.324266	6.754309	13.60761	0.002017	0.024578
Dync1h1	0.324107	8.773549	12.12051	0.00312	0.031703
Mrtfa	0.3241	5.715152	10.24923	0.005616	0.0441
Tmsb4x	-0.32376	9.508642	10.90704	0.004543	0.039084
Glg1	0.323697	8.263578	12.09164	0.003147	0.031922
Ift80	-0.32364	5.664587	10.4084	0.005332	0.042694
Cks1b	0.323593	6.297973	14.83054	0.001435	0.020181
Hectd4	0.32356	6.450246	11.0549	0.004336	0.038255
Efhd1	-0.32329	5.390972	9.675833	0.006791	0.049182

Psmc6	-0.32288	6.786427	12.53883	0.002753	0.02957
Plin2	0.322814	5.766961	10.90691	0.004544	0.039084
Tfe3	0.322801	5.684799	12.07248	0.003166	0.031998
Actr1b	0.322583	7.459605	14.43199	0.0016	0.02149
Tbc1d22b	0.322431	5.562829	11.32751	0.00398	0.036372
Golgb1	-0.32237	7.215833	10.86875	0.004599	0.039276
Slc39a5	0.322254	6.427885	12.83323	0.002523	0.027857
Usp21	0.322057	6.134378	14.60037	0.001528	0.020966
Ccdc8	0.32198	6.868894	12.54435	0.002748	0.029564
Foxk2	0.321906	6.576155	15.06895	0.001345	0.019368
Gaa	0.321857	7.39695	10.46303	0.005239	0.042336
Pdcd4	-0.32176	6.472425	13.30545	0.0022	0.025957
Stk40	0.321694	6.200551	13.04904	0.002369	0.026792
Frem2	0.32167	7.852793	9.873949	0.006356	0.047268
U2af1	0.321664	6.094503	11.24289	0.004087	0.037004
Fgfr1op2	-0.32155	6.511705	12.41317	0.002858	0.030253
Prmt7	0.321403	5.621127	13.71586	0.001956	0.024098
Plxna3	0.321271	5.735302	10.80125	0.004699	0.039723
Ppan	0.32126	6.062621	13.87699	0.001869	0.023418
Nme2	0.321166	7.048744	11.95738	0.003278	0.032596
Pros1	-0.32114	5.793969	12.53763	0.002754	0.02957
Rpap1	0.321087	5.966354	11.63994	0.003613	0.034416
Llgl1	0.321068	6.913775	10.5168	0.005148	0.041947
Necap1	0.320923	5.473265	11.25625	0.00407	0.036925
Odc1	0.320868	8.332696	10.52356	0.005137	0.041938
Egfl7	0.320716	7.430664	10.2012	0.005705	0.044519
Zmym5	-0.32059	6.26438	10.83575	0.004648	0.039476
Usp22	0.320443	7.7381	12.69266	0.00263	0.028713
Agrn	0.319983	9.89124	11.38289	0.003912	0.036022
Zfp609	0.319327	6.747718	10.07678	0.005943	0.045516
Gorasp1	0.319179	5.095284	10.87886	0.004584	0.039206
Apbb3	0.318865	5.109532	9.665776	0.006814	0.049262
Polrmt	0.318836	5.824013	12.25953	0.002992	0.030824
Elk4	-0.3188	5.766069	11.18361	0.004163	0.037354
Pcdh12	0.318702	5.419726	11.12698	0.004238	0.03774
Ubl3	-0.31868	6.101995	13.88309	0.001866	0.023418
B230354K17Rik	-0.31838	4.977344	10.12559	0.005849	0.045093
Trrap	0.318352	7.858587	13.08654	0.002343	0.026753
Cope	0.318277	7.193	12.79391	0.002553	0.028128
Setd1a	0.31827	5.500596	10.72558	0.004814	0.040145
Arpc5	-0.31795	6.831205	15.42364	0.001223	0.018216
Cacybp	-0.31786	6.224215	11.55065	0.003714	0.035001
Kdelr2	0.317852	6.986382	12.06631	0.003172	0.03203
Nop9	0.317851	5.415036	9.633429	0.006888	0.049603
Edc4	0.317768	6.705393	15.67834	0.001143	0.017571
Zc3h4	0.317705	7.118061	9.999941	0.006096	0.04597
Sec24b	0.316906	6.862351	13.12803	0.002315	0.026638

Akt1	0.316875	8.250842	11.2088	0.004131	0.037172
Ppm1g	0.316754	7.134328	11.78382	0.003456	0.033597
Cpt1a	0.316728	7.029486	14.60382	0.001526	0.020966
Sec31a	0.316721	7.796864	14.86618	0.001421	0.02004
Cfl1	0.316661	9.67807	10.39754	0.005351	0.042751
Fxr1	-0.31663	7.030164	13.70368	0.001963	0.024123
Rab5b	0.316555	6.792275	15.74656	0.001122	0.017393
Lbh	0.316497	8.123004	11.85028	0.003387	0.033252
1810058I24Rik	0.316324	5.731718	10.29197	0.005538	0.043679
Dgcr2	0.316225	6.92406	11.02358	0.004379	0.038297
Kif26a	0.316148	5.629805	11.74739	0.003495	0.033766
Rps24	0.316113	9.322688	10.40654	0.005336	0.042694
Ier5l	-0.316	5.619827	9.640199	0.006873	0.049538
Zfp24	-0.31582	6.238988	12.55545	0.002739	0.029521
Ube2o	0.315686	5.928164	13.18759	0.002276	0.026443
Rhobtb1	0.315635	5.475302	12.47609	0.002805	0.029953
Aldh7a1	0.315467	7.358509	14.64575	0.001509	0.020852
Eri1	-0.31528	5.746808	10.85824	0.004614	0.03935
Arhgef17	0.315006	7.9144	11.17858	0.00417	0.037376
Atxn2l	0.314893	7.971075	15.49389	0.0012	0.018091
Pip4k2b	0.314741	6.36873	14.02037	0.001795	0.022943
Uckl1	0.31453	5.473998	11.30501	0.004008	0.036497
Coro1c	0.314402	7.387777	11.38612	0.003908	0.036011
Agps	-0.31424	6.999083	12.27683	0.002977	0.030775
1110038B12Rik	0.314135	5.926552	11.43303	0.003851	0.035712
Ube2b	-0.31401	6.647156	12.36471	0.002899	0.030361
Ipo7	-0.31397	7.432662	11.80554	0.003434	0.033484
Serpinh1	0.313853	10.52547	11.19695	0.004146	0.037255
Rtl5	0.313823	5.29254	10.955	0.004475	0.038704
Dennd2a	0.313787	6.264679	11.77295	0.003468	0.033598
Pygo2	0.313667	6.480865	11.76114	0.003481	0.033664
Rnasel	-0.31349	5.070254	9.61509	0.006931	0.049789
Tpcn1	0.313066	7.152064	14.59107	0.001532	0.021004
Gnb1	0.312987	8.852441	13.74703	0.001939	0.023969
Kcp	0.312745	6.207802	13.50465	0.002077	0.025036
Bmpr1a	-0.31271	7.183436	11.08786	0.004291	0.038016
Sec16a	0.312532	7.172193	12.32104	0.002937	0.030598
Plcg1	0.312403	7.550208	14.4524	0.001591	0.021408
Apcdd1	-0.31233	7.174764	9.796932	0.006521	0.048006
Ptk7	0.312253	8.41118	10.49246	0.005189	0.042047
Clcn3	-0.31222	6.291809	10.03972	0.006016	0.045684
BC031181	-0.31217	5.919213	12.43285	0.002841	0.030196
Crot	-0.31214	6.076838	12.48963	0.002793	0.029886
Mical1	0.311982	5.28072	10.75727	0.004765	0.039936
Elac2	0.311294	5.584275	10.77428	0.00474	0.039862
C1qtnf6	0.311286	5.837372	11.33705	0.003968	0.036283
Hdac7	0.310909	7.22721	14.22616	0.001695	0.02227

Bmp7	0.310816	6.946268	11.00697	0.004402	0.038395
Sap130	0.310675	6.237219	12.86076	0.002503	0.027772
Txnl1	-0.31066	6.737009	12.44447	0.002831	0.030131
Gm4204	-0.31044	6.879361	13.99004	0.00181	0.023072
Chaf1a	0.31036	5.995157	10.4049	0.005338	0.042694
Trabd2b	0.310212	6.133529	13.91816	0.001847	0.023409
Rae1	0.310206	6.308161	13.80823	0.001905	0.023691
Lrrc14	0.310135	5.26115	9.796114	0.006523	0.048006
Tbc1d13	0.310134	6.494905	13.82811	0.001895	0.023606
Zfp395	0.309937	6.979443	10.131	0.005838	0.045085
Cs	0.309728	8.230001	13.40407	0.002138	0.025514
Clcn6	0.309645	5.420259	10.39099	0.005363	0.042788
Homer3	0.309501	6.144123	11.90803	0.003328	0.032868
Rpn2	0.309487	8.245869	12.56148	0.002734	0.029486
Pcnx3	0.309349	7.224864	12.84515	0.002515	0.027857
Hnrnph2	-0.30903	6.895674	10.01746	0.006061	0.045842
Bak1	0.309028	5.820329	11.49202	0.003782	0.035359
Bcr	0.308916	6.056138	10.69868	0.004856	0.040395
Tmod3	-0.3088	7.260242	9.601561	0.006963	0.049935
Plcb3	0.308746	7.009298	14.64928	0.001508	0.020852
Myo5a	-0.30866	6.159458	12.28202	0.002972	0.03077
Mindy2	-0.30856	5.673714	10.00118	0.006093	0.04597
Cacna1g	0.308334	6.849026	13.86041	0.001878	0.023461
Cstf1	0.30816	6.006117	13.30388	0.002201	0.025957
Smap2	0.307646	5.769975	10.27958	0.005561	0.043778
Thbs1	0.307559	7.860849	13.04857	0.00237	0.026792
Ppp6r1	0.307412	7.309448	13.18213	0.002279	0.026447
Psmd4	0.306967	7.033517	13.06662	0.002357	0.026766
Atp6v1d	-0.30671	6.324425	11.15758	0.004198	0.037568
Mbtd1	-0.30649	6.838721	10.72707	0.004812	0.040145
Tnpo2	0.306398	6.960448	14.70628	0.001484	0.020647
Slc7a8	0.306199	6.647544	12.43785	0.002837	0.030169
Tpd52	-0.30619	6.592534	11.70841	0.003537	0.034009
Sulf2	0.306117	7.334727	10.95672	0.004472	0.038704
Mknk2	0.306015	7.547443	11.42271	0.003864	0.035758
Clcn2	0.305872	5.687264	9.855172	0.006396	0.047484
Asap2	0.305639	5.765729	10.16757	0.005768	0.044816
Gga1	0.305611	6.595713	10.56318	0.005072	0.041652
Htatsf1	-0.30536	6.317901	12.38163	0.002885	0.030284
Larp1	0.304955	7.413262	12.07475	0.003164	0.031994
Aars	0.304654	7.050977	10.52315	0.005138	0.041938
Kdm6b	0.304563	7.100062	10.66745	0.004905	0.040603
Nrf1	0.303962	5.973516	11.50988	0.003761	0.035285
Adgra2	0.303833	7.750952	10.68189	0.004882	0.04054
Arhgef7	0.303701	7.115818	11.83433	0.003403	0.033355
Nid1	0.303627	8.822396	12.33267	0.002927	0.030527
Eif3d	0.303599	7.325091	10.67923	0.004886	0.040555

Prpf31	0.30358	6.251083	12.07639	0.003162	0.031994
Fam43a	0.303218	5.606767	11.00679	0.004402	0.038395
Dcaf15	0.302877	5.89033	10.41407	0.005323	0.04268
Aebp1	0.302573	6.672368	11.65671	0.003594	0.034337
Epas1	0.302382	6.969695	12.89352	0.002479	0.027593
Csrp1	0.30234	8.138355	10.26736	0.005583	0.043914
Sparc	0.30224	10.07985	10.14541	0.005811	0.044968
Spout1	0.302149	5.756694	12.04647	0.003191	0.032133
Tkt	0.30213	8.590793	9.689897	0.006759	0.04905
Zbtb46	0.301755	5.454073	10.82862	0.004658	0.039521
Ppme1	0.301692	6.06363	12.15511	0.003088	0.031515
Pld3	0.301689	6.806222	10.2865	0.005548	0.043699
Flii	0.301545	7.112195	13.03814	0.002377	0.026856
Gm10052	0.301528	7.801373	14.06026	0.001775	0.022886
Tcf3	0.301442	7.967173	11.12019	0.004247	0.037782
Phax	-0.30129	5.513264	10.54885	0.005095	0.041765
Ptbp1	0.301283	9.39159	10.12786	0.005844	0.045088
Adamts10	0.301196	7.234581	13.70366	0.001963	0.024123
Piezo1	0.301166	6.970922	12.28083	0.002973	0.03077
Snrpb	0.301035	7.933095	10.58997	0.005028	0.041387
Ppp6c	-0.30094	6.007841	10.7917	0.004713	0.039754
Maml1	0.300303	6.169102	10.54031	0.005109	0.041841
Ppp4r1	0.300218	6.626543	12.7844	0.00256	0.028164
Raf1	0.300005	6.397516	11.3141	0.003997	0.036468
Cdk2ap1	0.29985	5.12996	9.813396	0.006486	0.047889
Gm2a	-0.2998	6.028905	10.04163	0.006013	0.045684
Gatad2a	0.299355	6.880721	10.97324	0.004449	0.038619
Add1	0.299277	8.30793	11.78624	0.003454	0.033597
Wasf2	0.298954	7.6293	10.77769	0.004735	0.039838
Tmem131l	0.298478	5.856184	10.33425	0.005463	0.043273
Rnf216	0.298092	6.376294	10.9834	0.004435	0.038572
Nudt4	-0.29799	8.862793	10.9124	0.004536	0.039078
Pcgf2	0.29768	6.157334	12.27259	0.00298	0.030775
Fndc4	0.297353	5.186978	9.665859	0.006814	0.049262
Miip	0.296345	5.606764	11.30707	0.004005	0.036497
Usp5	0.296307	7.271599	13.29089	0.002209	0.026028
Ddah2	0.296054	7.648355	9.950131	0.006197	0.046438
Zswim8	0.295895	7.496475	12.11636	0.003124	0.031721
Stk39	-0.29564	5.451252	10.18906	0.005728	0.044638
Chst15	0.295629	5.8177	11.66676	0.003583	0.034299
Arhgap28	0.295537	5.80375	9.611434	0.00694	0.04983
Inpp5e	0.295217	5.666687	10.52629	0.005133	0.041934
Snrpa	0.294929	7.206539	11.60628	0.00365	0.034663
Snx7	-0.29479	5.673189	10.15385	0.005795	0.04492
Dnajc10	-0.2942	7.064375	10.18322	0.005739	0.044674
Fgd5	0.293978	6.314944	12.15799	0.003085	0.031506
Dcps	0.293933	5.593056	10.8196	0.004672	0.039552

Eng	0.293771	7.446046	10.01565	0.006064	0.045842
Rad23b	0.293744	7.900188	12.01788	0.003218	0.032314
Ahdc1	0.293706	6.429034	9.636183	0.006882	0.049578
Atxn2	0.293553	6.679796	10.57272	0.005056	0.04156
Dennd1a	0.293514	5.706564	9.771905	0.006576	0.048235
Gmeb2	0.29343	5.42467	10.33691	0.005458	0.043273
Rbms2	0.293121	6.679339	11.84762	0.00339	0.033257
Tmem229a	-0.293	6.303853	10.85086	0.004625	0.039376
Eif4enif1	0.292829	6.431922	11.12328	0.004243	0.037764
Supt5	0.292651	7.419444	12.24653	0.003004	0.030837
Shank3	0.292494	6.396554	10.33471	0.005462	0.043273
Nkiras2	0.292368	5.850454	11.39121	0.003902	0.035991
Pla2g4a	-0.29232	5.804198	10.62345	0.004974	0.041077
C1s1	0.292029	5.92042	9.739359	0.006648	0.048522
Mlxip	0.291949	6.546538	10.42307	0.005307	0.042613
Cdk16	0.291825	7.564361	12.39936	0.002869	0.030253
Flot2	0.291442	6.080799	11.40556	0.003884	0.035887
Arhgap31	0.291176	6.081048	10.20349	0.005701	0.044519
Asna1	0.291159	6.432739	10.14538	0.005811	0.044968
As3mt	-0.29081	5.610588	9.853156	0.0064	0.047496
Gsn	0.290573	7.355256	9.626784	0.006904	0.049634
Tsc2	0.290534	7.127372	11.69557	0.003551	0.03407
Tmem161a	0.290325	5.848128	11.48787	0.003786	0.035359
Gramd4	0.289763	5.979183	10.61059	0.004995	0.04119
Plekhg5	0.289746	6.240061	11.49641	0.003776	0.035349
Eps15l1	0.289728	6.665051	12.57945	0.00272	0.029419
Gm7964	0.289656	7.458885	11.49109	0.003783	0.035359
Eif4a1	0.289468	7.986017	11.23593	0.004096	0.037048
Drg2	0.28926	6.270376	11.78216	0.003458	0.033597
Prr3	0.289237	5.34447	10.10915	0.00588	0.045229
Trappc12	0.289	5.930433	11.36685	0.003931	0.036077
Gps2	0.288552	5.680443	10.03891	0.006018	0.045684
Commd3	-0.28806	6.041625	11.77362	0.003467	0.033598
Arap1	0.287993	7.336757	9.690561	0.006757	0.04905
Ganab	0.287977	8.450298	11.55936	0.003704	0.034963
Pxdn	0.287886	8.652397	10.79464	0.004709	0.039736
Sbf1	0.287408	7.011508	11.9462	0.003289	0.032599
Ncln	0.287149	7.028029	10.50975	0.00516	0.041985
Fbxw8	0.287	6.956677	11.04966	0.004343	0.038255
Map3k3	0.286924	6.4593	11.82357	0.003415	0.03341
Atf4	0.286803	7.442864	10.08393	0.005929	0.045441
Tsc22d2	-0.28673	6.280233	10.41814	0.005316	0.042643
Pex5	0.286488	6.341161	9.748219	0.006628	0.048439
Mfsd14b	-0.28631	6.146424	9.757093	0.006609	0.048375
Cant1	0.286304	6.347682	10.19089	0.005725	0.044631
Tufm	0.285973	6.400309	11.73642	0.003507	0.03381
Sema3f	0.285718	7.104249	11.38178	0.003913	0.036022

Plp2	0.285548	6.396623	9.88814	0.006326	0.047105
Gm13509	0.285287	6.23825	10.87496	0.00459	0.039217
Arf3	0.285236	7.794676	10.82676	0.004661	0.039521
Acvrl1	0.285212	5.819798	10.50548	0.005167	0.042012
Tfip11	0.284583	6.18221	9.723811	0.006683	0.048675
Sf3b3	0.284551	8.109836	9.80022	0.006514	0.048001
Rxrb	0.284473	6.409261	9.898266	0.006305	0.047025
Pard3	0.28438	6.609072	10.21335	0.005683	0.04444
Nploc4	0.284359	6.40192	10.29909	0.005526	0.043597
Oaz1	0.283836	7.789998	10.14347	0.005814	0.044975
Anxa6	0.28365	8.00013	10.98568	0.004431	0.038569
Zcchc24	0.28354	6.103966	10.20122	0.005705	0.044519
Nfic	0.28344	6.895394	10.23281	0.005646	0.044268
Rasa3	0.283412	5.670253	10.63105	0.004962	0.040996
Pkd1	0.283346	7.256564	10.21168	0.005686	0.044445
Pes1	0.283095	6.827382	9.805244	0.006503	0.04796
Ambra1	0.28275	6.192109	9.775875	0.006567	0.048231
Gart	0.282724	6.572371	10.3261	0.005477	0.043324
Aktip	-0.28253	6.294994	11.21554	0.004122	0.037146
Cbx1	-0.28234	7.535639	11.24287	0.004087	0.037004
Tut4	-0.28227	6.592482	9.913015	0.006274	0.046835
Pear1	0.282187	6.902538	10.24899	0.005617	0.0441
Myo9b	0.282185	6.804273	11.86161	0.003375	0.033189
Slc48a1	0.281896	6.611073	11.58432	0.003675	0.034805
Tm9sf3	-0.28177	8.286107	10.24732	0.00562	0.044105
Eif2a	-0.28155	6.265985	9.629732	0.006897	0.049605
Ergic2	-0.28129	6.070836	10.92938	0.004511	0.03892
Abi1	-0.28106	6.331812	10.21733	0.005675	0.044422
Npm3	0.280957	5.995265	10.0395	0.006017	0.045684
Caskin2	0.280794	6.727457	10.05742	0.005981	0.045684
Fastk	0.280781	6.841001	10.33563	0.00546	0.043273
Tspyl2	0.279888	5.560287	9.653391	0.006842	0.049412
Cxcl12	0.279875	8.580501	10.03847	0.006019	0.045684
Arhgap27	0.279811	6.027084	9.83197	0.006445	0.047736
Kank2	0.27923	6.800851	10.37657	0.005388	0.042911
Ap3d1	0.278259	7.662399	11.87376	0.003363	0.033132
Cd276	0.278021	5.886446	10.75819	0.004764	0.039936
Ccnd3	0.277329	7.746418	9.805656	0.006502	0.04796
Nfatc4	0.27724	6.565116	11.78197	0.003458	0.033597
Pias3	0.276948	5.888785	10.23059	0.005651	0.044268
Klhdc3	0.276592	7.165765	9.725331	0.006679	0.048671
Nectin3	-0.27623	6.37494	10.08309	0.005931	0.045441
Hnf4a	0.276097	7.771915	11.03636	0.004361	0.038255
Amotl1	0.27578	7.438518	9.605895	0.006952	0.049903
Sec13	0.274853	6.785324	10.35528	0.005425	0.043093
Dhx30	0.274685	7.041457	11.23142	0.004101	0.037057
Ets2	0.27448	7.505303	10.14013	0.005821	0.045004

Itga3	0.274404	6.968714	9.785395	0.006546	0.048158
Sucla2	-0.27439	6.749186	11.03783	0.004359	0.038255
Mfn2	0.27428	6.505735	9.745377	0.006635	0.048465
Foxo1	0.274185	5.849701	10.35722	0.005422	0.043086
Snrpf	0.274132	6.242916	11.22015	0.004116	0.037135
Rab21	-0.27407	6.342865	10.10425	0.00589	0.045229
Ssrp1	0.27391	8.151187	9.740431	0.006646	0.048522
Dpf2	0.273566	7.083427	11.5734	0.003687	0.034886
Tubb4b	0.273361	8.401497	9.707361	0.00672	0.048864
Nsa2	-0.27306	6.556727	9.719783	0.006692	0.048715
Ubxn4	-0.27287	6.893002	10.67379	0.004895	0.040569
Tnk2	0.272443	6.507322	10.26329	0.00559	0.043953
Cab39	-0.2723	7.032452	10.88557	0.004574	0.039177
Git2	0.272147	6.270557	10.43967	0.005279	0.042407
Arhgef25	0.271605	6.913123	11.81009	0.003429	0.033455
Hdac6	0.271504	7.160148	10.98656	0.00443	0.038569
Midn	0.268667	7.779026	10.56011	0.005077	0.041652
Ahsa1	0.267824	6.991168	11.10881	0.004262	0.037879
Elmo2	0.266933	6.706936	10.40359	0.005341	0.042694
Nfkb1	0.265599	6.522923	10.49582	0.005183	0.042039
Atp6ap1	0.265571	7.459527	11.18205	0.004165	0.037354
Sdhc	0.265524	7.565241	9.755183	0.006613	0.048386
Shc1	0.264983	7.338788	10.44491	0.00527	0.042407
Slc6a8	0.263026	6.566207	9.660377	0.006826	0.049317
Cd81	0.262071	8.401477	10.08684	0.005924	0.045424
Tle3	0.261878	7.361177	9.98319	0.00613	0.046127
Gak	0.257957	6.927581	10.10897	0.005881	0.045229
Csk	0.25792	6.786921	9.781337	0.006555	0.048183
Inppl1	0.256863	7.47772	9.782611	0.006552	0.048183
Lamb2	0.256552	7.849122	10.59114	0.005026	0.041387
Cap1	0.255896	7.801036	9.933344	0.006232	0.046599
