# Supplementary material for: Disrupted glucocorticoid receptor cell signalling causes a ciliogenesis defect in the fetal mouse renal tubule
Source: EMBO Rep. 2025 Apr 17;26(11):2883–909. doi: 10.1038/s44319-025-00454-0 (PMC12152183; doi:10.1038/s44319-025-00454-0)
Supplement: Supplementary file 2 — Appendix [file 44319_2025_454_MOESM2_ESM.pdf]

Disrupted glucocorticoid receptor cell signalling causes a ciliogenesis defect in the fetal mouse renal tubule

Kelly L. Short[1#], Jianshen Lao[1#], Rachel Lam[2], Julie L.M. Moreau[2], Judy Ng[1], Mehran Piran[2], Alexander N. Combes[2], Denny L. Cottle[2] and Timothy J. Cole[1*]

Appendix

Table of Contents

Appendix Figure S1: Glucocorticoid localisation in fetal mouse kidney proximal tubule and collecting duct cells at E14.5, E16.5 and E18.5 3

Appendix Figure S2: Glucocorticoid localisation in fetal mouse kidney podocytes and stroma cells at E14.5, E16.5 and E18.5 5

Appendix Figure S3: PAS staining of E18.5 fetal kidneys from conditional GR-null mice 7

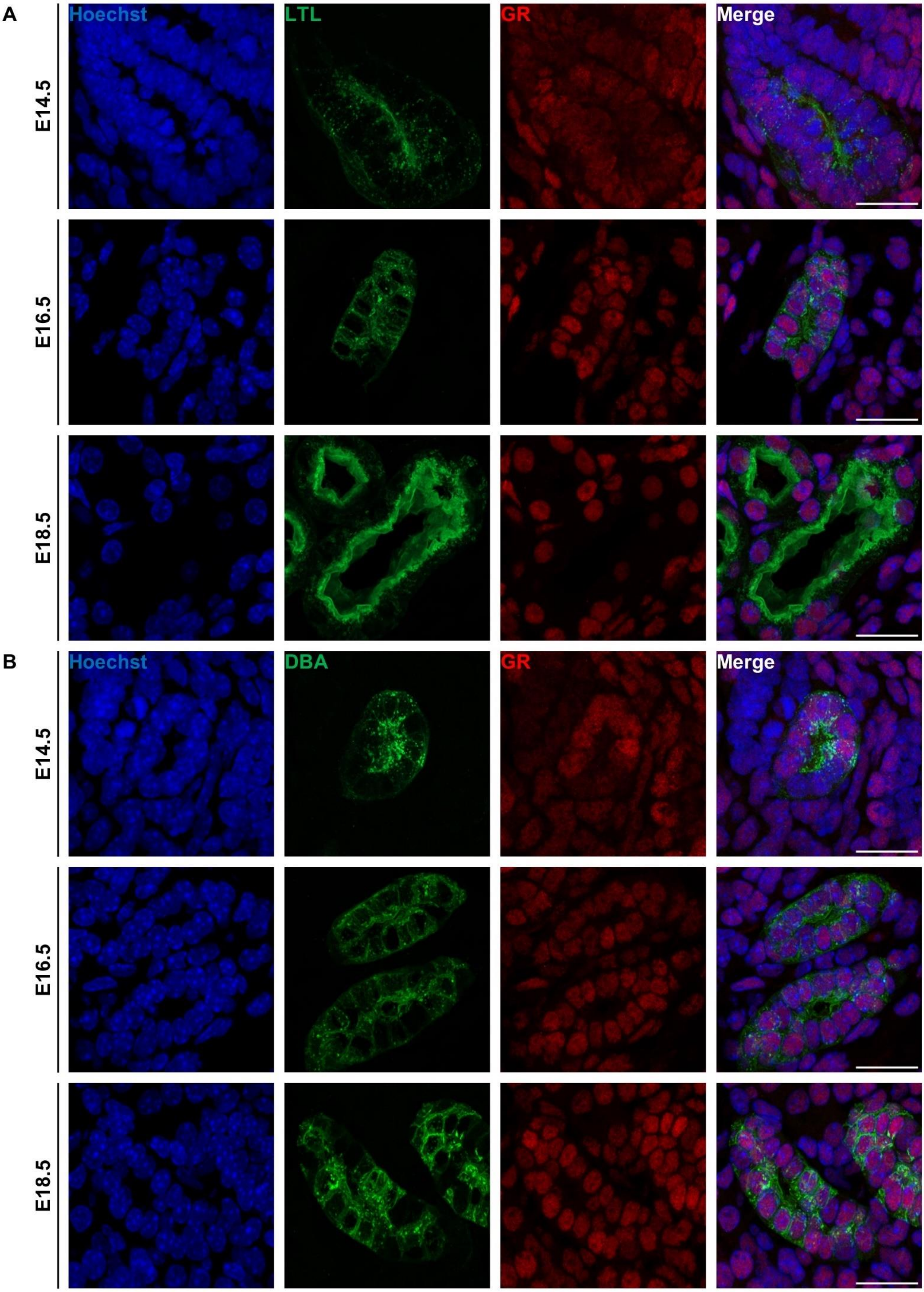
A
Hoechst
LTL
GR
Merge
E14.5
E16.5
E18.5
B
Hoechst
DBA
GR
Merge
E14.5
E16.5
E18.5

Appendix Figure S1: Glucocorticoid localisation in fetal mouse kidney proximal tubule and collecting duct cells at E14.5, E16.5 and E18.5

A. Immunofluorescence of glucocorticoid receptor (GR) in fetal kidney proximal tubule cells at E14.5, E16.5 and E18.5. Sections were stained with Hoechst (blue, nucleus), Lotus Tetragonolobus Lectin (LTL) (green, proximal tubule) and GR (red, GR). Slides were imaged with a Zeiss LSM 980 confocal microscope (63x objective, 2x digital zoom). Scale bar represents 20 µm. All images are representative of E14.5 (n=3), E16.5 (n=4), E18.5 (n=4) animals per age group.

B. Immunofluorescence of glucocorticoid receptor (GR) in fetal kidney collecting duct cells at E14.5, E16.5 and E18.5. Sections were stained with Hoechst (blue, nucleus), Dolichos Biflorus Agglutinin (DBA) (green, collecting duct) and GR (red, GR). Slides were imaged with a Zeiss LSM 980 confocal microscope (63x objective, 2x digital zoom). Scale bar represents 20 µm. All images are representative of E14.5 (n=3), E16.5 (n=4), E18.5 (n=4) animals per age group.

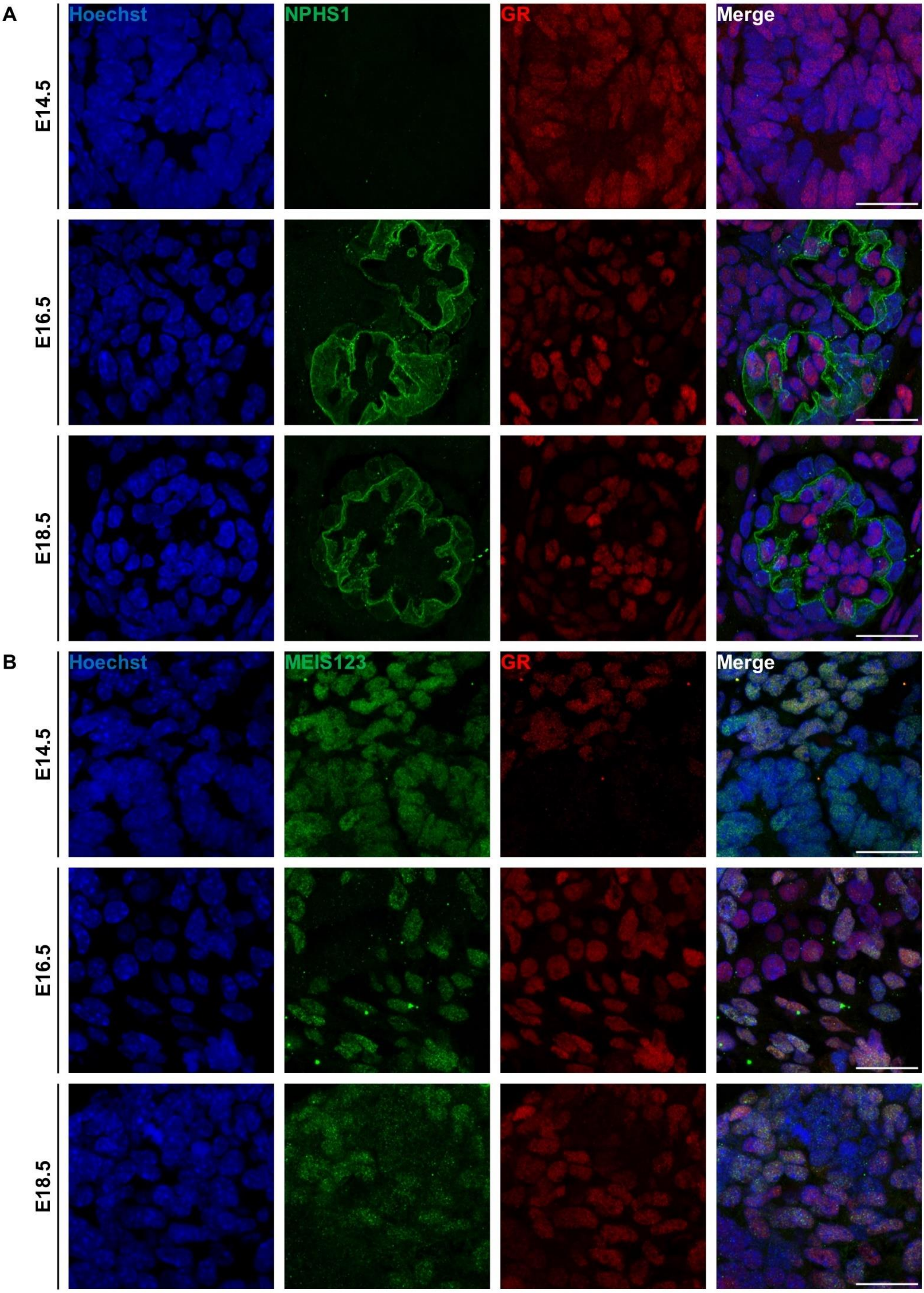
A
Hoechst
NPHS1
GR
Merge
E14.5
E16.5
E18.5
B
Hoechst
MEIS123
GR
Merge
E14.5
E16.5
E18.5

Appendix Figure S2: Glucocorticoid localisation in fetal mouse kidney podocytes and stroma cells at E14.5, E16.5 and E18.5

A. Immunofluorescence of glucocorticoid receptor (GR) in fetal kidney podocyte cells at E14.5, E16.5 and E18.5. Sections were stained with Hoechst (blue, nucleus), nephrin (NPHS1) (green, podocyte) and GR (red, GR). Slides were imaged with a Zeiss LSM 980 confocal microscope (63x objective, 2x digital zoom). Scale bar represents 20 µm. All images are representative of E14.5 (n=3), E16.5 (n=4), E18.5 (n=4) animals per age group.

B. Immunofluorescence of glucocorticoid receptor (GR) in fetal kidney stroma cells at E14.5, E16.5 and E18.5. Sections were stained with Hoechst (blue, nucleus), MEIS123 (green, stroma) and GR (red, GR). Slides were imaged with a Zeiss LSM 980 confocal microscope (63x objective, 2x digital zoom). Scale bar represents 20 µm. All images are representative of E14.5 (n=3), E16.5 (n=4), E18.5 (n=4) animals per age group.

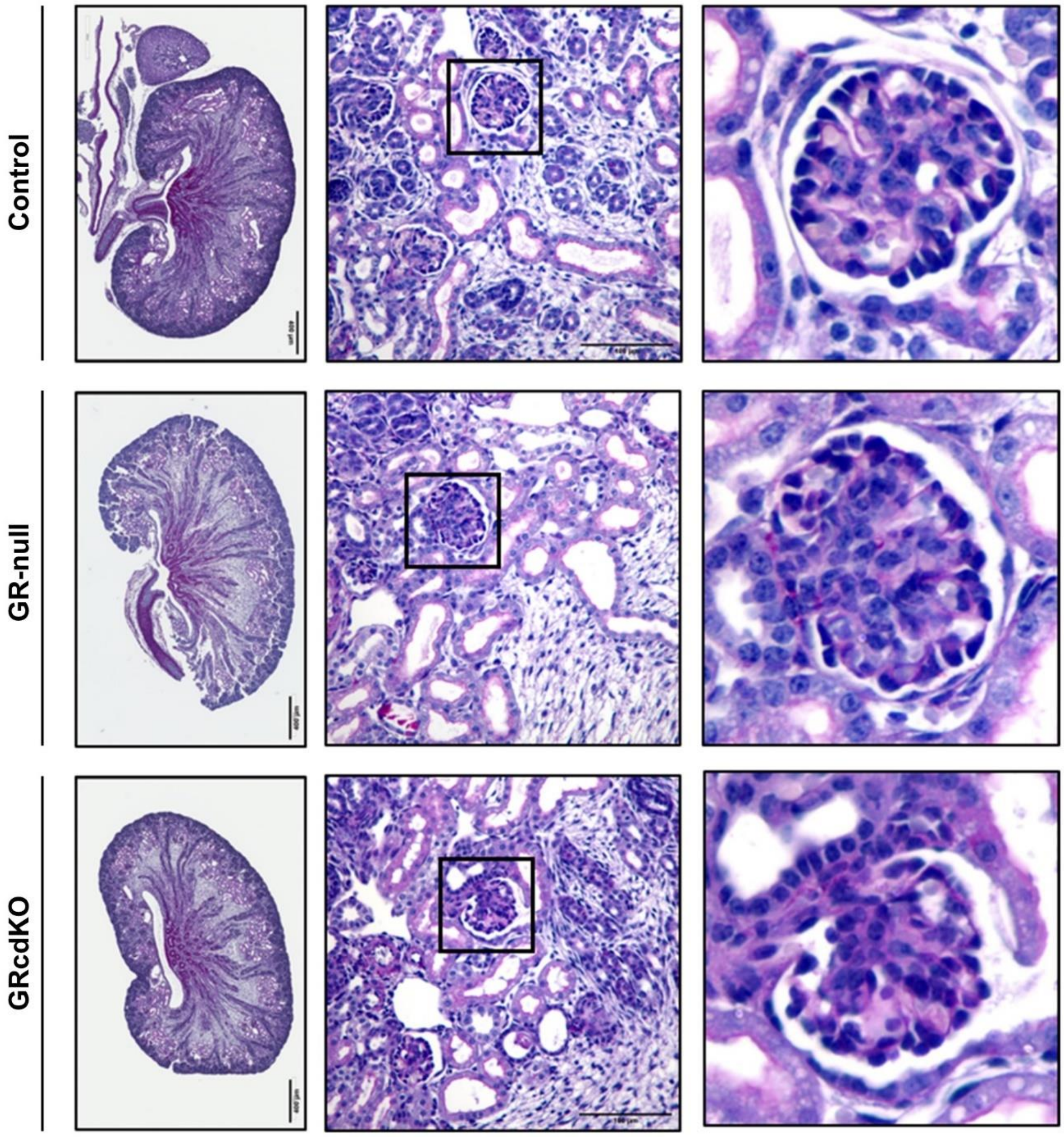
Control
GR-null
GRcdKO

Appendix Figure S3: PAS staining of E18.5 fetal kidneys from conditional GR-null mice

Histological section of E18.5 fetal mouse kidneys were PAS stained for control, GR-null and HoxB7Cre-GR flox (GRcdKO) mice. Images are shown 10x, 20x and 100x magnification, scale bar represents 400 μm.
