# Supplementary figures and images for: Disrupted glucocorticoid receptor cell signalling causes a ciliogenesis defect in the fetal mouse renal tubule
Source: EMBO Rep. 2025 Apr 17;26(11):2883–909. doi: 10.1038/s44319-025-00454-0 (PMC12152183; doi:10.1038/s44319-025-00454-0)

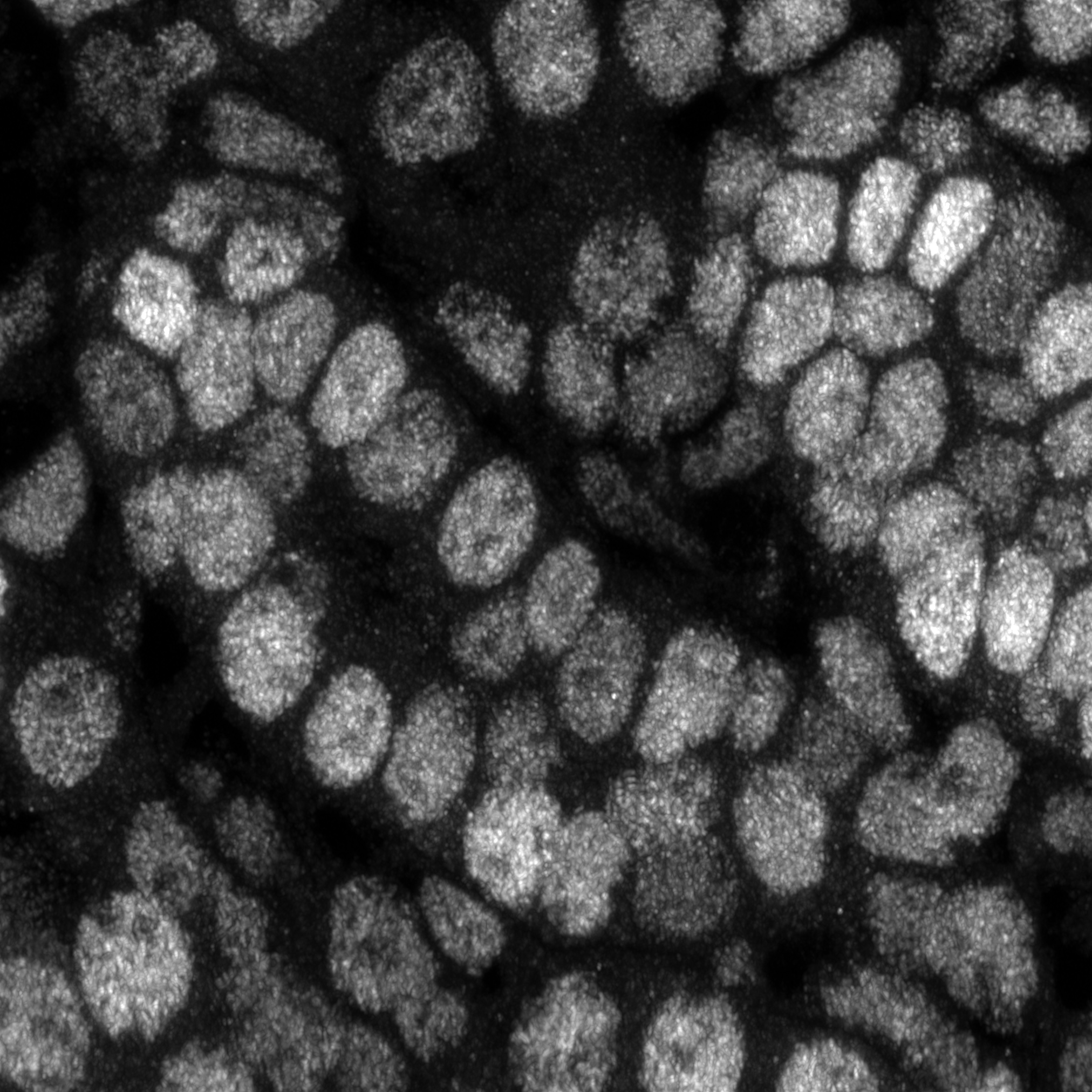

Supplement: Supplementary file 4 — Source data Fig. 1 [file 44319_2025_454_MOESM4_ESM.zip › Source Data - Figure 1/1H/Hoechst, DBA, GR E18.5.tif]

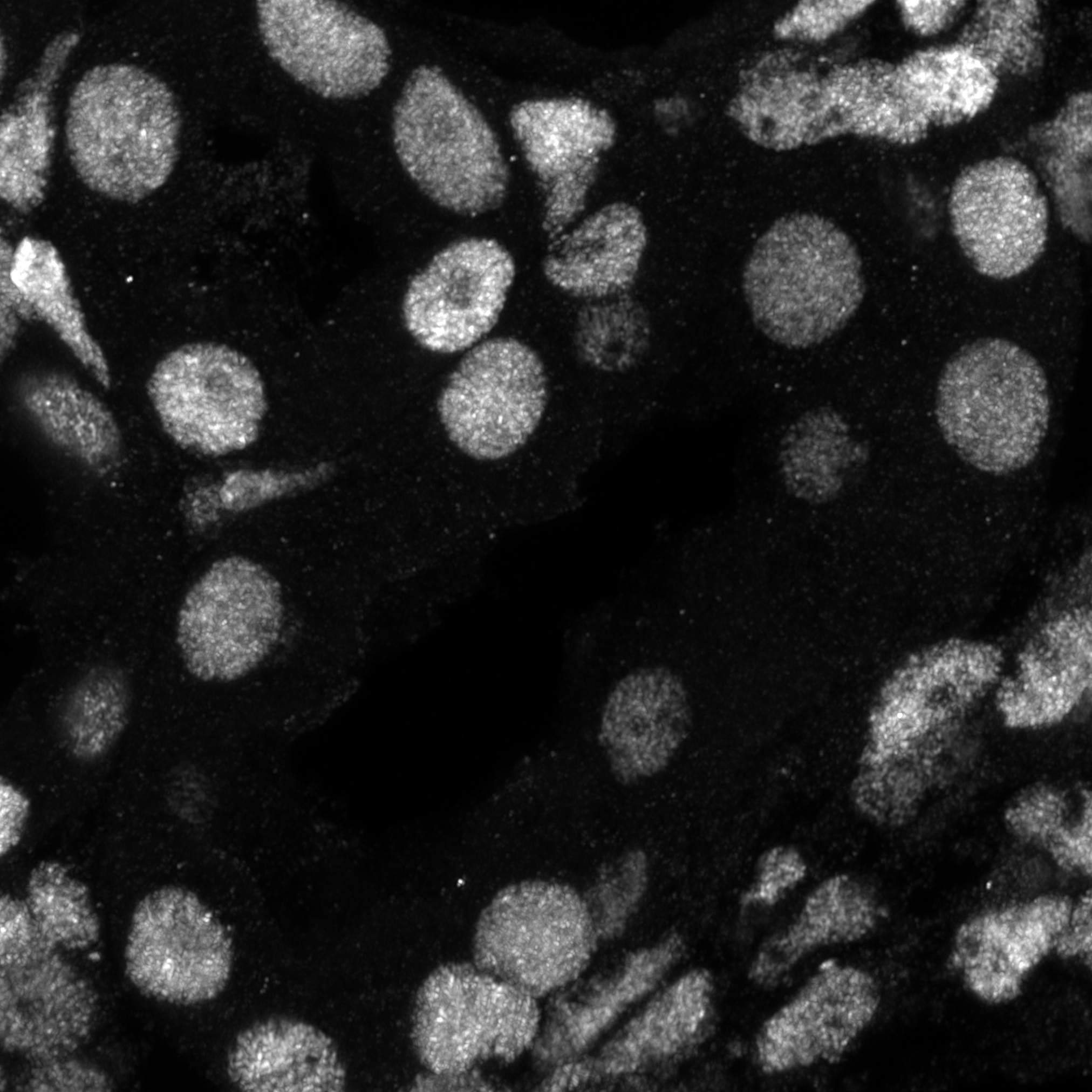

Supplement: Supplementary file 4 — Source data Fig. 1 [file 44319_2025_454_MOESM4_ESM.zip › Source Data - Figure 1/1H/Hoechst, LTL, GR E18.5.tif]

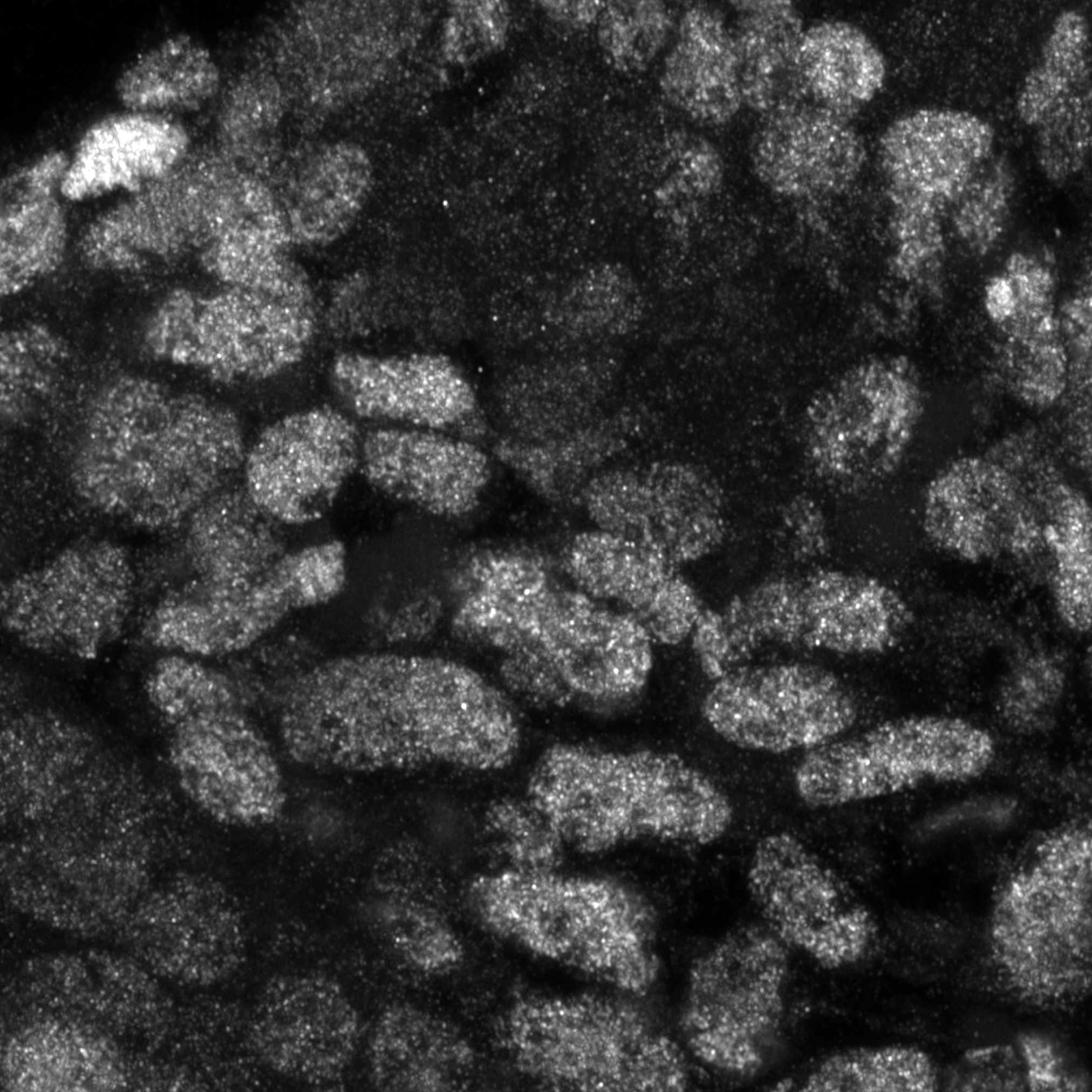

Supplement: Supplementary file 4 — Source data Fig. 1 [file 44319_2025_454_MOESM4_ESM.zip › Source Data - Figure 1/1H/Hoechst, MEIS123, GR E18.5.tif]

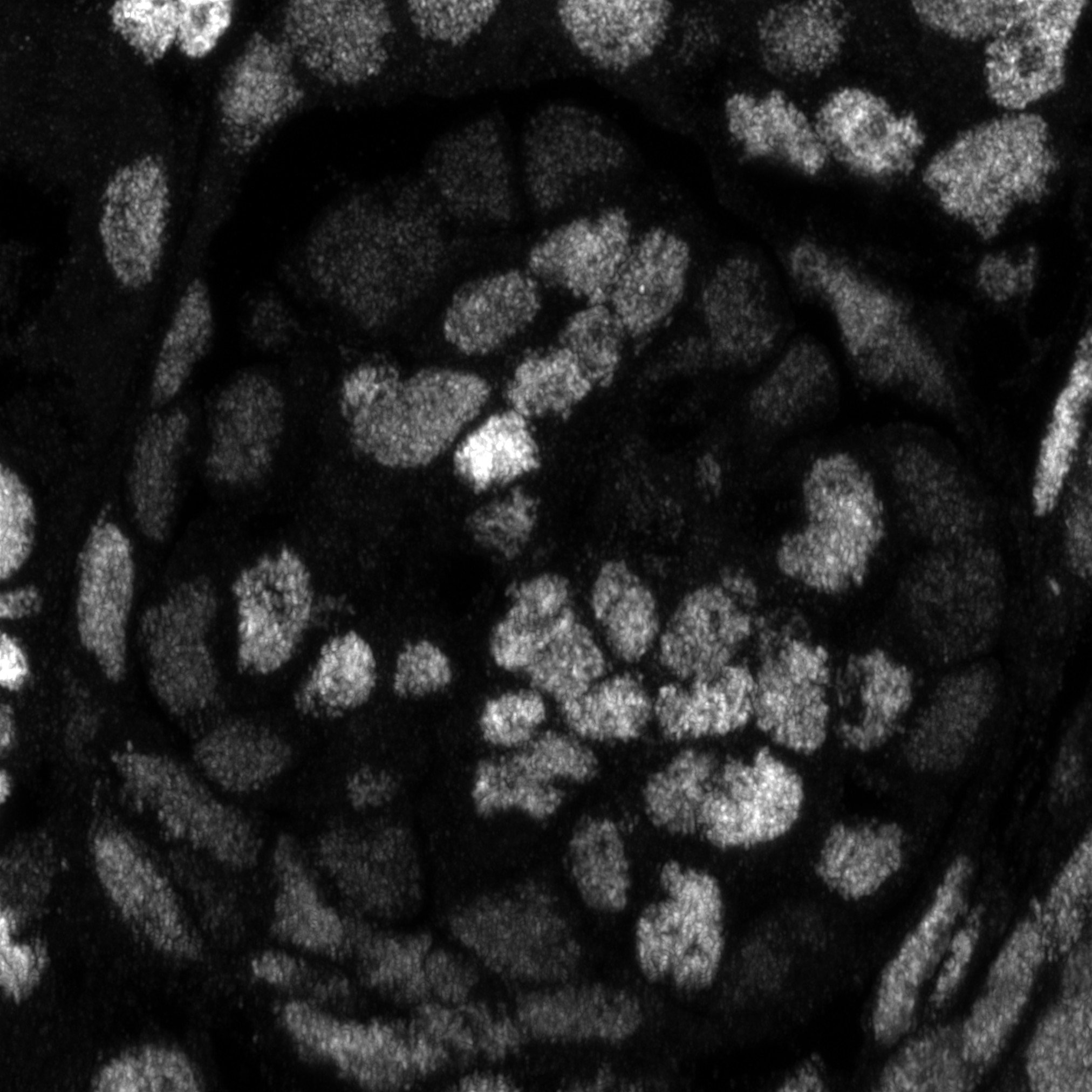

Supplement: Supplementary file 4 — Source data Fig. 1 [file 44319_2025_454_MOESM4_ESM.zip › Source Data - Figure 1/1H/Hoechst, NPHS1, GR E18.5.tif]

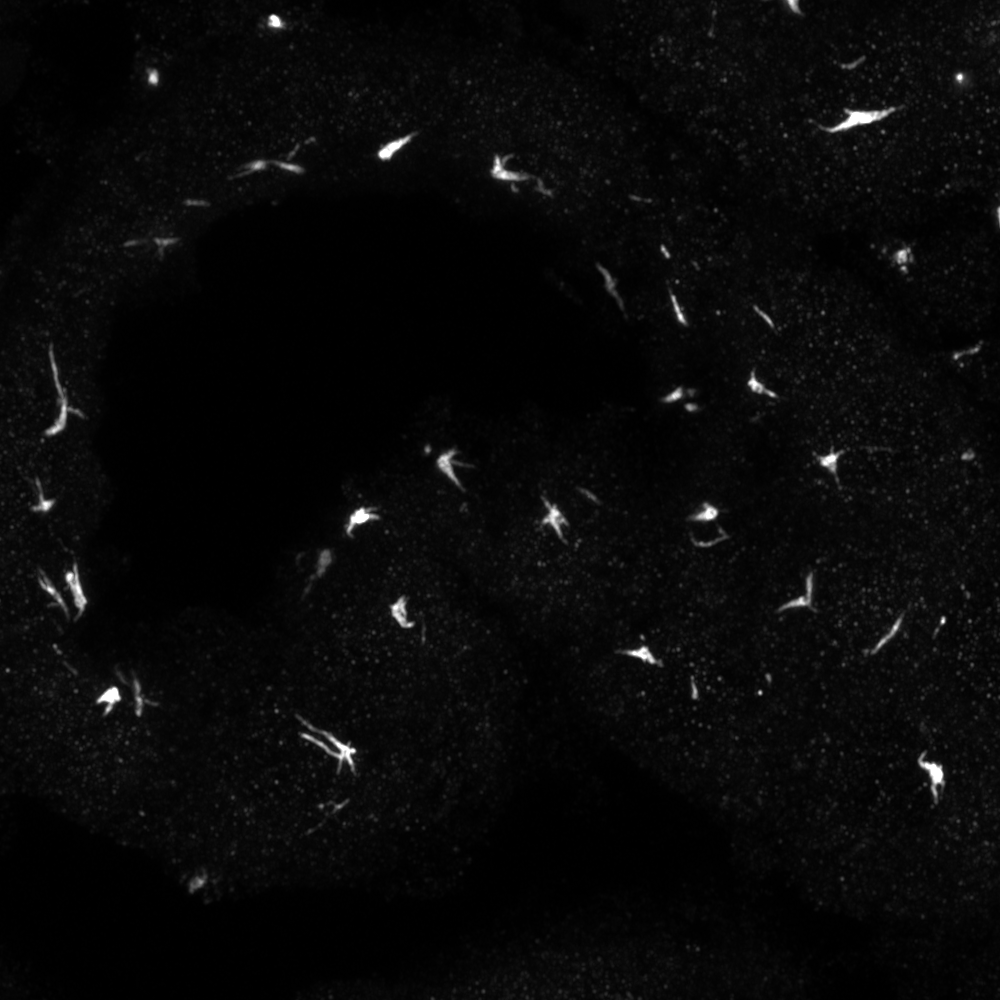

Supplement: Supplementary file 6 — Source data Fig. 3 [file 44319_2025_454_MOESM6_ESM.zip › Source Data - Figure 3/3A/Control hoechst, AceTub, PCNT, LTL E18.5.tif]

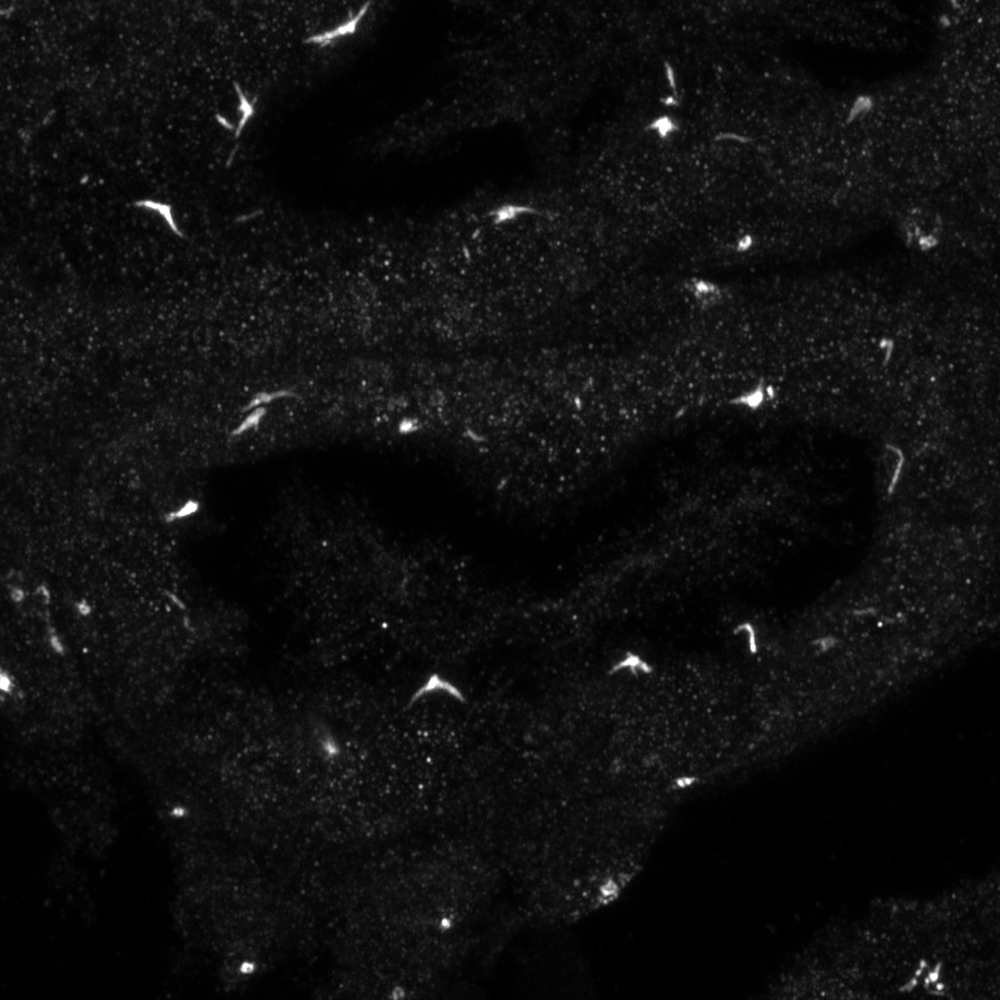

Supplement: Supplementary file 6 — Source data Fig. 3 [file 44319_2025_454_MOESM6_ESM.zip › Source Data - Figure 3/3A/GR-null hoechst, AceTub, PCNT, LTL E18.5.tif]

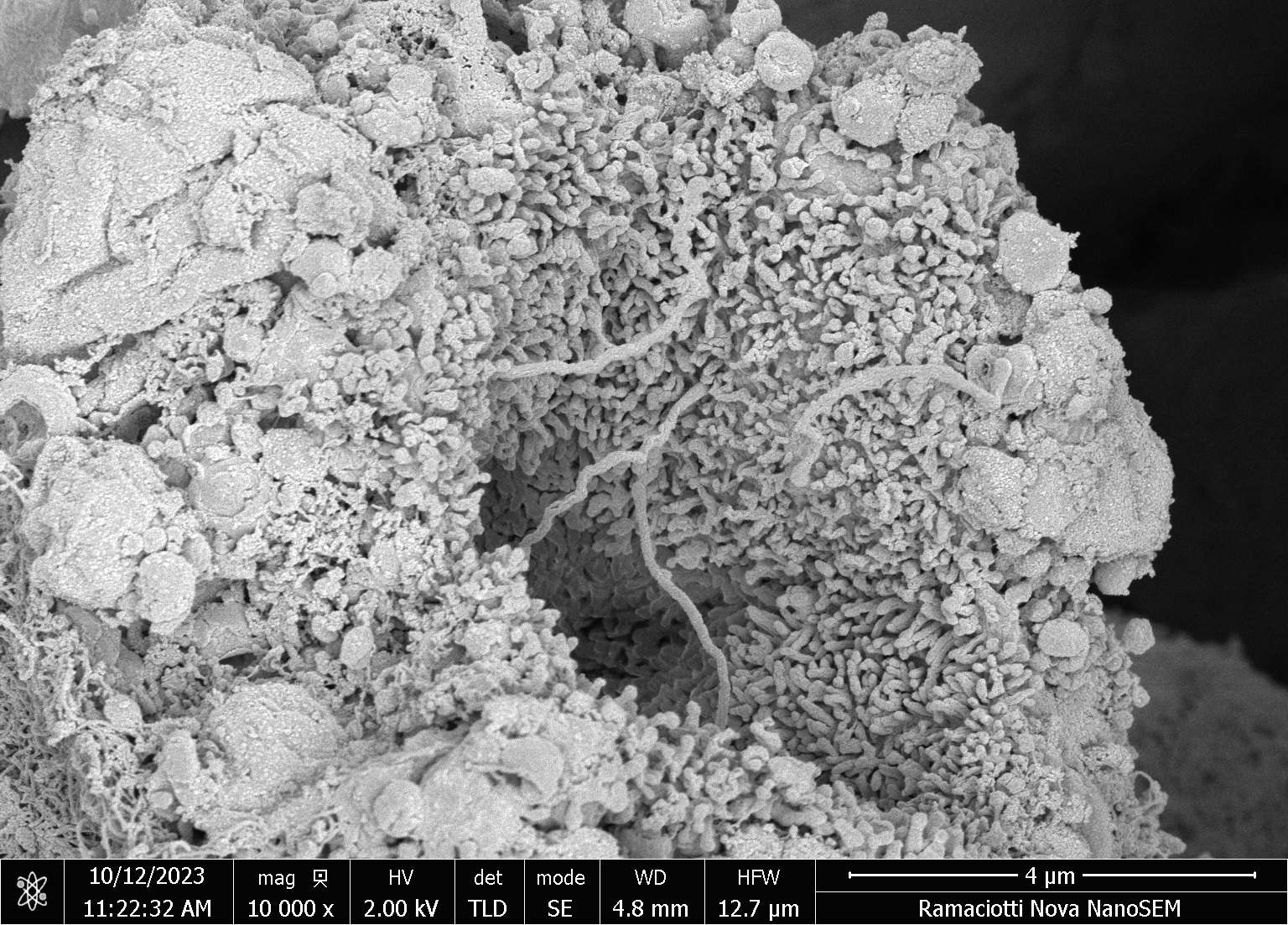

Supplement: Supplementary file 6 — Source data Fig. 3 [file 44319_2025_454_MOESM6_ESM.zip › Source Data - Figure 3/3E/Control electron microscopy.tif]

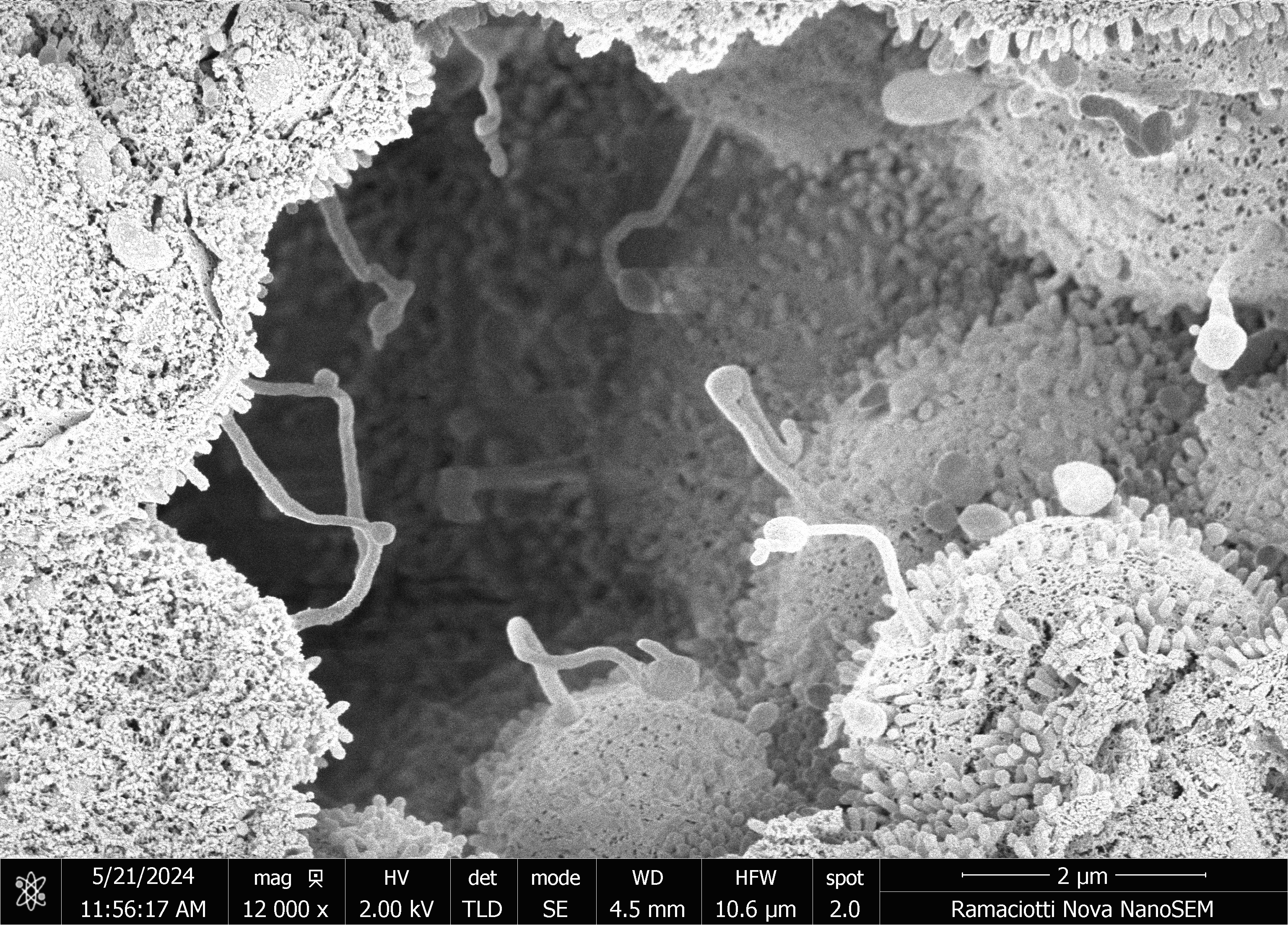

Supplement: Supplementary file 6 — Source data Fig. 3 [file 44319_2025_454_MOESM6_ESM.zip › Source Data - Figure 3/3E/GR-null electron microscopy.tif]

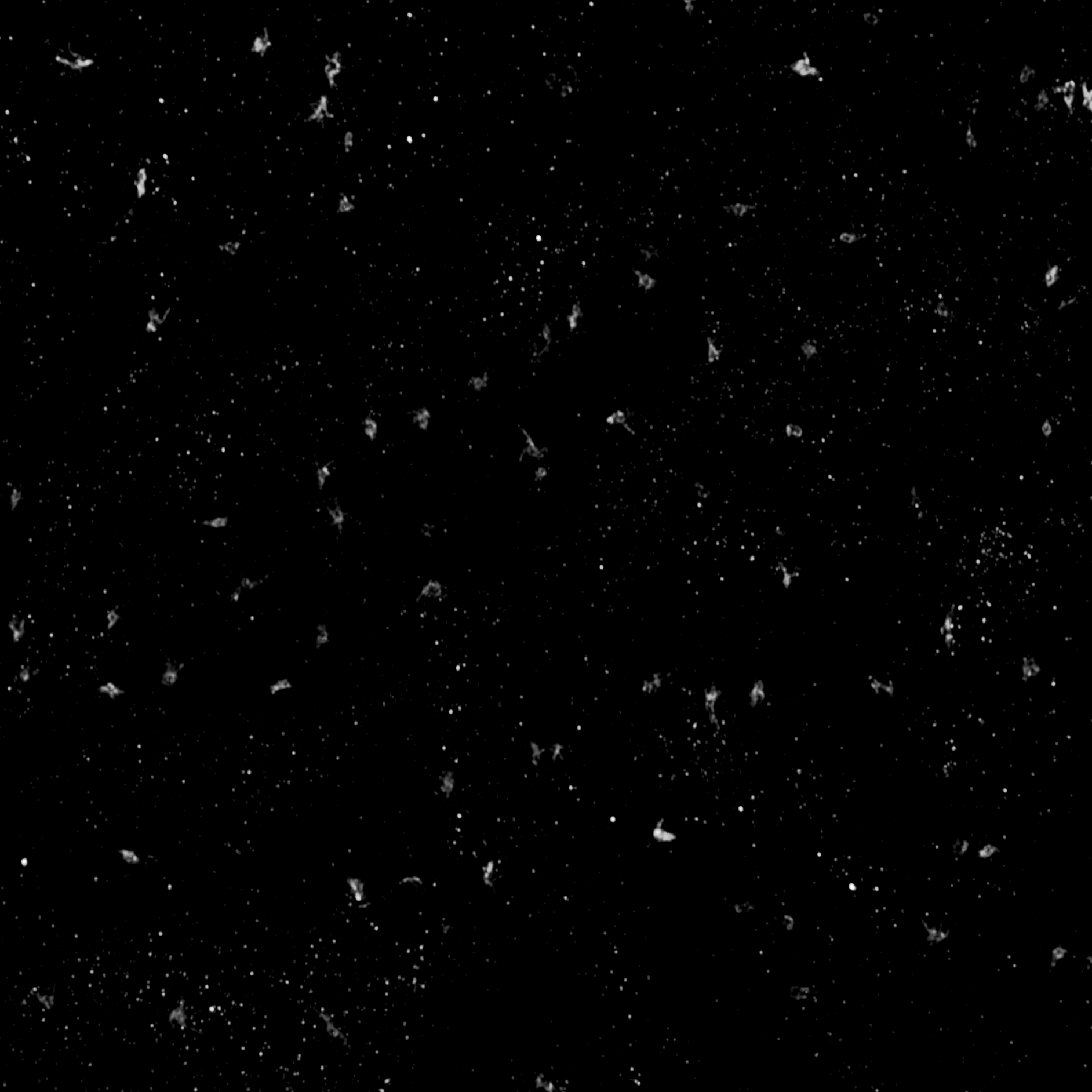

Supplement: Supplementary file 7 — Source data Fig. 4 [file 44319_2025_454_MOESM7_ESM.zip › Source Data - Figure 4/4A/Control hoechst, AceTub, PCNT, DBA P11.tif]

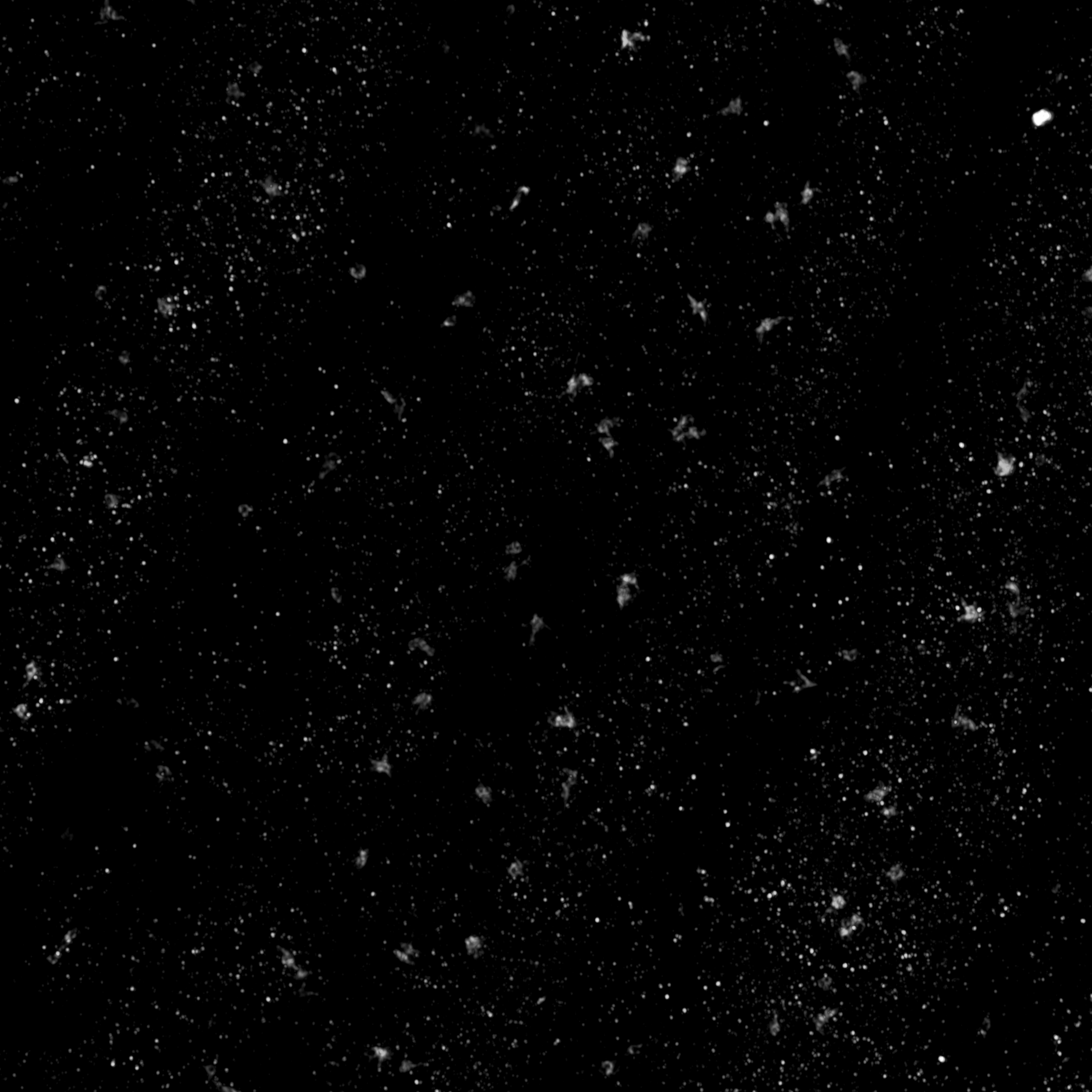

Supplement: Supplementary file 7 — Source data Fig. 4 [file 44319_2025_454_MOESM7_ESM.zip › Source Data - Figure 4/4A/GRcdKO hoechst, AceTub, PCNT, DBA P11.tif]

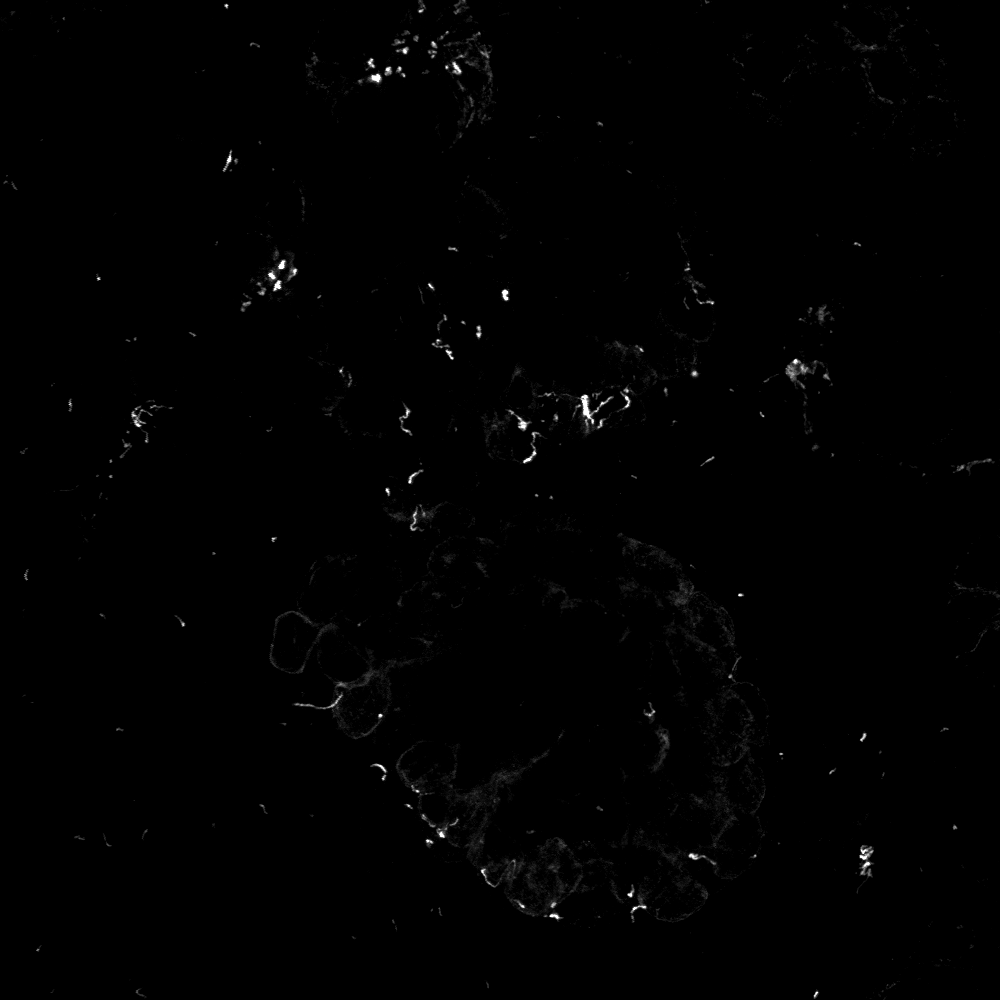

Supplement: Supplementary file 8 — Source data Fig. 5 [file 44319_2025_454_MOESM8_ESM.zip › Source Data - Figure 5/5A/Control hoechst, AceTub, NPHS1 E18.5.tif]

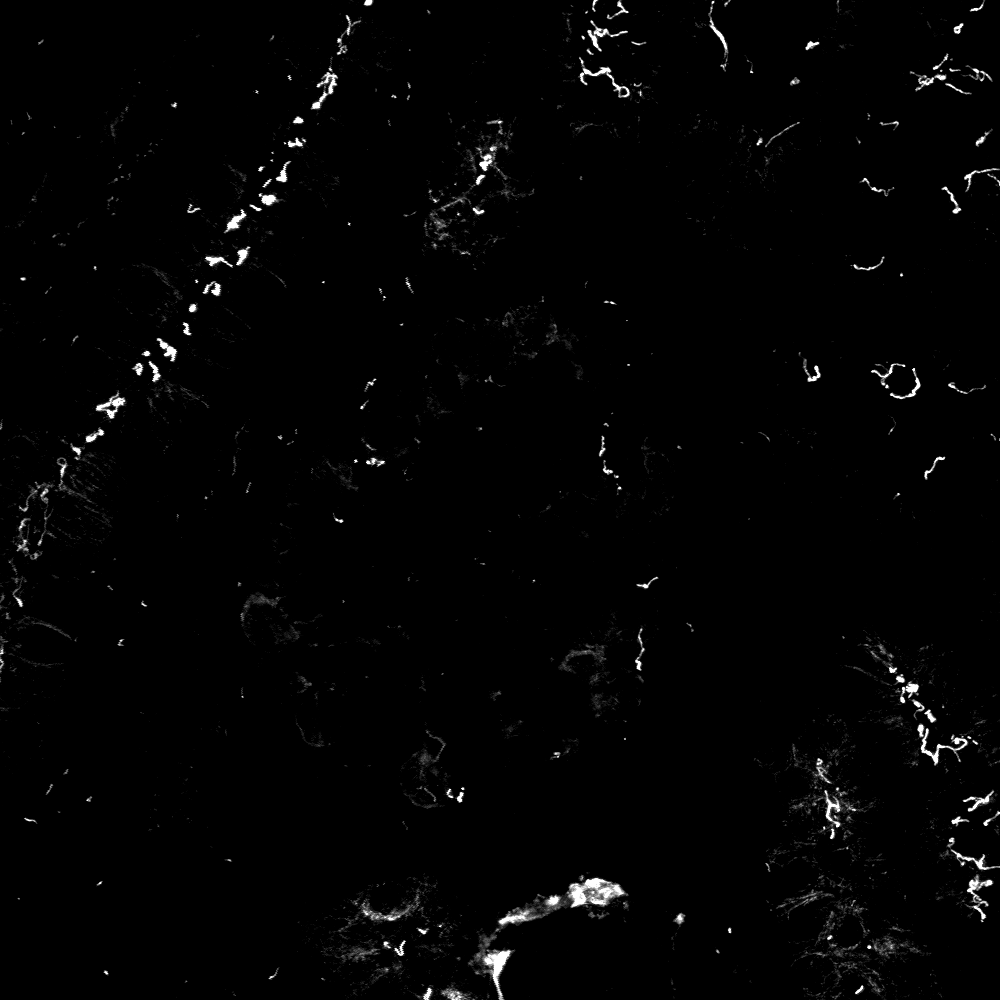

Supplement: Supplementary file 8 — Source data Fig. 5 [file 44319_2025_454_MOESM8_ESM.zip › Source Data - Figure 5/5A/GR-null hoechst, AceTub, NPHS1 E18.5.tif]

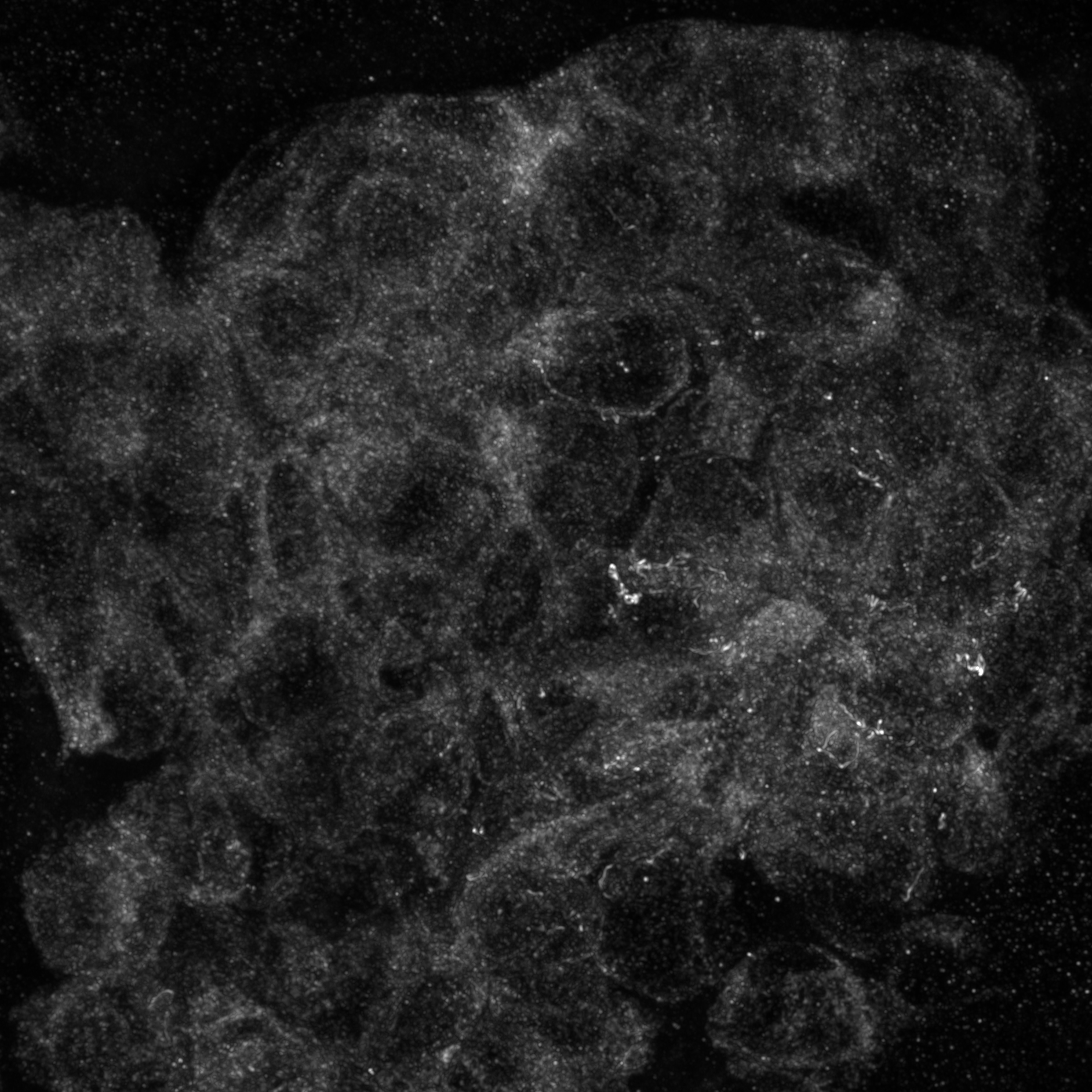

Supplement: Supplementary file 8 — Source data Fig. 5 [file 44319_2025_454_MOESM8_ESM.zip › Source Data - Figure 5/5E/iPSC dex hoechst, AceTub, NPHS1.tif]

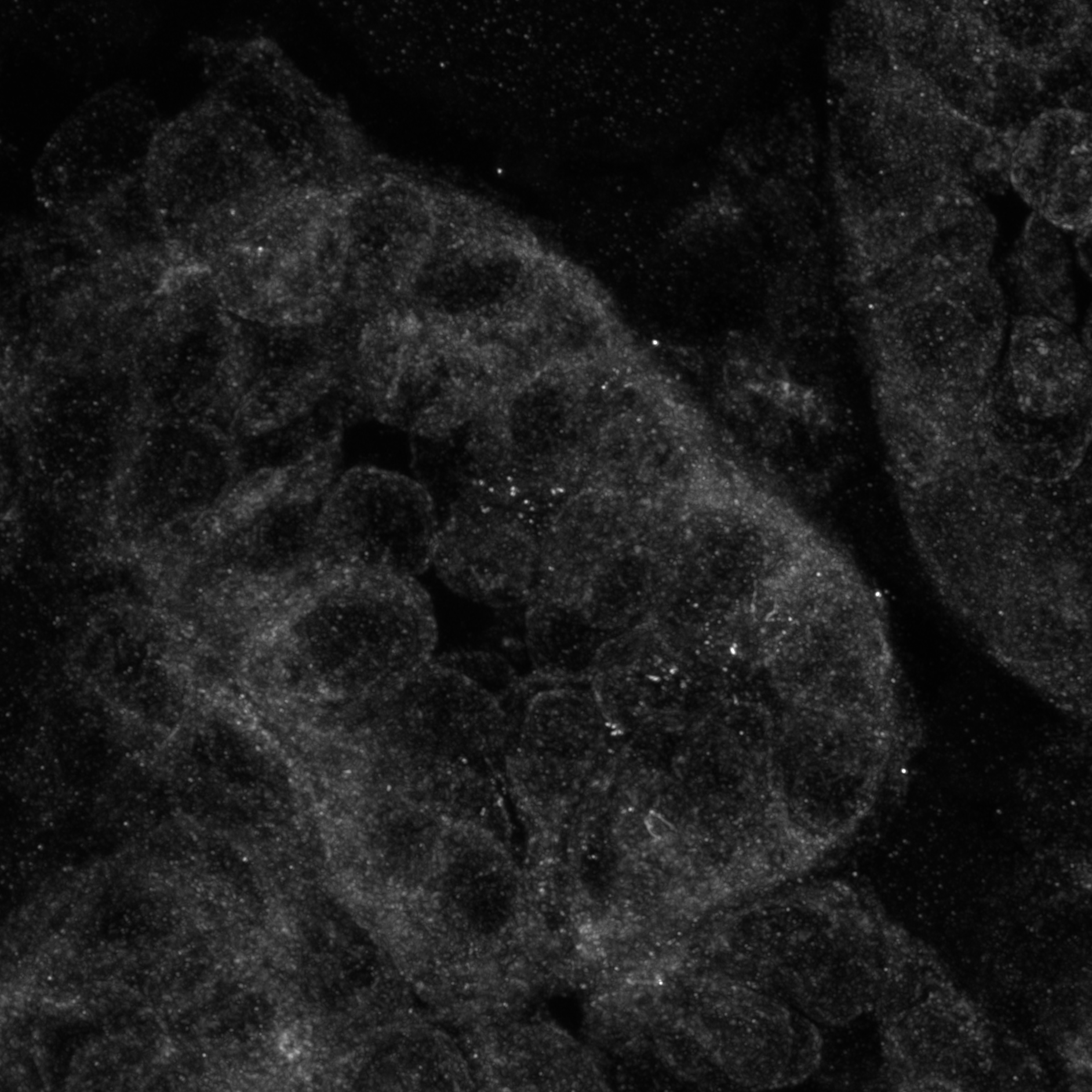

Supplement: Supplementary file 8 — Source data Fig. 5 [file 44319_2025_454_MOESM8_ESM.zip › Source Data - Figure 5/5E/iPSC veh hoechst, AceTub, NPHS1.tif]

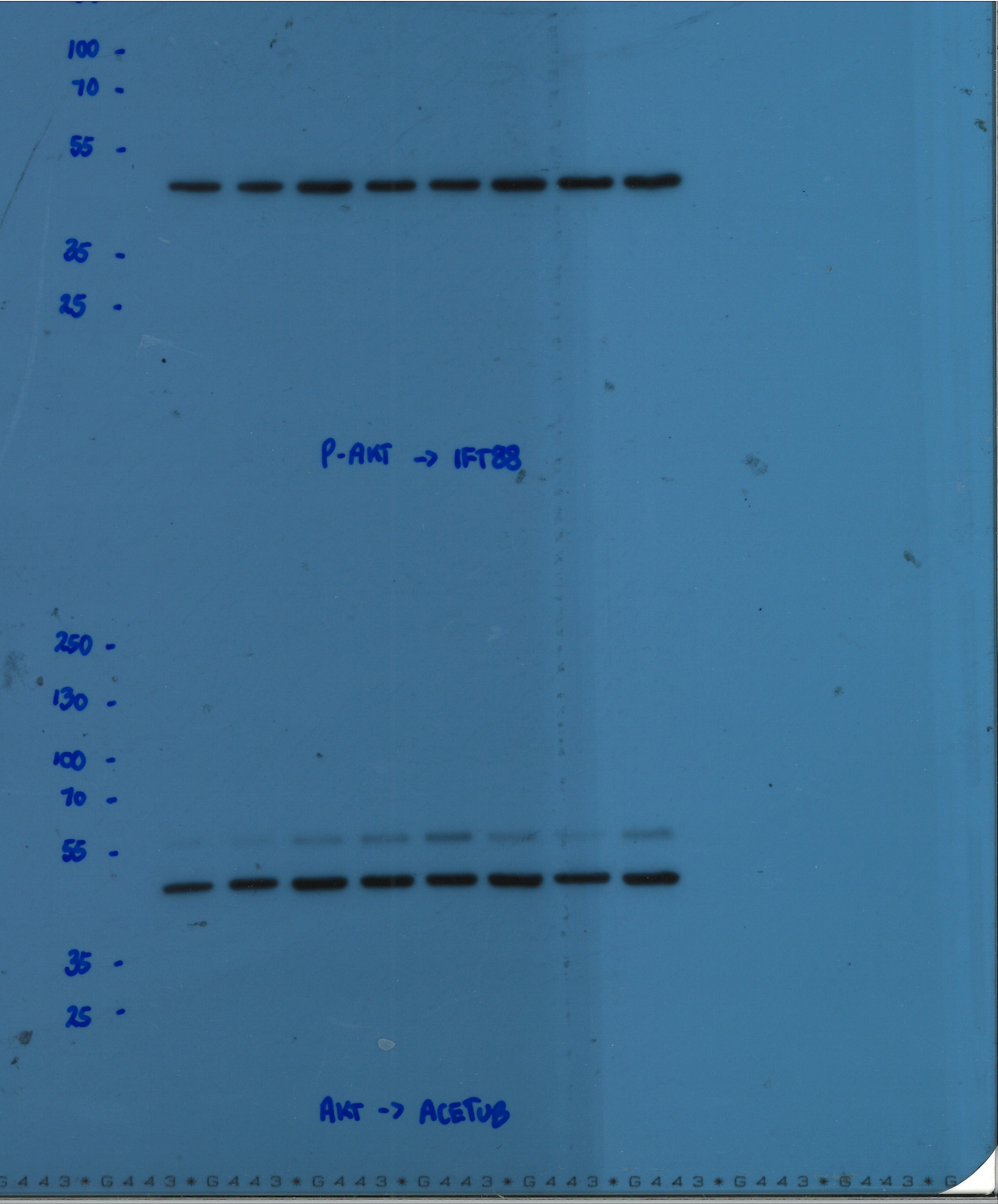

Supplement: Supplementary file 10 — Source data Fig. 7 [file 44319_2025_454_MOESM10_ESM.zip › Source Data - Figure 7/7A/P-AKT and AKT beta-actin.jpg]

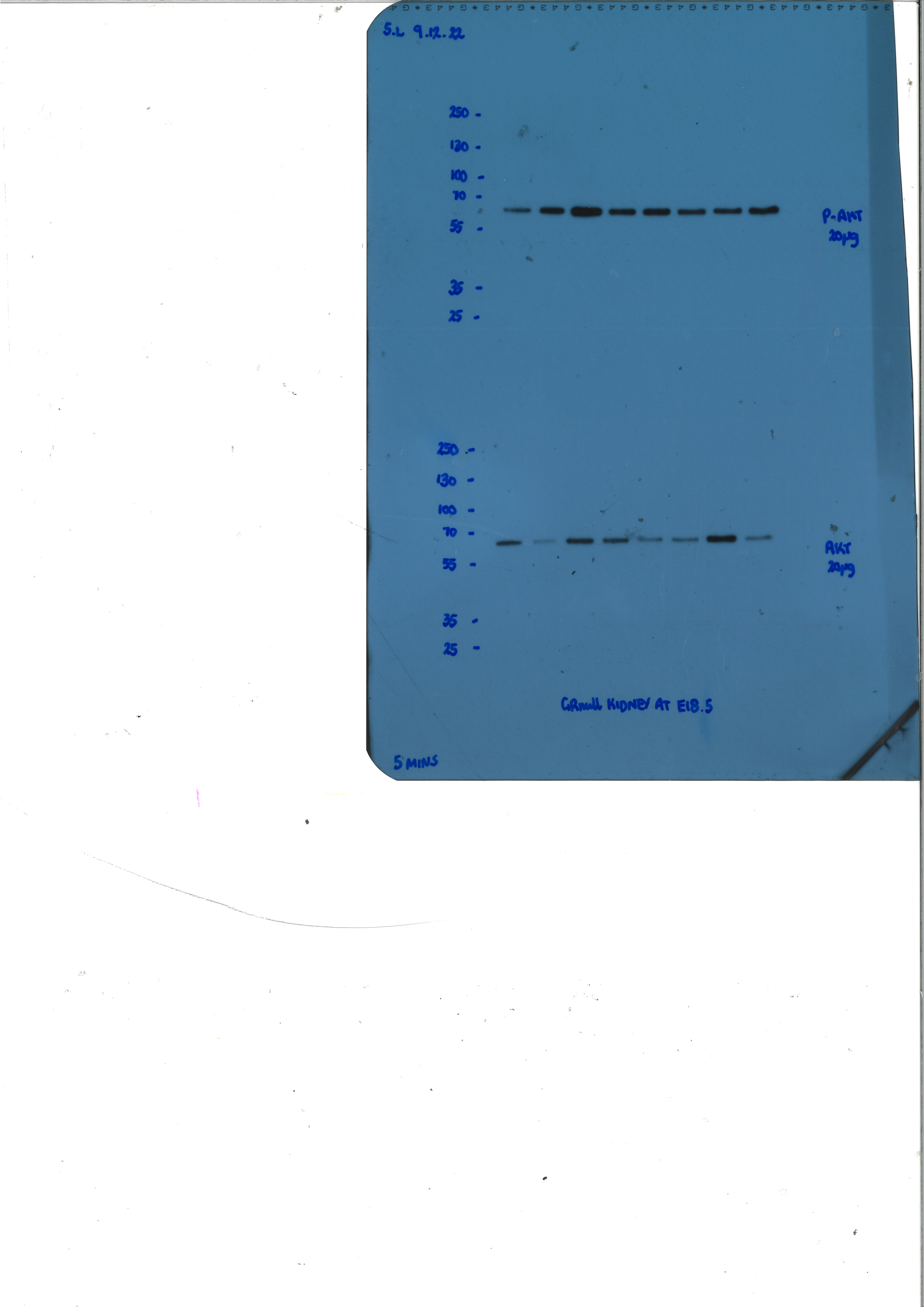

Supplement: Supplementary file 10 — Source data Fig. 7 [file 44319_2025_454_MOESM10_ESM.zip › Source Data - Figure 7/7A/P-AKT and AKT.jpg]

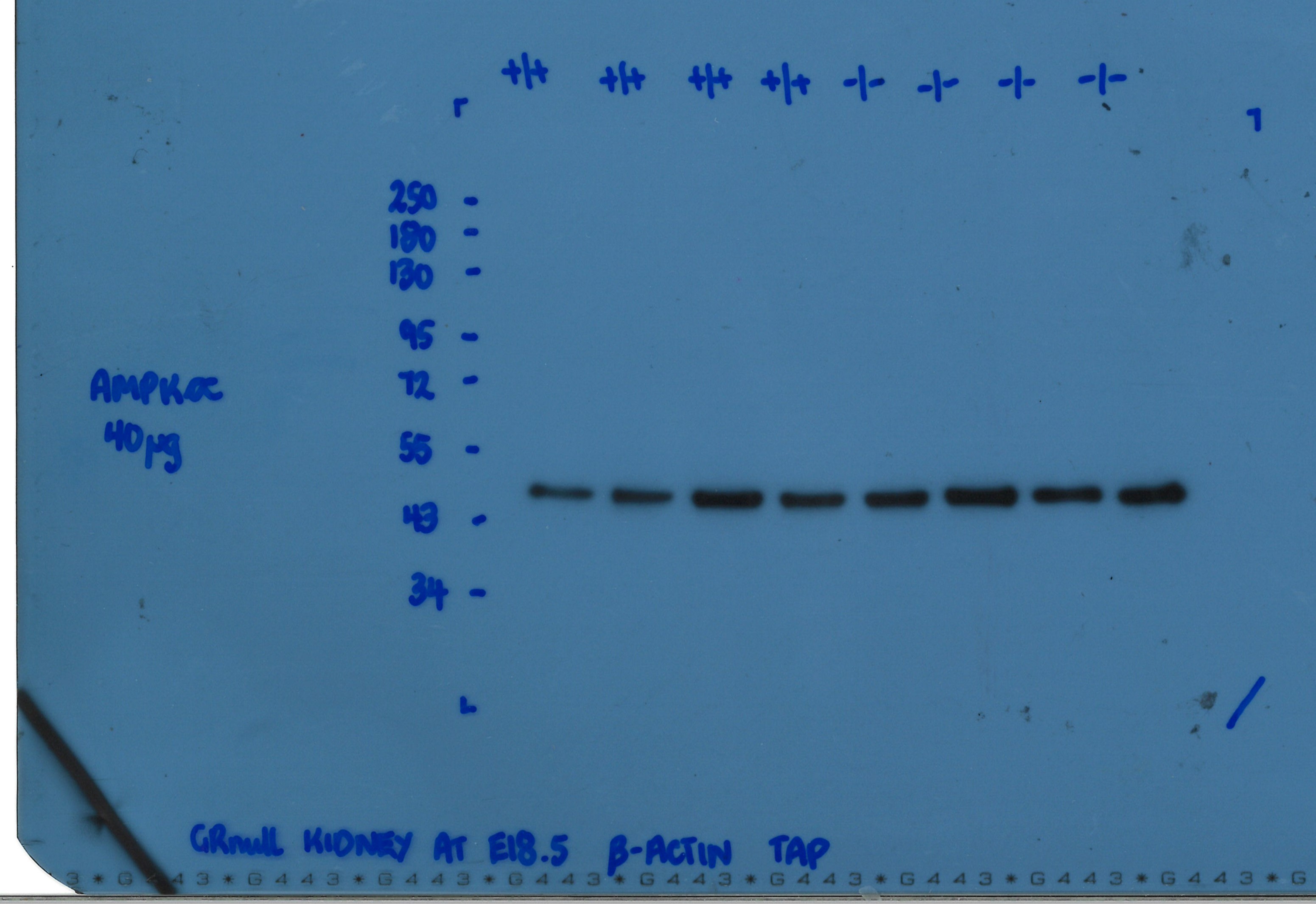

Supplement: Supplementary file 10 — Source data Fig. 7 [file 44319_2025_454_MOESM10_ESM.zip › Source Data - Figure 7/7B/AMPKa beta-actin.jpg]

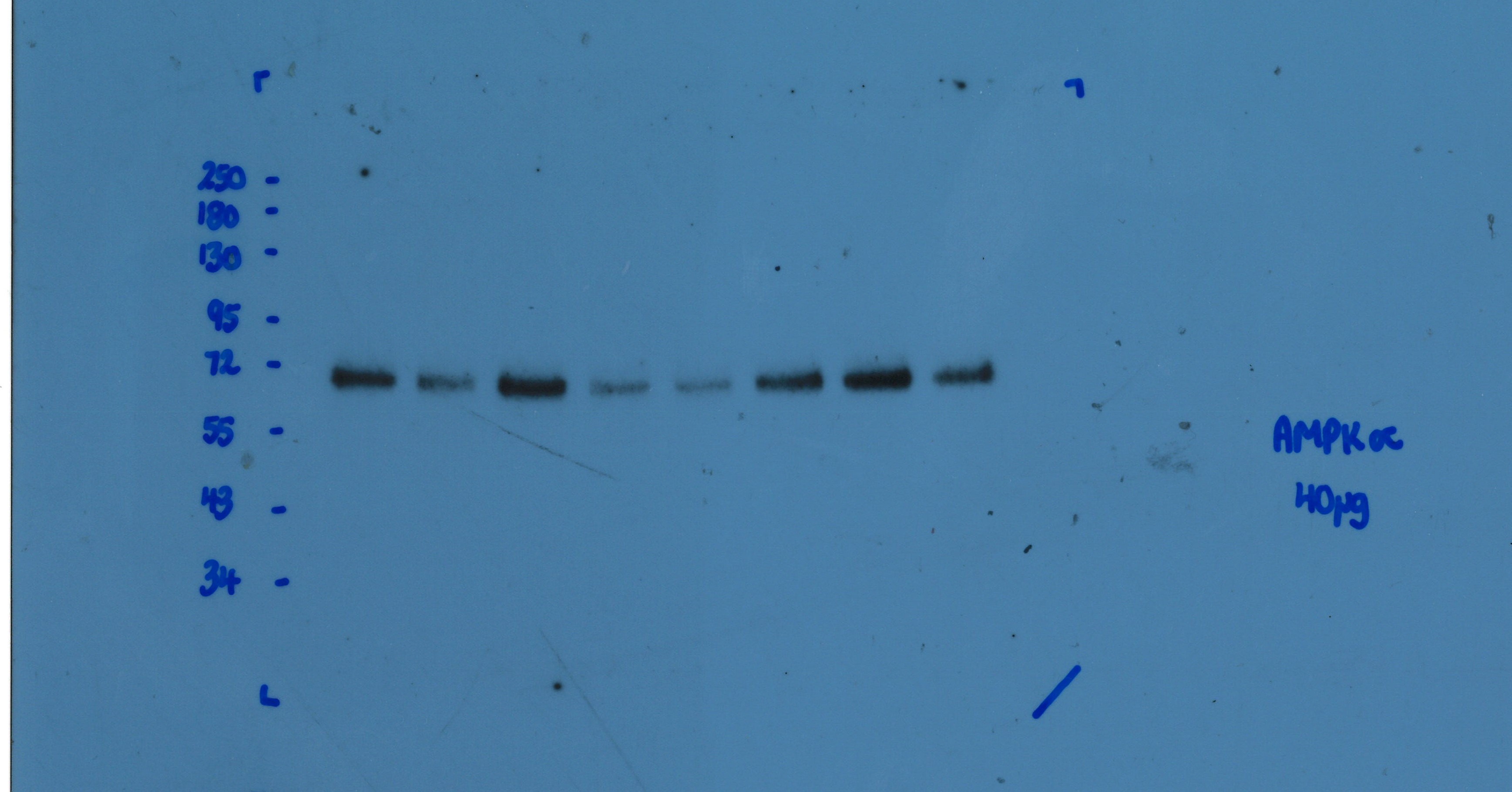

Supplement: Supplementary file 10 — Source data Fig. 7 [file 44319_2025_454_MOESM10_ESM.zip › Source Data - Figure 7/7B/AMPKa.jpg]

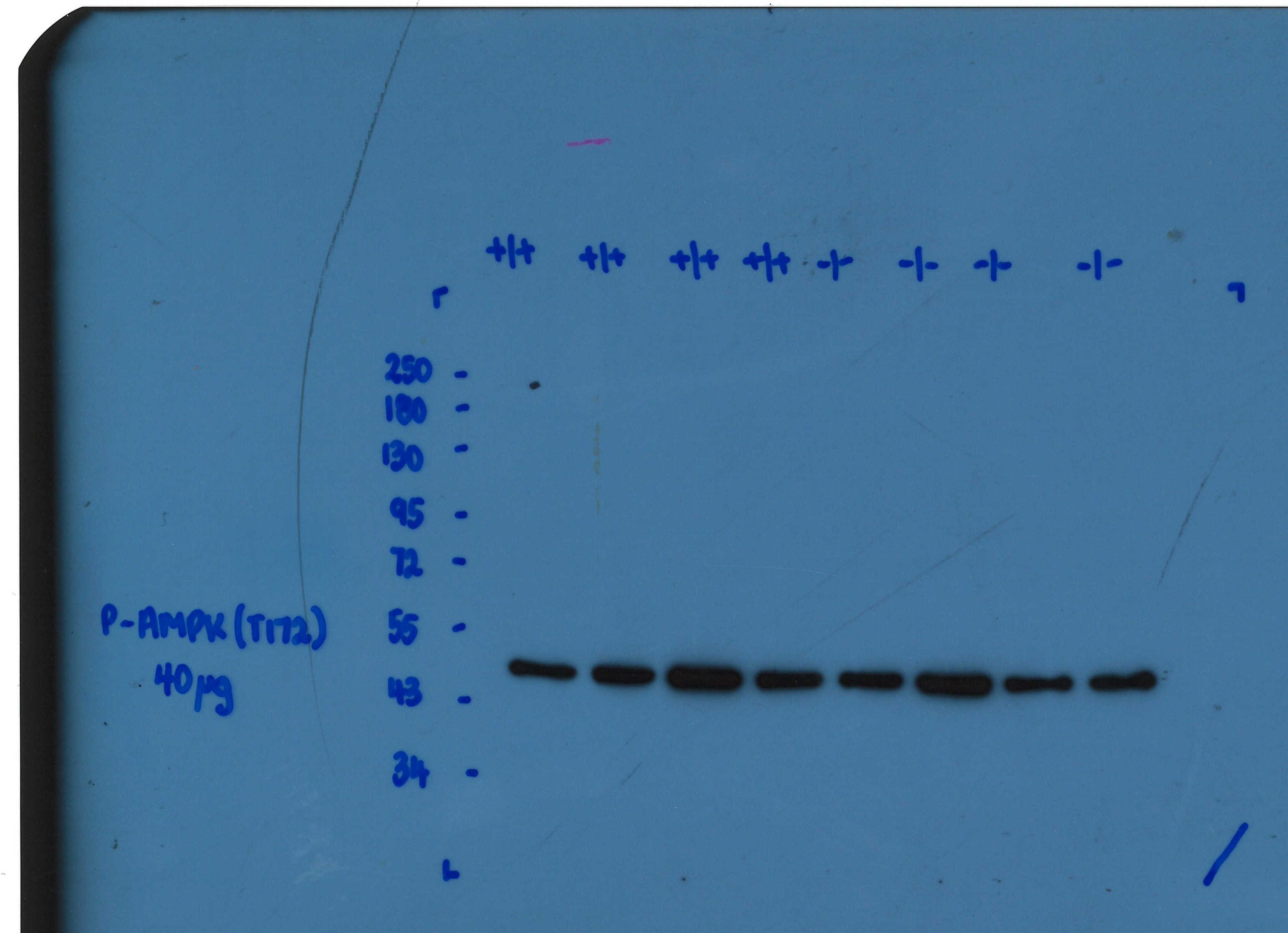

Supplement: Supplementary file 10 — Source data Fig. 7 [file 44319_2025_454_MOESM10_ESM.zip › Source Data - Figure 7/7B/P-AMPKa beta-actin.jpg]

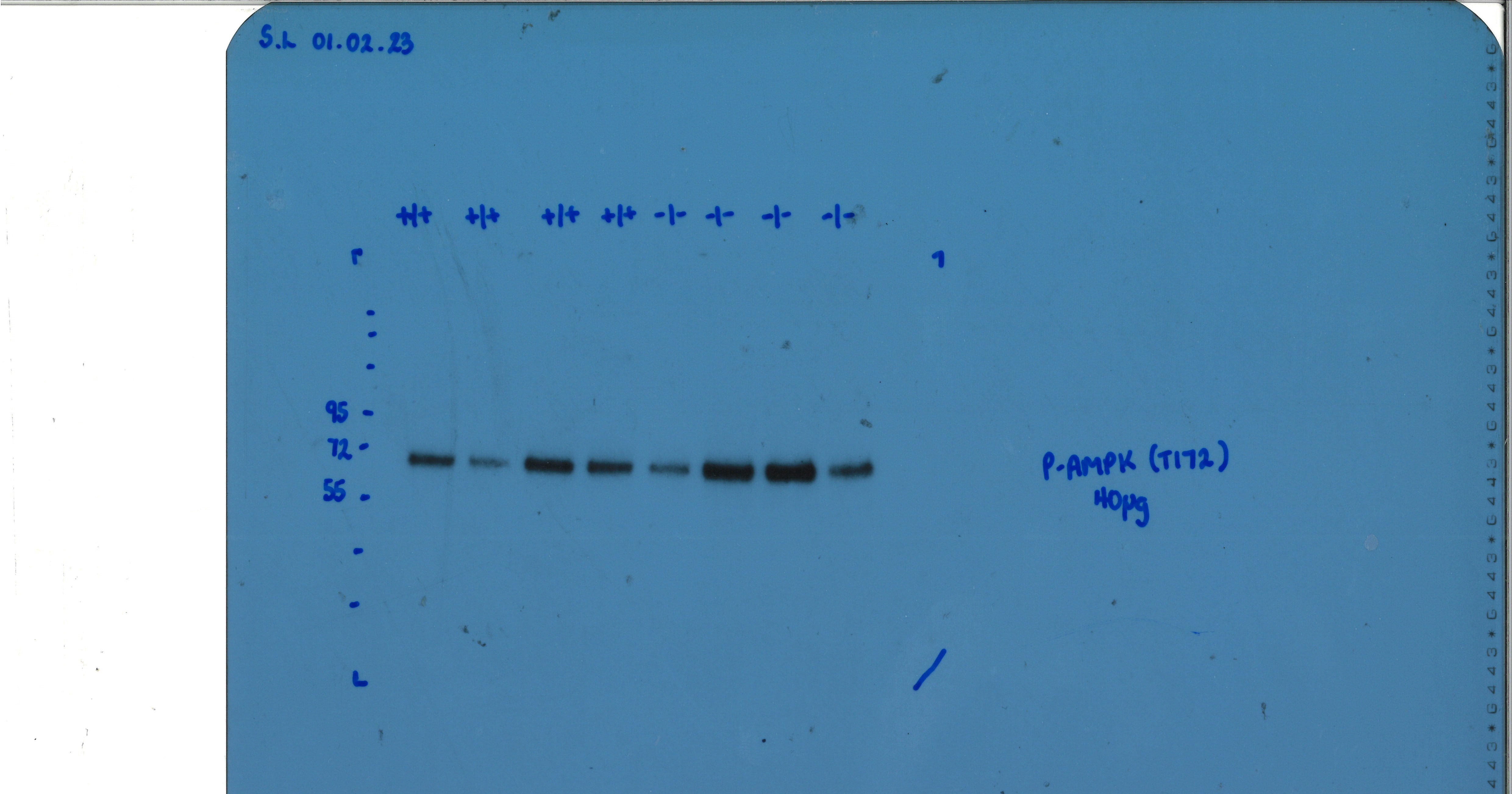

Supplement: Supplementary file 10 — Source data Fig. 7 [file 44319_2025_454_MOESM10_ESM.zip › Source Data - Figure 7/7B/P-AMPKa.jpg]

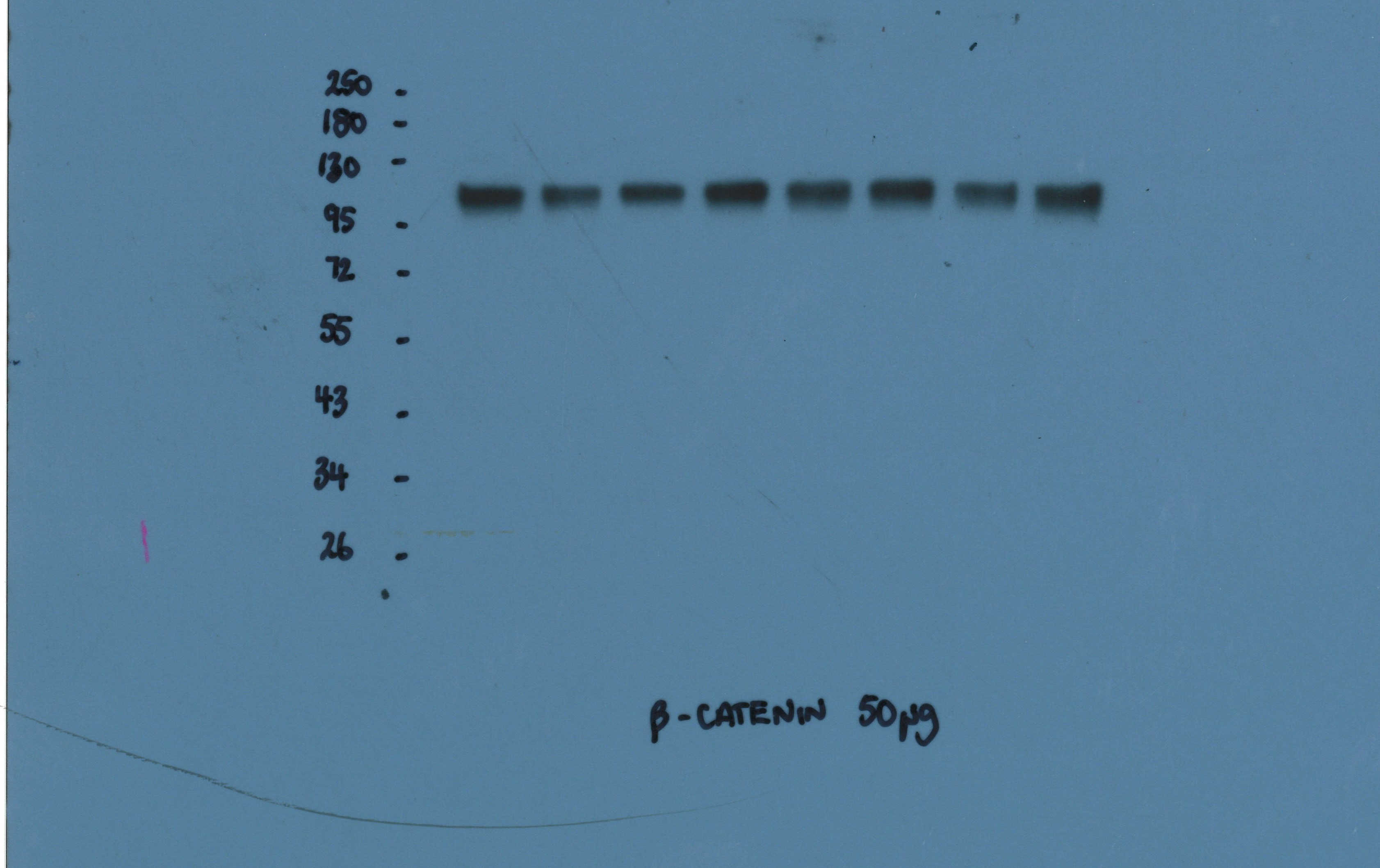

Supplement: Supplementary file 10 — Source data Fig. 7 [file 44319_2025_454_MOESM10_ESM.zip › Source Data - Figure 7/7C/Beta-catenin.jpg]

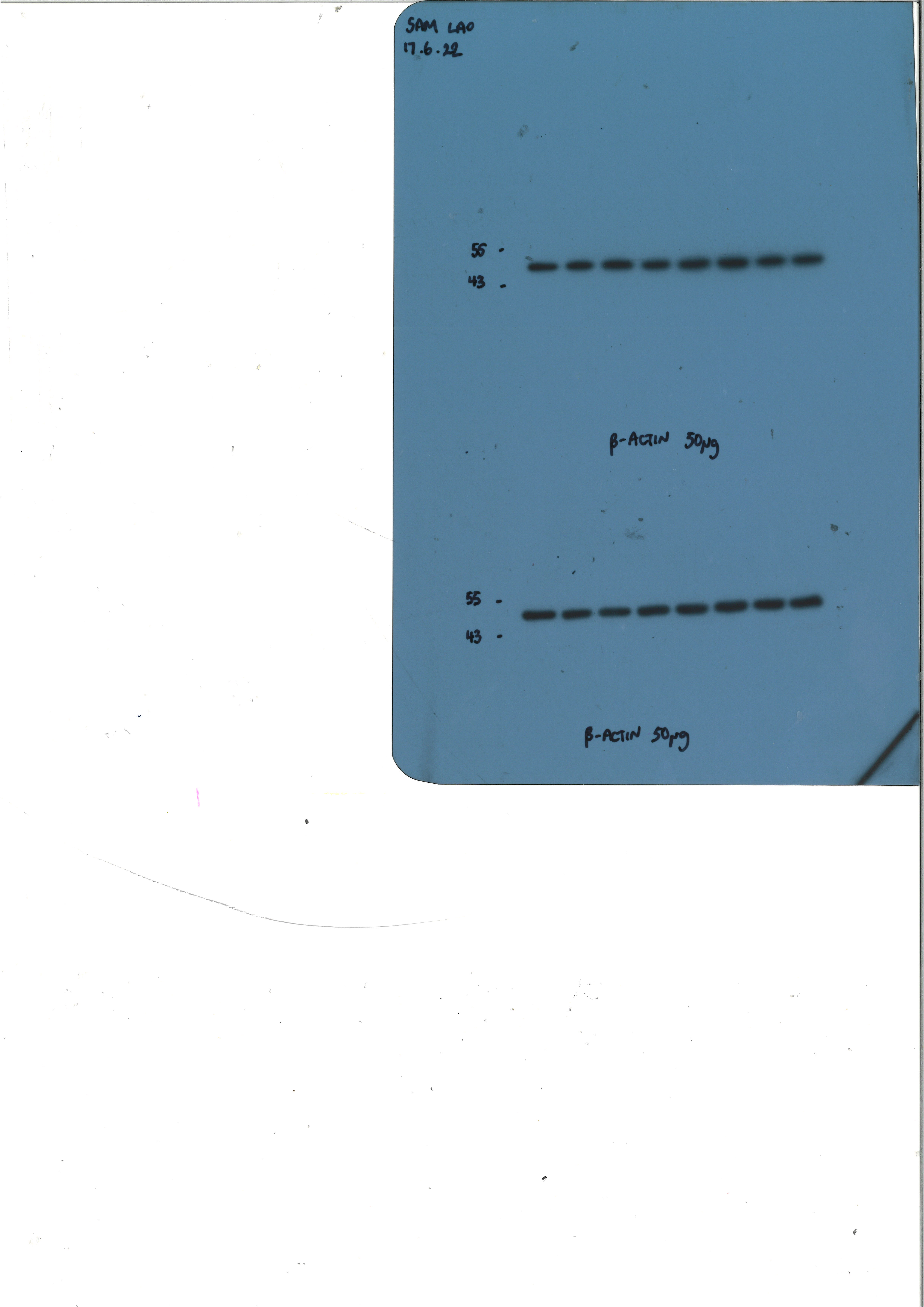

Supplement: Supplementary file 10 — Source data Fig. 7 [file 44319_2025_454_MOESM10_ESM.zip › Source Data - Figure 7/7C/P-beta catenin and beta-catenin beta-actin.jpg]

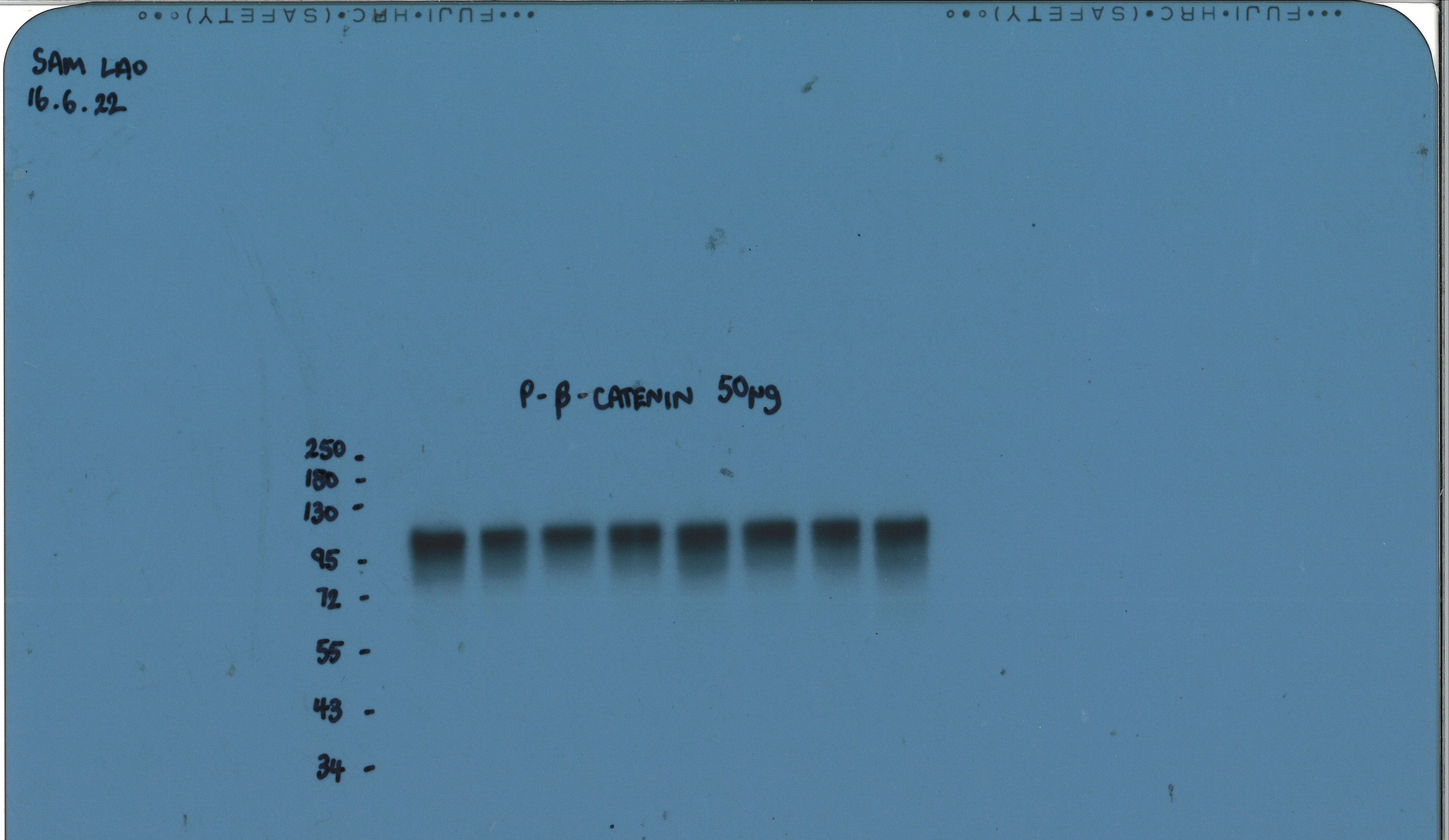

Supplement: Supplementary file 10 — Source data Fig. 7 [file 44319_2025_454_MOESM10_ESM.zip › Source Data - Figure 7/7C/P-beta-catenin.jpg]

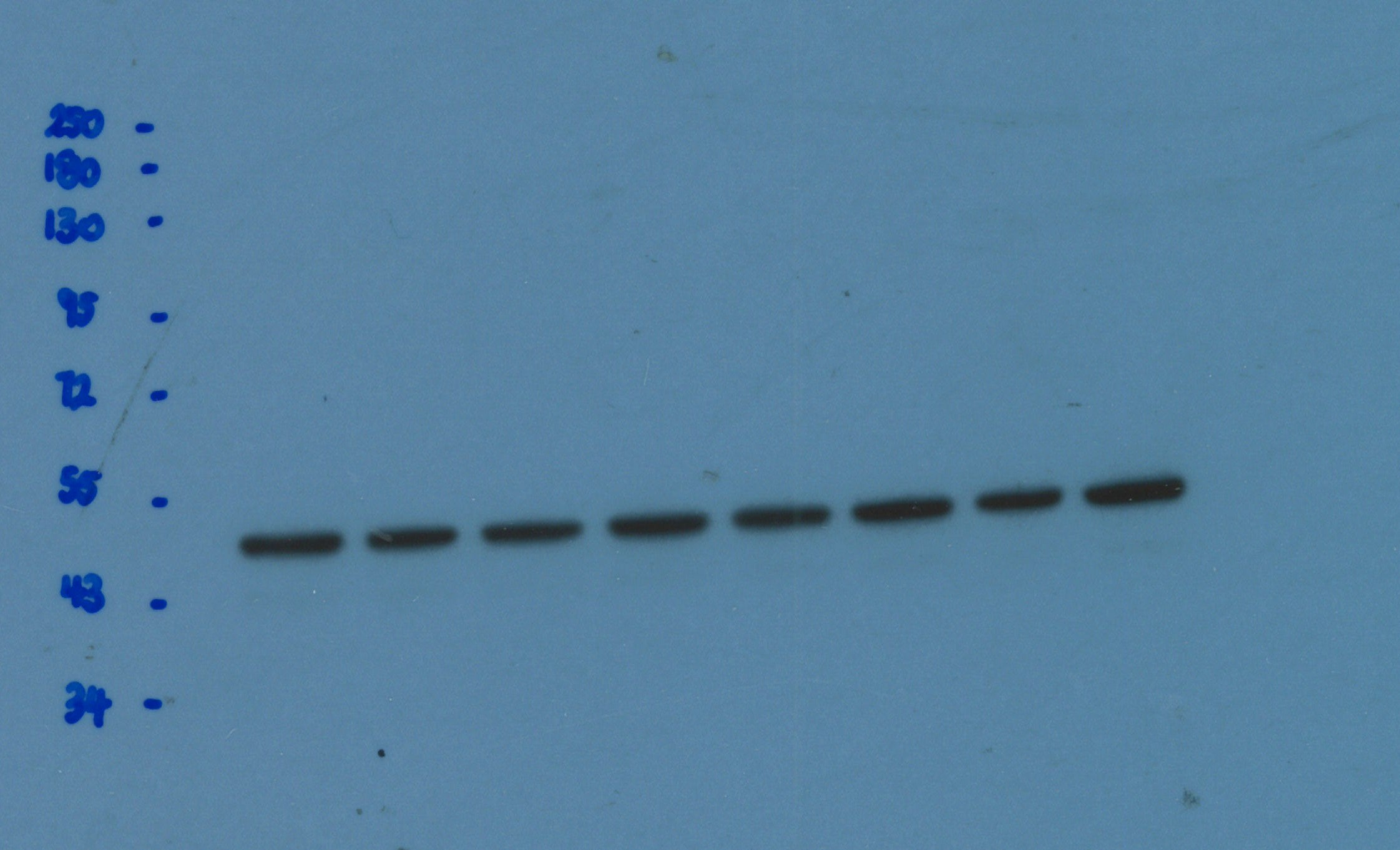

Supplement: Supplementary file 10 — Source data Fig. 7 [file 44319_2025_454_MOESM10_ESM.zip › Source Data - Figure 7/7D/ERK beta-actin.jpg]

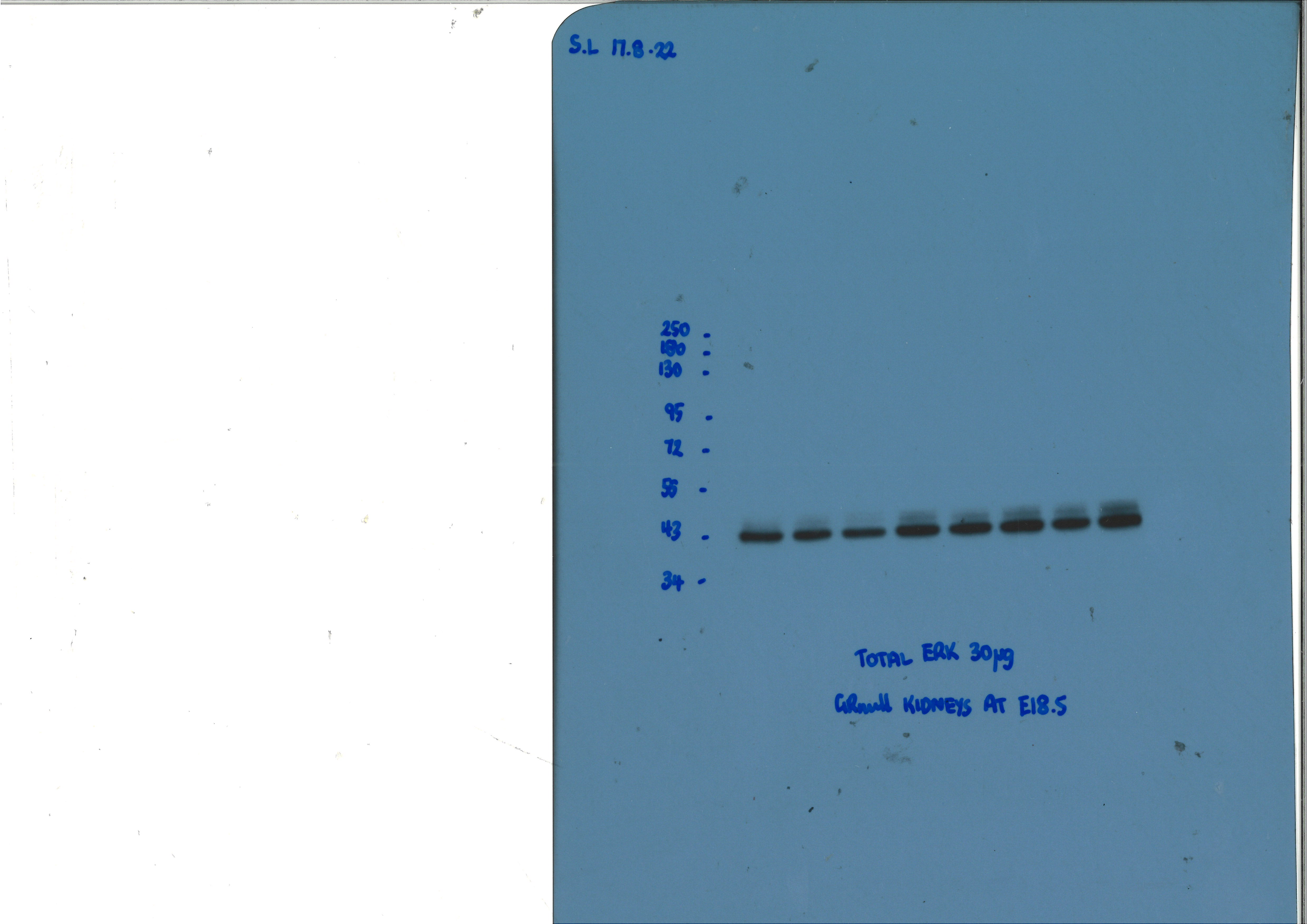

Supplement: Supplementary file 10 — Source data Fig. 7 [file 44319_2025_454_MOESM10_ESM.zip › Source Data - Figure 7/7D/ERK.jpg]

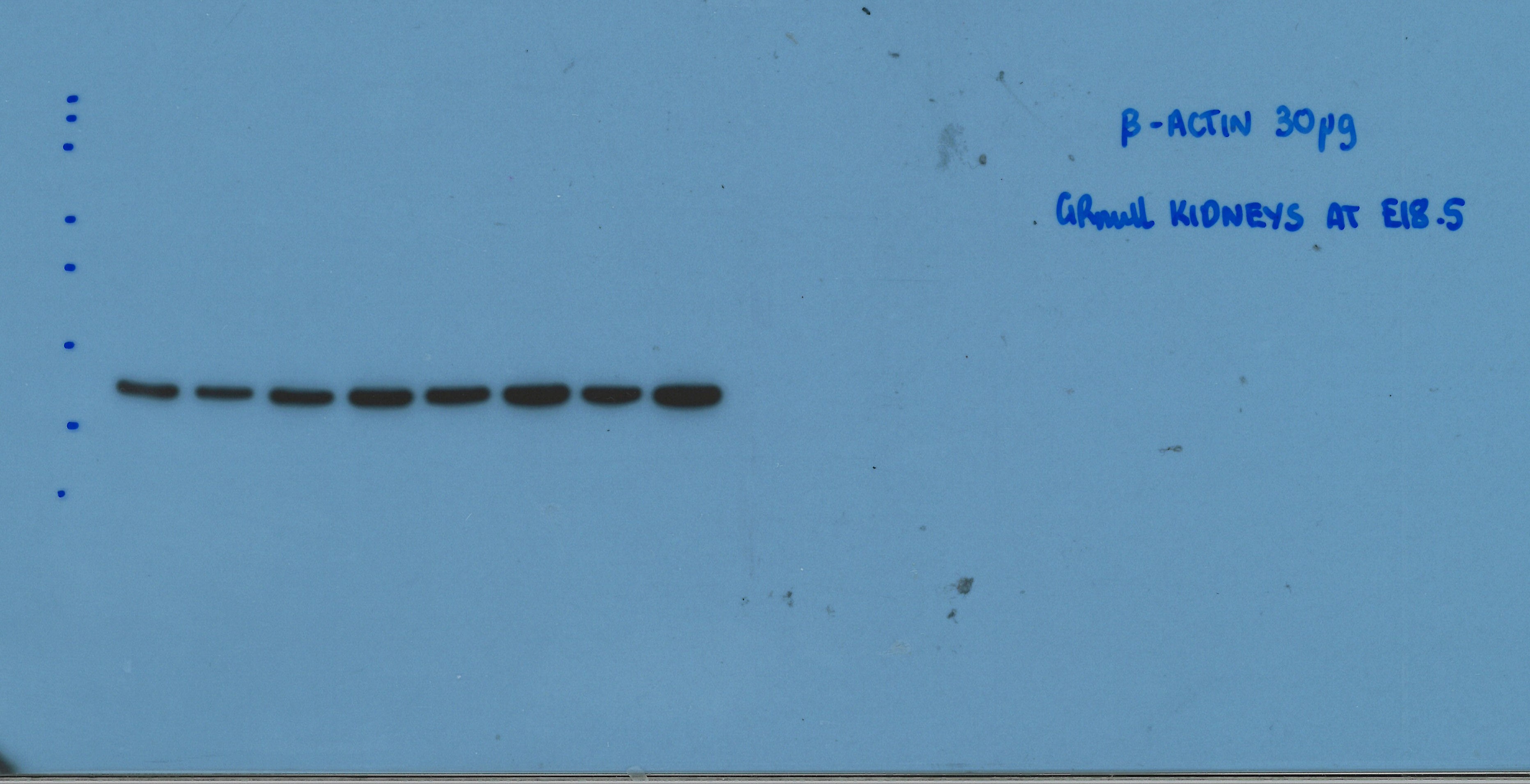

Supplement: Supplementary file 10 — Source data Fig. 7 [file 44319_2025_454_MOESM10_ESM.zip › Source Data - Figure 7/7D/P-ERK beta-actin.jpg]

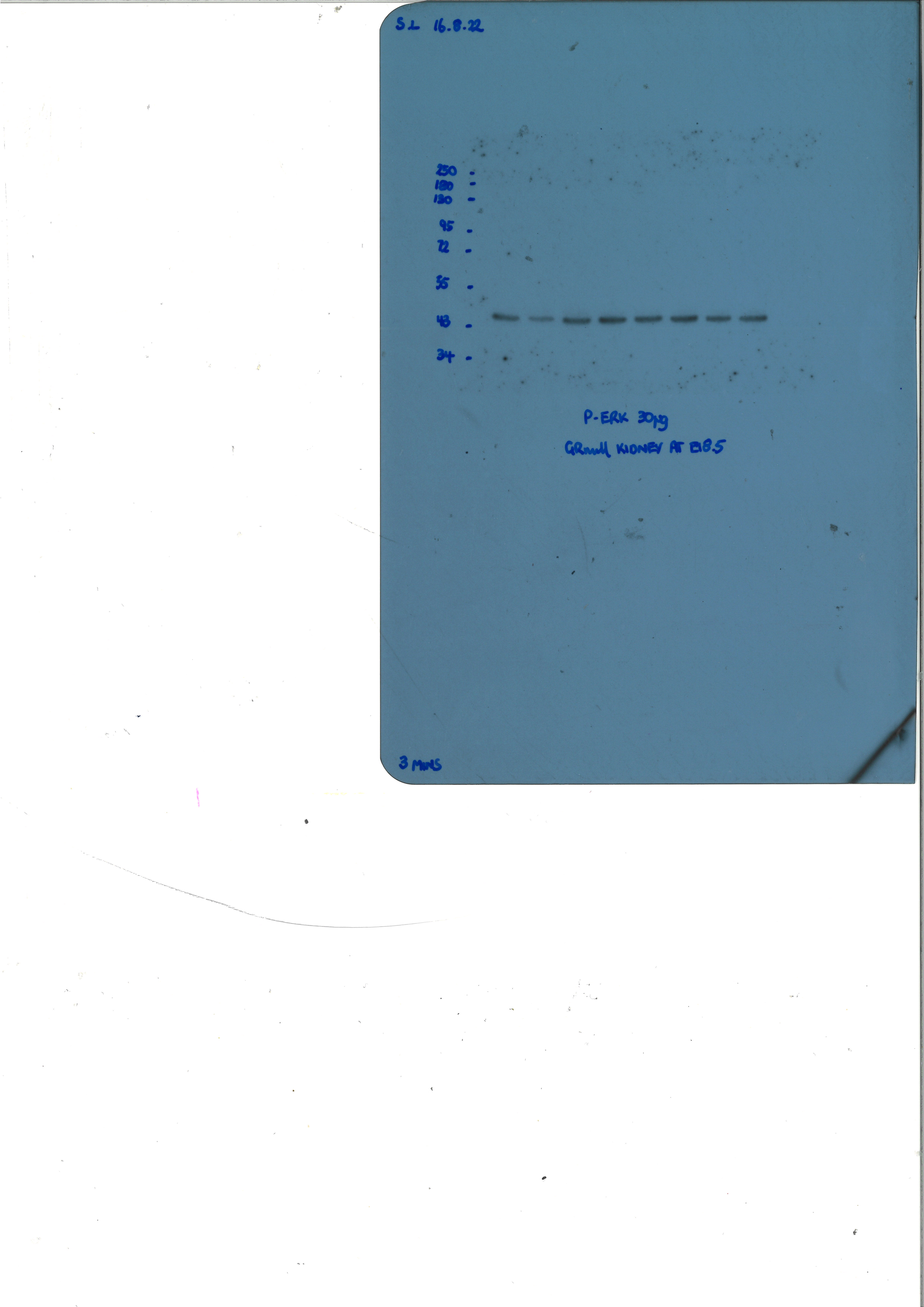

Supplement: Supplementary file 10 — Source data Fig. 7 [file 44319_2025_454_MOESM10_ESM.zip › Source Data - Figure 7/7D/P-ERK.jpg]

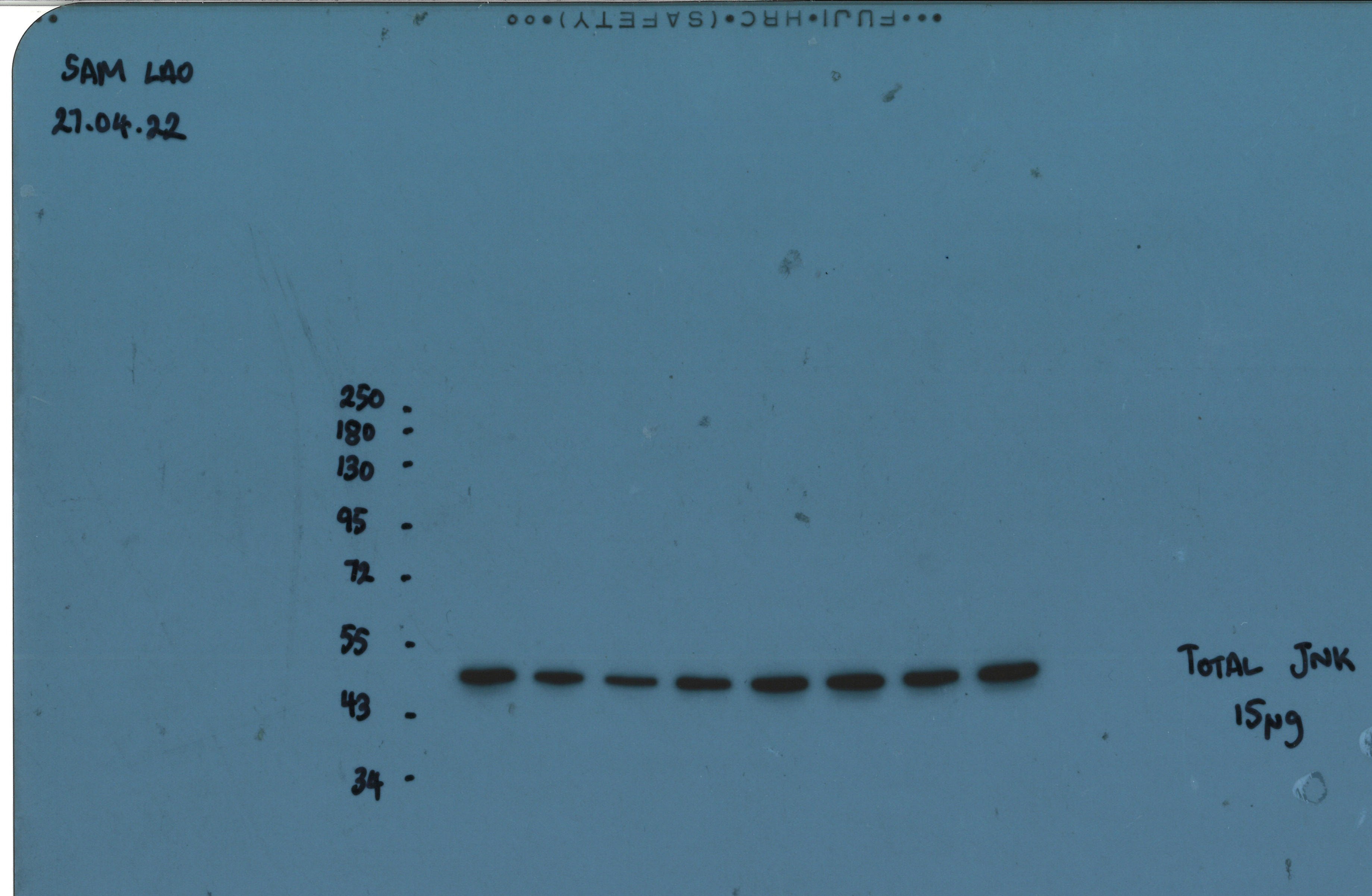

Supplement: Supplementary file 10 — Source data Fig. 7 [file 44319_2025_454_MOESM10_ESM.zip › Source Data - Figure 7/7E/JNK beta-actin.jpg]

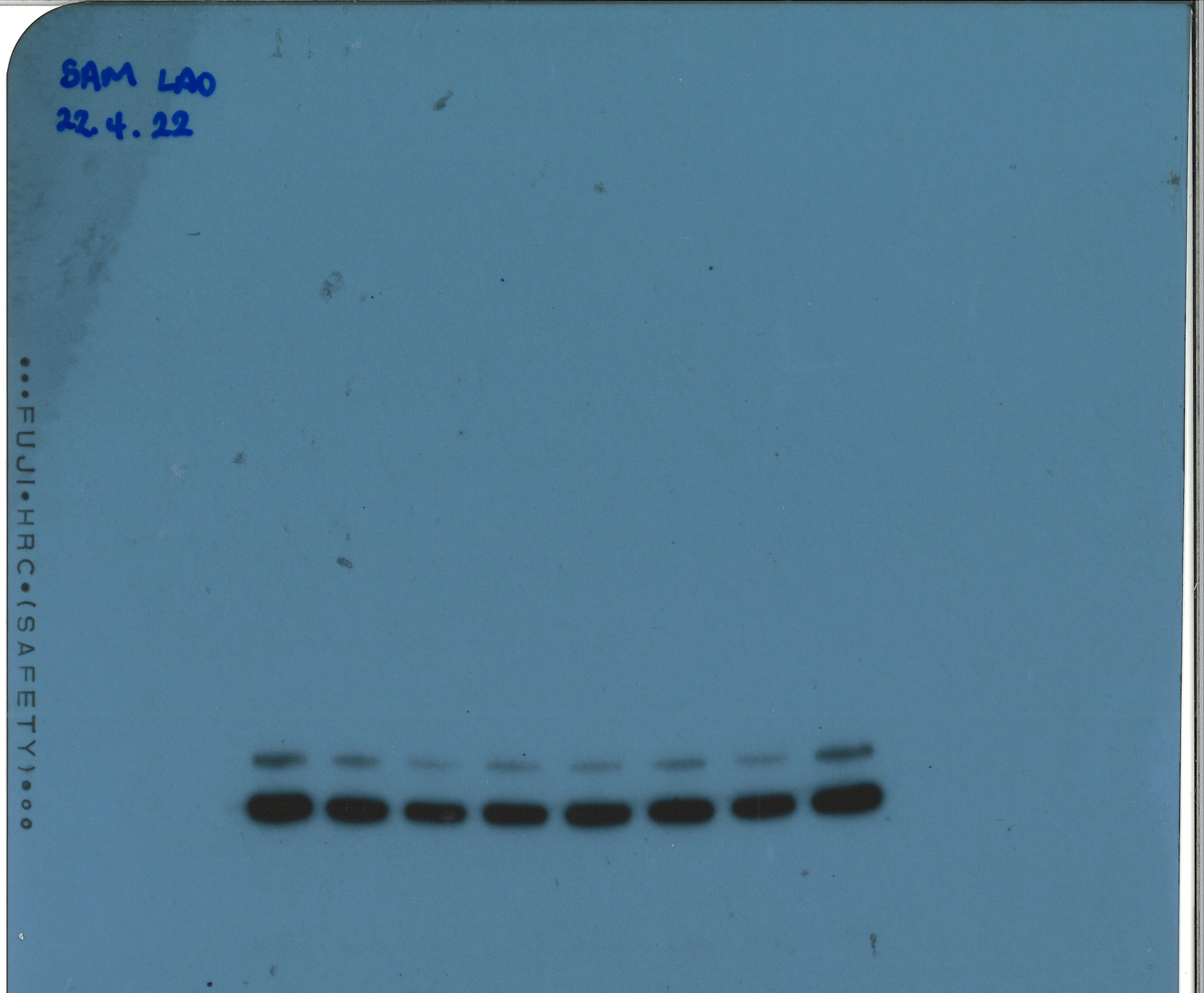

Supplement: Supplementary file 10 — Source data Fig. 7 [file 44319_2025_454_MOESM10_ESM.zip › Source Data - Figure 7/7E/JNK.jpg]

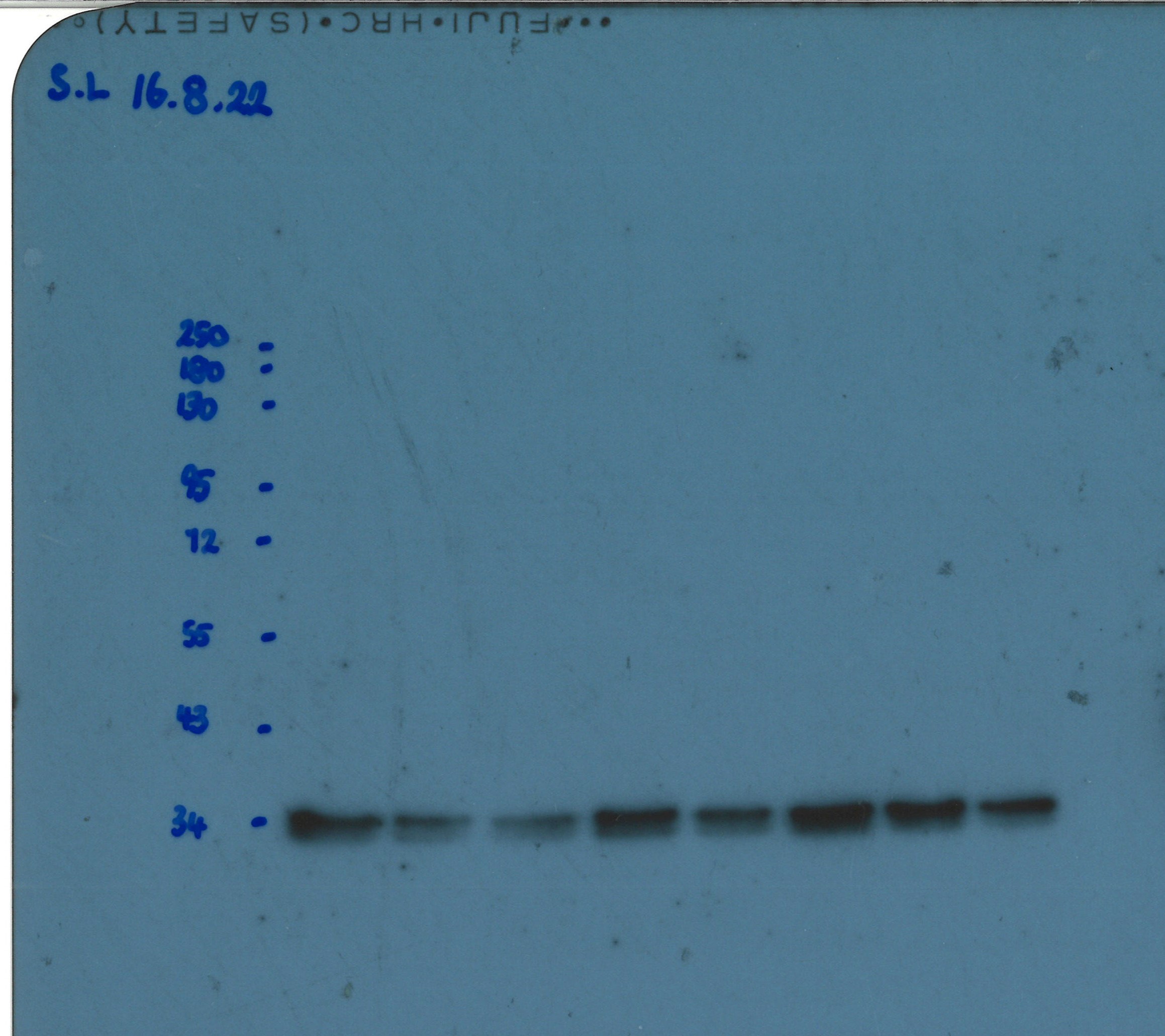

Supplement: Supplementary file 10 — Source data Fig. 7 [file 44319_2025_454_MOESM10_ESM.zip › Source Data - Figure 7/7F/P-S6.jpg]

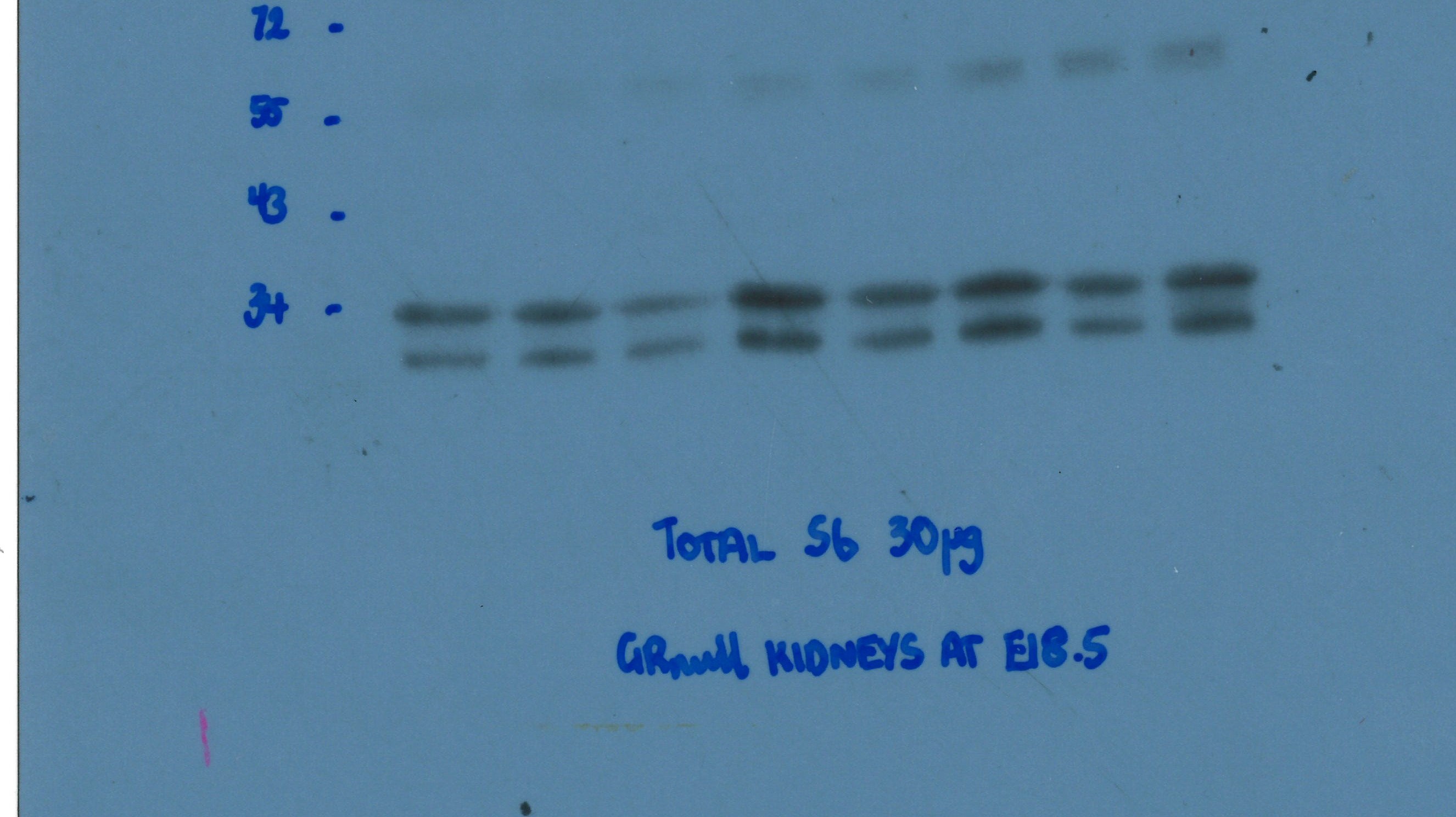

Supplement: Supplementary file 10 — Source data Fig. 7 [file 44319_2025_454_MOESM10_ESM.zip › Source Data - Figure 7/7F/S6.jpg]

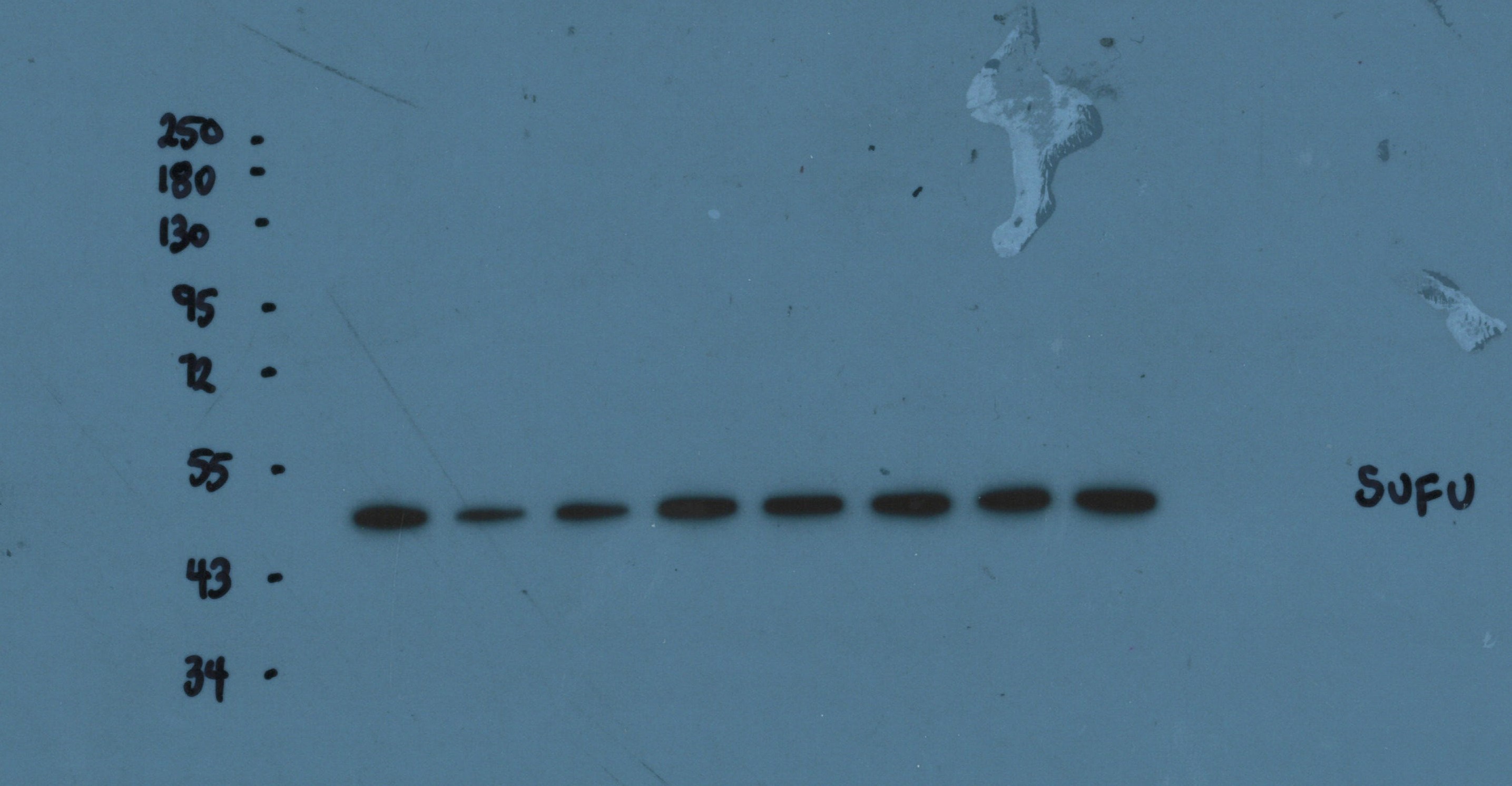

Supplement: Supplementary file 10 — Source data Fig. 7 [file 44319_2025_454_MOESM10_ESM.zip › Source Data - Figure 7/7G/SUFU beta-actin.jpg]

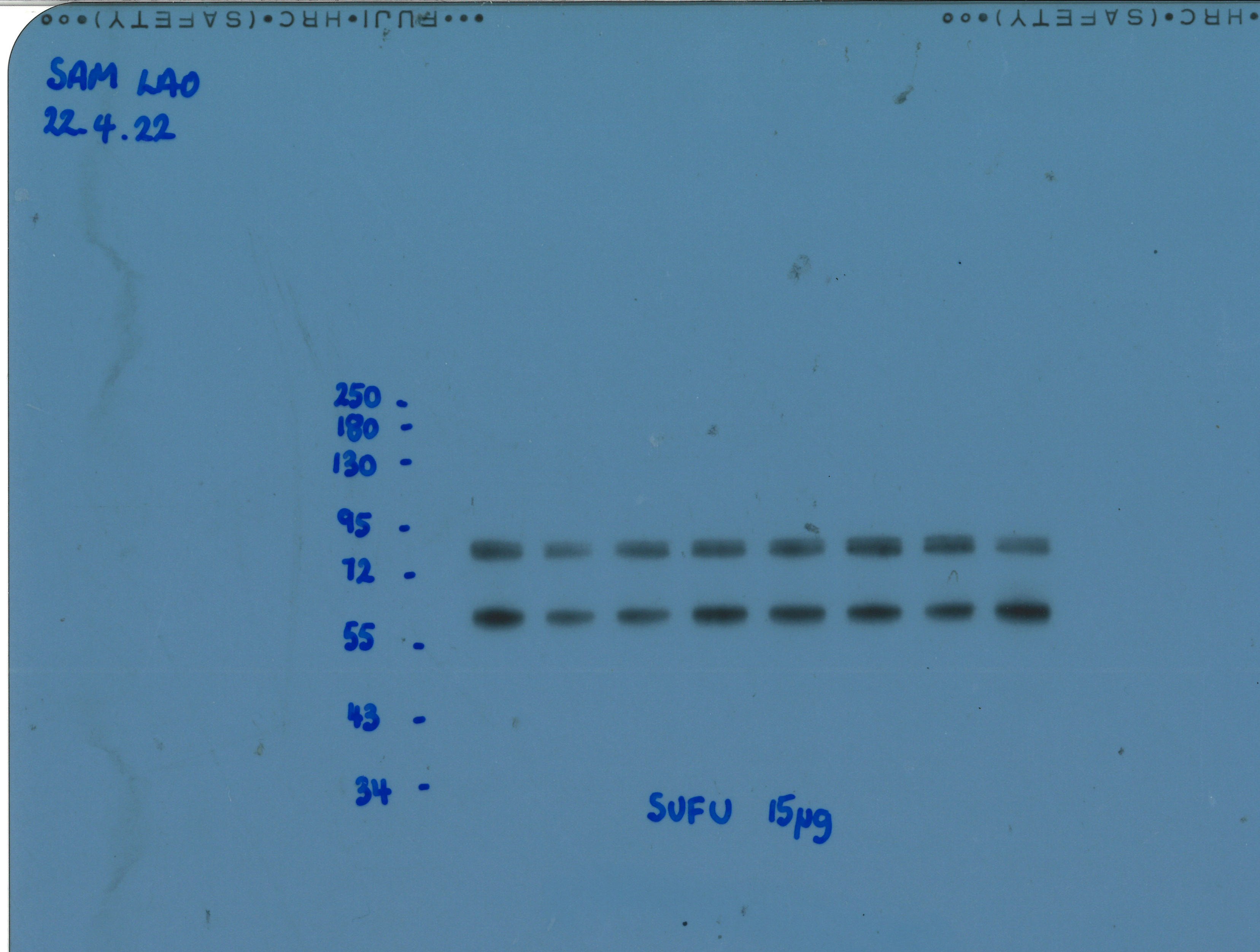

Supplement: Supplementary file 10 — Source data Fig. 7 [file 44319_2025_454_MOESM10_ESM.zip › Source Data - Figure 7/7G/SUFU.jpg]

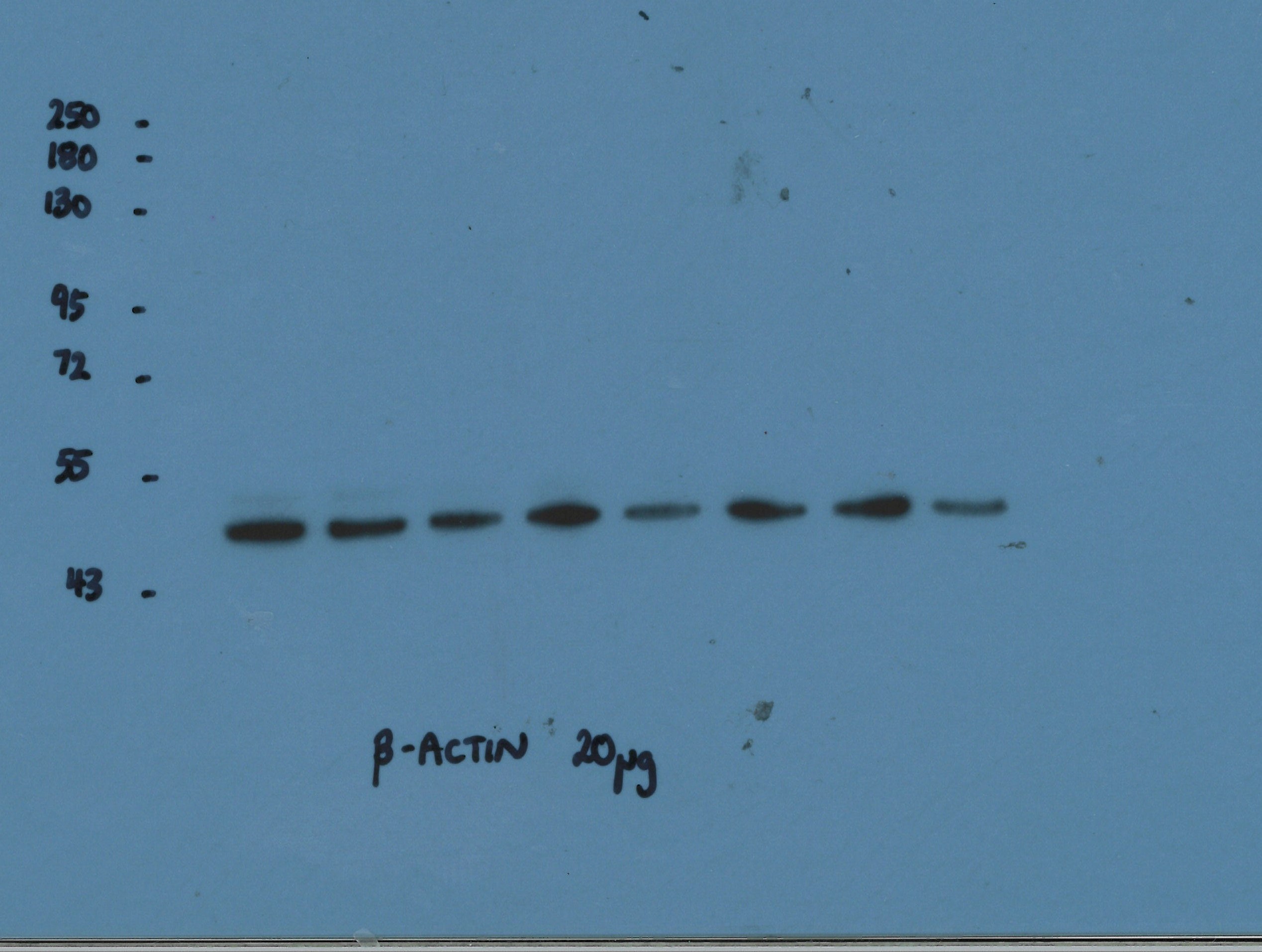

Supplement: Supplementary file 10 — Source data Fig. 7 [file 44319_2025_454_MOESM10_ESM.zip › Source Data - Figure 7/7H/AURKA beta-actin.jpg]

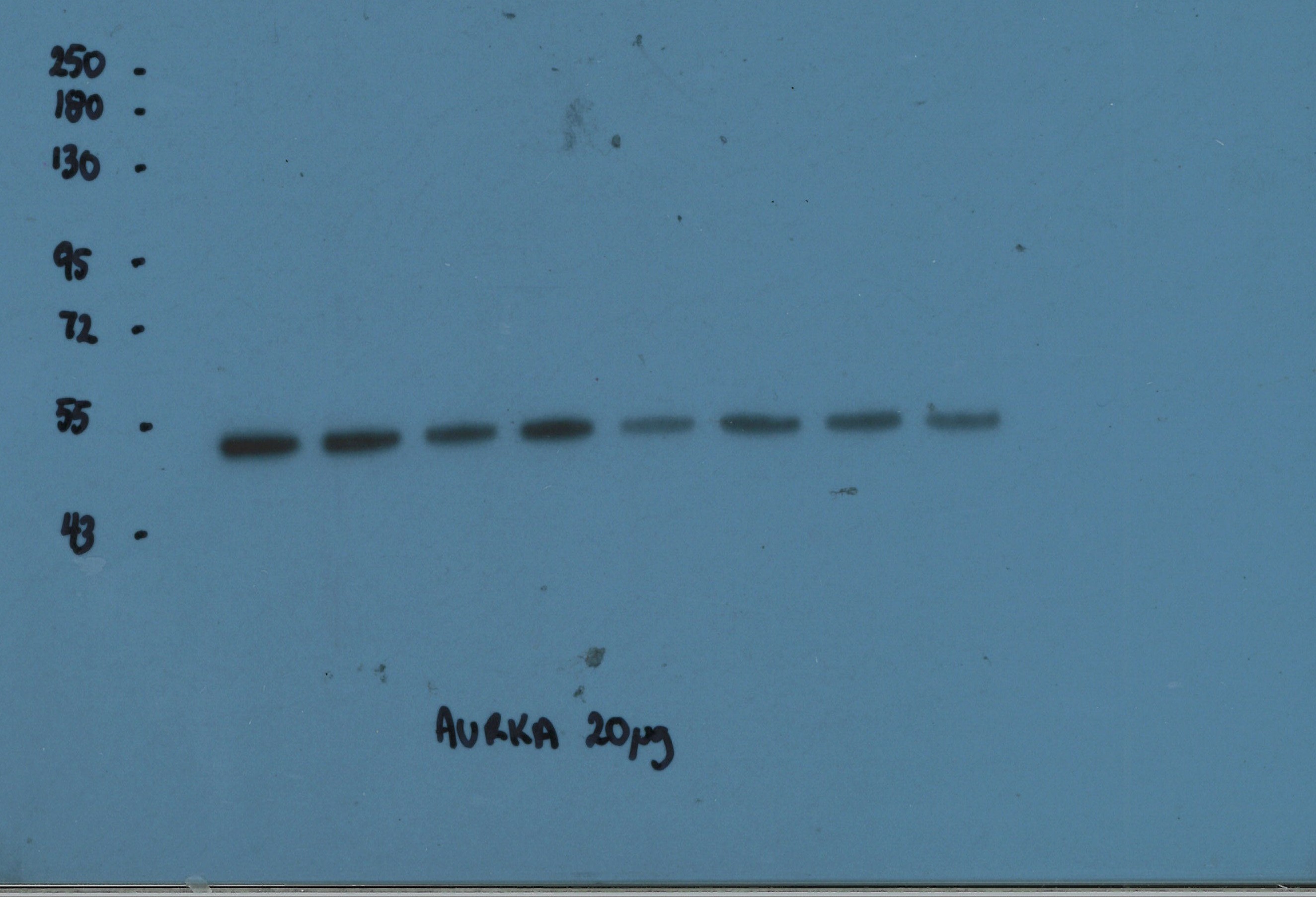

Supplement: Supplementary file 10 — Source data Fig. 7 [file 44319_2025_454_MOESM10_ESM.zip › Source Data - Figure 7/7H/AURKA.jpg]

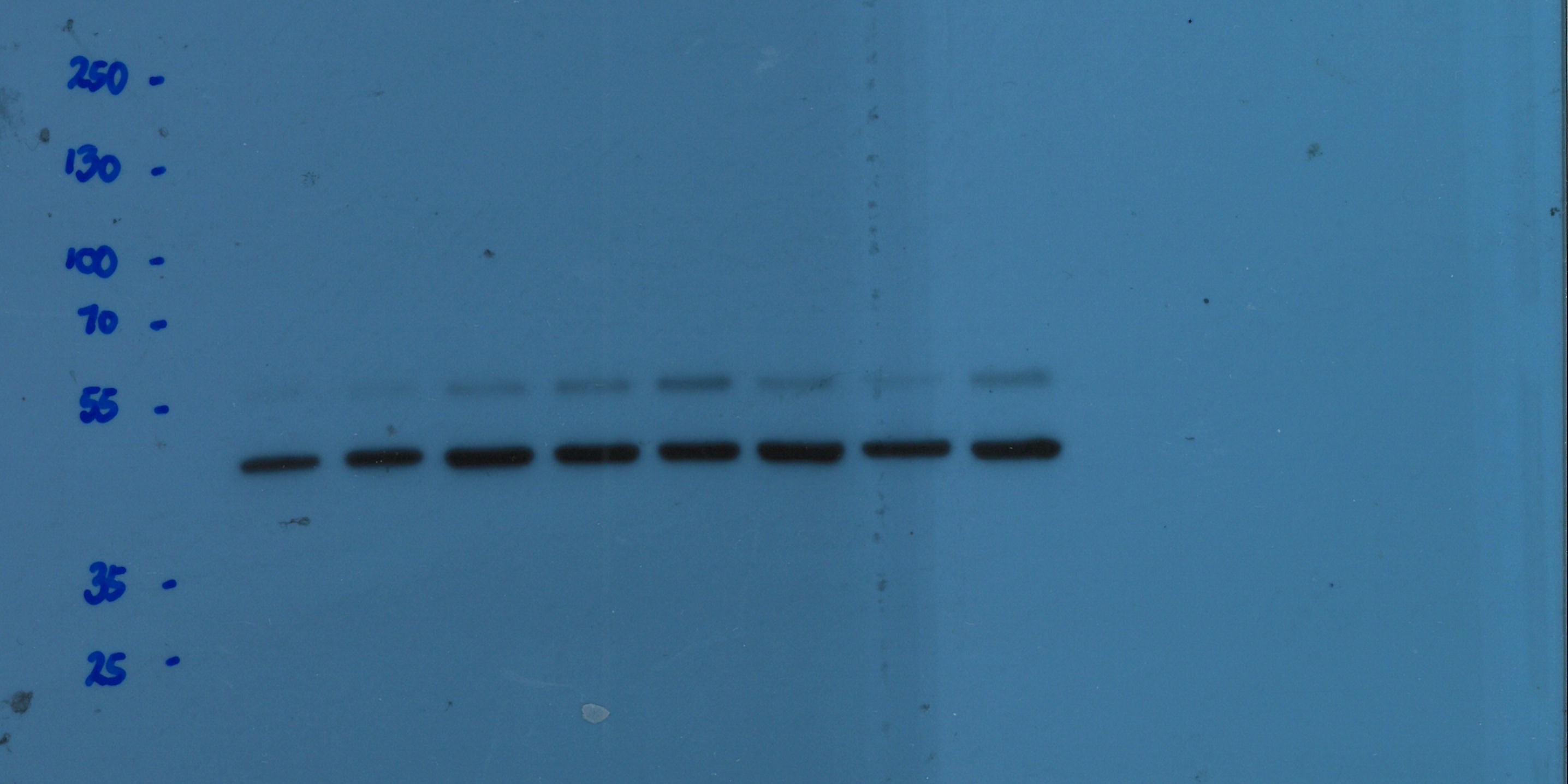

Supplement: Supplementary file 10 — Source data Fig. 7 [file 44319_2025_454_MOESM10_ESM.zip › Source Data - Figure 7/7I/AceTub beta-actin.jpg]

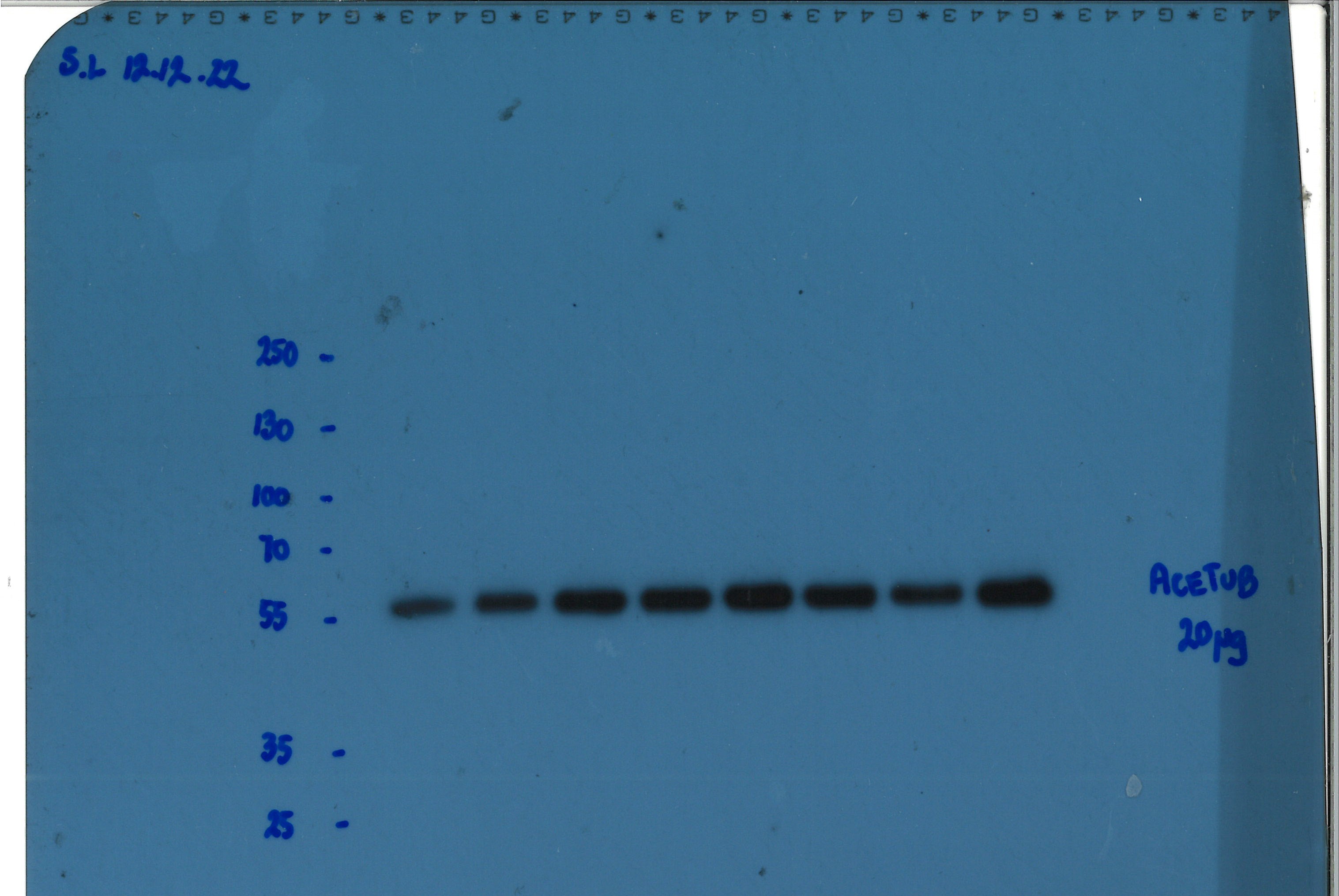

Supplement: Supplementary file 10 — Source data Fig. 7 [file 44319_2025_454_MOESM10_ESM.zip › Source Data - Figure 7/7I/AceTub.jpg]

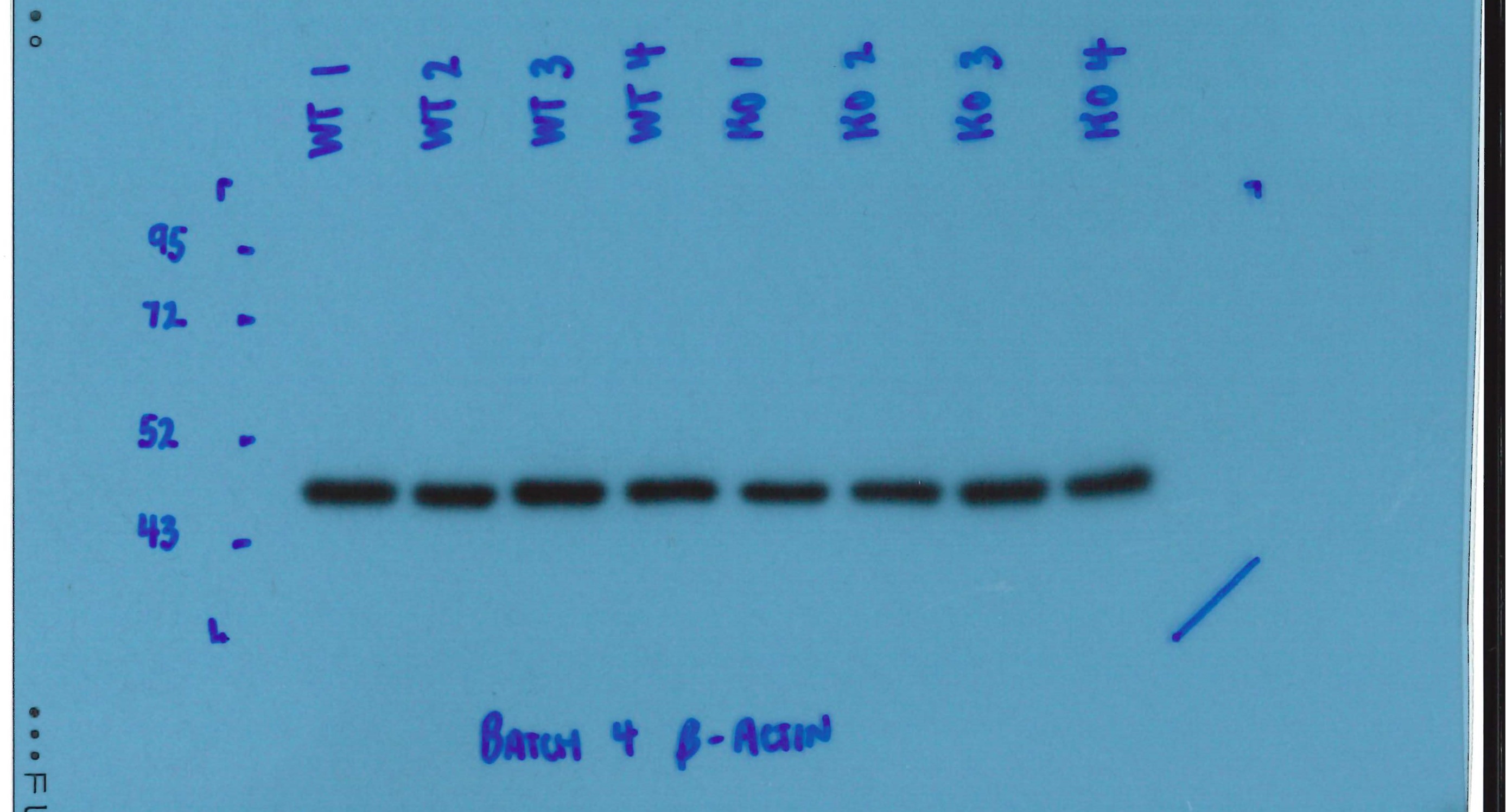

Supplement: Supplementary file 10 — Source data Fig. 7 [file 44319_2025_454_MOESM10_ESM.zip › Source Data - Figure 7/7J/CEP290 beta-actin.jpeg]

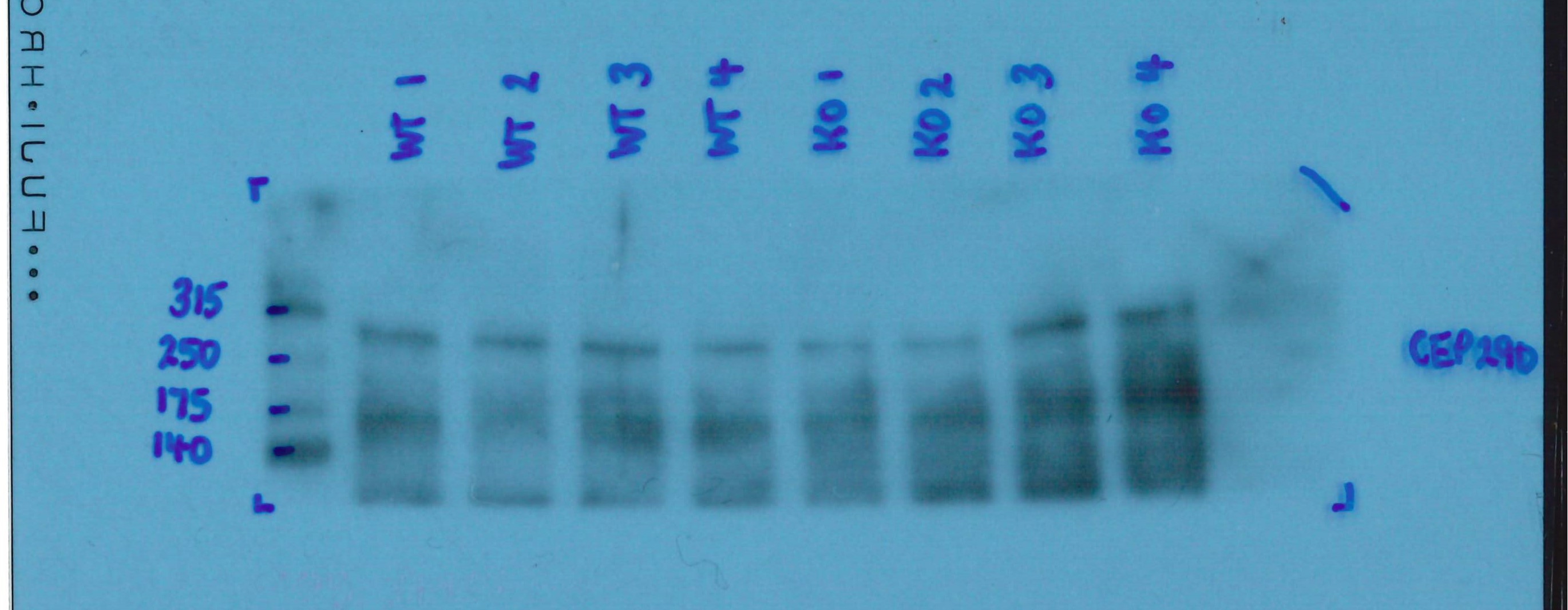

Supplement: Supplementary file 10 — Source data Fig. 7 [file 44319_2025_454_MOESM10_ESM.zip › Source Data - Figure 7/7J/CEP290.jpeg]

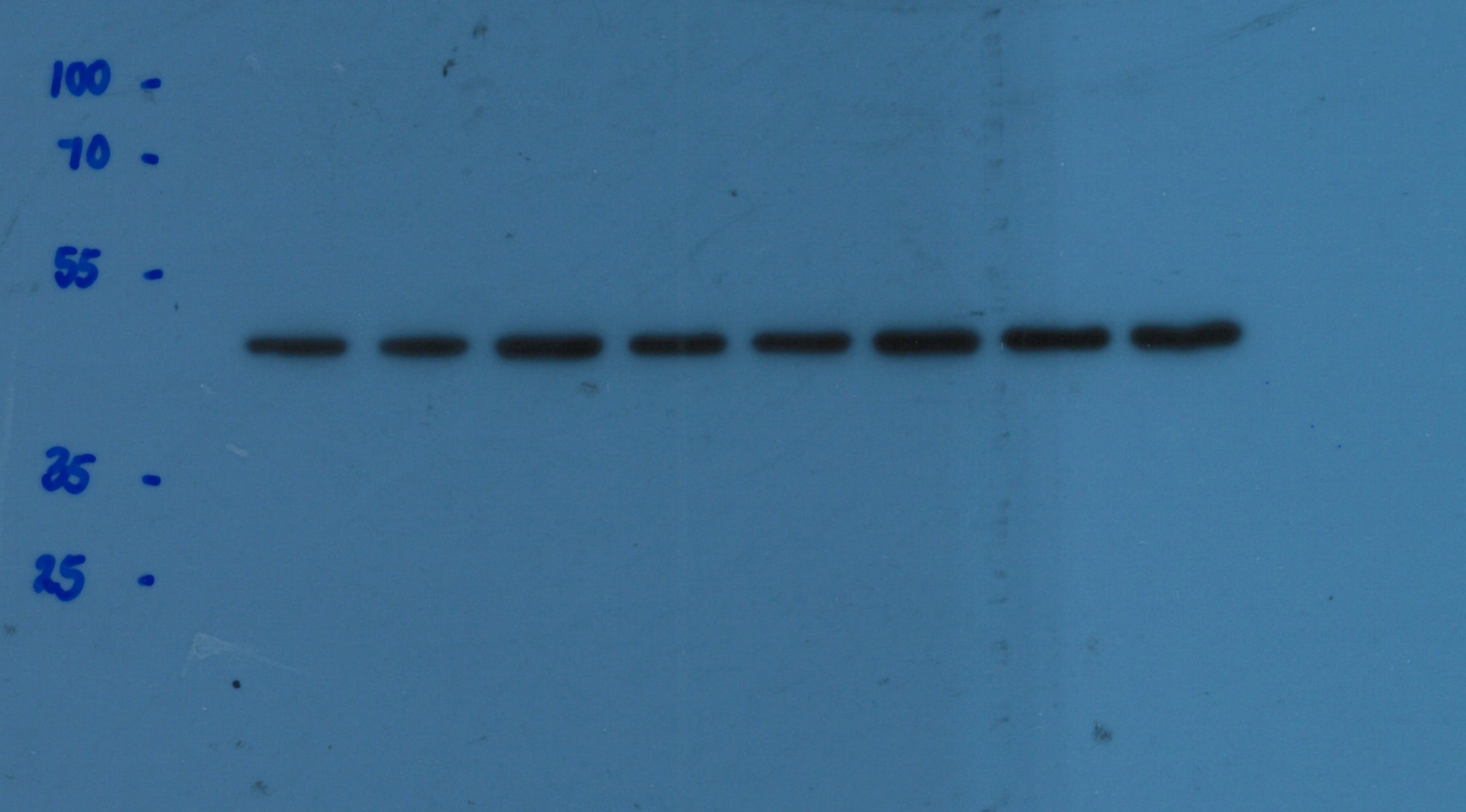

Supplement: Supplementary file 10 — Source data Fig. 7 [file 44319_2025_454_MOESM10_ESM.zip › Source Data - Figure 7/7K/IFT88 beta-actin.jpg]

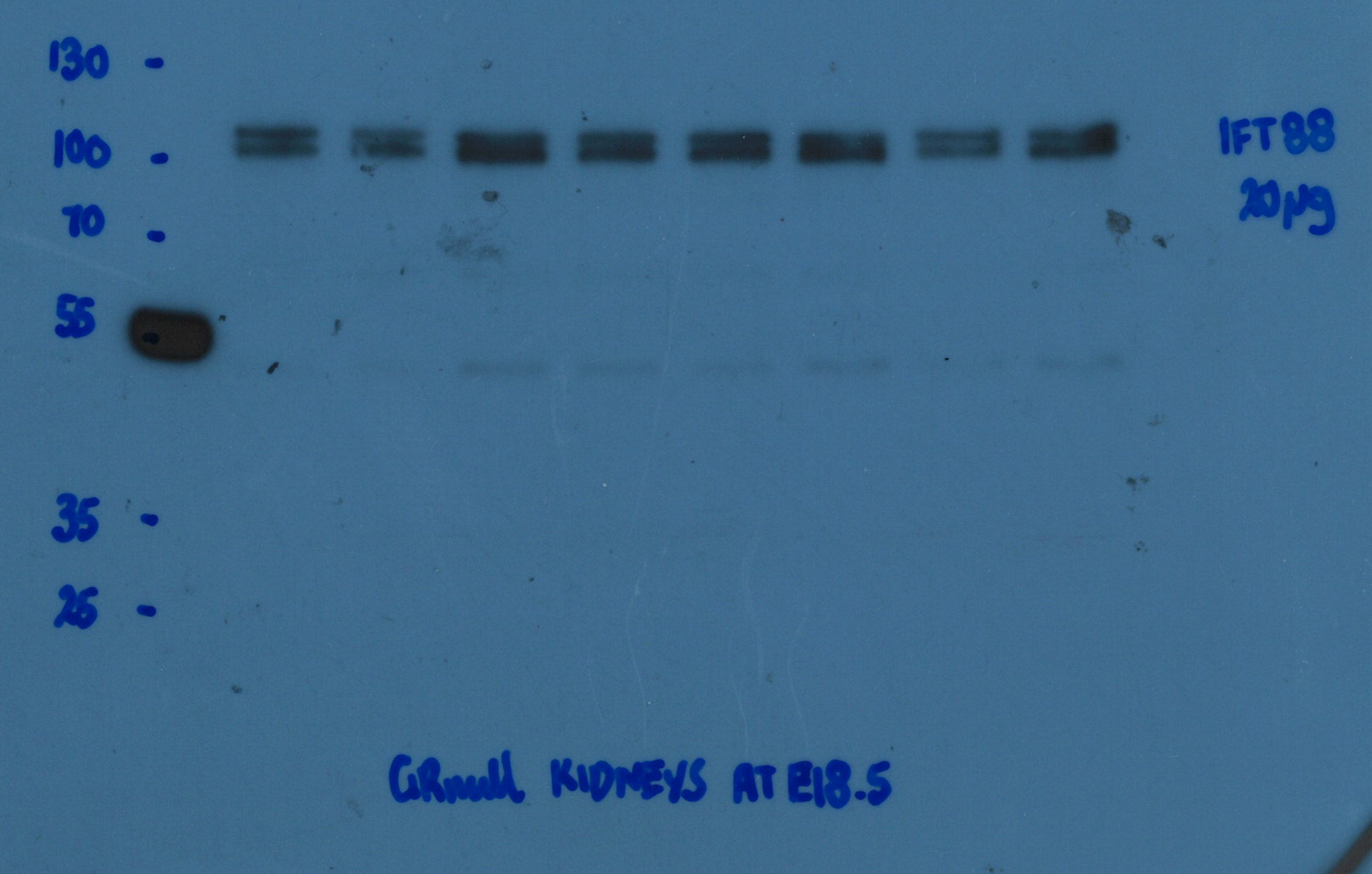

Supplement: Supplementary file 10 — Source data Fig. 7 [file 44319_2025_454_MOESM10_ESM.zip › Source Data - Figure 7/7K/IFT88.jpg]

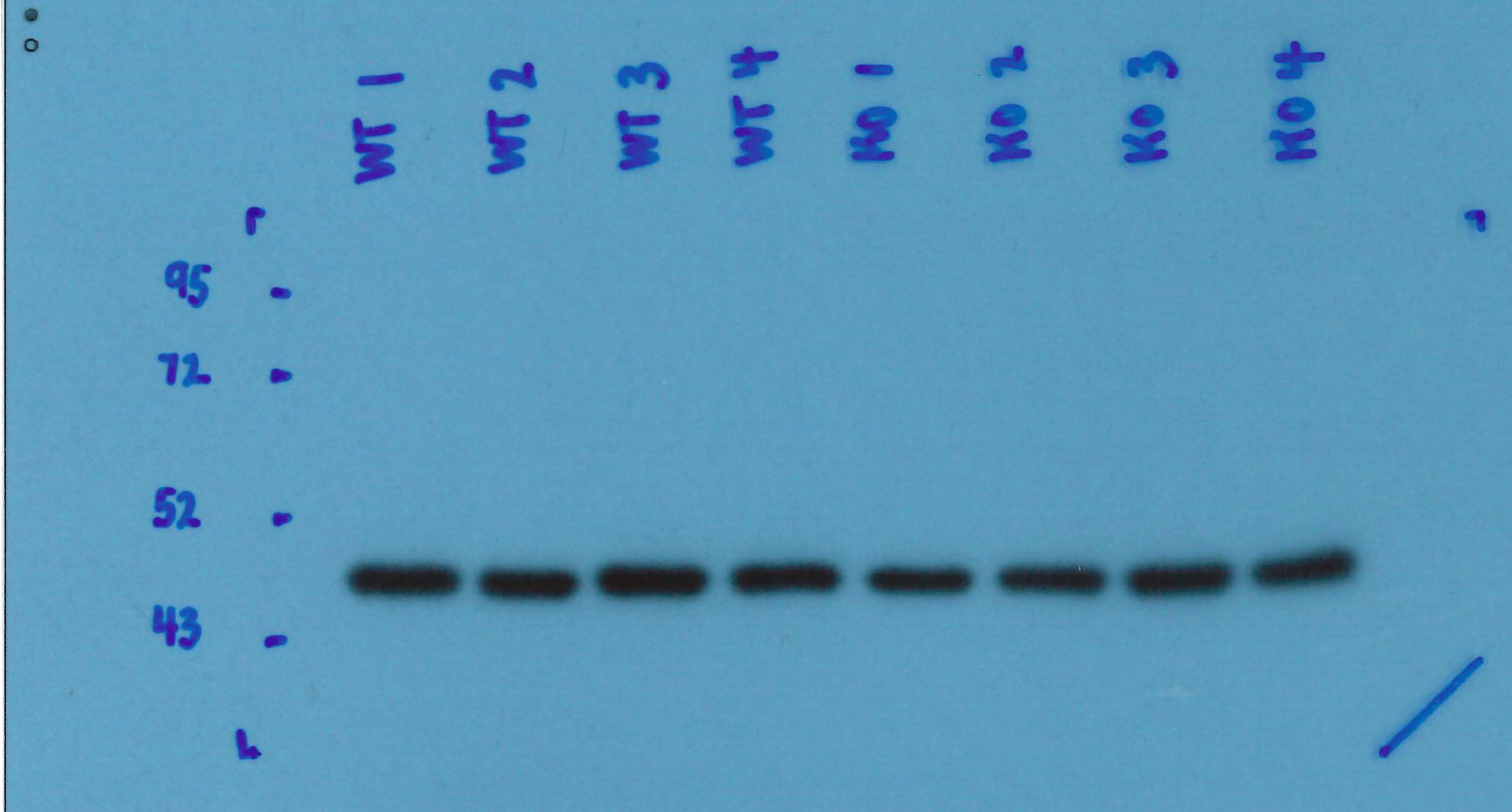

Supplement: Supplementary file 10 — Source data Fig. 7 [file 44319_2025_454_MOESM10_ESM.zip › Source Data - Figure 7/7L/KIF3A beta-actin.jpeg]

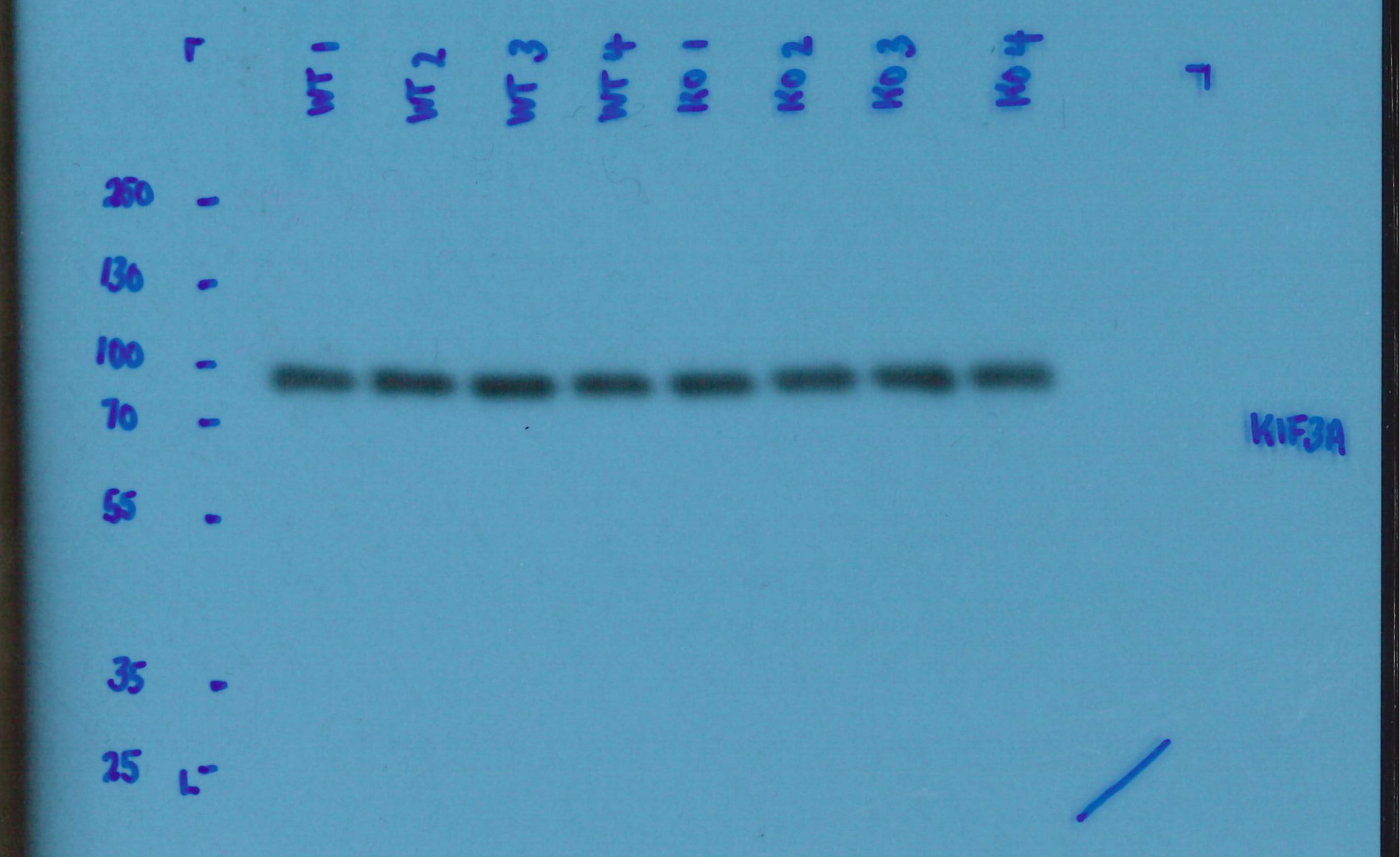

Supplement: Supplementary file 10 — Source data Fig. 7 [file 44319_2025_454_MOESM10_ESM.zip › Source Data - Figure 7/7L/KIF3A.jpeg]

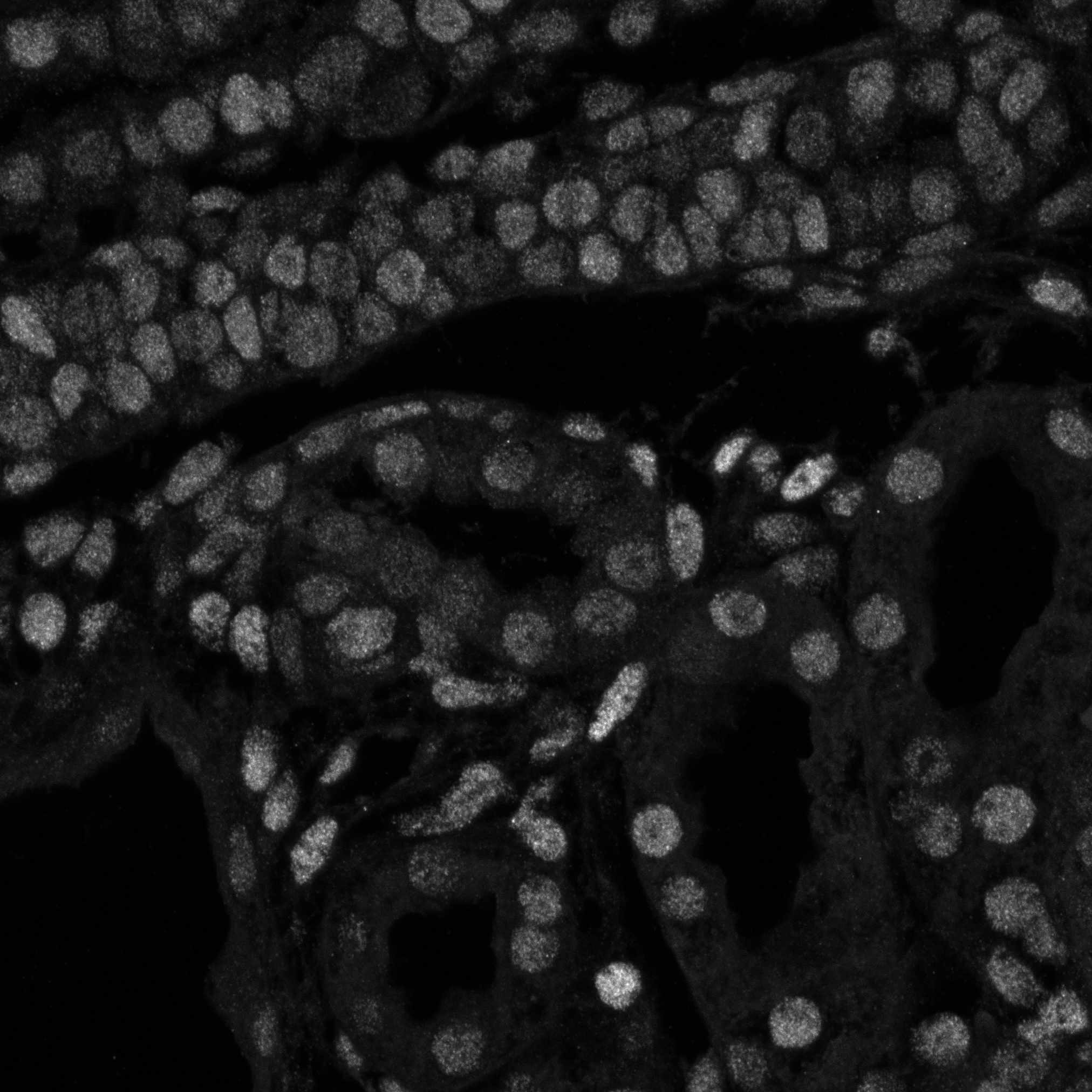

Supplement: Supplementary file 11 — Figure EV2 Source Data [file 44319_2025_454_MOESM11_ESM.zip › Source Data - Figure EV2/EV2A/Control hoescht, DBA, GR, LTL.tif]

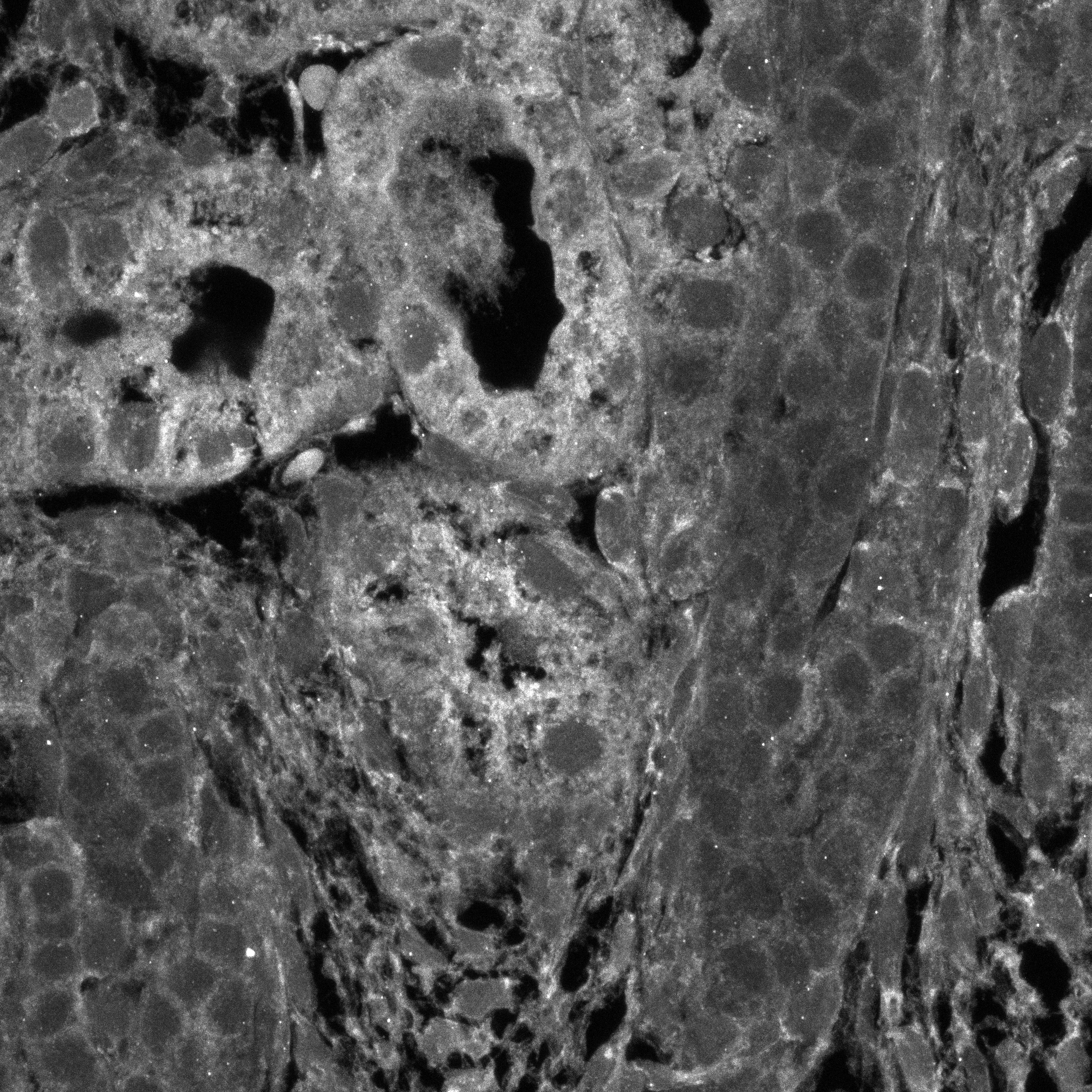

Supplement: Supplementary file 11 — Figure EV2 Source Data [file 44319_2025_454_MOESM11_ESM.zip › Source Data - Figure EV2/EV2A/GR-null hoescht, DBA, GR, LTL.tif]

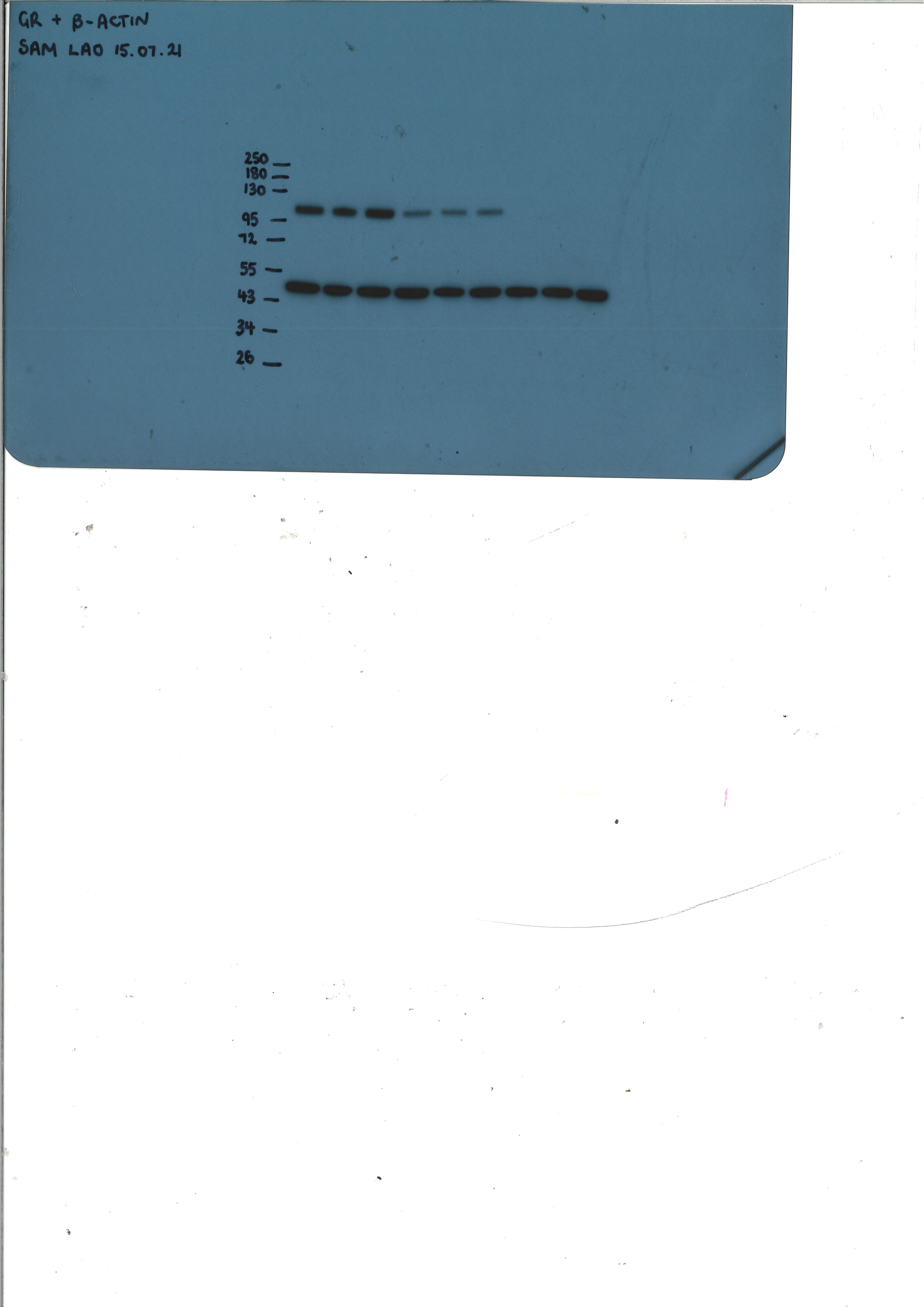

Supplement: Supplementary file 11 — Figure EV2 Source Data [file 44319_2025_454_MOESM11_ESM.zip › Source Data - Figure EV2/EV2B/GR and beta-actin.jpg]

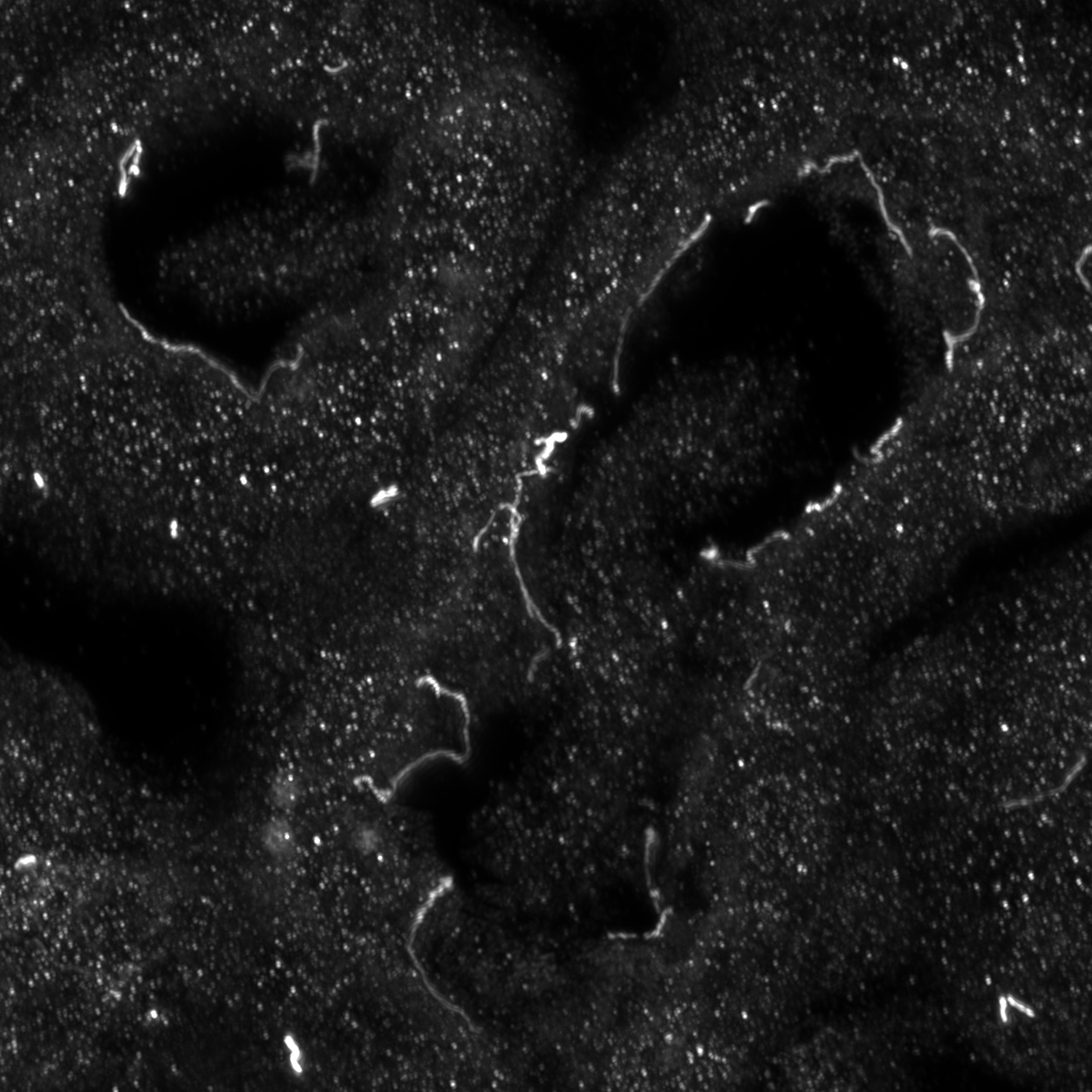

Supplement: Supplementary file 12 — Figure EV4 Source Data [file 44319_2025_454_MOESM12_ESM.zip › Source Data - Figure EV4/EV4A/Control hoescht, AceTub, ARL13B.tif]

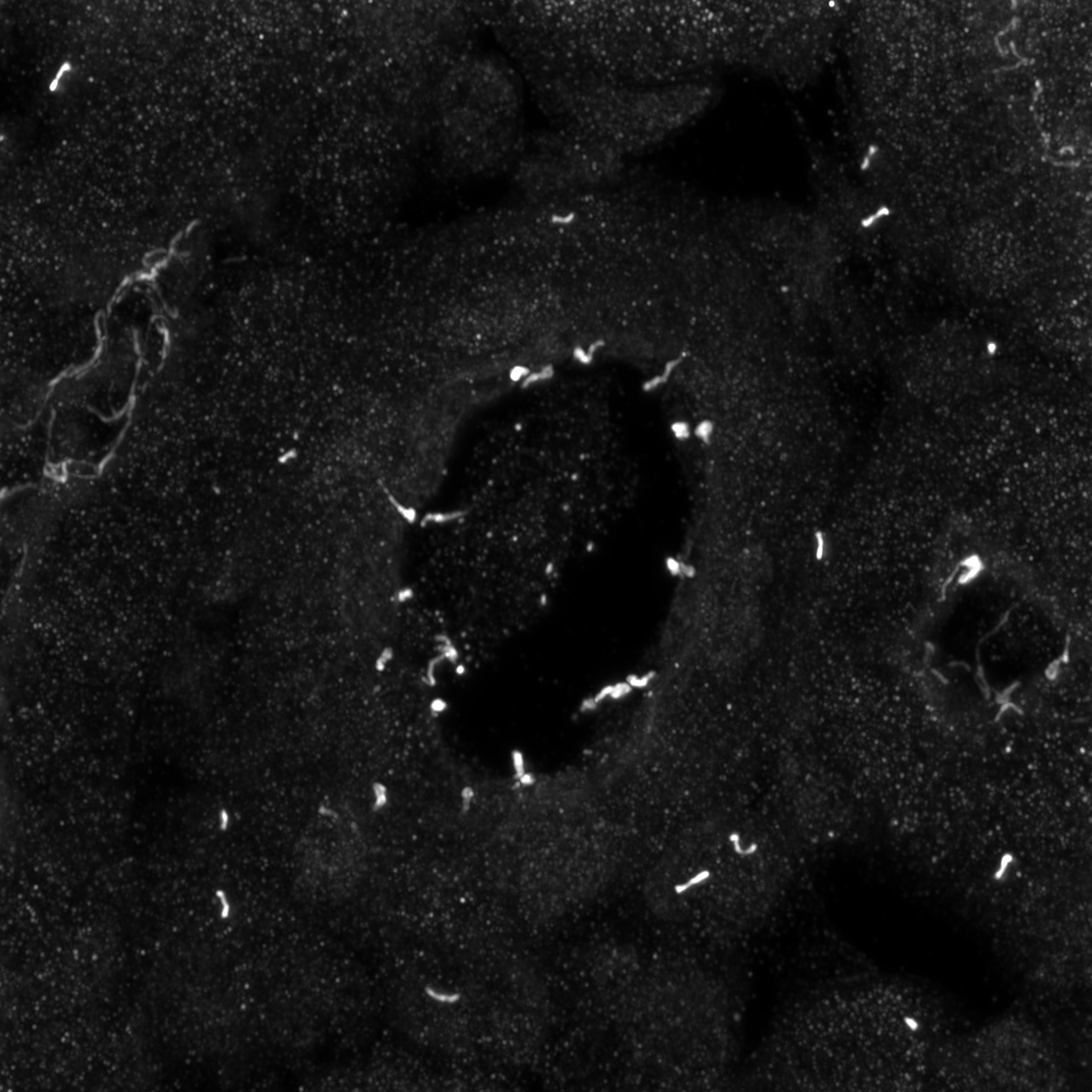

Supplement: Supplementary file 12 — Figure EV4 Source Data [file 44319_2025_454_MOESM12_ESM.zip › Source Data - Figure EV4/EV4A/GR-null hoescht, AceTub, ARL13B.tif]

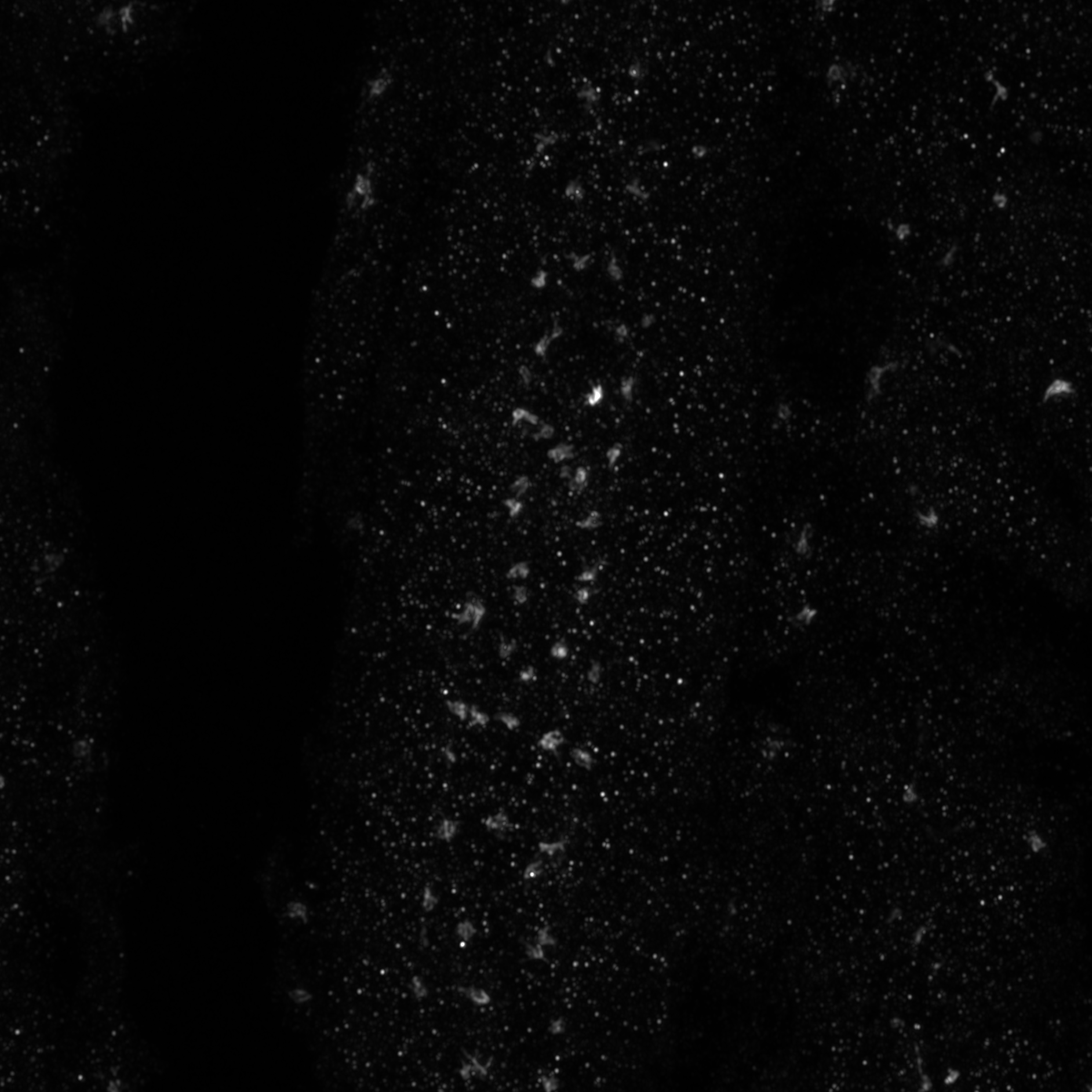

Supplement: Supplementary file 12 — Figure EV4 Source Data [file 44319_2025_454_MOESM12_ESM.zip › Source Data - Figure EV4/EV4B/Control hoescht, AceTub, DBA, PCNT.tif]

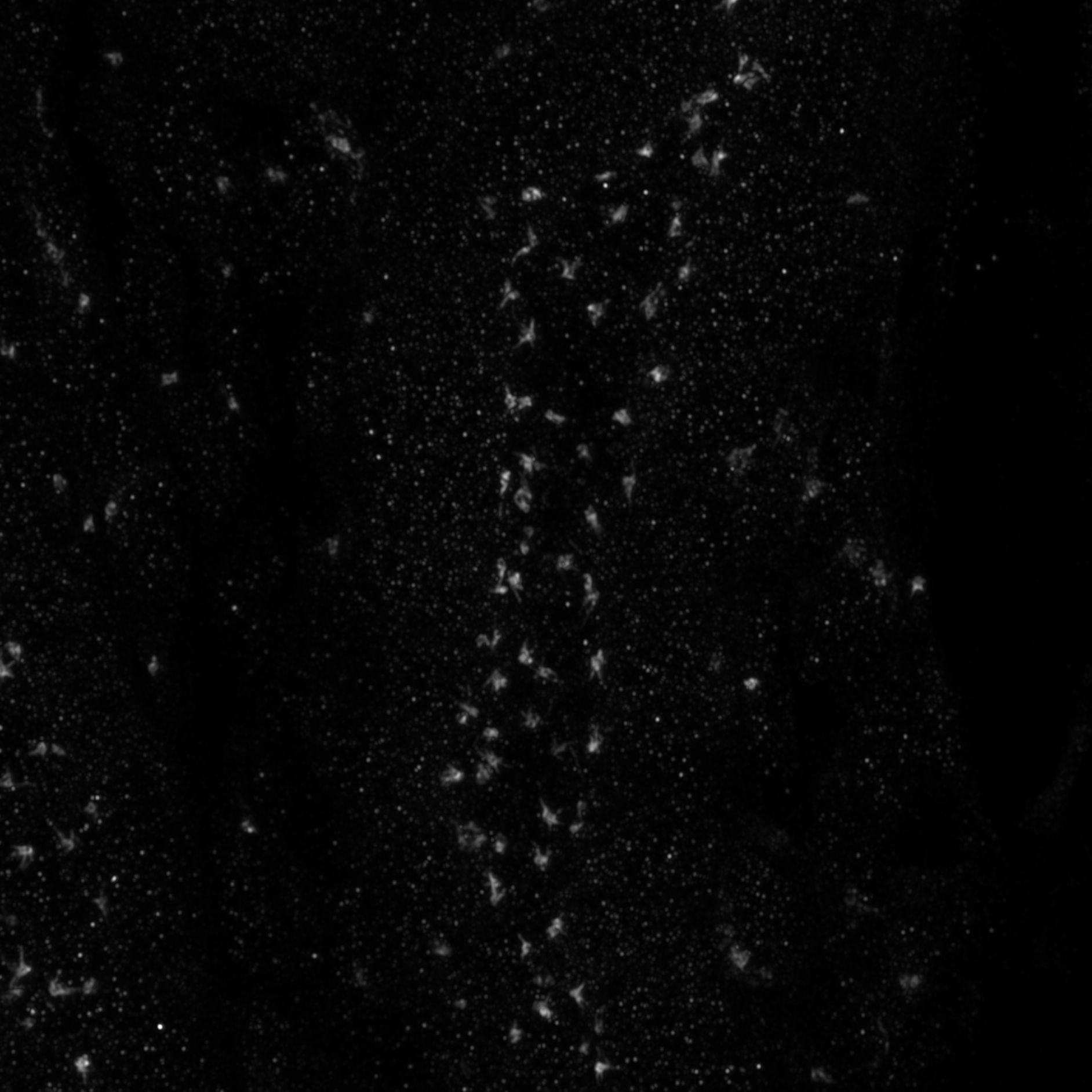

Supplement: Supplementary file 12 — Figure EV4 Source Data [file 44319_2025_454_MOESM12_ESM.zip › Source Data - Figure EV4/EV4B/GR-null hoescht, AceTub, DBA, PCNT.tif]

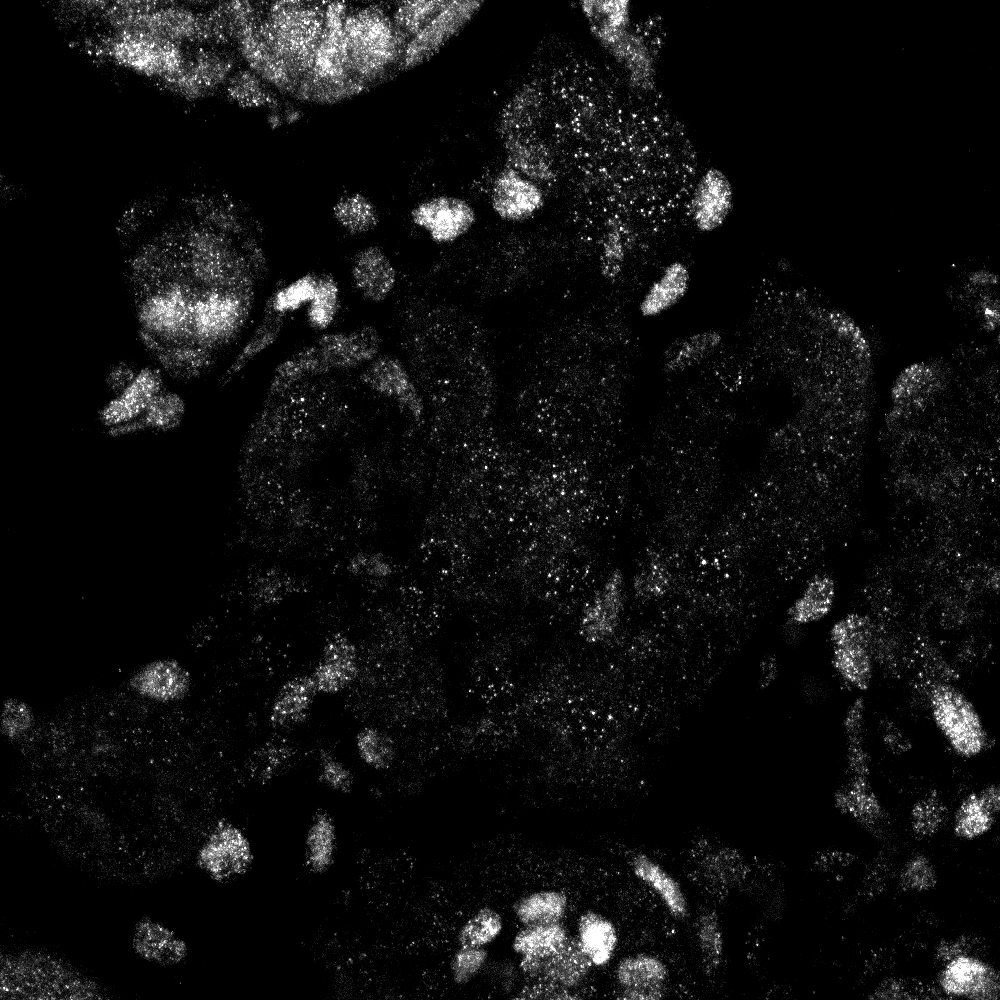

Supplement: Supplementary file 13 — Figure EV5 Source Data [file 44319_2025_454_MOESM13_ESM.zip › Source Data - Figure EV5/EV5A/Control hoescht, AceTub, KI67, LTL.tif]

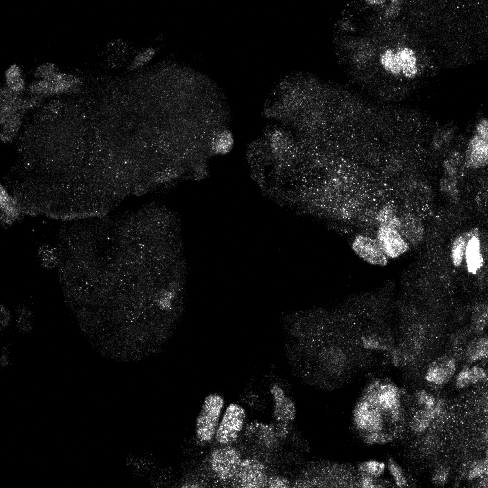

Supplement: Supplementary file 13 — Figure EV5 Source Data [file 44319_2025_454_MOESM13_ESM.zip › Source Data - Figure EV5/EV5A/GR-null hoescht, AceTub, KI67, LTL.tif]
