# Supplementary material for: Disrupted glucocorticoid receptor cell signalling causes a ciliogenesis defect in the fetal mouse renal tubule
Source: EMBO Rep. 2025 Apr 17;26(11):2883–909. doi: 10.1038/s44319-025-00454-0 (PMC12152183; doi:10.1038/s44319-025-00454-0)
Supplement: Supplementary file 14 — Expanded View Figures [file 44319_2025_454_MOESM14_ESM.pdf]

Expanded View Figures

Figure EV1. Single cell analysis and localisation of target gene expression in the fetal mouse kidney at E13.5, E15.5 and E18.5.

Expression of cell type-specific marker genes within single cell clusters at E13.5 (**A**), E15.5 (**B**) and E18.5 (**C**). Colour scale represents average expression, dot size represents percent of cells within cluster that express the gene.

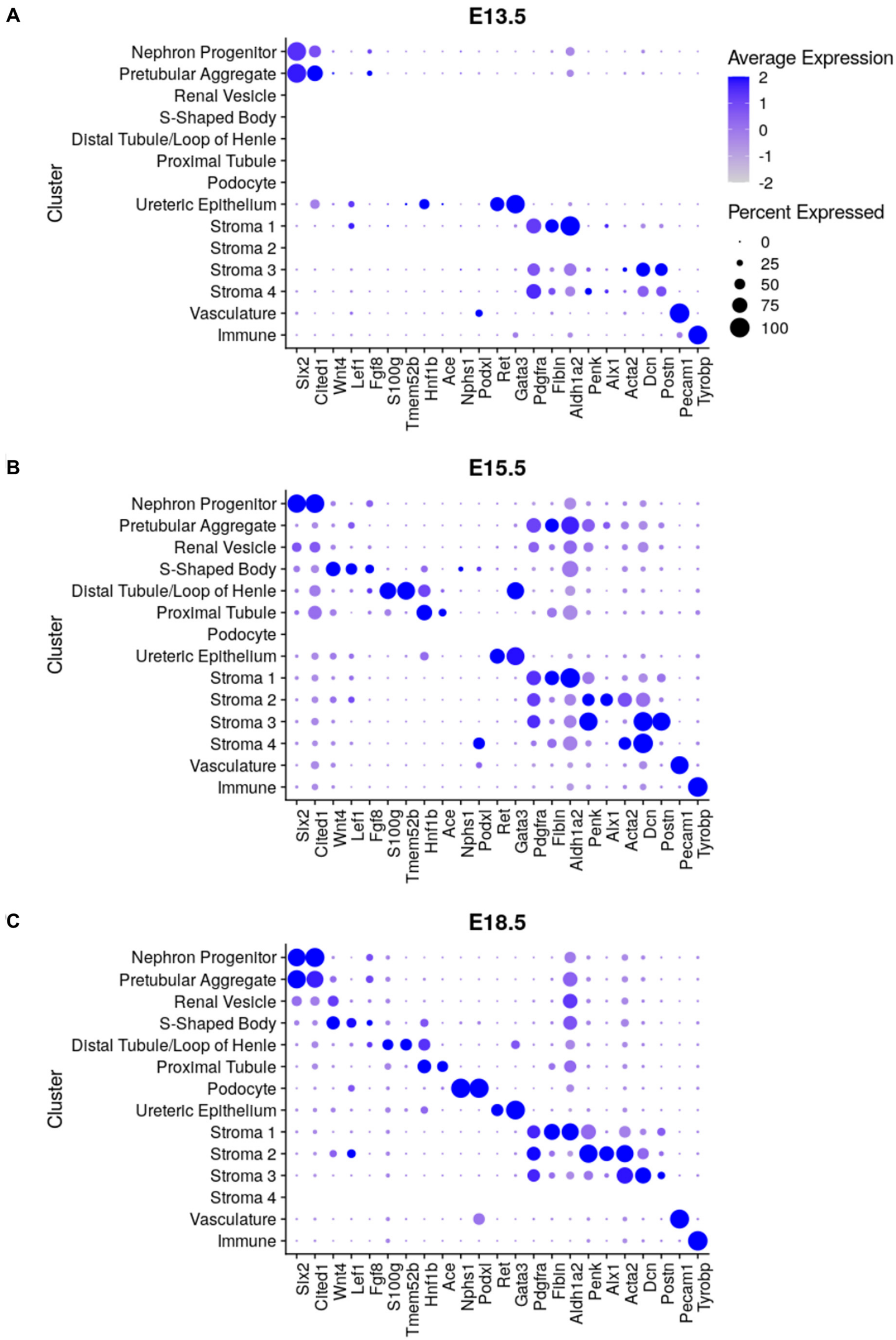
A
E13.5
B
E15.5
C
E18.5
Cluster
Nephron Progenitor
Pretubular Aggregate
Renal Vesicle
S-Shaped Body
Distal Tubule/Loop of Henle
Proximal Tubule
Podocyte
Ureteric Epithelium
Stroma 1
Stroma 2
Stroma 3
Stroma 4
Vasculature
Immune
Six2 Cited1 Wnt4 Lef1 Fgf8 S100g Tmem52b Hnf1b Ace Nphs1 Podxl Ret Gata3 Pdgfra Fbln Aldh1a2 Penk Alx1 Acta2 Dcn Postn Pecam1 Tyrobp
Average Expression
2
1
0
-1
-2
Percent Expressed
0
25
50
75
100

 © The Author(s)

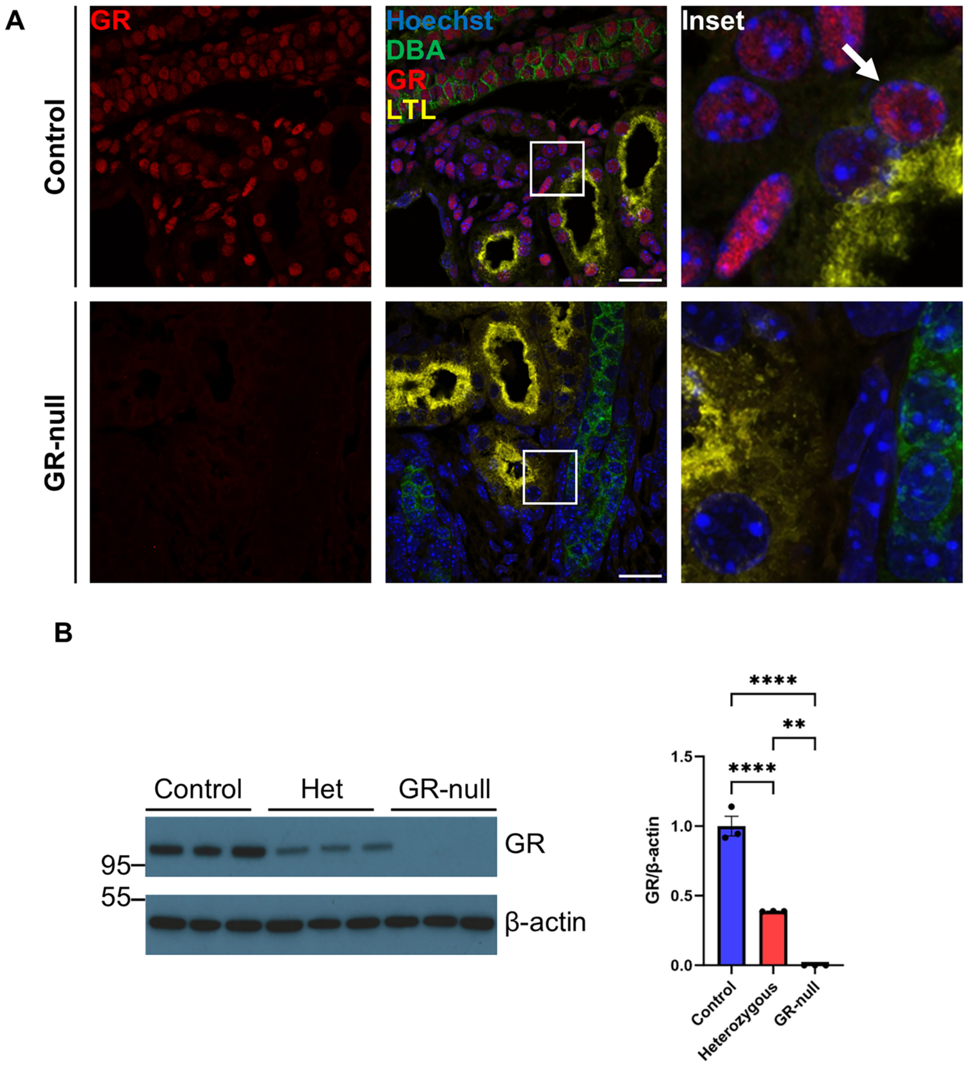

Figure EV2. Glucocorticoid receptor localisation and deletion in the GR-null fetal mouse kidney.

(**A**) Immunofluorescence of glucocorticoid receptor (GR) localisation and deletion in control and GR-null fetal kidney at E18.5. Sections were stained with Hoechst (blue, nucleus), Dolichos Biflorus Agglutinin (DBA) (green, collecting duct), GR (red, GR) and Lotus Tetragonolobus Lectin (LTL) (yellow, proximal tubule). White arrow indicates GR localisation. Slides were imaged with a Zeiss LSM 980 confocal microscopy (63x objective, 2x digital zoom), scale bar represents 20 µm. All images are representative of n = 4 animals per experimental group. (**B**) Western blot analysis of GR protein in GR-null fetal kidney at E18.5. All data presented as mean ± SEM, significant differences were analysed by one-way ANOVA with multiple comparisons indicated by $*P \leq 0.05$, $**P \leq 0.01$, $***P \leq 0.001$, $****P \leq 0.0001$, ns=not significant, between control, heterozygous (het) and GR-null. Control vs het ($P = 0.0001$), control vs GR-null ($P = 0.0001$), het vs GR-null ($P = 0.0012$). Data from $n = 3$ animals per experimental group. Source data are available online for this figure.

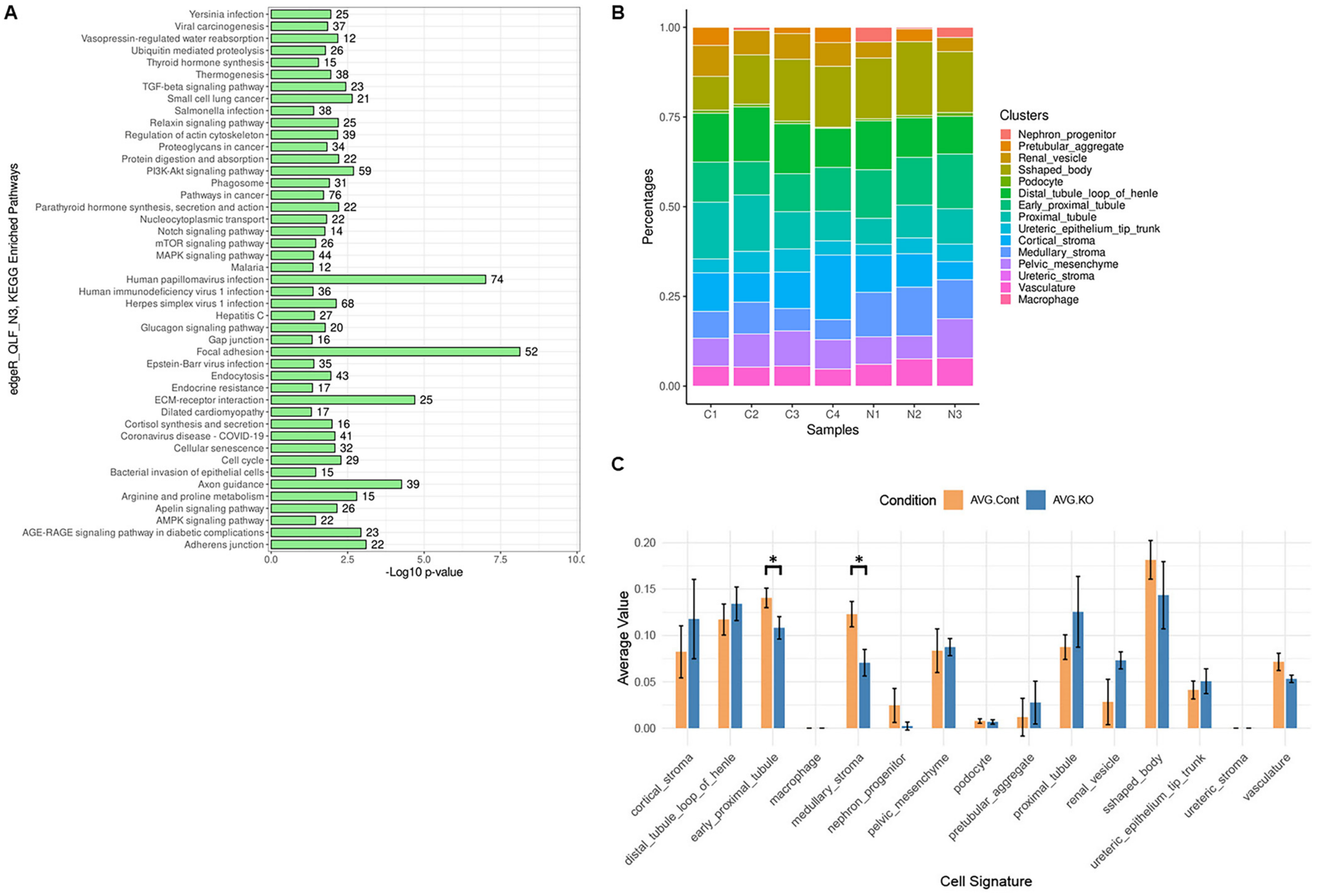

Figure EV3. Gene set enrichment analysis and RNA-seq deconvolution.

(**A**) Gene set enrichment analysis highlights impacted pathways between control and GR-null bulk RNA-seq datasets. RNA-seq was performed on total RNA isolated from control ($n = 4$) and GR-null ($n = 3$) mouse kidneys at E18.5. P value for each term is plotted on the x axis. (**B**) Bar plot of single cell deconvolution analysis performed to estimate changes in cell signatures in the bulk RNA-seq profiles of control (C1-4) and GR-null (N1-N3) samples. Cell type signatures based on the E18.5 single cell reference are colour coded as per the legend. Analysis of cell proportions between replicates from control and GR-null groups identified small but significant reductions in signatures related to the early proximal tubule ($P = 0.0143$) and medullary stroma ($P = 0.055$) in the GR-null group (two-tailed t test with unequal variance). (**C**) Bar plot of single cell deconvolution analysis performed to estimate changes in cell signatures in the bulk RNA-seq profiles of control (AVG. Cont, $n = 4$) and GR-null (AVG.KO, $n = 3$) samples. Bars illustrate the percentage average in each group tagged with standard error, significant differences were analysed by two-tailed t test with unequal variance, indicated by $*P \le 0.05$, $**P \le 0.01$, $***P \le 0.001$, $****P \le 0.0001$, ns=not significant. Analysis of cell proportions between replicates from control and GR-null groups identified small but significant reductions in signatures related to the early proximal tubule ($P = 0.0143$) and medullary stroma ($P = 0.055$) in the GR-null group.

 © The Author(s)

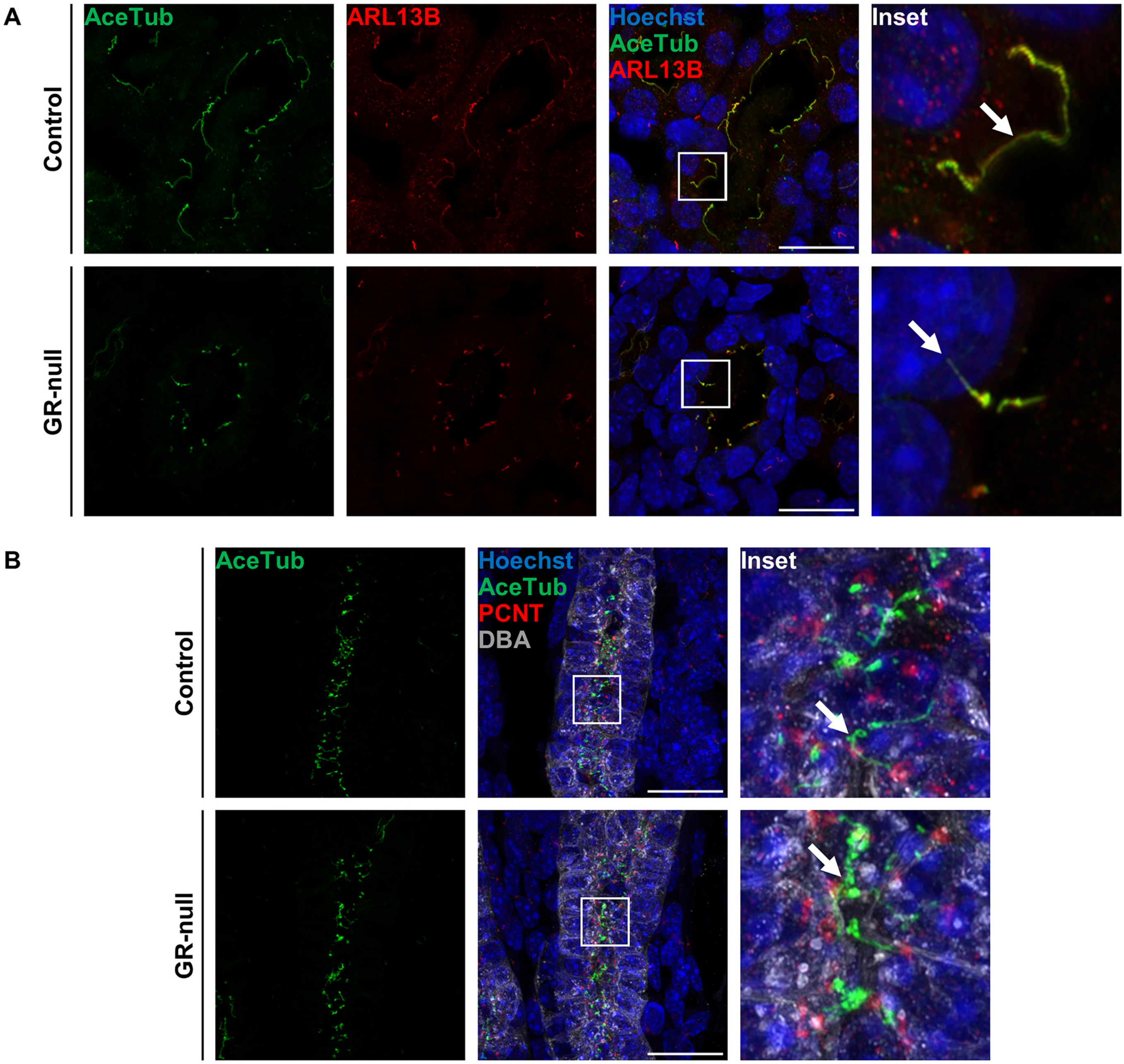

Figure EV4. Glucocorticoid regulation of primary cilia length in GR-null kidney at E18.5.

(**A**) Immunofluorescence of primary cilia morphology in control and GR-null fetal kidney at E18.5. Sections were stained with Hoechst (blue, nucleus), acetylated tubulin (AceTub) (green, microtubules) and ARL13B (red, cilia axoneme). White arrows indicate primary cilia morphology. Slides were imaged with a Zeiss LSM 980 confocal microscopy (×63 objective, 2× digital zoom), scale bar represents 20 µm. All images are representative of $n = 3$ animals per experimental group. (**B**) Immunofluorescence of primary cilia morphology in control and GR-null collecting ducts at E18.5. Sections were stained with Hoechst (blue, nucleus), acetylated tubulin (AceTub) (green, microtubules), pericentrin (PCNT) (red, basal body) and Dolichos Biflorus Agglutinin (DBA) (grey, collecting duct). White arrows indicate primary cilia morphology. Slides were imaged with a Zeiss LSM 980 confocal microscope (×63 objective, 2× digital zoom), scale bar represents 20 µm. All images are representative of $n = 4$ animals per experimental group. Source data are available online for this figure.

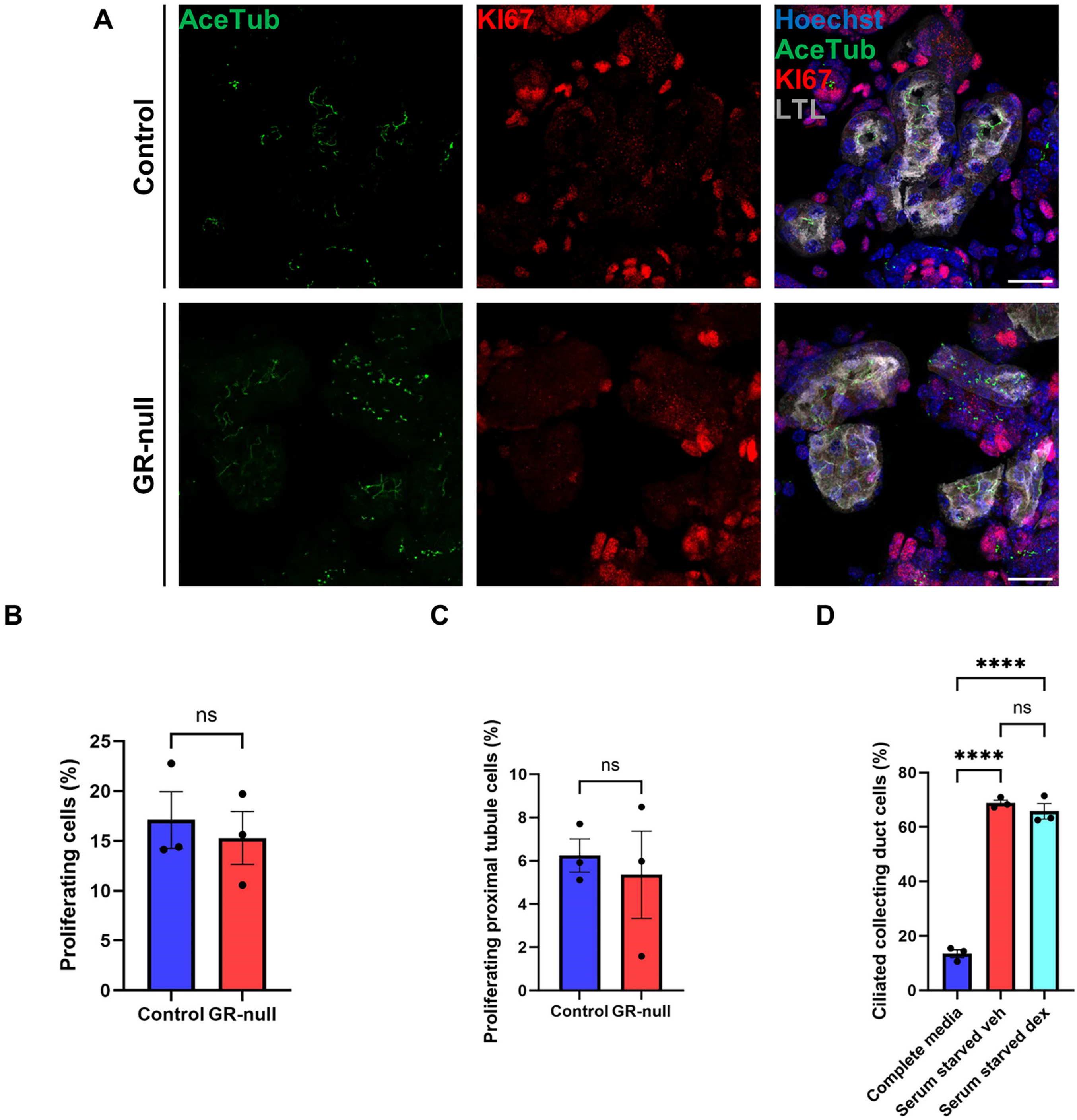
A
AceTub
KI67
Hoechst
AceTub
KI67
LTL
Control
GR-null
B
ns
Proliferating cells (%)
25
20
15
10
5
0
Control
GR-null
C
ns
Proliferating proximal tubule cells (%)
10
8
6
4
2
0
Control
GR-null
D
****
ns
****
Ciliated collecting duct cells (%)
80
60
40
20
0
Complete media
Serum starved veh
Serum starved dex

© The Author(s)

Figure EV5. Cell proliferation localisation in the GR-null fetal mouse kidney proximal tubule cells at E18.5 and primary cilia expression in IMCD3 cells after serum starved media conditions.

(**A**) Immunofluorescence of proliferation localisation in control and GR-null fetal kidney at E18.5. Sections were stained with Hoechst (blue, nucleus), acetylated tubulin (AceTub) (green, microtubules), KI67 (red, proliferative cells) and Lotus Tetragonolobus Lectin (LTL) (grey, proximal tubule). White arrow indicates proliferative cells. Slides were imaged with a Zeiss LSM 980 confocal microscopy (×63 objective, 2× digital zoom), scale bar represents 20 µm. All images are representative of $n = 4$ animals per experimental group. (**B**) Percentage of proliferating cells. All data presented as mean ± SEM, significant differences were analysed by unpaired T tests indicated by $^*P \le 0.05$, $^{**}P \le 0.01$, $^{***}P \le 0.001$, $^{****}P \le 0.0001$, ns=not significant, between control and GR-null ($P = 0.67$), $n = 3$ animals per experimental group. (**C**) Percentage of proliferating proximal tubule cells. All data presented as mean ± SEM, significant differences were analysed by unpaired T tests indicated by $^*P \le 0.05$, $^{**}P \le 0.01$, $^{***}P \le 0.001$, $^{****}P \le 0.0001$, ns=not significant, between control and GR-null ($P = 0.70$), $n = 3$ animals per experimental group. (**D**) Percentage of cells with a primary cilium in complete media versus serum starved vehicle (veh) and serum starved dexamethasone (dex) in IMCD3 cells. All data presented as mean ± SEM, significant differences were analysed by one-way ANOVA with multiple comparisons indicated by $^*P \le 0.05$, $^{**}P \le 0.01$, $^{***}P \le 0.001$, $^{****}P \le 0.0001$, ns=not significant, between complete media, serum starved veh and serum starved dex. Complete media vs serum starved veh ($P = 0.0001$), complete media vs serum starved dex ($P = 0.0001$), serum starved veh vs serum starved dex ($P = 0.54$). Data from $n = 3$ biological replicates per experimental group. Source data are available online for this figure.

© The Author(s)
